# Synthesis of Arylamines via Aminium Radicals

**DOI:** 10.1002/anie.201708693

**Published:** 2017-10-24

**Authors:** Thomas D. Svejstrup, Alessandro Ruffoni, Fabio Juliá, Valentin M. Aubert, Daniele Leonori

**Affiliations:** ^1^ School of Chemistry University of Manchester Oxford Road Manchester M13 9PL UK

**Keywords:** aminium radicals, aryl amines, late-stage amination, N-arylation, synthetic methods

## Abstract

Arylamines constitute the core structure of many therapeutic agents, agrochemicals, and organic materials. The development of methods for the efficient and selective construction of these structural motifs from simple building blocks is desirable but still challenging. We demonstrate that protonated electron‐poor O‐aryl hydroxylamines give aminium radicals in the presence of Ru(bpy)_3_Cl_2_. These highly electrophilic species undergo polarized radical addition to aromatic compounds in high yield and selectivity. We successfully applied this method to the late‐stage modification of chiral catalyst templates, therapeutic agents, and natural products.

N,N‐Dialkyl arylamines are a privileged scaffold found in blockbuster drugs, agrochemicals, and organic materials (Scheme 1 A).^1^ These molecular frameworks are usually assembled through Pd^0^‐ or Cu^I/II^‐catalysed cross‐couplings of amine nucleophiles and aryl halides (i.e., Ullmann^2^ and Buchwald–Hartwig^3^ coupling reactions) or arylboronic acids (i.e., Chan–Lam coupling^4^).^5^ However, these approaches require the use of sometimes expensive catalysts, forcing reaction conditions, and pre‐functionalized aromatics. This latter aspect can be problematic when the aromatic partner is difficult to make or the introduction of halides or B functionalities suffers from directionality issues (*ortho* vs. *meta* vs. *para*). As such, methods for the direct amination of unfunctionalized aromatic compounds are very desirable but far from being general.^6^

**Scheme 1:**
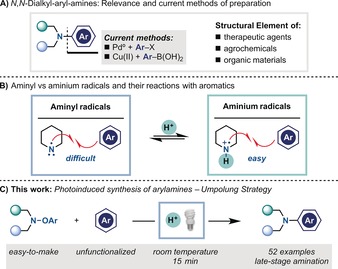
Aryl amines and aminium radicals.

Nitrogen radicals^7^ are highly reactive species that are now witnessing a resurgence of synthetic interest owing to the ability of photoredox catalysis^8^ to promote single‐electron transfer (SET)^9^ processes under mild conditions.^10^ While highly electrophilic amidyl^11^ and sulfamidyl radicals^12^ have been successfully coupled with highly electron‐rich aromatic compounds (e.g., indole, pyrrole), the use of dialkyl‐substituted nitrogen radicals (aminyl radicals) in related arylations has yet to be described. This lack of synthetic application can be explained by the intrinsic nucleophilic nature of aminyls, which causes repulsive interactions between their lone pair and the aromatic ring.^13^ However, upon protonation, aminyl radicals are converted into aminium radicals10d,10i, 14 that are isoelectronic to alkyl radicals but carry a formal positive charge (Scheme 1 B). This makes them powerful electrophiles that undergo highly polarized radical processes. Indeed, pioneering work from Minisci and co‐workers showed that N−Cl amines can be arylated upon photochemical N−Cl bond homolysis.^15^ While successful, these reactions have not been employed in mainstream organic synthesis owing to three main limitations: 1) the aromatic compound is a co‐solvent (10–20 equiv); 2) high‐energy light (*λ*<280 nm) is required and 3) the reactions are run in refluxing AcOH/H_2_SO_4_.15c

We recently developed a visible‐light‐mediated synthesis of iminyl^16^ and amidyl11c radicals through reductive SET fragmentation of electron‐poor O‐aryl oximes and aryloxyamides. We envisaged that such an approach might have enabled access to aminium radicals for direct aromatic amination. In this paper we describe our work in the area that has resulted in a powerful method for the fast construction of aryl amines (Scheme 1 C).

At the outset, we envisioned a strategy relying on our reductive SET approach for nitrogen‐radical generation in the presence of a Brönsted acid (Scheme 2 A). Depending on the p*K*_a_ of the acid, two mechanistic pathways are possible. In the case of a weak acid, SET reduction and fragmentation of electron‐poor O‐aryl hydroxylamine **A** would generate the aminyl radical **B**, which upon protonation would give the key aminium radical **C**, ready for intermolecular arylation (Path a). Since aminyl and aminium radicals are short‐lived (*τ*_0_≈1–10 μs)^17^ and undergo fast H‐atom abstraction,^18^ a better approach would rely in the use of a strong acid that is able to protonate **A** to give the ammonium salt **E** (Path b). This initial protonation intrinsically leads to many advantages: 1) SET reduction of **E** is expected to be more facile,^19^ 2) the formation of aminyl **B** is by‐passed, 3) the *τ*_0_ of **C** will be enhanced13c and 4) the strong acid will maintain the N atom in protonated form during the entire reaction sequence, thereby insulating arylamine **D** from SET oxidation (decomposition) and/or further amination (over reactivity). Furthermore, since **E** is expected to be the strongest electrophore in the system, any potential SET oxidation of the aromatic partner^20^ will be minimised. Overall, this approach would represent an umpolung alternative to the photoredox amination methods developed by Nicewicz20a and Lei,20b where nucleophilic pyrazoles react with aromatic radical cations.

**Scheme 2:**
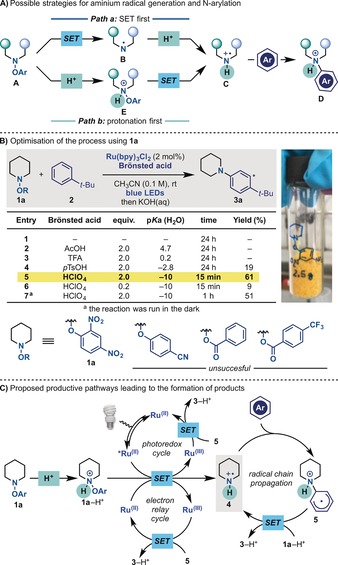
Possible mechanistic pathways, reaction optimization, and proposed mechanistic analysis.

This strategy was evaluated using piperidine **1 a** (*E* ^red^=−0.9 V vs. SCE in CH_3_CN) prepared in one step from commercial materials on a multigram scale,^21^ *t*‐Bu‐benzene **2**, and Ru(bpy)_3_Cl_2_ as the photoredox catalyst in CH_3_CN under blue‐LED irradiation (450 nm; Scheme 2 B). Without Brönsted acid or in the presence of AcOH or TFA, no product was obtained along with quantitative recovery of **1 a** and **2** (entries 1–3). To our delight, when using *p*TsOH, **3 a** was formed in 19 % yield (3:1 *para*/*meta*; entry 4), and further evaluation revealed that HClO_4_ (70 % in H_2_O) exhibited optimal conversion in just 15 min at room temperature (entry 5). Other aminium radical precursors with different aromatic substitution patterns were evaluated but they provided **3 a** in very low yields (if any), leaving **1 a** as the optimum electrophore for this arylation strategy.^21^ In line with our working hypothesis, 1) the acid could not be used in sub‐stoichiometric amounts (entry 6) and 2) ^1^H NMR studies revealed that only *p*TsOH and HClO_4_ led to protonation of **1 a**.^21^ Control experiments confirmed the requirement for Ru(bpy)_3_Cl_2_ but blue‐LED irradiation was not necessary to observe product formation (entry 7).^22^ However, higher yields were consistently obtained when the reactions were run under continuous irradiation during the scope evaluation.^23^ This suggests a complex interplay of photochemical and “dark” pathways cooperating for the formation of the reaction product. For example, **1 a**‐H^+^ could undergo SET reduction from the visible‐light‐excited *Ru^II^ to generate the highly electrophilic piperidinium radical **4** (calculated electrophilicity index, *ω*_rc_^+^=13.0;^21^ Scheme 2 C). Polarized radical amination would form the carbon radical **5** that can deliver the protonated product **3**‐H^+^ by 1) closing the photoredox cycle or 2) reacting in a propagative manner with **1 a**‐H^+^. In order to evaluate the feasibility of the latter pathway, we studied by DFT the key step in the propagation cycle (**5**+**1 a**‐H^+^→**4**+**3**‐H^+^) and found it to be exergonic (Δ*G*°=−15.2 kcal mol^−1^).^21^ The observed “dark” reactivity might arise from the ability of ground‐state Ru(bpy)_3_Cl_2_ (*E*_1/2_^ox^=+1.29 V vs. SCE in CH_3_CN)^24^ to promote the SET reduction of **1 a**‐H^+^ and act as an electron‐relay^25^ catalyst/radical‐chain initiator.^21^ Indeed, we were able to detected the formation of Ru^III^ by UV/Vis spectroscopy in stoichiometric “dark” experiments. We believe that upon protonation, **1 a**‐H^+^ becomes a powerful oxidant^19^, ^26^ (calculated^27^ Ered(1a-H+) =+1.7 V vs. SCE) and might be responsible for this unforeseen reactivity.^21^

With optimal conditions in hand, we examined the aromatic partner scope using piperidine **1 a** (Scheme 3). Pleasingly, the reaction displayed broad applicability and a series of differentially substituted benzene derivatives were successfully employed. The high electrophilicity of aminium radicals is advantageous since it enables reaction with weakly (**3 a**–**d**) as well as strongly (**3 e**–**j**) electron‐rich aromatic partners. In the case of monosubstituted benzenes, reaction occurred preferentially at the *para* position, thus underlining the importance of polar and steric effects in the radical amination step. This selectivity can be rationalised by computing the Fukui indices^28^ for the aromatic partners.^21^ Halogenated anisoles (**3 k**,**l**), benzodioxolane (**3 m**), and chromanone (**3 n**) also underwent amination in useful yields. Polycyclic aromatic compounds reacted very well, and we expanded the chemistry to the selective amination of naphthalene (**3 o**), 2‐methoxy (**3 p**), 2‐bromo‐ (**3 q**), and 2‐acetyl‐ (**3 r**) naphtalene, as well as 1‐bromo‐ (**3 s**), 1‐chloro‐, (**3 t**) and 1‐cyano‐ (**3 u**) naphtalene. The successful implementation of halogen‐containing aromatic partners (**3 l**,**q**,**s**,**t**) offers orthogonal reactivity to the well‐established Buchwald–Hartwig coupling. The reaction was also amenable to the installation of the piperidine ring onto fluorene (**3 v**), fluorenone (**3 w**), dibenzothiophene (**3 y**), azulene (**3 x**), 8‐MeO‐quinoline (**3 z**), PMP‐pyrrole (**3 aa**) and *N*‐Me‐indole (**3 ab**).

**Scheme 3:**
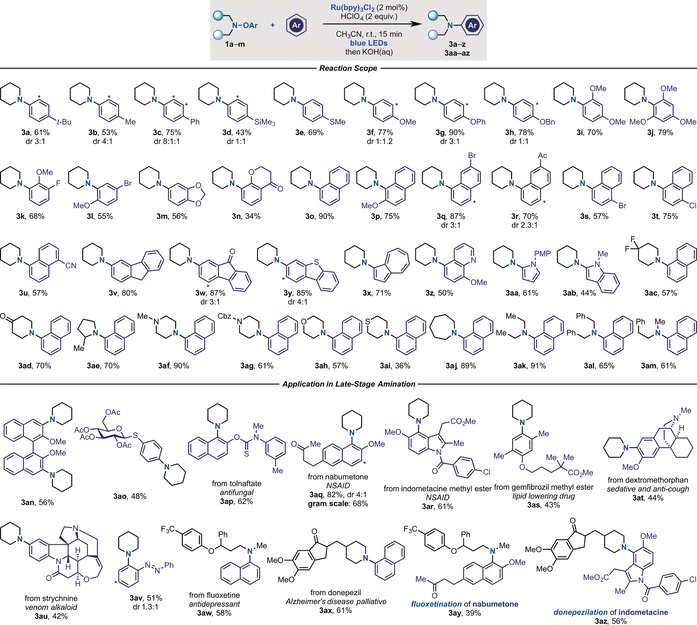
Reaction scope. Cbz=carboxybenzyl.

We were particularly keen to demonstrate the power of this strategy by engaging some of the most frequent nitrogen heterocycles found in small‐molecule drugs.1a, 1c Pleasingly, the method enabled the introduction of substituted piperidines (**3 ac**,**ad**), 2‐methylpyrrolidine (**3 ae**), *N*‐Me‐ and *N*‐Cbz‐piperazine (**3 af**, **ag**), morpholine (**3 ah**), thiomorpholine (**3 ai**), and azepine (**3 aj**). Non‐cyclic amines were also amenable, as demonstrated by the formation of products containing diethyl‐ (**3 ak**), dibenzyl‐ (**3 al**) and methyl,ethylbenzyl‐ (**3 am**) amine residues in high yields.

We then decided to benchmark this strategy in the late‐stage amination of complex molecules.^29^ To highlight this possibility, we selected substrates with broad utility as templates for chiral catalyst design, commercially available blockbuster drugs, natural products, and molecular switches. As shown in Scheme 3, we performed selective C‐4 amination of a binol derivative (**3 an**), which opens access to a novel class of chiral catalysts. We then showcased the power of the approach with the selective “piperidination” of an acid‐labile thioglucoside (**3 ao**), as well as the antifungal compound tolnaftate (**3 ap**), the NSAIDs nabumetone (**3 aq**; also produced on a gram scale) and indometacin (**3 ar**), the lipid‐lowering drug gemfibrozil (**3 as**), and the cough suppressant and sedative dextromethorphan (**3 at**). Since the drugs were all used without any pre‐functionalization at the aromatic unit and in equimolar amount and the reaction displayed excellent selectivity, we believe that this approach has the potential to become a useful tool in the rapid assembly of drug libraries. Regarding natural products, we successfully introduced a piperidine ring onto the highly complex venom alkaloid strychnine (**3 au**), which contains many redox‐active functionalities. Furthermore, we were able to perform amination of azobenzene (**3 av**), which is used as a molecular switch in supramolecular chemistry and optopharmacology.^30^ We also evaluated the late‐stage arylation of bioactive secondary amines. In this case, we successfully modified the anti‐depressant fluoxetine (**3 aw**), and the Alzheimer's disease palliative treatment donepezil, which underwent arylation with naphthalene in good yields (**3 ax**). Finally we used this method to “click” two biologically active molecules and form “two‐drugs‐in‐one” hybrids as shown by the “fluoxetination” of nabumetone (**3 ay**) and the “donepezilation” of indometacin (**3 az**).

In conclusion, we have developed a powerful and selective strategy for the amination of aromatics via aminium radicals. This approach provides fast access to aryl amines from unfunctionalized aromatic compounds in just 15 minutes. The generality of the process was illustrated by the late‐stage modification of chiral catalyst templates, blockbuster drugs, natural products, and photoswitches.

## Conflict of interest

The authors declare no conflict of interest.

## Supporting information

As a service to our authors and readers, this journal provides supporting information supplied by the authors. Such materials are peer reviewed and may be re‐organized for online delivery, but are not copy‐edited or typeset. Technical support issues arising from supporting information (other than missing files) should be addressed to the authors.

Supplementary
