# Supplementary material for: Synthesis of Arylamines via Aminium Radicals
Source: Angew Chem Int Ed Engl. 2017 Oct 24;56(47):14948–52. doi: 10.1002/anie.201708693 (PMC5698739; doi:10.1002/anie.201708693)
Supplement: Supplementary file 1 — Supplementary [file ANIE-56-14948-s001.pdf]

Angewandte Chemie
Eine Zeitschrift der Gesellschaft Deutscher Chemiker

Supporting Information

Synthesis of Arylamines via Aminium Radicals

*Thomas D. Svejstrup, Alessandro Ruffoni, Fabio Juliá, Valentin M. Aubert, and Daniele Leonori**

anie_201708693_sm_miscellaneous_information.pdf

Table of Contents

1 General Experimental Details 3

2 Starting Materials Synthesis 4

3 Arylation Reactions 15

3.1 Reaction Optimization 15
3.2 Alternative Aminium Radical Precursors 18
3.3 Substrate Scope 19

4 Mechanistic Considerations 46

4.1 Mechanism Based on the Closed Photoredox Cycle 47
4.2 Mechanism Based on the Ru(II)-Catalysed Electron Relay 49
4.2.1 Detection of $[Ru(bpy)_3]^{3+}$ 50
4.2.2 Further Studies to Support the Electron Relay Mode 51
4.3 Mechanism Based on the Radical Chain Propagation 54
4.4 Stability of $Ru(bpy)_3Cl_2$ in the presence of $HClO_4$ 57
4.5 Arylation of Aminium Radicals 58
4.5.1 Electrophilicity of Aminium Radicals 58
4.5.2 Reaction Selectivity – Fukui's Indices 58
4.6 Protonation Studies 60
4.7 Electrochemical Studies 62
4.7.1 General Experimental Details 62
4.7.2 Electrochemical Potentials 63
4.8 Emission Quenching Experiments 64

5 Computational Data 67

5.1 Computational Studies 67
5.1.1 Computational methods 67
5.2 Computational Data 68
5.2.1 Optimized structures for Fukui indices calculations. 68
5.2.2 Electrophilicity of Aminyl and Aminium Radicals 74
5.2.3 Optimized Energies (in Hartrees) and Geometries 75

6 NMR Spectra 90

7 References 165

1 General Experimental Details

All required fine chemicals were used directly without purification unless stated otherwise. All air and moisture sensitive reactions were carried out under nitrogen atmosphere using standard Schlenk manifold technique. THF was distilled from sodium/benzophenone, CH_2Cl_2 and was distilled from CaH_2, CH_3CN was distilled from activated 4Å molecular sieves, Et_3N was distilled over KOH. ^1H and ^{13}C Nuclear Magnetic Resonance (NMR) spectra were acquired at various field strengths as indicated and were referenced to $CHCl_3$ (7.27 and 77.0 ppm for ^1H and ^{13}C respectively). ^1H NMR coupling constants are reported in Hertz and refer to apparent multiplicities and not true coupling constants. Data are reported as follows: chemical shift, integration, multiplicity (s = singlet, br s = broad singlet, d = doublet, t = triplet, q = quartet, qi = quintet, sx = sextet, sp = septet, m = multiplet, dd = doublet of doublets, etc.), proton assignment (determined by 2D NMR experiments: COSY, HSQC and HMBC) where possible. High-resolution mass spectra were obtained using a JEOL JMS-700 spectrometer or a Fissions VG Trio 2000 quadrupole mass spectrometer. Spectra were obtained using electron impact ionization (EI) and chemical ionization (CI) techniques, or positive electrospray (ES). Infra-red spectra were recorded using a JASCO FT/IR 410 spectrometer or using an ATI Mattson Genesis Seris FTIR spectrometer as evaporated films or liquid films. Analytical TLC: aluminum backed plates pre-coated (0.25 mm) with Merck Silica Gel 60 F254. Compounds were visualized by exposure to UV-light or by dipping the plates in permanganate ($KMnO_4$) stain followed by heating. Flash column chromatography was performed using Merck Silica Gel 60 (40–63 μm). All mixed solvent eluents are reported as v/v solutions. UV/Vis spectra were obtained using an Agilent 6453 spectrometer and 1 mm High Precision Cell made of quartz from Hellma Analytics.

The LEDs were bought from LEDLightZone.

All the reactions were conducted in CEM 10 mL glass microwave tubes.

2 Starting Materials Synthesis

Commercially Available N-hydroxylamines

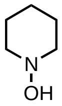

S1
CAS 4801-58-5

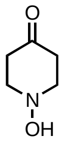

S2
CAS 113684-50-7

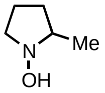

S3
CAS 151489-27-9

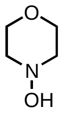

S4
CAS 5765-63-9

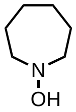

S5
CAS 6763-87-7

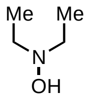

S6
CAS 3710-84-7

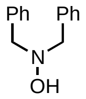

S7
CAS 621-07-8

General Procedure for the Synthesis of 2,4-$(NO_2)_2$-C_6H_3-Hydroxylamines 1a–l – GP1

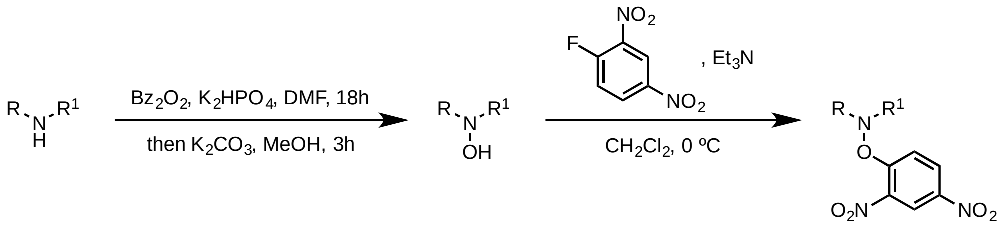

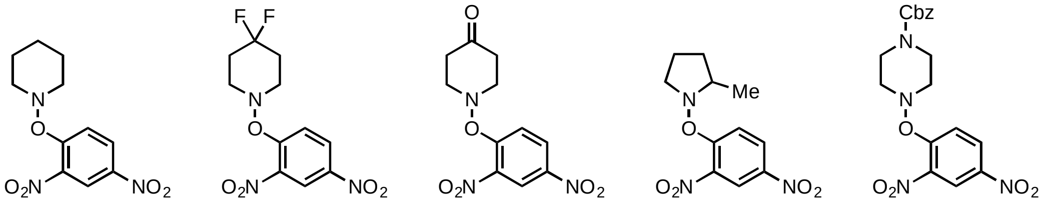

1a **1b** **1c** **1d** **1e**

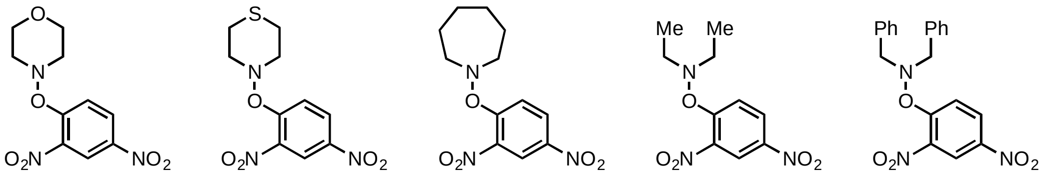

1f **1g** **1h** **1i** **1j**

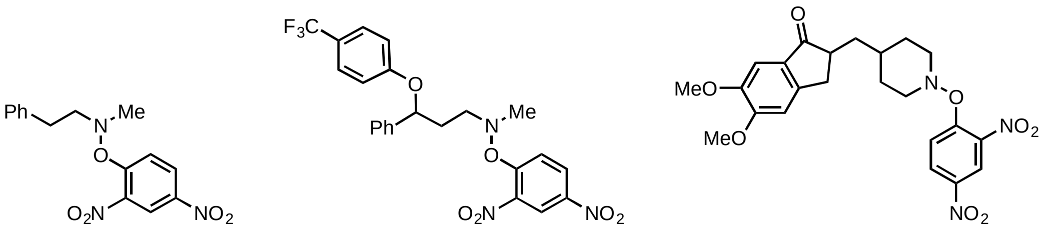

1k **1l** **1m**

GP1.1: Benzoyl peroxide (1.2 equiv.) was added to a solution of the amine (or the amine hydrochloride) and K_2HPO_4 (2.0 equiv.) in DMF (0.2 M). The mixture was stirred for 18 hours, diluted with H_2O and extracted with EtOAc (2 x). The combined organic layers were washed with Brine (x 3) and aqueous NH_4Cl, dried ($MgSO_4$), filtered and evaporated to give the crude *O*-benzoyl hydroxylamine that was used without further purification. The *O*-benzoyl hydroxylamine (1.0 equiv.) was dissolved in MeOH (0.5 M), treated with K_2CO_3 (2.0 equiv.) and stirred for 3 hours. The MeOH was removed under vacuum and the mixture was diluted with H_2O and EtOAc and the layers were separated. The aqueous layer was extracted with EtOAc (x 3) and the combined organic layers were dried ($MgSO_4$), filtered and evaporated to give the crude hydroxylamine that was used without further purification.

GP1.2: The crude hydroxylamine (1.0 equiv.) was dissolved in CH_2Cl_2 (0.2 M), cooled to 0 °C and treated with 1-fluoro-2,4-dinitrobenzene (1.2 equiv.) and Et_3N (2.0

equiv.). The mixture was allowed to warm to room temperature overnight. The reaction was diluted with H_2O and CH_2Cl_2 and the layers were separated. The aqueous layer was extracted with EtOAc (x 3) and the combined organic layers were dried ($MgSO_4$), filtered and evaporated. Purification by column chromatography on silica gel eluting with petrol:EtOAc (9:1) gave the product.

1-(2,4-Dinitrophenoxy)piperidine (1a)

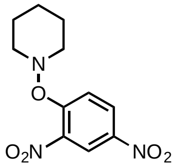

Following **GP1.2**, **S1** (2.0 g, 23.5 mmol) gave **1a** (4.4 g, 70%) as yellow crystals. mp: 74–75 °C; R_f 0.29 [petrol–EtOAc (9:1)]; FT-IR ν_{max} (film)/cm^{-1} 2948, 2856, 1603, 1525, 1472, 1342, 1277, 1141, 1064; ^{1}H NMR (400 MHz, $CDCl_3$) δ 8.78 (1H, d, *J* = 2.7 Hz), 8.38 (1H, dd, *J* = 9.4, 2.7 Hz), 7.88 (1H, d, *J* = 9.4 Hz), 3.45–3.26 (2H, m), 2.89 (2H, td, *J* = 10.8, 2.9 Hz), 1.96–1.85 (2H, m), 1.80–1.61 (3H, m), 1.42–1.23 (1H, m); ^{13}C NMR (101 MHz, $CDCl_3$) δ 158.0, 140.2, 136.6, 129.4, 122.2, 117.0, 57.2 (x 2), 25.3 (x 2), 23.1; HRMS (ASAP POS): Found MH$^+$ 268.0919 $C_{11}H_{14}N_3O_5$ requires 268.0928.

1-(2,4-Dinitrophenoxy)-4,4-difluoropiperidine (1b)

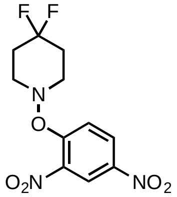

Following **GP1**, 4,4-difluoropiperidine hydrochloride (500 mg, 3.18 mmol) gave **1b** (395 mg, 41%) as yellow crystals. mp: 88–89 °C; R_f 0.23 [petrol–EtOAc (9:1)]; FT-IR ν_{max} (film)/cm^{-1} 3115, 2950, 1603, 1529, 1342, 1248, 1123; ^{1}H NMR (500 MHz, $CDCl_3$) δ 8.82 (1H, d, *J* = 2.6 Hz), 8.42 (1H, dd, *J* = 9.4, 2.6 Hz), 7.90 (1H, d, *J* = 9.3 Hz), 3.51–3.16 (4H, m), 2.59–2.24 (2H, m), 2.23–1.95 (2H, m); ^{13}C NMR (126 MHz, $CDCl_3$) δ 157.3, 140.8, 136.7, 129.7, 122.3, 120.5 (t, *J* = 242.5 Hz), 116.9, 52.4 (t, *J* = 4.6 Hz), 30.8 (t, *J* = 24.3 Hz); ^{19}F NMR (376 MHz, $CDCl_3$) δ –94.2 (d, *J* = 235.5 Hz), –102.2 (d, *J* = 238.1 Hz); HRMS (ASAP POS): Found MH$^+$ 304.0732 $C_{11}H_{12}N_3O_5F_2$ requires 304.0740.

1-(2,4-Dinitrophenoxy)piperidin-4-one (1c)

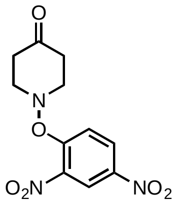

Following **GP1.2**, **S2** (250 mg, 2.18 mmol) gave **1c** (360 mg, 59%) as orange crystals. mp: 101–102 °C; R_f 0.29 [petrol–EtOAc (9:1)]; FT-IR ν_{max} (film)/cm^{-1} 3113, 2921, 1717, 1601, 1522, 1471, 1340, 1260, 1141, 1064; ^{1}H NMR (500 MHz, $CDCl_3$) δ 8.85 (1H, d, *J* = 2.7 Hz), 8.45 (1H, dd, *J* = 9.3, 2.7 Hz), 7.97 (1H, d, *J* = 9.3 Hz), 3.74–3.61 (2H, m), 3.58–3.38 (2H, m), 3.03–2.84 (2H, m), 2.59–2.34 (2H, m); ^{13}C NMR (126 MHz, $CDCl_3$) δ 205.5, 157.3, 140.9, 136.7, 129.8, 122.4, 116.9, 55.2 (x 2), 38.1 (x 2); HRMS (ASAP POS): Found MH$^+$ 282.0733 $C_{11}H_{12}N_3O_6$ requires 282.0726.

1-(2,4-Dinitrophenoxy)-2-methylpyrrolidine (1d)

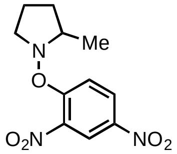

Following **GP1.2**, **S3** (1.0 g, 11.76 mmol) gave **1d** (2.0 g, 64%) as yellow crystals. mp: 72–73 °C; R_f 0.29 [petrol–EtOAc (9:1)]; FT-IR ν_{max} (film)/cm^{-1} 2936, 2853, 1609, 1542, 1455, 1334, 1277, 1054; ^{1}H NMR (400 MHz, $CDCl_3$) δ 8.79 (1H, d, *J* = 2.7 Hz), 8.38 (1H, dd, *J* = 9.4, 2.7 Hz), 7.98 (1H, d, *J* = 9.4 Hz), 3.46–3.35 (2H, m), 3.23–3.08 (1H, m), 2.26–1.78 (3H, m), 1.50 (1H, s), 1.33–1.04 (3H, m); ^{13}C NMR (101 MHz, $CDCl_3$) δ 159.2, 140.1, 136.3, 129.3, 122.1, 117.5, 64.0, 55.3, 29.8, 28.9, 20.3, 18.9; HRMS (ESI): Found MH$^+$ 268.0935 $C_{11}H_{14}N_3O_5Na$ requires 268.0933.

Benzyl 4-(2,4-Dinitrophenoxy)piperazine-1-carboxylate (1e)

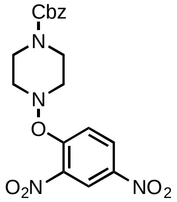

Following **GP1**, benzyl piperazine-1-carboxylate (1.0 g, 4.54 mmol) gave **1e** (1.46 g, 81%) as red crystals. mp: 91–92 °C; R_f 0.29 [petrol–EtOAc (9:1)]; FT-IR ν_{max} (film)/cm^{-1} 2961, 2864, 1700, 1604, 1529, 1470, 1428, 1342, 1264, 1225, 1126, 1027;

^{1}H NMR (400 MHz, $CDCl_3$) δ 8.81 (1H, d, J = 2.7 Hz), 8.41 (1H, dd, J = 9.3, 2.7 Hz), 7.87 (1H, d, J = 9.3 Hz), 5.16 (2H, s), 4.28–3.97 (2H, m), 3.52–3.28 (4H, m), 3.08 (2H, br t, J = 9.1 Hz); ^{13}C NMR (101 MHz, $CDCl_3$) δ 157.2, 155.1, 140.8, 136.8, 136.4, 129.5, 128.8 (x 2), 128.4, 128.2 (x 2), 122.3, 116.8, 67.8, 55.4 (x 2), 42.0 (x 2); HRMS (ASAP POS): Found MH$^+$ 403.1239 $C_{18}H_{19}N_4O_7$ requires 403.1248.

4-(2,4-Dinitrophenoxy)morpholine (1f)

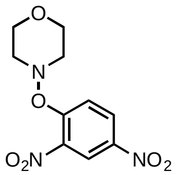

Following **GP1.2**, **S4** (1.0 g, 11.49 mmol) gave **1f** (2.1 g, 67%) as orange crystals. mp: 101–102 °C; R_f 0.29 [petrol–EtOAc (9:1)]; FT-IR ν_{max} (film)/cm^{-1} 2964, 2862, 1670, 1603, 1525, 1472, 1343, 1261, 1050; ^{1}H NMR (400 MHz, $CDCl_3$) δ 8.80 (1H, d, J = 2.7 Hz), 8.40 (1H, dd, J = 9.4, 2.7 Hz), 7.89 (1H, d, J = 9.4 Hz), 4.06 (2H, br d, J = 12.2 Hz), 3.75 (2H, ddd, J = 12.2, 10.3, 2.3 Hz), 3.48–3.29 (2H, m), 3.15 (2H, td, J = 10.6, 3.3 Hz); ^{13}C NMR (101 MHz, $CDCl_3$) δ 157.2, 140.6, 137.6, 129.3, 122.1, 116.7, 65.7 (x 2), 56.5 (x 2); HRMS (ASAP POS): Found MH$^+$ 270.0711 $C_{10}H_{12}N_3O_6$ requires 270.0721.

4-(2,4-Dinitrophenoxy)thiomorpholine (1g)

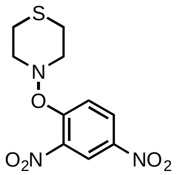

Following **GP1**, thiomorpholine (1.0 g, 9.71 mmol) gave **1g** (1.5 g, 55%) as yellow crystals. mp: 99–101 °C; R_f 0.29 [petrol–EtOAc (9:1)]; FT-IR ν_{max} (film)/cm^{-1} 3090, 2930, 2852, 1602, 1525, 1471, 1341, 1265, 1142, 1063; ^{1}H NMR (400 MHz, $CDCl_3$) δ 8.80 (1H, d, J = 2.7 Hz), 8.39 (1H, dd, J = 9.4, 2.7 Hz), 7.82 (1H, d, J = 9.4 Hz), 3.65–3.54 (2H, m), 3.40–3.23 (2H, m), 3.03–2.77 (4H, m); ^{13}C NMR ($CDCl_3$) δ 157.2, 140.7, 136.7, 129.5, 122.3, 116.81, 57.2 (x 2), 26.5 (x 2); HRMS (ASAP POS): Found MH$^+$ 286.0482 $C_{10}H_{12}N_3O_5S$ requires 286.0492.

1-(2,4-Dinitrophenoxy)azepane (1h)

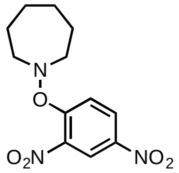

Following **GP1.2**, **S5** (500 mg, 5.1 mmol) gave **1h** (1.0 g, 71%) as a yellow oil. R_f 0.29 [petrol–EtOAc (9:1)]; FT-IR ν_{max} (film)/cm^{-1} 2931, 2857, 1601, 1522, 1470, 1339, 1264, 1064; ^{1}H NMR (400 MHz, $CDCl_3$) δ 8.78 (1H, d, *J* = 2.7 Hz), 8.39 (1H, dd, *J* = 9.4, 2.7 Hz), 7.88 (1H, d, *J* = 9.4 Hz), 3.29 (4H, br s), 1.80 (4H, br s), 1.73–1.65 (4H, m); ^{13}C NMR (101 MHz, $CDCl_3$) δ 158.1, 140.2, 136.5, 129.5, 122.1, 117.1, 59.4 (x 2), 26.2 (x 2), 24.4 (x 2); HRMS (ASAP POS): Found MH$^+$ 282.1077 $C_{12}H_{16}N_3O_6$ requires 282.1084.

O-(2,4-Dinitrophenyl)-*N*,*N*-diethylhydroxylamine (1i)

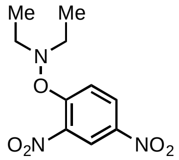

Following **GP1.2**, **S7** (500 mg, 5.6 mmol) gave **1i** (791 mg, 55%) as yellow crystals. mp: 73–74 °C; R_f 0.29 [petrol–EtOAc (9:1)]; FT-IR ν_{max} (film)/cm^{-1} 3116, 2981, 2877, 1603, 1526, 1472, 1341, 1316, 1263; ^{1}H NMR (400 MHz, $CDCl_3$) δ 8.76 (1H, d, *J* = 2.7 Hz), 8.37 (1H, dd, *J* = 9.4, 2.8 Hz), 7.96 (1H, d, *J* = 9.4 Hz), 3.14–3.02 (4H, m), 1.13 (6H, t, *J* = 7.1 Hz); ^{13}C NMR (101 MHz, $CDCl_3$) δ 159.7, 140.2, 136.0, 129.2, 122.0, 117.7, 53.7 (x 2), 12.1 (x 2); HRMS (ASAP POS): Found MH$^+$ 256.0923 $C_{10}H_{14}N_3O_5$ requires 256.0928.

***N*,*N*-Dibenzyl-O-(2,4-dinitrophenyl)hydroxylamine (1j)**

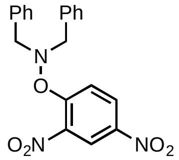

Following **GP1.2**, **S8** (500 mg, 2.3 mmol) gave **1j** (357 mg, 41%) as yellow crystals. mp: 103-104 °C; R_f 0.29 [petrol–EtOAc (9:1)]; FT-IR ν_{max} (film)/cm^{-1} 3031, 1602, 1342, 1276, 1236; ^{1}H NMR (400 MHz, $CDCl_3$) δ 8.55 (1H, d, *J* = 2.7 Hz), 7.99 (1H, dd, *J* = 9.4, 2.8 Hz), 7.45 (1H, d, *J* = 9.4 Hz), 7.37–7.31 (4H, m), 7.28–7.16 (6H, m),

4.22 (4H, s); ^{13}C NMR (101 MHz, $CDCl_3$, 1C missing) δ 158.6, 140.0, 135.0, 130.0 (x 4), 128.7 (x 4), 128.4, 128.35 (x 2), 121.5, 117.6, 63.7 (x 2); HRMS (ASAP POS): Found MH^+ 380.1239 $C_{20}H_{18}N_3O_5$ requires 380.1241.

O-(2,4-Dinitrophenyl)-*N*-methyl-*N*-phenethylhydroxylamine (1k)

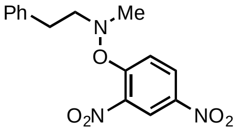

Following **GP1**, *N*-methyl-2-phenylethan-1-amine (500 mg, 3.7 mmol) gave **1k** (389 mg, 33%) as orange crystals. mp: 67-68 °C; R_f 0.51 [petrol–EtOAc (9:1)]; FT-IR ν_{max} (film)/cm^{-1} 3081, 2927, 1602, 1495,1340, 1315, 1266, 1064; ^{1}H NMR (500 MHz, $CDCl_3$) δ 8.78 (1H, br s), 8.31 (1H, dd, *J* = 9.4, 2.8 Hz), 7.69 (1H, d, *J* = 9.3 Hz), 7.26 (2H, td, *J* = 7.2, 1.4 Hz), 7.23–7.18 (1H, m), 7.13 (2H, d, *J* = 6.9 Hz), 3.53–3.11 (2H, m), 2.93–2.82 (5H, m); ^{13}C NMR (126 MHz, $CDCl_3$) δ 158.3, 140.4, 138.8, 136.3, 129.3, 128.8 (x 2), 128.6 (x 2), 126.6, 122.1, 117.2, 62.5, 46.5, 33.5; HRMS (ASAP POS): Found MH^+ 318.1079 $C_{15}H_{16}N_3O_5$ requires 318.1084.

O-(2,4-Dinitrophenyl)-*N*-methyl-*N*-(3-phenyl-3-(4-(trifluoromethyl)phenoxy) propyl)hydroxylamine (1l)

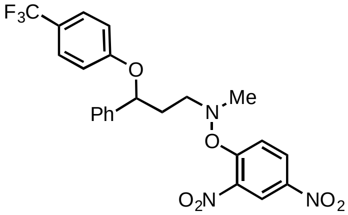

Following **GP1**, fluoxetine hydrochloride (500 mg, 1.45 mmol) gave **1l** (380 mg, 53%) as an oil. R_f 0.28 [petrol–EtOAc (9:1)]; FT-IR ν_{max} (film)/cm^{-1} 2929, 1604, 1527, 1342, 1327, 1248, 1111, 1067; ^{1}H NMR (500 MHz, $CDCl_3$) δ 8.79 (1H, br s), 8.35 (1H, br s), 7.81 (1H, br d, *J* = 9.3 Hz), 7.44 (2H, d, *J* = 8.4 Hz), 7.33 (2H, t, *J* = 7.3 Hz), 7.30–7.21 (3H, m), 6.87 (2H, d, *J* = 8.4 Hz), 5.26 (1H, dd, *J* = 8.9, 4.2 Hz), 3.25 (2H, br s), 2.90 (3H, s), 2.27 (1H, dq, *J* = 13.9, 7.4 Hz), 2.12 (1H, dq, *J* = 13.9 6.8 Hz); ^{13}C NMR (126 MHz, $CDCl_3$) δ 160.2, 158.0, 140.4, 140.2, 136.3, 129.3, 129.0 (x 2), 128.2, 126.8 (q, *J* = 3.6, x 2), 125.6 (x 2), 123.5–122.8 (m), 122.1, 116.7, 116.1, 115.7 (x 2), 78.1, 57.6, 46.5, 36.0; ^{19}F NMR (376 MHz, $CDCl_3$) δ –61.7 (x 3); HRMS (ASAP POS): Found MH^+ 492.1368 $C_{23}H_{21}N_3O_6F_3$ requires 492.1377.

2-((1-(2,4-Dinitrophenoxy)piperidin-4-yl)methyl)-5,6-dimethoxy-2,3-dihydro-1*H*-inden-1-one (1m)

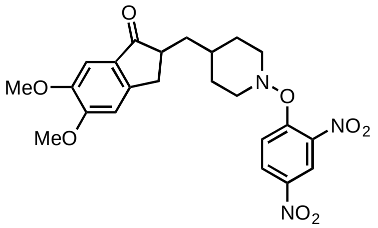

Following **GP1**, desbenzyl donezepil hydrochloride (250 mg, 0.77 mmol) gave **1m** (170 mg, 46%) as an oil. R_f 0.11 [petrol–EtOAc (8:2)]; FT-IR ν_{max} (film)/cm^{-1} 2928 2845, 1694, 1603, 1525, 1500, 1469, 1341, 1313, 1264, 1125, 1037; ^{1}H NMR (400 MHz, $CDCl_3$) δ 8.75 (1H, d, *J* = 2.8 Hz), 8.37 (1H, dd, *J* = 9.3, 2.7 Hz), 7.88 (1H, d, *J* = 9.4 Hz), 7.15 (1H, s), 6.86 (1H, s), 3.95 (3H, s), 3.89 (3H, s), 3.42 (2H, br t, *J* = 8.1, Hz), 3.26 (1H, dd, *J* = 16.9, 7.6 Hz), 3.16–2.82 (2H, m), 2.75–2.62 (2H, m), 2.07–1.80 (4H, m), 1.75–1.31 (4H, m); ^{13}C NMR (101 MHz, $CDCl_3$) δ 207.2, 157.9, 155.7, 149.6, 148.7, 140.3, 136.5, 129.4, 129.2, 122.1, 117.0, 107.5, 104.5, 56.7, 56.6, 56.3, 56.2, 45.5, 38.0, 33.2, 33.2, 32.5, 31.4; HRMS (ASAP POS): Found MH^+ 472.1714 $C_{23}H_{26}N_3O_8$ requires 472.1714.

Piperidin-1-yl benzoate (S8)

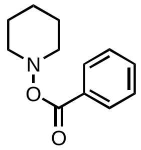

Following **GP1.1**, piperidine (9.9 mL, 100 mmol) gave **S8** (18 g, 88%) as a solid. ^{1}H NMR (400 MHz, $CDCl_3$) δ 8.01–7.99 (2H, m), 7.56–7.52 (1H, m), 7.44–7.40 (2H, m), 3.51–3.49 (2H, m), 2.78 – 2.76 (2H, m), 1.86–1.79 (4H, m), 1.69–1.66 (1H, m), 1.31–1.25 (1H, m); ^{13}C NMR (100 MHz, $CDCl_3$) 164.9, 133.0, 129.8, 129.5 (x 2), 128.5 (x 2), 57.6 (x 2), 25.1 (x 2), 23.5. Data in accordance with the literature.[1]

4-(Piperidin-1-yloxy)benzonitrile (S9)

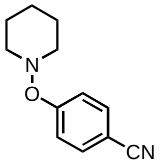

Following **GP1.2** but using 4-F-benzonitrile, **S1** (303 mg, 3 mmol) gave **S9** (116 mg, 19%) as a white solid. mp: 103–104 °C; R_f 0.34 [petrol–EtOAc (95:5)]; FT-IR ν_{max} (film)/cm^{-1} 2921, 2870, 2231, 1602, 1506, 1237, 1160; ^{1}H NMR (500 MHz, $CDCl_3$) δ 7.54 (2H, d, *J* = 8.3 Hz), 7.14 (2H, d, *J* = 8.3 Hz), 3.31 (2H, br d, *J* = 9.8 Hz), 2.66 (2H, br t, *J* = 11.1 Hz), 1.83 (2H, br d, *J* = 14.6 Hz), 1.74–1.62 (3H, m), 1.24 (1H, br s); ^{13}C NMR (126 MHz, $CDCl_3$) δ 162.8, 134.0 (x 2), 119.6, 114.6 (x 2), 103.9, 56.9 (x 2), 25.5 (x 2), 23.3; HRMS (APCI): Found MH$^+$ 203.1170 $C_{12}H_{15}N_2O$ requires 203.1179.

General Procedure for the Preparation of S10,11 – GP2

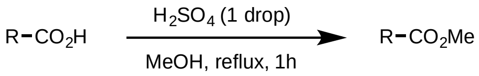

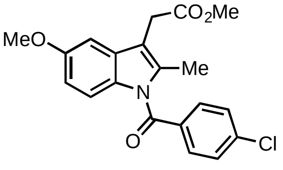

S10

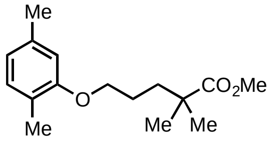

S11

The carboxylic acid (1.0 equiv.) was dissolved in MeOH (0.05M) and a drop of H_2SO_{4conc} was added. The mixture was refluxed for 1 hour, cooled to room temperature and evaporated. Et_2O (10 mL) was added and the organic layer was washed with $NaHCO_{3sat}$ (10 mL). The organic layer was dried ($MgSO_4$), filtered and evapoarted to give the methyl ester. No further purification was required.

Methyl 2-(1-(4-Chlorobenzoyl)-5-methoxy-2-methyl-1*H*-indol-3-yl)acetate (S10)

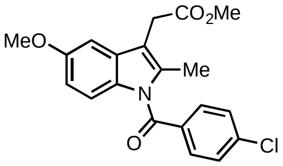

Following **GP2**, indomethacin (200 mg, 0.55 mmol) gave **S10** (127 mg, 62%) as a solid. ^{1}H NMR (400 MHz, $CDCl_3$) δ 7.66 (2H, d, *J* = 8.5 Hz), 7.47 (2H, d, *J* = 8.5 Hz), 6.96 (1H, d, *J* = 2.5 Hz), 6.86 (1H, d, *J* = 9.0 Hz), 6.67 (1H, dd, *J* = 9.0, 2.5 Hz), 3.83 (3H, s), 3.70 (3H, s), 3.67 (2H, s), 2.38 (3H, s); ^{13}C NMR (101 MHz, $CDCl_3$) δ 171.3, 168.2, 156.0, 139.2, 135.9, 133.8, 131.1 (x 2), 130.7, 130.6, 129.1 (x 2), 114.9, 112.5, 111.5, 101.2, 55.7, 52.1, 30.1, 13.3. Data in accordance with the literature.[2]

Methyl 5-(2,5-Dimethylphenoxy)-2,2-dimethylpentanoate (S11)

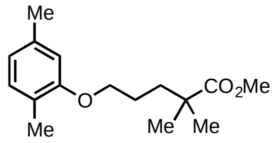

Following **GP2**, gemfibrozil (200 mg, 0.80 mmol) gave **S11** (182 mg, 86%) as an oil. ^{1}H NMR (500 MHz, $CDCl_3$) δ 7.00 (1H, d, *J* = 7.4 Hz), 6.66 (1H, d, *J* = 7.4 Hz), 6.61 (1H, s), 3.97–3.88 (2H, m), 3.67 (2H, app s), 2.31 (3H, s), 2.18 (3H, s), 1.85–1.69 (4H, m), 1.24 (6H, s); ^{13}C NMR (125 MHz, $CDCl_3$) δ 178.4, 156.9, 136.5, 130.3,

123.6, 120.7, 111.9, 67.9, 51.8, 42.1, 37.1, 36.9, 25.2, 21.5, 15.8. Data in accordance with the literature.[3]

3 Arylation Reactions

3.1 Reaction Optimization

General Procedure for the Reaction Optimization – GP3

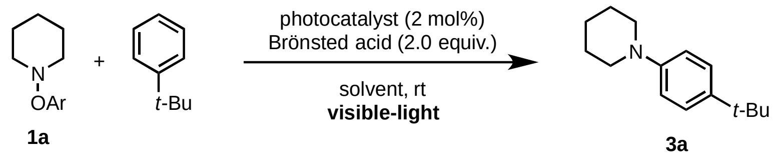

To a dry tube was added **1a** (27 mg, 0.1 mmol, 1.0 equiv.) and the photocatalyst (2 mol%). A stirrer bar was added and the tube was capped with a Supelco aluminium crimp seal with septum (PTFE/butyl). The tube was evacuated and refilled with N_2 (x 3) and *t*-Bu-benzene (34 µL, 0.2 mmol, 2.0 equiv.), the solvent (dry and degassed by bubbling through with N_2 for 20 min) and the Brönsted acid. The mixture was stirred in front of blue LEDs for 15 min. KOH (1 M, 1.0 mL), water (10 mL) and EtOAc (10 mL) were added and the mixture was stirred for 1 min. 1,3,5-trimethoxybenzene (16.8 mg, 0.1 mmol, 1.0 equiv.) was added and the layers were separated. The aqueous layer was extracted with EOAc (x 3), the combined organic layers were dried ($MgSO_4$), filtered and evaporated. $CDCl_3$ (0.4 mL) was added and the mixture was analysed by ^1H NMR spectroscopy to determine the NMR yield.

The optimum reaction conditions identified by this optimisation study were:

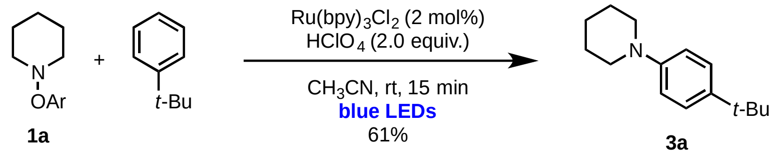

Table S1 reports all the experiments performed.

Table S1.

**Entry**	**Photocatalyst**	**Brönsted Acid (equiv.)**	**Time**	**Solvent**	**Yield (%)**
**1**	$Ru(bpy)_3Cl_2$	–	24 h	CH_3CN	–
**2**	$Ru(bpy)_3Cl_2$	AcOH (2.0)	24 h	CH_3CN	–
**3**	$Ru(bpy)_3Cl_2$	TFA (2.0)	24 h	CH_3CN	–
**4**	$Ru(bpy)_3Cl_2$	TFA (10.0)	24 h	CH_3CN	–
**5**	$Ru(bpy)_3Cl_2$	*p*-TsOH (2.0)	24 h	CH_3CN	19
**6**	$Ru(bpy)_3Cl_2$	$HClO_4$ (2.0)	15 min	CH_3CN	61
**7**[a]	$Ru(bpy)_3Cl_2$	$HClO_4$ (2.0)	15 min	CH_3CN	51
**8**	$Ru(bpy)_3Cl_2$	$HClO_4$ (1.5)	15 min	CH_3CN	39
**9**	$Ru(bpy)_3Cl_2$	$HClO_4$ (1.0)	15 min	CH_3CN	18
**10**	$Ru(bpy)_3Cl_2$	$HClO_4$ (0.2)	15 min	CH_3CN	9
**11**	$Ru(bpy)_3Cl_2$	$HClO_4$ (2.0)	15 min	MeOH	–
**12**	$Ru(bpy)_3Cl_2$	$HClO_4$ (2.0)	15 min	CH_2Cl_2	9
**13**	$Ru(bpy)_3Cl_2$	$HClO_4$ (2.0)	15 min	acetone	–
**14**	$Ir(ppy)_3$	$HClO_4$ (2.0)	24 h	CH_3CN	–
**15**	Fukuzumi's acridinium	$HClO_4$ (2.0)	24 h	CH_3CN	–
**16**	Rhodamine G	$HClO_4$ (0.5)	24 h	CH_3CN	–

[a] The reaction was run in the dark

Running the reaction under the conditions of entry 6 gave the following crude ^{1}H NMR spectrum (400 MHz, $CDCl_3$):

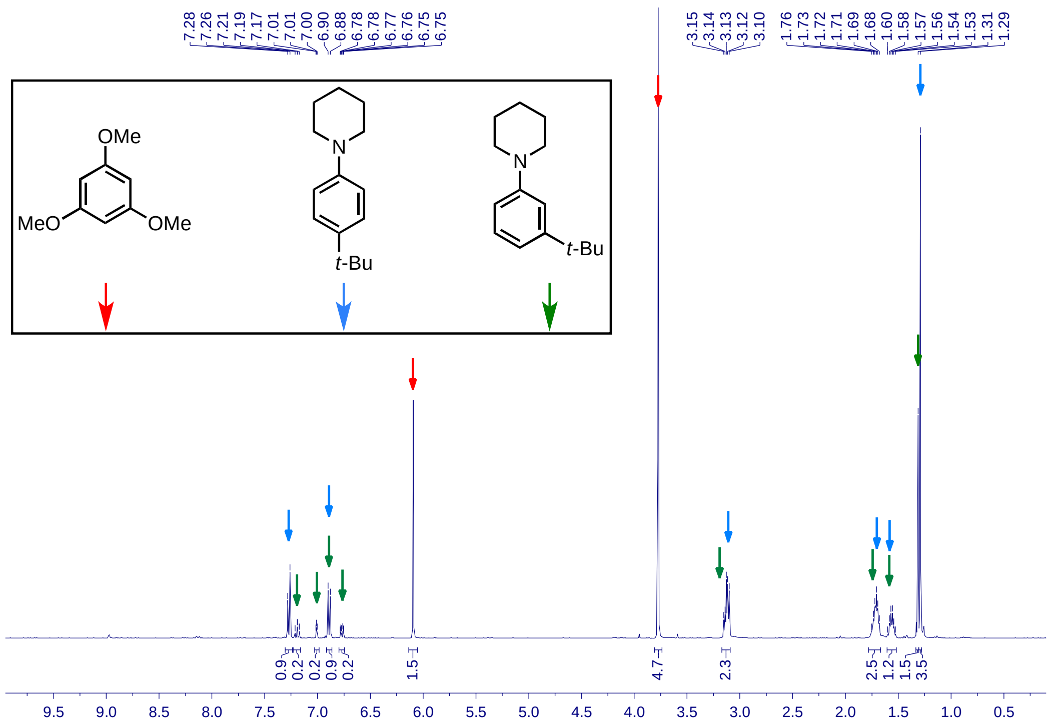

3.2 Alternative Aminium Radical Precursors

We have evaluated the possibility of using alternative aminium radical precursors and subjected **S8** and **S9** to our reaction conditions. As reported in Table S2, none of them provided **3a** and as a result cannot be considered as viable options in our reaction manifold.

Table S2.

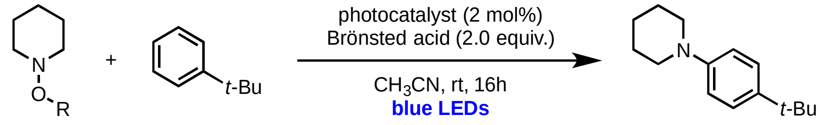

S8,9 **3a**

Entry	Photocatalyst	Brönsted acid	Yield (%)
	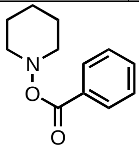 **S8**		
**1**	$Ru(bpy)_3Cl_2$	$HClO_4$	–
**2**	$Ru(bpy)_3Cl_2$	TFA	–
**3**	$Ir(ppy)_3$	$HClO_4$	traces
**4**	$Ir(dF\text{-}CF_3\text{-}ppy_2)(dtbpy)(PF_6)$	$HClO_4$	traces
**5**	$Ir(dF\text{-}ppy)_3$	$HClO_4$	traces
	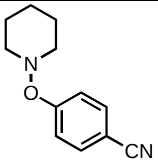 **S9**		
**8**	$Ru(bpy)_3Cl_2$	$HClO_4$	17
**9**	$Ru(bpy)_3Cl_2$	TFA	–

3.3 Substrate Scope

General Procedure for the Amination Reaction – GP4

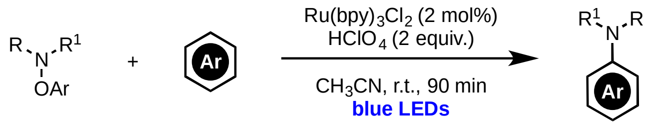

To a dry tube was added with the O-aryl hydroxylamine (1.0 equiv.) and Ru(bpy)$_3$Cl$_2$ (2 mol%) and the aromatic (2.0 equiv.) if solid. A stirrer bar was added and the tube was capped with a Supelco aluminium crimp seal with septum (PTFE/butyl). The tube was evacuated and refilled with N_2 (x 3) and the aromatic (2.0 equiv.) if liquid, CH_3CN (0.1 M). The blue LEDs were turned on, and $HClO_4$ (2.0 equiv., 70% in H_2O) was added and the mixture was stirred under irradiation for 15 min. KOH (1 M, 1.0 mL), water (10 mL) and EtOAc (10 mL) were added, the mixture was stirred for 5 min and the layers were separated. The aqueous layer was extracted with EOAc (x 3), the combined organic layers were dried ($MgSO_4$), filtered and evaporated. Purification by column chromatography on silica gel gave the product.

The structures of the unknown products have been determined by 2D analysis using COSY, HSQC, HMBC and NOESY spectroscopy.

1-(4-(*tert*-Butyl)phenyl)piperidine (3a) and 1-(3-(*tert*-Butyl)phenyl)piperidine (3a')

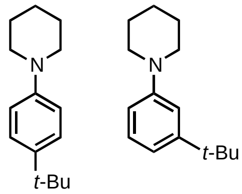

Following **GP4**, **1a** (27 mg, 0.1 mmol) gave **3a** (10 mg, 46%) as an oil and **3a'** (4 mg, 15%) as an oil. **3a**:**3a'** = 3:1.

Data for **3a**: ^{1}H NMR (500 MHz, $CDCl_3$) δ 7.30 (2H, d, *J* = 8.5 Hz), 6.92 (2H, d, *J* = 8.5 Hz), 3.14 (4H, t, *J* = 5.5 Hz), 1.75–1.71 (4H, m), 1.60–1.55 (2H, m), 1.31 (s, 9 H); ^{13}C NMR (126 MHz, $CDCl_3$) δ 149.9, 141.9, 125.7 (x 2), 116.2 (x 2), 50.9 (x 2), 33.9, 31.4 (x 3), 26.0 (x 2), 24.3. Data in accordance with the literature.[4]

Data for **3a'**: ^{1}H NMR (500 MHz, $CDCl_3$) 7.19 (1H, t, *J* = 8.0 Hz), 7.01 (1H, t, *J* = 2.1 Hz), 6.89 (1H, d, *J* = 8.8 Hz), 6.77 (1H, dd, *J* = 8.0, 2.1 Hz), 3.16–3.12 (4H, m), 1.76–1.65 (4H, m), 1.60–1.53 (2H, m), 1.31 (9H, s).

1-(*p*-Tolyl)piperidine (3b) and 1-(*m*-tolyl)piperidine (3b')

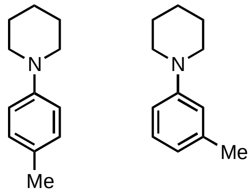

Following **GP4**, **1a** (27 mg, 0.1 mmol) gave **3b** (7 mg, 42%) as an oil and **3b'** (3 mg, 11%) as an oil. **3b**:**3b'** = 4:1.

Data for **3b**: ^{1}H NMR (400 MHz, $CDCl_3$) δ 7.06 (2H, d, *J* = 8.0 Hz), 6.86 (2H, d, *J* = 7.9 Hz), 3.08 (4H, t, *J* = 5.5 Hz), 2.26 (3H,s), 1.71 (4H, t, *J* = 5.8 Hz), 1.60–1.52 (2H, m); ^{13}C NMR (101 MHz, $CDCl_3$) δ 150.2, 129.5 (x 2), 128.6 (x 2), 116.9, 51.2 (x 2), 25.9 (x 2), 24.3, 20.3. Data in accordance with the literature.[5]

Data for **3b'**: ^{1}H NMR (400 MHz, $CDCl_3$) δ 7.12 (1H, t, *J* = 7.5 Hz), 6.75–6.73 (2H, m), 6.64 (1H, d, *J* = 7.5 Hz), 3.12 (4H, t, *J* = 5.5 Hz), 2.30 (3H, s), 1.71–1.66 (4H, m), 1.57–1.53 (2H, m); ^{13}C NMR (126 MHz, $CDCl_3$) δ 152.3, 138.5, 128.8, 120.1, 117.4, 113.6, 50.7 (x 2), 25.9 (x 2), 24.3, 21.7. Data in accordance with the literature.[5]

1-([1,1'-Biphenyl]-4-yl)piperidine (3c) and 1-([1,1'-Biphenyl]-3-yl)piperidine (3c) (3c') and 1-([1,1'-Biphenyl]-2-yl)piperidine (3c'')

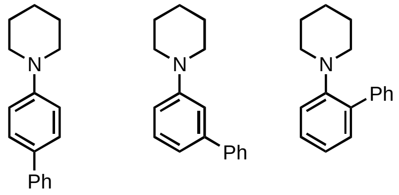

Following **GP4**, **1a** (27 mg, 0.1 mmol) gave **3c** (18 mg, 75%) as an oil as well as **3c'** and **3c''** which were not purified. **3c**:**3c'**:**3c''** = 8:1:1.

Data for **3c**: ^{1}H NMR (500 MHz, $CDCl_3$) δ 7.56 (2H, d, *J* = 7.9 Hz), 7.51 (2H, d, *J* = 8.7 Hz), 7.40 (2H, t, *J* = 7.7 Hz), 7.28 (1H, d, *J* = 7.5 Hz), 7.01 (2H, d, *J* = 8.4 Hz), 3.25–3.16 (4H, t, *J* = 5.5 Hz), 1.73 (4H, p, *J* = 5.7 Hz), 1.60 (4H, p, *J* = 6.0 Hz); ^{13}C NMR (126 MHz, $CDCl_3$) δ 151.6, 141.2, 131.8, 128.8 (x 2), 127.8 (x 2), 126.6 (x 2), 126.4, 116.6 (x 2), 50.6 (x 2), 25.9 (x 2), 24.5. Data in accordance with the literature.[6]

1-(4-(Trimethylsilyl)phenyl)piperidine (3d) and 1-(3-(Trimethylsilyl)phenyl) piperidine (3d')

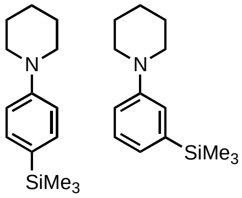

Following **GP4**, **1a** (27 mg, 0.1 mmol) gave **3d** and **3d'** (10 mg, 43%) as an inseparable mixture, as an oil. **3d**:**3d'** = 1:1. R_f 0.26 [petrol–EtOAc (98:2)]; FT-IR ν_{max} (film)/cm^{-1} 2934, 2853, 1568, 1237, 1113, 1027; ^{1}H NMR (500 MHz, $CDCl_3$, isomers) δ 7.42–7.36 (1H, m), 7.25 (0.5H, dd, *J* = 8.2, 7.1 Hz), 7.11 (0.5H, dd, *J* = 2.6, 1.0 Hz), 6.99 (0.5H, dt, *J* = 7.1, 1.1 Hz), 6.96–6.88 (1.5H, m), 3.22–3.15 (2H, m), 3.18–3.12 (2H, m), 1.78–1.65 (4H, m), 1.62–1.56 (2H, m), 0.25 (4.5H, s), 0.23 (4.5H, s); ^{13}C NMR (126 MHz, $CDCl_3$, isomers) δ 152.6, 151.9, 141.1, 134.4 (x 2), 128.8, 128.6, 124.5, 122.0, 117.3, 115.6 (x 2), 51.1 (x 2), 50.1 (x 2), 26.2 (x 2), 25.9 (x 2), 24.5, 24.5, –0.8 (x 3), –0.9 (x 3); HRMS (APCI): Found MH^+ 234.1679 $C_{14}H_{24}NSi$ requires 234.1678.

1-(4-(Methylthio)phenyl)piperidine (3e)

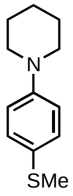

Following **GP4**, **1a** (27 mg, 0.1 mmol) gave **3e** (18 mg, 69%) as an oil. R_f 0.55 [petrol–EtOAc (98:2)]; FT-IR ν_{max} (film)/cm^{-1} 2961, 2929, 2851, 1597, 1496, 1259, 1230, 1026; ^{1}H NMR (500 MHz, $CDCl_3$) δ 7.24 (2H, d, *J* = 8.8 Hz), 6.87 (2H, d, *J* = 8.8 Hz), 4.57–2.99 (4H, m), 2.43 (3H, s), 1.74–1.67 (4H, m), 1.60–1.55 (2H, m); ^{13}C NMR (126 MHz, $CDCl_3$) δ 150.9, 130.3 (x 2), 126.7, 117.2 (x 2), 50.7 (x 2), 25.9 (x 2), 24.4, 18.4; HRMS (APCI): Found MH^+ 208.1155 $C_{12}H_{18}NS$ requires 208.1154.

1-(4-Methoxyphenyl)piperidine (3f) and 1-(2-Methoxyphenyl)piperidine (3f')

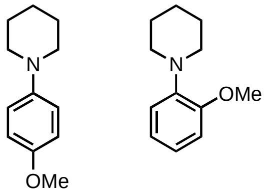

Following **GP4**, **1a** (27 mg, 0.1 mmol) gave **3f** (7 mg, 35%) as an oil and **3f'** (8 mg, 42%) as an oil. **3f**:**3f'** = 1:1.2.

Data for **3f**: ^{1}H NMR (400 MHz, $CDCl_3$) 6.93 (2H, d, *J* = 9.0 Hz), 6.84 (2H, d, *J* = 9.0 Hz), 3.78 (3H, s), 3.04 (4H, t, *J* = 5.4 Hz), 1.76–1.71 (4H, m), 1.57–1.54 (2H, m); ^{13}C NMR (101 MHz, $CDCl_3$) 153.6, 146.9, 118.7 (x 2), 114.3 (x 2), 55.6, 52.3 (x 2), 26.1 (x 2), 24.2. Data in accordance with the literature.[7]

Data for **3f'**: ^{1}H NMR (400 MHz, $CDCl_3$) δ 7.00–6.94 (m, 2 H), 6.93–6.88 (m, 1 H), 6.86–6.83 (m, 1 H), 3.86 (s, 3 H), 2.98 (t, J = 4.8 Hz, 4 H), 1.80–1.72 (m, 4 H), 1.62–1.55 (m, 2 H); ^{13}C NMR (101 MHz, $CDCl_3$) 152.5, 142.9, 122.6, 121.0, 118.5, 111.2, 55.5, 52.5, 26.5, 24.6. Data in accordance with the literature.[8]

1-(4-phenoxyphenyl)piperidine (3g) and 1-(3-phenoxyphenyl)piperidine (3g')

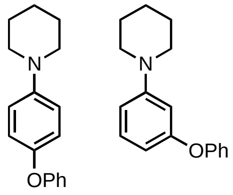

Following **GP4**, **1a** (27 mg, 0.1 mmol) gave **3g** (17 mg, 68%) as an oil **3g'** (6 mg, 22%) as an oil. **3g**:**3g'** = 3:1.

Data for **3g**: R_f 0.27 [petrol–EtOAc (95:5)]; FT-IR ν_{max} (film)/cm^{-1} 2933, 2852, 1505, 1488, 1227; ^{1}H NMR (500 MHz, $CDCl_3$) 7.29 (2H, t, *J* = 7.5 Hz), 7.02 (1H, t, *J* = 7.4 Hz), 6.97–6.91 (6H, m), 3.10 (4H, t, *J* = 5.4 Hz), 1.75–1.71 (4H, m), 1.59–1.55 (2H, m); ^{13}C NMR (126 MHz, $CDCl_3$) δ 158.6, 149.4, 149.0, 129.5 (x 2), 122.2, 120.4 (x 2), 118.1 (x 2), 117.5 (x 2), 51.5 (x 2), 26.0 (x 2), 24.2; HRMS (APCI): Found MH^+ 254.1532 $C_{17}H_{20}NO$ requires 254.1539.

Data for **3g'**: R_f 0.32 [petrol–EtOAc (95:5)]; FT-IR ν_{max} (film)/cm^{-1} 2933, 1590, 1488, 1243, 1222; ^{1}H NMR (500 MHz, CD_3OD) δ 7.27 (2H, dd, *J* = 8.5, 7.5 Hz), 7.10 (1H, td, *J* = 7.7, 7.2, 1.5 Hz), 7.06 (1H, dd, *J* = 8.0, 1.7 Hz), 7.01 (1H, t, *J* = 7.4 Hz), 6.96 (1H, td, *J* = 7.5, 1.8 Hz), 6.90 (1H, dd, *J* = 7.9, 1.4 Hz), 6.86 (2H, d, *J* = 7.8 Hz), 3.01–2.95 (4H, m), 1.56–1.43 (6 H, m). ^{13}C NMR (126 MHz, CD_3OD) δ 159.1, 150.0, 146.6, 130.4 (x 2), 125.9, 123.7, 123.2, 122.3, 120.8, 118.0 (x 2), 53.2, 27.2, 25.3; HRMS (APCI): Found MH^+ 254.1530 $C_{17}H_{20}NO$ requires 254.1539.

1-(3-(benzyloxy)phenyl)piperidine (3h) and (3h')

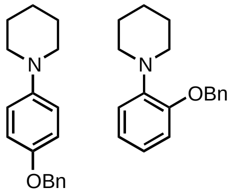

Following **GP4**, **1a** (27 mg, 0.1 mmol) gave **3h** (11 mg, 39%) as an oil and **3h'** (11 mg, 39%) as an oil. **3h**:**3h'** = 1:1.

Data for **3h**: R_f 0.35 [petrol–EtOAc (95:5)]; FT-IR ν_{max} (film)/cm^{-1} 2932, 2855, 1511, 1452, 1250; ^{1}H NMR (500 MHz, $CDCl_3$) δ 7.43 (2H, d, *J* = 7.5 Hz), 7.37 (2H, t, *J* = 7.4 Hz), 7.31 (1H, t, *J* = 7.1 Hz), 6.91 (4H, s), 5.01 (2H, s), 3.03 (4H, t, *J* = 5.3 Hz), 1.77–1.67 (4H, m), 1.58–1.50 (2H, m). ^{13}C NMR (126 MHz, $CDCl_3$) δ 152.8, 147.2,

137.6, 128.7, 128.0 (x 2), 127.6 (x 2), 118.8 (x 2), 115.5 (x 2), 70.6, 52.3, 26.3, 24.3. HRMS (APCI): Found MH^+ 268.1689 $C_{18}H_{22}NO$ requires 268.1696.

Data for **3h'**: R_f 0.38 [petrol–EtOAc (95:5)]; FT-IR ν_{max} (film)/cm^{-1} 2931, 2852, 1501, 1449, 1222; ^1H NMR (500 MHz, CD_3OD) δ 7.51 (2H, d, *J* = 7.1 Hz), 7.38 (2H, t, *J* = 7.4 Hz), 7.31 (1H, t, *J* = 7.3 Hz), 7.00 (2H, dd, *J* = 7.4, 1.3 Hz), 6.96 (1H, td, *J* = 7.4, 1.9 Hz), 6.91 (1H, td, *J* = 7.4, 1.7 Hz), 5.13 (2H, s), 3.05–2.96 (4H, m), 1.77–1.67 (3H, m), 1.62–1.52 (2H, m); ^{13}C NMR (126 MHz, CD_3OD) δ 153.1, 144.1, 139.0, 129.4 (x 2), 128.7, 128.3 (x 2), 124.2, 122.6, 119.8, 114.7, 71.4, 53.7, 27.3, 25.4; HRMS (APCI): Found MH^+ 268.1685 $C_{18}H_{22}NO$ requires 268.1696.

1-(2,4-Dimethoxyphenyl)piperidine (3i)

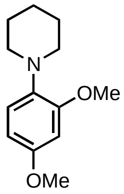

Following **GP4**, **1a** (27 mg, 0.1 mmol) gave **3i** (16 mg, 70%) as an oil. ^1H NMR (500 MHz, $CDCl_3$) δ 6.87 (1H, d, *J* = 8.6 Hz), 6.47 (1H, d, *J* = 2.7 Hz), 6.41 (1H, dd, *J* = 8.6, 2.7 Hz), 3.84 (3H, s), 3.77 (3H, s), 2.90 (4H, t, *J* = 5.2 Hz), 1.78–1.71 (4H, m), 1.56–1.51 (2H, m); ^{13}C NMR (126 MHz, $CDCl_3$) δ 155.8, 153.5, 136.7, 118.5, 103.1, 99.7, 55.5, 55.4, 52.9 (x 2), 26.4 (x 2), 24.4. Data in accordance with the literature.[9]

1-(2,4,6-Trimethoxyphenyl)piperidine (3j)

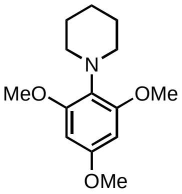

Following **GP4**, **1a** (27 mg, 0.1 mmol) gave **3j** (17 mg, 79%) as an oil. ^1H NMR (500 MHz, $CDCl_3$) δ 6.13 (2H, s), 3.80 (6H, s), 3.77 (3H, s), 3.01 (4H, t, *J* = 5.1 Hz), 1.74–1.58 (4H, m), 1.52 (2H, p, 6.1 Hz); ^{13}C NMR (126 MHz, $CDCl_3$) δ 151.7, 144.2, 124.9, 121.8, 113.3, 112.6, 55.8, 52.2 (x 2), 26.3 (x 2), 24.4. Data in accordance with the literature.[9]

1-(3-Fluoro-2-methoxyphenyl)piperidine (3k)

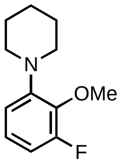

Following **GP4**, **1a** (27 mg, 0.1 mmol) gave **3k** (14 mg, 68%) as an oil. R_f 0.19 [petrol–EtOAc (98:2)]; FT-IR ν_{max} (film)/cm^{-1} 2932, 2853, 1578, 1516, 1452, 1274, 1229, 1123, 1024; ^{1}H NMR (500 MHz, $CDCl_3$) δ 6.87 (1H, t, *J* = 8.9 Hz), 6.72 (1H, app br dd, *J* = 14.3, 2.8 Hz), 6.62 (1H, br d, *J* = 8.9 Hz), 3.83 (3H, s), 3.04 (4H, t, *J* = 5.5 Hz), 1.70 (4H, p, *J* = 5.8 Hz), 1.55 (2H, p, *J* = 5.6 Hz); ^{13}C NMR (126 MHz, $CDCl_3$) δ 153.1 (d, *J* = 243.6 Hz), 147.5, 141.0 (d, *J* = 11.3 Hz), 114.9 (d, *J* = 3.2 Hz), 112.2, 106.1 (d, *J* = 21.1 Hz), 57.2, 51.7 (x 2), 26.0 (x 2), 24.3; ^{19}F NMR (375 MHz, $CDCl_3$) –177.8; HRMS (ASAP POS): Found MH^+ 210.1283 $C_{12}H_{17}NOF$ requires 210.1289.

1-(5-Bromo-2-methoxyphenyl)piperidine (3l)

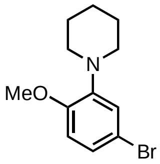

Following **GP4**, **1a** (27 mg, 0.1 mmol) gave **3l** (14 mg, 55%) as an oil. R_f 0.25 [petrol–EtOAc (95:5)]; FT-IR ν_{max} (film)/cm^{-1} 2934, 2852, 2804, 1584, 1559, 1440, 1241, 1224, 1129; ^{1}H NMR (500 MHz, $CDCl_3$) δ 7.05 (1H, dd, *J* = 8.6, 2.4 Hz), 7.01 (1H, d, *J* = 2.4 Hz), 6.69 (1H, d, *J* = 8.5 Hz), 3.84 (3H, s), 2.95 (4H, t, *J* = 5.3 Hz), 1.81–1.69 (4H, m), 1.60–1.53 (2H, m); ^{13}C NMR (126 MHz, $CDCl_3$) δ 151.7, 144.2, 124.9, 121.8, 113.3, 112.6, 55.8, 52.2 (x 2), 26.3 (x 2), 24.4; HRMS (APCI): Found MH^+ 270.0485 $C_{12}H_{17}NOBr$ requires 270.0488.

1-(Benzo[d][1,3]dioxol-5-yl)piperidine (3m)

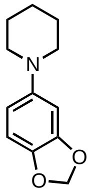

Following **GP4**, **1a** (27 mg, 0.1 mmol) gave **3m** (12 mg, 56%) as an oil. ^{1}H NMR (500 MHz, $CDCl_3$) δ 6.70 (1H, d, *J* = 8.4 Hz), 6.57 (1H, d, *J* = 2.4 Hz), 6.38 (1H, dd,

J = 8.4, 2.4 Hz), 5.88 (2H, s), 3.23–2.84 (4H, m), 1.70 (4H, p, J = 5.7 Hz), 1.55 (2H, dt, J = 9.9, 6.3 Hz); ^{13}C NMR (126 MHz, $CDCl_3$) δ 148.8, 148.2, 141.4, 109.7, 108.2, 100.9, 100.6, 52.6 (x 2), 26.3 (x 2), 24.3. Data in accordance with the literature.[9]

6-(Piperidin-1-yl)chroman-4-one (3n)

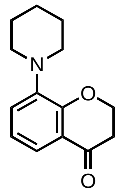

Following **GP4**, **1a** (27 mg, 0.1 mmol) gave **3n** (8 mg, 34%) as an oil. R_f 0.09 [petrol–EtOAc (98:2)]; FT-IR ν_{max} (film)/cm^{-1} 2932, 2852, 1688, 1579, 1483, 1443, 1291, 1153, 1029; ^{1}H NMR (400 MHz, $CDCl_3$) δ 7.56 (1H, dd, J = 7.9, 1.6 Hz), 7.11 (1H, dd, J = 7.8, 1.7 Hz), 6.94 (1H, t, J = 7.8 Hz), 4.61 (2H, t, J = 6.4 Hz), 2.98 (4H, br t, J = 5.3 Hz), 2.81 (2H, t, J = 6.4 Hz), 1.76 (4H, br p, J = 5.7 Hz), 1.63–1.54 (2H, m); ^{13}C NMR (101 MHz, $CDCl_3$) δ 192.5, 155.4, 143.2, 124.4, 122.0, 121.2, 120.2, 67.3, 52.5 (x 2), 37.7, 26.3 (x 2), 24.4; HRMS (ASAP POS): Found MH^+ 232.1324 $C_{14}H_{18}NO_2$ requires 232.1332.

1-(Naphthalen-1-yl)piperidine (3o)

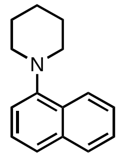

Following **GP4**, **1a** (27 mg, 0.1 mmol) gave **3o** (19 mg, 90%) as an oil. ^{1}H NMR (500 MHz, $CDCl_3$) δ 8.32–8.08 (1H, m, 1H), 7.82 (1H, dd, J = 6.8, 2.4 Hz), 7.53 (1H, d, J = 8.2 Hz), 7.47 (2H, ddd, J = 6.9, 4.3, 1.9 Hz), 7.40 (1H, t, J = 7.8 Hz), 7.07 (1 H, d, J = 7.3 Hz), 3.32–2.84 (4H, br s), 1.86 (4H, q, J = 5.6 Hz), 1.68 (2 H, br s); ^{13}C NMR (126 MHz, $CDCl_3$) δ 150.3, 134.9, 128.9, 128.5, 125.9, 125.9, 125.5, 123.9 (x 2), 114.8, 55.0 (x 2), 26.5 (x 2), 24.5. Data in accordance with the literature.[7]

1-(2-Methoxynaphthalen-1-yl)piperidine (3p)

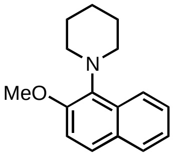

Following **GP4**, **1a** (27 mg, 0.1 mmol) gave **3p** (18 mg, 75%) as an oil. R_f 0.39 [petrol–EtOAc (98:2)]; FT-IR ν_{max} (film)/cm^{-1} 2932, 2850, 1683, 1633, 1603, 1455, 1280, 1262, 1087; ^{1}H NMR (500 MHz, $CDCl_3$) δ 8.43 (1H, d, *J* = 8.5 Hz), 7.74 (1H, d, *J* = 8.1 Hz), 7.63 (1H, d, *J* = 8.9 Hz), 7.46 (1H, t, *J* = 7.6 Hz), 7.33 (1H, t, *J* = 7.5 Hz), 7.24 (1H, d, *J* = 8.9 Hz), 3.93 (3H, s), 3.39–3.31 (2H, m), 2.97 (2H, br d, *J* = 11.4 Hz), 1.96–1.75 (3H, m), 1.84–1.78 (1H, m), 1.77–1.68 (2H, m), 1.67–1.59 (2H, m), 1.38–1.30 (1H, m),; ^{13}C NMR (126 MHz, $CDCl_3$) δ 154.7, 135.7, 133.4, 130.0, 127.7, 126.1, 126.0, 124.0, 123.9, 115.7, 56.9, 52.2 (x 2), 27.5 (x 2), 24.8; HRMS (APCI): Found MH^+ 242.1537 $C_{16}H_{20}NO$ requires 242.1539.

1-(2-Bromonaphthalen-1-yl)piperidine (3q) and (3q')

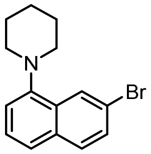

Following **GP4**, **1a** (27 mg, 0.1 mmol) gave **3q** (19 mg, 65%) as an oil. **3q**:**3q'** = 3:1.
Data for **3q**: R_f 0.45 [petrol–EtOAc (98:2)]; FT-IR ν_{max} (film)/cm^{-1} 2933, 2806, 1595, 1387, 1253, 1229, 1021; ^{1}H NMR (400 MHz, $CDCl_3$) δ 8.27 (1H, d, *J* = 2.0 Hz), 7.60 (1H, d, *J* = 8.7 Hz), 7.44 (1H, dd, *J* = 8.7, 2.0 Hz), 7.40 (1H, d, *J* = 8.2 Hz), 7.32 (1H, t, *J* = 7.8 Hz), 7.02 (1H, dd, *J* = 7.3, 1.1 Hz), 2.95 (4H, br s), 1.78 (4H, p, *J* = 5.7 Hz), 1.59 (2H, br s); ^{13}C NMR (101 MHz, $CDCl_3$) δ 150.6, 133.2, 130.63, 130.1, 129.2, 126.5, 126.4, 122.9, 119.7, 115.9, 54.8 (x 2), 26.7 (x 2), 24.7; HRMS (ASAP POS): Found MH^+ 290.0531 $C_{15}H_{17}NBr$ requires 290.0539.

Data for **3q'**: R_f 0.34 [petrol–EtOAc (98:2)]; FT-IR ν_{max} (film)/cm^{-1} 2940, 2821, 1591, 1382, 1249, 1235, 1028; ^{1}H NMR (400 MHz, $CDCl_3$) δ 8.06 (1H, d, *J* = 9.0 Hz), 7.95 (1H, d, *J* = 2.0 Hz), 7.52 (1H, dd, *J* = 9.0, 2.0 Hz), 7.42–7.37 (2H, m), 7.06 (1H, dd, *J* = 5.2, 3.3 Hz), 3.02 (4H, s), 1.83 (4H, p, *J* = 5.7 Hz), 1.66 (2H, br s); ^{13}C NMR (126 MHz, $CDCl_3$) δ 151.5, 136.1, 130.3, 128.5, 127.8, 127.3, 126.1, 122.1, 120.0, 115.1, 54.8 (x 2), 26.7 (x 2), 24.7; HRMS (ASAP POS): Found MH^+ 290.0540 $C_{15}H_{17}NBr$ requires 290.0539

1-(8-(piperidin-1-yl)naphthalen-2-yl)ethanone (3r) , 1-(5-(piperidin-1-yl)naphthalen-2-yl)ethanone (3r')

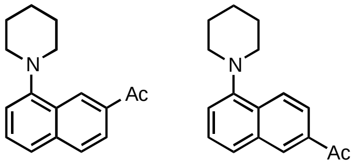

Following **GP4**, **1a** (27 mg, 0.1 mmol) gave **3r** (13 mg, 50%) as an oil and **3r'** (5 mg, 20%) as an oil. **3r**:**3r'** = 2.3:1.

Data for **3r**: R_f 0.32 [petrol–EtOAc (95:2)]; FT-IR ν_{max} (film)/cm^{-1} 2932, 2851, 1677, 1442, 1271, 1257; ^{1}H NMR (500 MHz, $CDCl_3$) δ 8.86 (1H, d, *J* = 1.8 Hz), 8.01 (1H, dd, *J* = 8.6, 1.8 Hz), 7.84 (1H, d, *J* = 8.6 Hz), 7.58–7.44 (2H, m), 7.11 (1H, dd, *J* = 6.5, 2.1 Hz), 3.09 (4H, br s), 2.75 (3H, s), 1.96–1.84 (4H, m), 1.70 (2H, br s); ^{13}C NMR (126 MHz, $CDCl_3$) δ 198.5, 152.7, 137.2, 133.8, 128.9 (x 2), 128.3, 126.8, 123.7, 122.6, 115.5, 55.0 (x 2), 26.8, 26.7 24.7; HRMS (APCI): Found MH$^+$ 254.1532 $C_{17}H_{20}NO$ requires 254.1539.

Data for **3r'**: R_f 0.28 [petrol–EtOAc (95:2)]; FT-IR ν_{max} (film)/cm^{-1} 2930, 2851, 1678, 1371, 1270, 1219; ^{1}H NMR (500 MHz, $CDCl_3$) δ 8.43 (1H, d, *J* = 1.8 Hz), 8.24 (1H, d, *J* = 8.9 Hz), 8.01 (1H, dd, *J* = 8.9, 1.8 Hz), 7.62 (1H, d, *J* = 8.4 Hz), 7.46 (1H, t, *J* = 7.8 Hz), 7.17 (1H, dd, *J* = 7.5, 1.1 Hz), 3.05 (4H, br s), 2.72 (3H, s), 1.89–1.82 (4H, m), 1.67 (2H, br s); ^{13}C NMR (126 MHz, $CDCl_3$) δ 198.3, 151.1, 134.3, 133.9, 131.4, 130.6, 126.9, 124.5, 124.2, 123.1, 117.0, 54.6, 26.7, 26.6, 24.5; HRMS (APCI): Found MH$^+$ 254.1529 $C_{17}H_{20}NO$ requires 254.1539.

1-(1-Bromonaphthalen-2-yl)piperidine (3s)

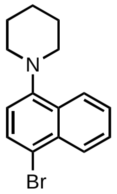

Following **GP4**, **1a** (27 mg, 0.1 mmol) gave **3s** (16 mg, 57%) as an oil. ^{1}H NMR (400 MHz, $CDCl_3$) δ 8.21 (2H, dd, *J* = 9.2, 7.3 Hz), 7.67 (1H, dd, *J* = 8.0, 1.3 Hz), 7.62–7.47 (2H, m), 6.92 (1H, d, *J* = 8.0 Hz), 3.02 (4H, s), 1.84 (4H, t, *J* = 5.7 Hz), 1.66 (2H, s); ^{13}C NMR (101 MHz, $CDCl_3$) δ 132.9, 129.9, 127.7, 127.2, 126.0, 125.5, 124.42, 115.4, 54.8 (x 2), 26.7 (x 2), 24.7. Data in accordance with the literature.[10]

1-(4-Chloronaphthalen-1-yl)piperidine (3t)

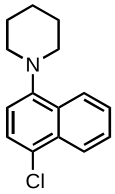

Following **GP4**, **1a** (27 mg, 0.1 mmol) gave **3t** (18 mg, 75%) as an oil. R_f 0.45 [petrol–EtOAc (98:2)]; FT-IR ν_{max} (film)/cm^{-1} 2933, 2803, 1585, 1381, 1255, 1225, 1009; ^{1}H NMR (400 MHz, $CDCl_3$) δ 8.26–8.24 (1H, m), 8.23–8.21 (1H, m), 7.62–7.48 (2H, m), 7.47 (1H, d, *J* = 8.0 Hz), 6.97 (1H, d, *J* = 8.1 Hz), 3.02 (4H, br s), 1.84 (4H, p, *J* = 5.6 Hz), 1.66 (2H, br s); ^{13}C NMR (101 MHz, $CDCl_3$) δ 150.6, 131.8, 130.4, 126.9, 126.2, 126.1, 126.0, 125.0, 124.4, 114.8, 54.9 (x 2), 26.7 (x 2), 24.7; HRMS (APCI): Found MH$^+$ 246.1038 $C_{15}H_{17}NCl$ requires 246.1044.

5-(Piperidin-1-yl)-1-naphthonitrile (3u)

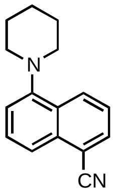

Following **GP4** but using 3.0 equiv. of 1-naphthonitrile, **1a** (27 mg, 0.1 mmol) gave **3u** (14 mg, 57%) as an oil. R_f 0.25 [petrol]; FT-IR ν_{max} (film)/cm^{-1} 2923, 2850, 2222, 1579, 1411, 1222; ^{1}H NMR (500 MHz, $CDCl_3$) δ 8.45 (1H, dd, *J* = 8.6, 1.2 Hz), 7.91 (1H, dd, *J* = 8.4, 1.0 Hz), 7.89 (1H, dd, *J* = 7.1, 1.2 Hz), 7.59 (1H, dd, *J* = 8.4, 7.5 Hz), 7.50 (1H, dd, *J* = 8.6, 7.1 Hz), 7.17 (1H, dd, *J* = 7.5, 1.0 Hz), 3.03 (4H, br s), 1.90–1.79 (4H, m), 1.67 (2H, br s); ^{13}C NMR (126 MHz, $CDCl_3$) δ 152.1, 134.0, 132.7, 129.6, 129.0, 128.9, 124.2, 119.9, 118.4, 116.3, 110.5, 54.9 (x 2), 26.7 (x 2), 24.6; HRMS (APCI): Found MH$^+$ 237.1377 $C_{16}H_{17}N_2$ requires 237.1386.

1-(9H-Fluoren-2-yl)piperidine (3v)

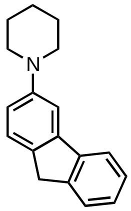

Following **GP4**, **1a** (27 mg, 0.1 mmol) gave **3v** (20 mg, 80%) as an oil. R_f 0.35 [petrol–EtOAc (98:2)]; FT-IR ν_{max} (film)/cm^{-1} 2933, 2853, 1612, 1572, 1455, 1276, 1222, 1119; ^{1}H NMR (500 MHz, $CDCl_3$) δ 7.66 (2H, app t, *J* = 8.4 Hz), 7.49 (1H, d, *J* = 7.4 Hz), 7.33 (1H, t, *J* = 7.5 Hz), 7.21 (1H, td, *J* = 7.4, 1.1 Hz), 7.16 (1H, d, *J* = 2.2 Hz), 6.99 (1H, dd, *J* = 8.4, 2.3 Hz), 3.85 (2H, s), 3.37–2.97 (4H, m), 1.76 (4H, p, *J* = 6.0 Hz), 1.61 (2H, dtd, *J* = 8.8, 5.3, 4.6, 2.2 Hz); ^{13}C NMR (126 MHz, $CDCl_3$) δ 152.0, 144.8, 142.8, 142.2, 133.6, 126.7, 125.3, 124.9, 120.4, 119.0, 115.8, 113.5, 51.4 (x 2), 37.2, 26.1 (x 2), 24.5; HRMS (APCI): Found MH$^+$ 250.1587 $C_{18}H_{20}N$ requires 250.1590.

2-(Piperidin-1-yl)-9H-fluoren-9-one (3w) and 4-(piperidin-1-yl)-9*H*-fluoren-9-one (3w')

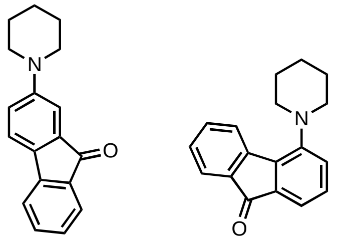

Following **GP4**, **1a** (27 mg, 0.1 mmol) gave **3w** (17 mg, 65%) as an oil. dr = 3:1.
Data for **3w**: R_f 0.24 [petrol–EtOAc (98:2)]; FT-IR ν_{max} (film)/cm^{-1} 2926, 2854, 1598, 1497, 1440, 1378, 1230; ^{1}H NMR (500 MHz, $CDCl_3$) δ 7.56 (1H, d, *J* = 7.3 Hz), 7.39 (1H, t, *J* = 7.4 Hz), 7.37–7.32 (2H, m), 7.24 (1H, s), 7.14 (1H, t, *J* = 7.4 Hz), 6.94 (1H, dd, *J* = 8.3, 2.2 Hz), 3.23 (4H, t, *J* = 5.5 Hz), 1.70 (4H, p, *J* = 5.6 Hz), 1.61 (2H, q, *J* = 6.0 Hz); ^{13}C NMR (126 MHz, $CDCl_3$) δ 194.9, 153.1, 145.6, 135.7, 134.9, 134.5, 134.4, 127.4, 124.3, 121.2, 120.7, 119.4, 112.2, 50.3 (x 2), 25.7 (x 2), 24.4; HRMS (ASAP POS): Found MH$^+$ 264.1378 $C_{18}H_{18}NO$ requires 264.1383.

1-(Dibenzo[b,d]thiophen-3-yl)piperidine (3y) and 1-(dibenzo[*b*,*d*]thiophen-2-yl)piperidine (3y')

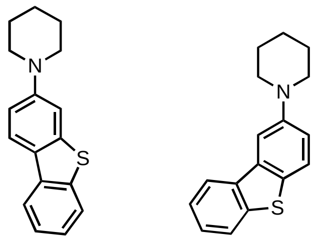

Following **GP4**, **1a** (28.1 mg, 0.1 mmol) gave **3y** (18 mg, 68%) as an oil. dr = 4:1. Data for **3y**: R_f 0.24 [petrol–EtOAc (98:2)]; FT-IR ν_{max} (film)/cm^{-1} 2960, 1716, 1669, 1453, 1258, 1074, 1016; ^{1}H NMR (400 MHz, $CDCl_3$) δ 8.07–7.92 (2H, m), 7.79–7.73 (1H, m), 7.46–7.29 (3H, m), 7.12 (1H, dd, *J* = 8.7, 2.3 Hz), 3.27 (4H, d, *J* = 5.5 Hz), 1.76 (4H, p, *J* = 5.8 Hz), 1.62 (2H, p, *J* = 6.2 Hz ^{13}C NMR (126 MHz, $CDCl_3$) δ 151.7, 141.2, 138.6, 136.0, 127.8, 125.2, 124.4, 122.7, 122.0, 120.6, 115.4, 108.8, 51.11 (x 2), 26.0 (x 2), 24.5; HRMS (APCI): Found MH^+ 268.1159 $C_{17}H_{18}NS$ requires 268.1159.

1-(Azulen-1-yl)piperidine (3y)

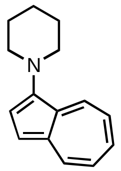

Following **GP4**, **1a** (27 mg, 0.1 mmol) gave **3x** (13 mg, 71%) as an oil. R_f 0.27 [petrol–EtOAc (95:5)]; FT-IR ν_{max} (film)/cm^{-1} 2933, 2852, 2788, 1571, 1504, 1451, 1392, 1378; ^{1}H NMR (400 MHz, $CDCl_3$) δ 8.27 (1H, d, *J* = 9.5 Hz), 8.11 (1H, d, *J* = 9.4 Hz), 7.64 (1H, d, *J* = 4.0 Hz), 7.43 (1H, t, *J* = 9.8 Hz), 7.24 (1H, d, *J* = 4.0 Hz, 1H), 6.92 (1H, d, *J* = 9.9 Hz), 6.87 (1H, d, *J* = 9.9 Hz), 3.10 (4H, t, *J* = 5.4 Hz), 1.84 (4H, p, *J* = 5.7 Hz), 1.64 (2H, td, *J* = 7.1, 6.3, 4.5 Hz); ^{13}C NMR (101 MHz, $CDCl_3$) δ 144.2, 138.3, 137.3, 137.1, 133.7, 129.0, 126.7, 121.1, 120.1, 115.1, 55.9 (x 2), 26.8 (x 2), 24.7; HRMS (APCI): Found MH^+ 212.1431 $C_{15}H_{18}N$ requires 212.1434.

8-Methoxy-5-(piperidin-1-yl)quinolone (3z)

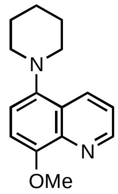

Following **GP4** but using 4.0 equiv. of $HClO_4$, **1a** (27 mg, 0.1 mmol) gave **3z** (12 mg, 50%) as an oil. R_f 0.50 [Et_2O-petrol-acetone (55:40:5)]; FT-IR ν_{max} (film)/cm^{-1} 2930, 2851, 1464, 1253, 1107; ^1H NMR (500 MHz, $CDCl_3$) δ 8.91 (1H, dd, *J* = 4.2, 1.8 Hz), 8.53 (1H, dd, *J* = 8.5, 1.8 Hz), 7.43 (1H, dd, *J* = 8.5, 4.2 Hz), 7.05 (1H, d, *J* = 8.3 Hz), 6.96 (1H, d, *J* = 8.3 Hz), 4.05 (3H, s), 2.96 (4H, br s), 1.86–1.77 (4H, m), 1.64 (2H, br s); ^{13}C NMR (126 MHz, $CDCl_3$) δ 151.7, 149.2, 144.0, 141.1, 132.5, 125.6, 121.0, 115.2, 107.2, 56.1, 55.1 (x 2), 26.8 (x 2), 24.6; HRMS (APCI): Found MH^+ 243.1483 $C_{15}H_{19}N_2O$ requires 243.1492.

1-(1-(4-Methoxyphenyl)-1H-pyrrol-2-yl)piperidine (3aa)

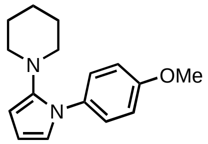

Following **GP4**, **1a** (27 mg, 0.1 mmol) gave **3aa** (13.1 mg, 51%) as an oil. R_f 0.15 [petrol–EtOAc (98:2)]; FT-IR ν_{max} (film)/cm^{-1} 2937, 2855, 1713, 1609, 1513, 1443, 1248, 1169, 1035; ^1H NMR (500 MHz, $CDCl_3$) δ 7.47 (2H, d, *J* = 8.9 Hz), 7.04–6.73 (2H, m), 6.63–6.53 (1H, m), 6.12 (1H, t, *J* = 3.4 Hz), 5.68 (1H, dd, *J* = 3.5, 2.0 Hz), 3.84 (3H, s), 2.71 (4H, br t, *J* = 5.1 Hz), 1.49 (4H, p, *J* = 5.6 Hz), 1.47–1.42 (2H, m); ^{13}C NMR (126 MHz, $CDCl_3$) δ 157.7, 144.8, 133.5, 125.6 (x 2), 117.2, 114.0 (x 2), 106.8, 94.5, 55.6, 53.4 (x 2), 26.1 (x 2), 24.3; HRMS (APCI): Found MH^+ 257.1645 $C_{16}H_{21}N_2O$ requires 257.1648.

1-Methyl-2-(piperidin-1-yl)-1H-indole (3ab)

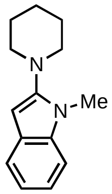

Following **GP4** but using 4.0 equiv. N-methylindole, **1a** (27 mg, 0.1 mmol) gave **3ab** (9.6 mg, 45%) as an oil. ^{1}H NMR (500 MHz, $CDCl_3$) δ 7.46 (1H, d, *J* = 7.8 Hz), 7.20 (1H, d, *J* = 8.0 Hz), 7.14–7.07 (1H, m), 7.08–7.01 (1H, m), 5.87 (1H, s), 3.60 (3H, s), 2.96 (4H, t, *J* = 5.5 Hz), 1.75 (4H, p, *J* = 5.7 Hz), 1.65 – 1.58 (2H, m); ^{13}C NMR (126 MHz, $CDCl_3$) δ 151.8, 135.4, 127.9, 112.0, 119.5, 119.2, 108.7, 86.4, 53.90, 29.2, 26.2, 24.4. Data in accordance with the literature.[11]

4,4-Difluoro-1-(naphthalen-1-yl)piperidine (3ac)

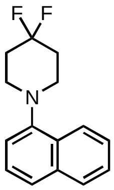

Following **GP4**, **1b** (30 mg, 0.1 mmol) gave **3ac** (14 mg, 57%) as an oil. R_f 0.24 [petrol–EtOAc (98:2)]; FT-IR ν_{max} (film)/cm^{-1} 2923, 2850, 1644, 1585, 1569, 1396, 1299, 1087; ^{1}H NMR (500 MHz, $CDCl_3$) δ 8.22–8.06 (1H, m), 7.92–7.77 (1H, m), 7.65–7.56 (1H, m), 7.55–7.46 (2H, m), 7.45–7.37 (1H, m), 7.19–7.06 (1H, m), 3.23 (4H, s), 2.28 (4H, ddd, *J* = 16.7, 11.1, 5.5 Hz); ^{13}C NMR (126 MHz, $CDCl_3$) δ 149.1, 134.9, 129.0, 128.6, 126.1, 125.9, 125.8, 124.2, 123.3, 122.2 (t, *J* = 242.4 Hz), 115.4, 50.2 (t, *J* = 5.5 Hz), 35.0 (t, *J* = 22.8 Hz); ^{19}F NMR (376 MHz, $CDCl_3$) δ 102.7, 92.1; HRMS (ASAP POS): Found MH^+ 248.1235 $C_{15}H_{15}NF_2$ requires 248.1245.

1-(Naphthalen-1-yl)piperidin-4-one (3ad)

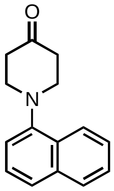

Following **GP4**, **1c** (28 mg, 0.1 mmol) gave **3ad** (20 mg, 70%) as an oil. R_f 0.11 [petrol–EtOAc (98:2)]; FT-IR ν_{max} (film)/cm^{-1} 2962, 1716, 1635, 1540, 1396, 1258,

1017; 1H NMR (500 MHz, $CDCl_3$) δ 8.26 (1H, dd, *J* = 8.3, 1.4 Hz), 7.86 (1H, dd, *J* = 7.7, 1.6 Hz), 7.61 (1H, d, *J* = 8.2 Hz), 7.56–7.49 (2H, m), 7.41 (1H, t, *J* = 7.8 Hz), 7.12 (1H, dd, *J* = 7.4, 1.0 Hz), 3.42 (4H, br s), 2.74 (4H, t, *J* = 6.1 Hz); ^{13}C NMR (126 MHz, $CDCl_3$) δ 208.7, 148.9, 134.9, 129.0, 128.7, 126.2, 125.9, 125.9, 124.3, 123.2, 115.4, 53.2 (x 2), 42.3 (x 2); HRMS (ASAP POS): Found MH^+ 226.1217 $C_{15}H_{16}NO$ requires 226.1226.

2-Methyl-1-(naphthalen-1-yl)pyrrolidine (3ae)

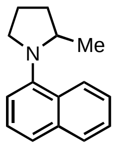

Following **GP4**, **1d** (27 mg, 0.1 mmol) gave **3ae** (15 mg, 70%) as an oil. ^{1}H NMR (400 MHz, $CDCl_3$) δ 8.29–8.19 (1H, m), 7.85–7.74 (1H, m), 7.55–7.33 (4H, m), 7.04 (1H, dd, *J* = 7.5, 2.3 Hz), 3.95–3.70 (2H, m), 3.03–2.78 (1H, m), 2.24 (1H, dt, *J* = 11.3, 7.7 Hz), 2.01 (1H, dt, *J* = 12.1, 4.1 Hz), 1.93–1.80 (1H, m), 1.77–1.63 (1H, m), 1.08 (3H, d, *J* = 5.5 Hz); ^{13}C NMR (101 MHz, $CDCl_3$) δ 147.0, 135.0, 128.2, 125.9, 125.7, 125.7 124.9, 124.7, 122.1, 114.2, 55.8, 55.5, 33.8, 23.7, 18.8. Data in accordance with the literature.[12]

1-Methyl-4-(naphthalen-1-yl)piperazine (3af)

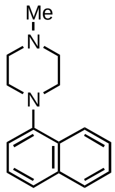

Following **GP4** but using 4.0 equiv. $HClO_4$, **1e** (28 mg, 0.1 mmol) gave **3af** (20 mg, 90%) as an oil. ^{1}H NMR (400 MHz, $CDCl_3$) δ 8.20–8.17 (1H, m), 7.81–7.75 (1H, m), 7.53 (1H, d, *J* = 8.0 Hz), 7.48-7.40 (2H, m), 7.36 (1H, t, *J* = 7.9 Hz), 7.07 (1H, t, *J* = 7.6 Hz), 3.12 (4H, br s), 2.72 (2H, br s), 2.38 (3H, s); ^{13}C NMR (101 MHz, $CDCl_3$) δ 149.7, 134.8, 129.0, 128.4, 125.9, 125.8, 125.3, 123.6, 123.5, 114.7, 66.7, 52.9, 46.2. Data in accordance with the literature.[11a]

Benzyl 4-(Naphthalen-1-yl)piperazine-1-carboxylate (3ag)

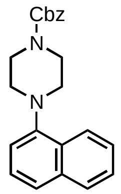

Following **GP4**, **1e** (40 mg, 0.1 mmol) gave **3ag** (21 mg, 61%) as an oil. R_f 0.34 [petrol–EtOAc (98:2)]; FT-IR ν_{max} (film)/cm^{-1} 2945, 2872, 1602, 1495, 1460, 1398, 1244; ^{1}H NMR (500 MHz, $CDCl_3$) δ 8.34–8.12 (1H, m), 7.98–7.74 (1H, m), 7.58 (1H, d, *J* = 8.2 Hz), 7.53–7.46 (2H, m), 7.45–7.30 (6H, m), 7.07 (1H, dd, *J* = 7.4, 0.9 Hz), 5.20 (2H, s), 4.36–2.38 (8H, m); ^{13}C NMR (126 MHz, $CDCl_3$) δ 155.5, 149.3, 136.8, 134.9, 128.9, 128.7 (x 2), 128.6, 128.2, 128.1 (x 2), 126.1, 125.9, 125.7, 124.1, 123.3, 115.1, 67.4, 53.0 (x 2), 44.6 (x 2); HRMS (APCI): Found MH^+ 347.1766 $C_{22}H_{23}N_2O_2$ requires 347.1760.

4-(Naphthalen-1-yl)morpholine (3ah)

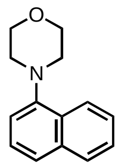

Following **GP4**, **1f** (27 mg, 0.1 mmol) gave **3ah** (12 mg, 57%) as an oil. ^{1}H NMR (500 MHz, $CDCl_3$) δ 8.26- 8.22 (1H, m), 7.87–7.84 (1H, m), 7.59 (1H, d, *J* = 8.14 Hz), 7.52–7.48 (2H, m), 7.43 (1H, t, *J* = 7.77 Hz), 7.12 (1H, d, *J* = 7.40 Hz), 4.03–3.98 (4H, m), 3.16–3.12 (4H, m); ^{13}C NMR (126 MHz, $CDCl_3$) δ 149.4, 134.8, 128.8, 128.4, 125.9, 125.8, 125.4, 123.8, 114.7, 67.5 (x 2), 53.5 (x 2). Data in accordance with the literature.[13]

4-(Naphthalen-1-yl)thiomorpholine (3ai)

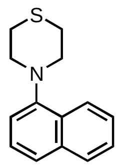

Following **GP4**, **1g** (29 mg, 0.1 mmol) gave **3ai** (9 mg, 38%) as an oil. R_f 0.44 [petrol–EtOAc (98:2)]; FT-IR ν_{max} (film)/cm^{-1} 3046, 2921, 2818, 1734, 1575, 1506, 1397, 1280, 1099; ^{1}H NMR (400 MHz, $CDCl_3$) δ 8.28–8.10 (1H, m), 7.87–7.77 (1H,

m), 7.57 (1H, dd, J = 8.2, 1.0 Hz), 7.53–7.44 (2H, m), 7.41 (1H, dd, J = 8.2, 7.4 Hz), 7.10 (1H, dd, J = 7.4, 1.1 Hz), 3.36 (4H, br s), 2.94 (4H, br s); ^{13}C NMR (126 MHz, $CDCl_3$) δ 150.8, 134.9, 129.24, 128.6, 126.0, 126.0, 125.7, 124.0, 123.4, 115.9, 55.7 (x 2), 28.8 (x 2); HRMS (ASAP POS): Found MH^+ 230.0992 $C_{14}H_{16}NS$ requires 230.0998.

1-(Naphthalen-1-yl)azepane (3aj)

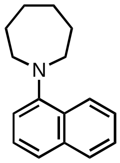

Following **GP4**, **1h** (28 mg, 0.1 mmol) gave **3aj** (20 mg, 89%) as an oil. R_f 0.24 [petrol–EtOAc (98:2)]; FT-IR ν_{max} (film)/cm^{-1} 2926, 2854, 1598, 1497, 1440, 1378, 1230; ^{1}H NMR (500 MHz, $CDCl_3$) δ 8.36–8.26 (1H, m), 7.86–7.78 (1H, m), 7.55–7.42 (3H, m), 7.38 (1H, t, J = 7.8 Hz), 7.15 (1H, d, J = 7.4 Hz), 3.28 (4H, t, J = 5.6 Hz), 2.19–1.86 (4H, m), 1.85–1.81 (4H, m); ^{13}C NMR (126 MHz, $CDCl_3$) δ 153.1, 135.0, 130.0, 128.3, 126.0, 125.8, 125.2, 124.4, 122.8, 116.3, 57.2 (x 2), 29.6 (x 2), 27.3 (x 2); HRMS (ASAP POS): Found MH^+ 226.1582 $C_{16}H_{20}N$ requires 226.1590.

***N*,*N*-Diethylnaphthalen-1-amine (3ak)**

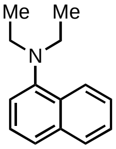

Following **GP4**, **1i** (26 mg, 0.1 mmol) gave **3ak** (18 mg, 91%) as an oil. ^{1}H NMR (500 MHz, $CDCl_3$) δ 8.35–8.29 (1H, m), 7.83–7.79 (1H, m), 7.58–7.37 (4H, m), 7.15 (1H, d, J = 7.5 Hz), 3.21 (4H, q, J = 7.2 Hz), 1.06 (6H, t, J = 7.2 Hz); ^{13}C NMR (126 MHz, $CDCl_3$) δ 148.1, 135.0, 131.4, 128.2, 125.7, 125.6, 125.2, 124.4, 123.4, 118.0, 47.9 (x 2), 12.4 (x 2). Data in accordance with the literature.[14]

***N,N*-Dibenzylnaphthalen-1-amine (3al)**

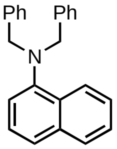

Following **GP4**, **1j** (38 mg, 0.1 mmol) gave **3al** (21 mg, 65%) as an oil. ^{1}H NMR (400 MHz, $CDCl_3$) δ 8.62 (1H, d, *J* = 8.2 Hz), 7.91 (1H, d, *J* = 8.0 Hz), 7.51–7.64 (3H, m), 7.25–7.39 (11H, m), 7.00 (1H, d, *J* = 7.4 Hz), 4.38 (4H, s); ^{13}C NMR (101 MHz, $CDCl_3$) δ 147.4, 138.2 (x 2), 134.9, 129.7, 128.5, 128.4 (x 4), 128.2 (x 4), 126.9 (x 2), 125.7, 125.5, 125.4, 123.7, 123.5, 118.4, 57.1 (x 2). Data in accordance with the literature.[15]

***N*-Methyl-*N*-phenethylnaphthalen-1-amine (3am)**

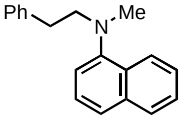

Following **GP4**, **1k** (32 mg, 0.1 mmol) gave **3am** (16 mg, 61%) as an oil. ^{1}H NMR (400 MHz, $CDCl_3$) δ 8.18–8.11 (1H, m), 7.88–7.79 (1H, m), 7.55 (1H, d, *J* = 8.2 Hz), 7.49–7.38 (3H, m), 7.30–7.24 (2H, m), 7.23–7.14 (4H, m), 3.40–3.29 (2H, m), 3.02–2.89 (2H, m), 2.95 (3H, s); ^{13}C NMR (126 MHz, $CDCl_3$) δ 150.2, 140.3, 135.0, 129.8, 129.0 (x 2), 128.5 (x 2), 128.5, 128.4, 126.2, 125.9, 125.8, 125.3, 124.1, 123.4, 115.89, 59.0, 42.7, 34.2. Data in accordance with the literature.[11a]

1,1'-(2,2'-dimethoxy-[1,1'-binaphthalene]-4,4'-diyl)dipiperidine (3an)

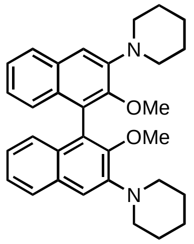

Following **GP4**, **1a** (27 mg, 0.1 mmol) gave **3an** (27 mg, 56%) as an oil. R_f 0.21 [petrol–EtOAc (95:2)]; FT-IR ν_{max} (film)/cm^{-1} 2931, 2848, 1538, 1441, 1370, 1207; ^{1}H NMR (500 MHz, $CDCl_3$) δ 8.19 (2H, d, *J* = 8.4 Hz), 7.29 (2H, t, *J* = 7.4 Hz), 7.17 (2H, t, *J* = 7.5 Hz), 7.11 (2H, d, *J* = 8.5 Hz), 7.03 (2H, s), 3.72 (6H, s), 3.19 (8H, br s), 1.91 (8H, br s), 1.71 (4H, br s); ^{13}C NMR (126 MHz, $CDCl_3$) δ 155.4 (x 2), 152.4 (x 2), 135.4 (x 2), 126.3 (x 2), 126.1 (x 2), 125.1 (x 2), 123.9 (x 2), 122.9 (x 2), 115.0 (x

2), 104.5 (x 2), 57.3 (x 2), 55.0 (x 4), 26.8 (x 4), 24.8 (x 2); HRMS (APCI): Found MH^+ 281.2836 $C_{32}H_{37}O_2N_2$ requires 281.2850

(2*R*,3*R*,4*S*,5*R*,6*S*)-2-(acetoxymethyl)-6-((4-(piperidin-1-yl)phenyl)thio) tetrahydro-2*H*-pyran-3,4,5-triyl triacetate (3ao)

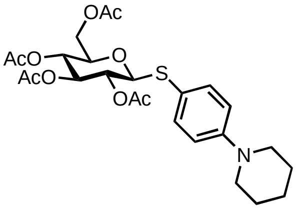

Following **GP4**, **1a** (27 mg, 0.1 mmol) gave **3ao** (25 mg, 48%) as an oil. R_f 0.22 [Et_2O–CH_2Cl_2 (5:95)]; FT-IR ν_{max} (film)/cm^{-1} 2921, 2849, 1754, 1497, 1374, 1222; ^1H NMR (500 MHz, $CDCl_3$) δ 7.36 (2H, d, *J* = 8.8 Hz), 6.83 (2H, d, *J* = 8.9 Hz), 5.19 (1H, t, *J* = 9.4 Hz), 5.00 (1H, t, *J* = 9.8 Hz), 4.90 (1H, dd, *J* = 10.0, 9.3 Hz), 4.53 (1H, d, *J* = 10.0 Hz), 4.20 (1H, *J* = 12.5, 2.8 Hz) 4.17 (1H, *J* = 12.5, 4.6 Hz), 3.66 (1H, ddd, *J* = 10.1, 4.6, 2.8 Hz), 3.24–3.18 (4H, m), 2.10 (3H, s), 2.08 (3H, s), 2.01 (3H, s), 1.98 (3H, s), 1.73–1.65 (4H, m), 1.63–1.58 (2H, m); ^{13}C NMR (126 MHz, $CDCl_3$) δ 170.8, 170.4, 169.6, 169.4, 152.5, 136.4 (x 2), 117.7, 115.9 (x 2), 86.2, 75.8, 74.3, 70.1, 68.4, 62.3, 49.8 (x 2), 25.8 (x 2), 24.4, 21.0, 21.0, 20.8, 20.8; HRMS (APCI): Found MH^+ 524.1930 $C_{25}H_{34}O_9NS$ requires 524.1949

***O*-(1-(Piperidin-1-yl)naphthalen-2-yl) methyl(*m*-tolyl)carbamothioate (3ap)**

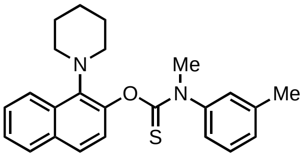

Following **GP4** but using 1.0 equiv. of the aromatic, **1a** (27 mg, 0.1 mmol) gave **3ap** (24 mg, 62%) as an oil. R_f 0.49 [petrol–Et_2O (80:20)]; FT-IR ν_{max} (film)/cm^{-1} 2930, 1590, 1463, 1375, 1289, 1203, 1156, 1115; ^1H NMR (500 MHz, $CDCl_3$) δ 7.80–7.71 (2H, m), 7.48 (1H, d, *J* = 8.3 Hz), 7.34 (2H, t, *J* = 7.7 Hz), 7.21–7.13 (4H, m), 7.05 (1H, d, *J* = 7.4 Hz), 3.76 (3H, s), 3.02 (4H, s), 2.41 (3H, s), 1.88–1.78 (4H, m), 1.76–1.58 (2H, m); 13C NMR (126 MHz, Chloroform-*d*) δ 188.4, 151.5, 151.2, 143.8, 139.6, 132.9, 129.4, 129.3, 128.6, 126.4, 125.8, 122.9, 122.8, 122.0, 116.0, 115.2, 54.7, 45.0, 26.8, 24.7, 21.5; HRMS (ASAP POS): Found MH^+ 391.1847 $C_{24}H_{27}N_2OS$ requires 391.1844

4-(6-Methoxy-5-(piperidin-1-yl)naphthalen-2-yl)butan-2-one (3aq) and 4-(6-methoxy-7-(piperidin-1-yl)naphthalen-2-yl)butan-2-one (3aq')

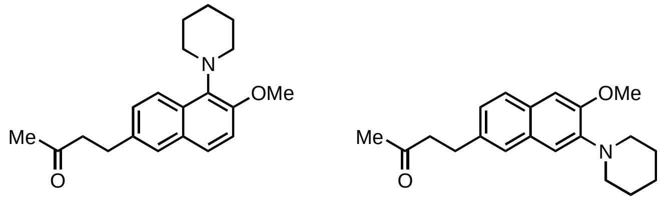

Following **GP4** but using 1.0 equiv. of the aromatic, **1a** (28.1 mg, 0.1 mmol) gave **3aq** and **3aq'** (26 mg, 82%) as an inseparable mixture, as an oil. **3aq**:**3aq'** = 4:1. R_f 0.24 [petrol–EtOAc (98:2)]; FT-IR ν_{max} (film)/cm^{-1} 2944, 2855, 1641, 1493, 1456, 1345, 1276; ^{1}H NMR (400 MHz, $CDCl_3$, isomer) δ 8.36 (0.8H, d, *J* = 8.7 Hz), 7.70–7.62 (0.4H, m), 7.56 (0.8H, d, *J* = 9.0 Hz), 7.53 (0.8H, s), 7.42–7.36 (0.2H, m) 7.31 (0.8H, d, *J* = 8.7 Hz), 7.22 (0.8H, d, *J* = 9.0 Hz), 7.16–7.11 (0.4H, m), 3.95 (0.6H, s), 3.92 (2.4H, s), 3.33 (1.6H, t, *J* = 10.9 Hz), 3.12–2.93 (4H, m), 2.88–2.79 (2.4H, m), 2.16 (3H, s), 1.94–1.63 (5.2, m), 1.53–1.36 (0.8H, m); ^{13}C NMR (101 MHz, $CDCl_3$, isomers) δ 208.3^m, 208.2^M, 157.2^m, 154.3^M, 136.4^M, 136.2^m, 135.7^M, 132.0^M, 130.3^m, 130.1^M, 129.5^m, 129.0^m, 127.6^m, 127.2^M, 127.1^m, 126.4^m, 126.2^M, 125.5^M, 124.8^m, 124.4^M, 121.4^m, 118.9^m, 118.4^m, 118.0^m, 116.4^m, 115.9^M, 106.4^m, 105.7^m, 102.4^m, 56.9^M, 55.4^m, 55.3^m, 54.5^m, 52.2^M, 45.4^m, 45.3^M, 33.8^m, 32.1^m, 30.3^m, 30.2^M, 30.15^m, 29.8^m, 29.75^M, 29.5^m, 27.4^M, 26.8^m, 24.8^M; HRMS (ESI): Found MH^+ 312.1955 $C_{20}H_{26}NO_2$ requires 312.1958.

Gram-scale Reaction: Following **GP4** but using 1.0 equiv. of the aromatic, **1a** (1.17 g, 4.38 mmol) gave **3aq** and **3aq'** (0.93 g, 68%) as an inseparable mixture, as an oil. **3aq**:**3aq'** = 4:1.

Methyl 2-(1-(4-Chlorobenzoyl)-5-methoxy-2-methyl-4-(piperidin-1-yl)-1H-indol-3-yl)acetate (3ar)

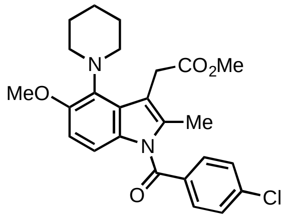

Following **GP4** but using 1.0 equiv. of the aromatic, **1a** (27 mg, 0.1 mmol) gave **3ar** (28 mg, 61%) as an oil. R_f 0.24 [petrol–EtOAc (98:2)]; FT-IR ν_{max} (film)/cm^{-1} 2931, 2849, 1734, 1683, 1591, 1484, 1432, 1349, 1248, 1124, 1015; ^{1}H NMR (400 MHz,

$CDCl_3$) δ 7.66 (2H, d, *J* = 8.5 Hz), 7.46 (2H, d, *J* = 8.4 Hz), 6.86 (1H, d, *J* = 8.9 Hz), 6.66 (1H, d, *J* = 9.0 Hz), 4.19 (2H, s), 3.80 (3H, s), 3.70 (3H, s), 3.41–3.23 (2H, m), 2.83 (2H, br d, *J* = 11.2 Hz), 2.24 (3H, s), 1.78 (1H, br d, *J* = 13.1 Hz), 1.65–1.59 (4H, m), 1.38–1.27 (1H, m); ^{13}C NMR (101 MHz, $CDCl_3$) δ 172.5, 168.5, 155.4, 139.2, 135.7, 134.4, 134.0, 132.3, 131.3 (x 2), 129.2 (x 2), 128.5, 113.7, 112.0, 109.6, 56.3, 51.8, 51.7 (x 2), 30.9 (x 2), 26.3 (x 2), 24.5; HRMS (ASAP POS): Found MH^+ 455.1719 $C_{25}H_{28}N2O_4Cl$ requires 455.1732.

Methyl 5-(2,5-Dimethyl-4-(piperidin-1-yl)phenoxy)-2,2-dimethylpentanoate (3as)

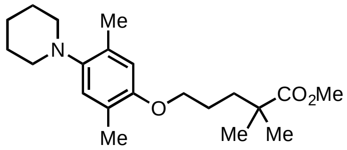

Following **GP4** but using 1.0 equiv. of the aromatic, **1a** (27 mg, 0.1 mmol) gave **3as** (15 mg, 43%) as an oil. R_f 0.24 [petrol–EtOAc (98:2)]; FT-IR ν_{max} (film)/cm^{-1}2949, 1731, 1683, 1507, 1457, 1200, 1146; ^{1}H NMR (400 MHz, $CDCl_3$) δ 6.81 (1H, s), 6.62 (1H, s), 3.90–3.85 (2H, m), 3.66 (3H, s), 2.76 (4H, br t, *J* = 5.2 Hz), 2.25 (3H, s), 2.17 (3H, s), 1.74–1.60 (8H, m), 1.59–1.50 (2H, m), 1.21 (6H, s); ^{13}C NMR (126 MHz, $CDCl_3$) δ 178.5, 152.8, 145.9, 131.1, 124.3, 121.9, 114.1, 68.5, 54.1 (x 2), 51.9, 42.3, 37.3, 31.1 (x 2), 26.9 (x 2), 25.3, 24.6, 17.7, 16.1; HRMS (ASAP POS): Found MH^+ 348.2521 $C_{21}H_{34}NO_3$ requires 348.2533.

(4b*S*,8a*S*,9*S*)-3-Methoxy-11-methyl-2-(piperidin-1-yl)-6,7,8,8a,9,10-hexahydro-5*H*-9,4b-(epiminoethano)phenanthrene (3at)

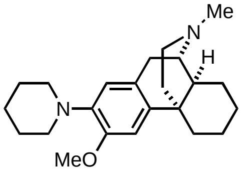

Following **GP4** but using 1.0 equiv. of dextromethorphan and 4.0 equiv. of $HClO_4$, **1a** (27 mg, 0.1 mmol) gave **3at** (16 mg, 44%) as an oil. R_f 0.57 [CH_2Cl_2–MeOH (90:10)]; FT-IR ν_{max} (film)/cm^{-1} 2928, 2854, 1506, 1463, 1275, 1238, 1226, 1117, 1034; ^{1}H NMR (500 MHz, $CDCl_3$) δ 6.67 (1H, s), 6.63 (1H, s), 3.82 (3H, s), 3.42 (1H, br s), 3.16–3.04 (2H, m), 3.03–2.88 (5H, m), 2.79 (3H, s), 2.35 (1H, d, *J* = 14.5 Hz), 2.22 (1H, t, *J* = 7.6 Hz), 2.00 (1H, q, *J* = 6.5 Hz), 1.75 (5H, p, *J* = 5.6 Hz), 1.69–1.61 (1H, m), 1.61–1.53 (4H, m), 1.52–1.38 (6H, m); 13C NMR (151 MHz, $CDCl_3$) δ

152.28, 141.7, 131.2, 125.0, 117.5, 108.0, 60.4, 55.7, 52.4 (x 2), 48.3, 41.8, 41.2, 39.1, 35.7, 35.3, 27.4, 27.3, 26.4 (x 2), 25.9, 25.7, 24.5, 22.8; HRMS (ASAP POS): Found MH$^+$ 355.2751 $C_{23}H_{35}N_2O$ requires 355.2749.

(4a*R*,4a^1*R*,5a*S*,8a*R*,8a^1*S*,15a*S*)-10-(piperidin-1-yl)-2,4a,4a^1,5,5a,7,8,8a^1,15,15a-decahydro-14*H*-4,6-methanoindolo[3,2,1-*ij*]oxepino[2,3,4-*de*]pyrrolo[2,3-*h*]quinolin-14-one (3au)

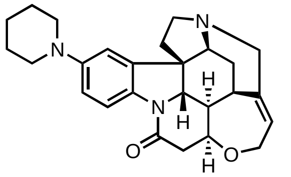

Following **GP4** but using 0.5 equiv. of strychnine and 3.0 equiv. of $HClO_4$, **1a** (33 mg, 0.1 mmol) gave **3au** (18 mg, 42%) as an oil. R_f 0.24 [CH_2Cl_2–MeOH (90:10)]; FT-IR ν_{max} (film)/cm^{-1} 2924, 2852, 1661, 1490, 1379, 1254, 1222; ^{1}H NMR (500 MHz, $CDCl_3$) δ 7.95 (1H, d, *J* = 8.7 Hz), 6.83 (1H, dd, *J* = 8.7, 2.4 Hz), 6.78 (1H, d, *J* = 2.4 Hz), 5.92 (1H, t, *J* = 7.1 Hz), 4.27 (1H, dt, *J* = 8.5, 3.3 Hz), 4.14 (1H, dd, *J* = 13.8, 7.0 Hz), 4.05 (1H, dd, *J* = 13.8, 6.0 Hz), 3.95 (1H, br s), 3.83 (1H, d, *J* = 10.5 Hz), 3.73 (1H, d, *J* = 14.7 Hz), 3.27–3.20 (1H, m), 3.14 (1H, br s), 3.15–3.02 (5H, m), 2.87 (1H, ddd, *J* = 12.1, 10.2, 6.9 Hz), 2.75 (1H, d, *J* = 14.8 Hz), 2.64 (1H, dd, *J* = 17.3, 3.3 Hz), 2.34 (1H, dt, *J* = 14.5, 4.4 Hz), 1.93–1.87 (2H, m), 1.76–1.67 (4H, m), 1.60–1.51 (2H, m), 1.48 (1H, d, *J* = 14.5 Hz), 1.28–1.26 (1H, d, *J* = 7.0 Hz); ^{13}C NMR (126 MHz, $CDCl_3$) δ 168.7, 150.2, 140.1, 135.4, 133.5, 127.9, 117.0, 116.8, 111.5, 78.0, 64.7, 60.4, 60.3, 52.8, 52.3, 51.9 (x 2), 50.5, 48.3, 42.8, 42.5, 31.7, 26.9, 26.2 (x 2), 24.3; HRMS (APCI): Found MH$^+$ 418.2471 $C_{26}H_{32}O_2N_3$ requires 418.2489.

(*E*)-1-(2-(phenyldiazenyl)phenyl)piperidine (3av), (*E*)-1-(4(Phenyldiazenyl) phenyl)piperidine (3av')

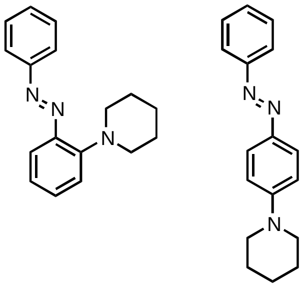

Following **GP4**, **1a** (27 mg, 0.1 mmol) gave **3av** (8 mg, 32%) as an oil and **3av'** (6 mg, 26%) as wax. **3av**:**3av'** = 1.3:1.

Data for **3av**. R_f 0.48 [petrol–EtOAc (98:2)]; FT-IR ν_{max} (film)/cm^{-1} 2972, 1555, 1223, 1110, 1022; ^{1}H NMR (500 MHz, $CDCl_3$) δ 7.98 – 7.92 (2H, m), 7.62 (1H, dd, *J* = 8.0, 1.6 Hz), 7.52 (2H, t, *J* = 7.6 Hz), 7.45 (1H, t, *J* = 7.3 Hz), 7.37 (1H, ddd, *J* = 8.5, 7.1, 1.6 Hz), 7.10 (1H, dd, *J* = 8.2, 0.9 Hz), 6.99 (1H, ddd, *J* = 8.1, 7.1, 1.2 Hz), 3.22 – 3.17 (4H, m), 1.85 – 1.78 (4H, m), 1.67 – 1.60 (2H, m); ^{13}C NMR (126 MHz, $CDCl_3$) δ 153.2, 151.6, 144.7, 132.0, 130.7, 129.2 (x 2), 123.1 (x 2), 121.4, 118.9, 117.1, 54.8 (x 2), 26.6 (x 2), 24.5. HRMS (APCI): Found MH^+ 266.1663 $C_{17}H_{20}N_3$ requires 266.1652

Data for **3av'**. R_f 0.40 [petrol–EtOAc (98:2)]; FT-IR ν_{max} (film)/cm^{-1} 2962, 1554, 1258, 1084, 1015; ^{1}H NMR (500 MHz, $CDCl_3$) δ 7.85 (4H, app t, *J* = 9.2 Hz), 7.48 (2H, t, *J* = 7.6 Hz), 7.42–7.36 (1H, m), 6.97 (2H, d, *J* = 8.5 Hz), 3.37 (4H, t, *J* = 5.3 Hz), 1.71 (4H, d, *J* = 5.5 Hz, 1H), 1.67–1.63 (2H, m); ^{13}C NMR (126 MHz, $CDCl_3$) δ 153.7, 153.3, 144.88, 129.8, 129.1 (x 2), 124.9 (x 2), 122.4 (x 2), 114.7 (x 2), 49.3 (x 2), 25.7 (x 2), 24.5. HRMS (APCI): Found MH^+ 266.1651 $C_{17}H_{20}N_3$ requires 266.1652.

***N*-methyl-*N*-(3-phenyl-3-(4-(trifluoromethyl)phenoxy)propyl)naphthalen-1-amine (3aw)**

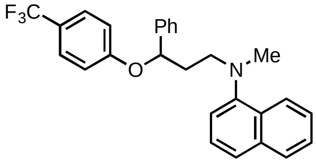

Following **GP4**, **1l** (49.1 mg, 0.1 mmol) gave **3aw** (25 mg, 58%) as an oil. R_f 0.44 [petrol–EtOAc (98:2)]; FT-IR ν_{max} (film)/cm^{-1} 2966, 2858, 1599, 1465, 1442, 1387,

1255; ^{1}H NMR (500 MHz, $CDCl_3$) δ 8.19 (1H, d, *J* = 8.4 Hz), 7.81 (1H, d, *J* = 8.1 Hz), 7.55 (1H, d, *J* = 8.1 Hz), 7.45 (1H, t, *J* = 7.5 Hz), 7.41–7.34 (4H, m), 7.31–7.22 (5H, m), 7.12 (1H, d, *J* = 7.4 Hz), 6.79 (2H, d, *J* = 8.4 Hz), 5.30 (1H, dd, *J* = 8.5, 4.6 Hz), 3.37 (1H, dt, *J* = 13.9, 7.2 Hz), 3.24 (1H, ddd, *J* = 13.0, 7.4, 5.6 Hz), 2.87 (3H, s), 2.38–2.22 (1H, m), 2.19–2.07 (1H, m); ^{13}C NMR (126 MHz, $CDCl_3$, one C missing) δ 160.7, 149.7, 141.2, 135.0, 129.9, 128.9 (x 2), 128.47, 127.9, 126.8 (q, *J* = 3.8 Hz, x 2), 125.9, 125.9 (x 2), 125.8, 125.5, 123.9, 123.7, 123.6–122.2 (m), 116.3, 115.8 (x 2), 78.3, 52.5, 43.6, 36.7; ^{19}F NMR (376 MHz, $CDCl_3$) δ–62.3; HRMS (APCI): Found MH^+ 436.1889 $C_{27}H_{25}NOF_3$ requires 436.1888.

5,6-Dimethoxy-2-((1-(naphthalen-1-yl)piperidin-4-yl)methyl)-2,3-dihydro-1H-inden-1-one (3ax)

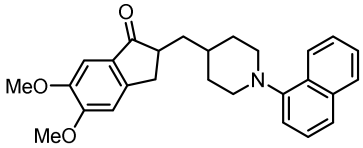

Following **GP4**, **1m** (49 mg, 0.1 mmol) gave **3ax** (25 mg, 61%) as an oil. R_f 0.54 [petrol–EtOAc (50:50)]; FT-IR ν_{max} (film)/cm^{-1} 2922, 2847, 1693, 1591, 1499, 1461, 1311, 1263, 1124, 1037; ^{1}H NMR (400 MHz, $CDCl_3$) δ 8.18 (1H, d, *J* = 7.3 Hz), 7.87–7.77 (1H, m), 7.52 (1H, d, *J* = 8.2 Hz), 7.47 (2H, tt, *J* = 6.9, 5.1 Hz), 7.39 (1H, t, *J* = 7.8 Hz), 7.21 (1H, s), 7.12–7.04 (1H, m), 6.89 (1H, s), 3.98 (3H, s), 3.92 (3H, s), 3.53–3.39 (2H, m), 3.37 – 3.25 (1H, m), 2.88–2.69 (4H, m), 2.13–2.01 (1H, m), 1.95 (1H, d, *J* = 12.0 Hz), 1.92–1.84 (1H, m), 1.77–1.57 (4H, m), 1.53–1.41 (1H, m); ^{13}C NMR (101 MHz, $CDCl_3$) δ 207.9, 155.6, 150.7, 149.6, 148.9, 134.9, 129.5, 129.2, 128.4, 126.0, 125.9, 125.4, 123.9, 123.2, 114.6, 107.5, 104.6, 56.4, 56.3, 54.0 (x 2), 45.6, 39.0, 34.8, 33.9, 33.5, 32.7; HRMS (ASAP POS): Found MH^+ 416.2220 $C_{27}H_{30}NO_3$ requires 416.2220.

4-(6-Methoxy-5-(methyl(3-phenyl-3-(4-(trifluoromethyl)phenoxy)propyl)amino) naphthalen-2-yl)butan-2-one (3ay)

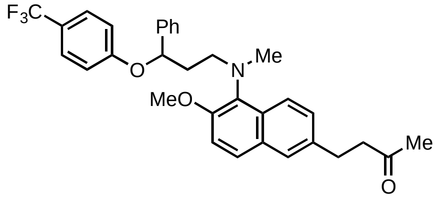

Following **GP4** but using 1.0 equiv. of the aromatic, **1l** (49 mg, 0.1 mmol) gave **3ay** (21 mg, 39%) as an oil. R_f 0.44 [petrol–EtOAc (98:2)]; FT-IR ν_{max} (film)/cm^{-1} 2922, 2859, 1716, 1653, 1594, 1516, 1456, 1324, 1243, 1160, 1111, 1067; ^{1}H NMR (500 MHz, $CDCl_3$) δ 8.27 (1H, br d, *J* = 10.5 Hz), 7.71–7.30 (7H, m), 7.23–6.49 (6H, m), 5.34 (1H, d, *J* = 8.7 Hz), 3.88 (3H, s), 3.56–3.17 (2H, m), 3.09–2.93 (2H, m), 2.85 (3H, s), 2.79 (2H, br s), 2.15 (3H, s), 2.11–1.76 (2H, m); ^{13}C NMR (151 MHz, $CDCl_3$) δ 208.3, 160.9, 154.8, 141.8, 136.3, 133.9–132.2 (x 2, m), 129.9, 129.5, 128.8 (x 2), 128.1–127.2 (x 2, m), 126.6 (x 2), 126.4, 126.3, 126.2–125.6 (x 2, m), 124.2 (d, *J* = 31.6 Hz), 116.1, 115.8 (x 2), 114.9, 78.3, 56.2, 51.5, 45.3, 42.30 (d, *J* = 105.0 Hz), 38.1 (d, *J* = 63.4 Hz), 30.3, 29.7; ^{19}F NMR (376 MHz, $CDCl_3$) δ –61.7 (m); HRMS (ASAP POS): Found MH^+ 536.2409 $C_{32}H_{33}NO_3F_3$ requires 536.2413.

Methyl 2-(1-(4-Chlorobenzoyl)-4-(4-((5,6-dimethoxy-1-oxo-2,3-dihydro-1*H*-inden-2-yl)methyl)piperidin-1-yl)-5-methoxy-2-methyl-1*H*-indol-3-yl)acetate (3az)

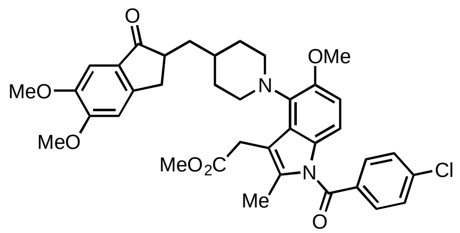

Following **GP4** but using 1.0 equiv. of the aromatic, **1m** (47 mg, 0.1 mmol) gave **3az** (37 mg, 56%) as an oil. R_f 0.35 [petrol–EtOAc (9:1)]; FT-IR ν_{max} (film)/cm^{-1} 2922, 2848, 1739, 1690, 1591, 1500, 1433, 1350, 1311, 1263, 1166, 1087; ^{1}H NMR (500 MHz, $CDCl_3$) δ 7.67 (2H, d, *J* = 8.4 Hz), 7.46 (2H, d, *J* = 8.5 Hz), 7.20 (1H, s), 6.91–6.86 (2H, m), 6.67 (1H, d, *J* = 9.0 Hz), 4.18 (1H, d, *J* = 17.4 Hz), 4.12 (1H, d, *J* = 17.4 Hz), 3.98 (3H, s), 3.92 (3H, s), 3.82 (3H, s), 3.72 (3H, s), 3.46–3.35 (2H, m), 3.33–3.23 (m, 1H), 2.90 (2H, t, *J* = 13.0 Hz), 2.81–2.72 (2H, m), 2.24 (3H, s), 2.01–

1.92 (1H, m), 1.77 (1H, d, *J* = 13.0 Hz), 1.73–1.59 (1H, m), 1.45–1.33 (3H, m); ^{13}C NMR (126 MHz, $CDCl_3$) δ 208.2, 172.5, 168.5, 155.6, 155.4, 149.6, 149.0, 139.2, 135.9, 134.4, 133.4, 132.3, 131.3 (x 2), 129.5, 129.2 (x 2), 128.6, 113.6, 112.1, 109.5, 107.5, 104.6, 56.4, 56.3 (x 2), 51.9, 51.2, 51.1, 45.6, 39.5, 34.6, 33.6, 33.5, 32.2, 30.8, 13.4; HRMS (ASAP POS): Found MH^+ 659.2519 $C_{37}H_{40}N2O_7Cl$ requires 659.2519.

4 Mechanistic Considerations

Several productive mechanisms can be operating under our reaction conditions. All possible mechanism, using *t*-Bu-benzene as the coupling partner, are illustrated in **Scheme S1**.

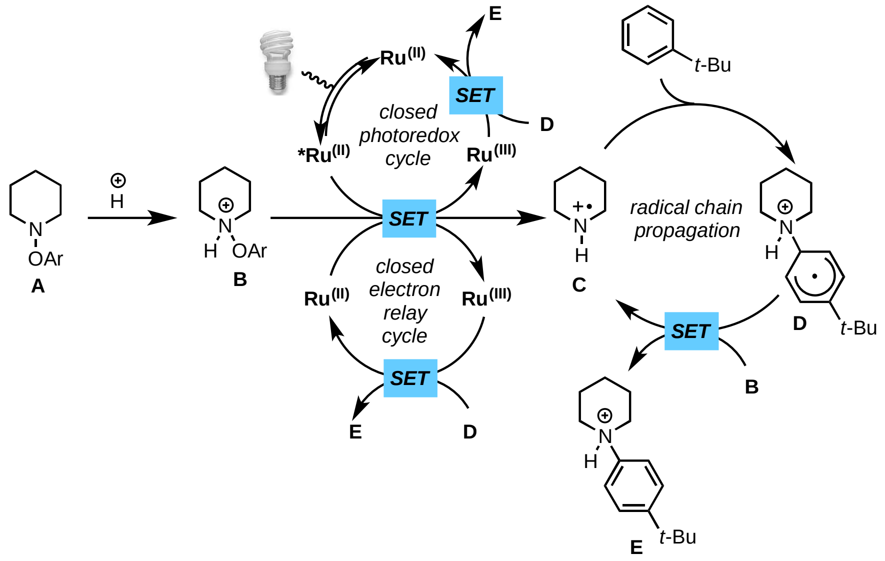

Scheme S1.

4.1 Mechanism Based on the Closed Photoredox Cycle

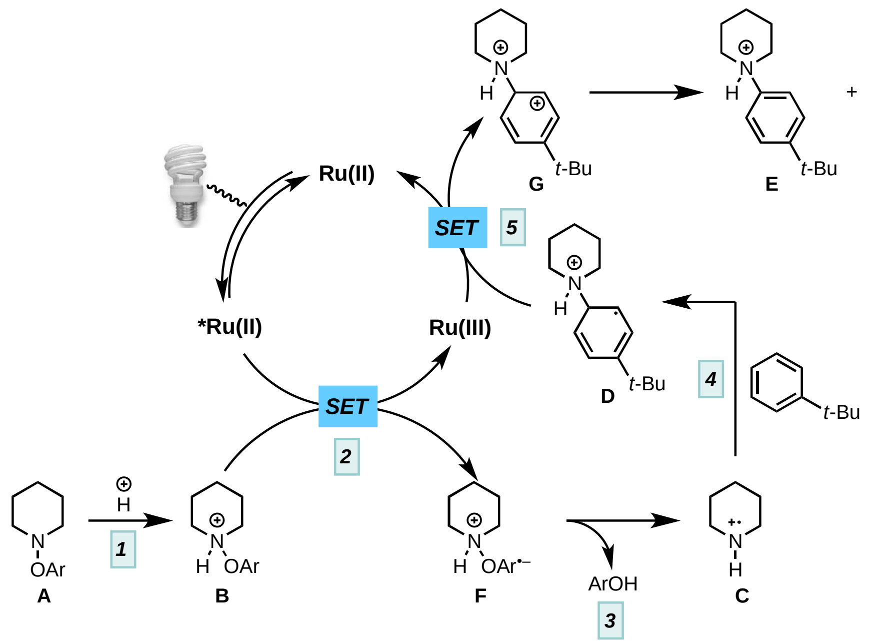

Scheme S2.

STEP 1: This step is supported by the NMR studies on the protonation of **1a** (see Section 4.6). According to these experiments, **1a** is a very weak base and requires a strong Brönsted acid in order to undergo protonation.

STEP 2: This step is supported by the electrochemical and the luminescence quenching studies (see Sections 4.7 and 4.8). **1a** has a reduction potential E^{red} = –0.90 (vs SCE in CH_3CN) and quenches $*Ru(bpy)_3^{2+}$ with $k_q = 2.1 \times 10^8$ M^{-1} s^{-1}.

Upon protonation, **1a** is expected to undergo very favourable SET from $*Ru(bpy)_3^{2+}$.

The addition of strong Brönsted acids, like $HClO_4$, has been known to increase dramatically the reduction potential of organic molecules and to accelerate the SET reduction from $*Ru(bpy)_3^{2+}$.[16] In some cases, the reduction potential can even reach positive values (Scheme S3).

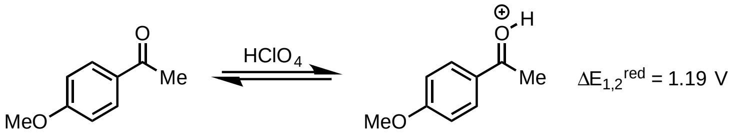

Scheme S3.

We have tried to determine the $E_{1/2}^{red}$ of **1a** but upon addition of $HClO_4$ (70% in H_2O) a very complex cyclic voltammogram was obtained which did not provide any conclusive data. As a result, we have evaluated the variation of **1a**'s reduction potential by DFT methods.[i] According to our calculations,[17] ΔE^{red} = 2.5 V which would make the SET reduction of **1a**–H^+ extremely exergonic (Scheme S4). According to this value, we believe **1a**–H^+ to be an extremely strong oxidant, especially if compared with other SET oxidants that contain a weak N–X bond like selectfluor (E^{red} = +0.25 V vs SCE).[18]

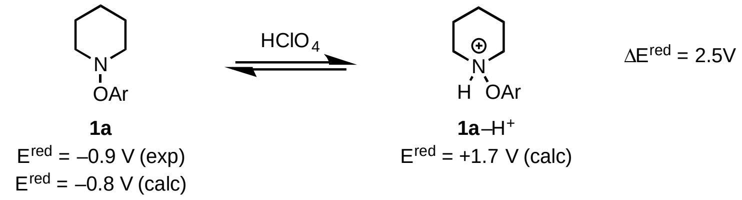

Scheme S4.

STEP 3: This step is supported by the quantitative isolation of the 2,4-$(NO_2)_2$-phenol at the end of our reactions.

STEP 4: We have studied the radical amination step by DFT and a discussion of our result is in Section 4.5.

STEP 5: We believe this SET to be exergonic but we do not have direct evidences.

---

[i] We have benchmarked our DFT studies by calculating a known $\Delta E_{1/2}^{red}$ for the protonation of *p*-OMe-acetophenone with $HClO_4$ (see also Scheme S3).

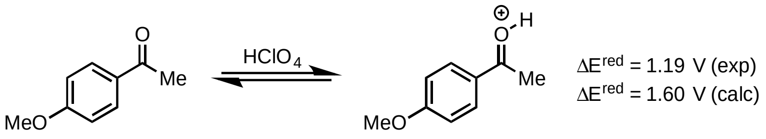

4.2 Mechanism Based on the Ru(II)-Catalysed Electron Relay

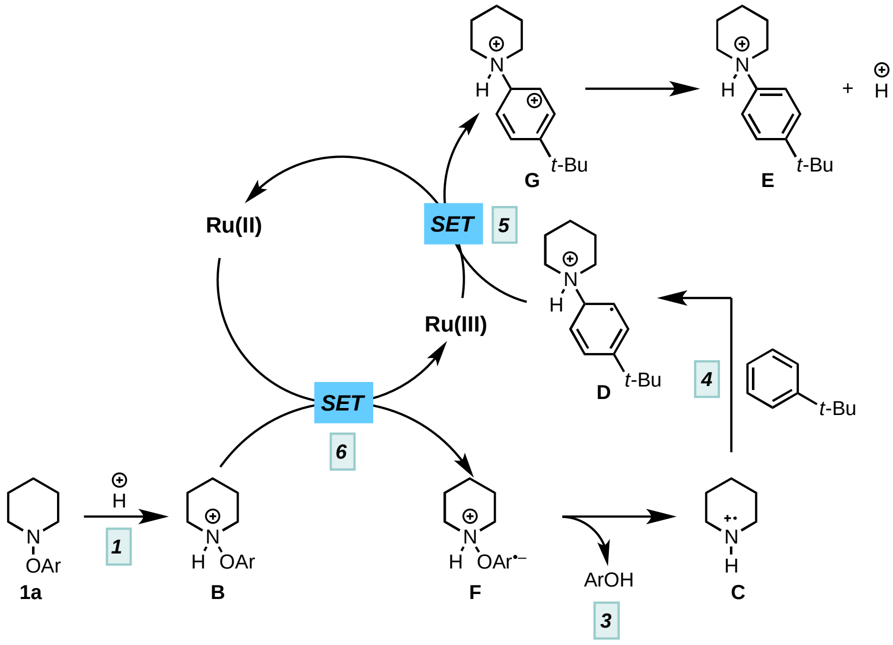

Scheme S5.

STEP 6: The possibility of Ru(II) to act as an electron relay catalyst is supported by the evidence that the reaction between **1a** and *t*-Bu-benzene leads to the formation of **3a** in the absence of light (se also Table S1). As shown in Scheme S6, in the absence of Ru(bpy)$_3$Cl$_2$ with or without blue LEDs irradiation, no product was formed (reactions 2 and 4). However, when the reaction was run with both Ru(bpy)$_3$Cl$_2$ and HClO$_4$ in the dark, **3a** was formed in 58% yield (reaction 3).

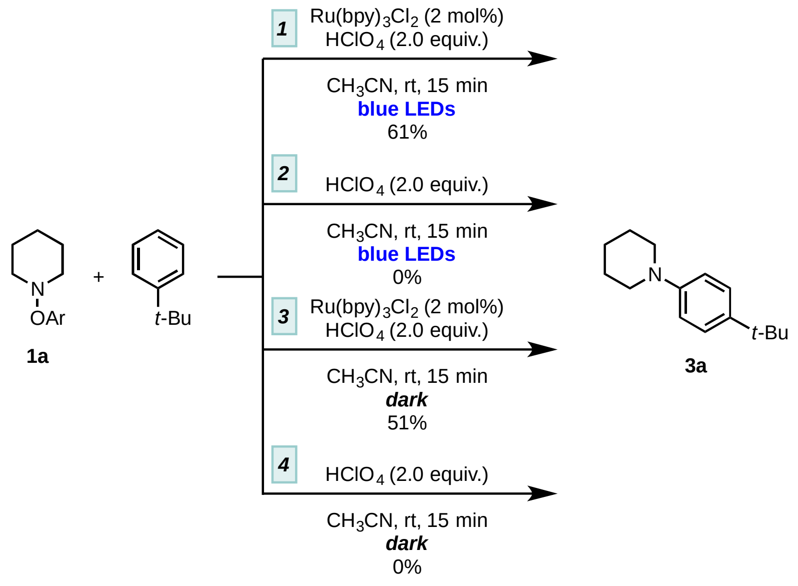

Scheme S6.

The $E_{1/2}^{ox}$ for Ru(II)→Ru(III) = 1.29 V (vs SCE in CH_3CN)[19] makes it a weak reductant but given the strong activation of **1a** upon protonation (see discussion regarding Scheme S4), we believe that this step can be feasible as our calculated E^{red} for **1a**–H^+ = +1.7 V. Furthermore, we have been able to detect the formation of $[Ru(bpy)_3]^{3+}$ under dark reaction conditions (see Section 4.2.1).

STEPs 3, 4 and 5 are identical to the ones in the **Photoredox Cycle** (see Section S4.1).

4.2.1 Detection of $[Ru(bpy)_3]^{3+}$

In order to get insights into the reactivity observed in the darkness, we studied the changes on the absorption profile of $[Ru(bpy)_3]Cl_2$ (5×10^{-5} M in MeCN) by UV/Vis spectroscopy upon sequential addition of **1a** (final concentration = 2.5×10^{-3} M, 50 equiv.) and $HClO_4$ (final concentration = 0.025 M). The ratio between the catalyst and the amine was chosen to mimic the reaction conditions, while the concentration of $HClO_4$ employed was enough to ensure the full protonation of the amine.

As described in Scheme S7 when a solution of $[Ru(bpy)_3]^{2+}$ was treated with the amine **1a**, no changes were observed and a superimposed spectrum resulted. However, upon addition of $HClO_4$ full consumption of the characteristic MLCT band (452 nm) of $[Ru(bpy)_3]^{2+}$ was observed. Moreover, the resultant spectrum shows bands at ~410 and 670 nm, matching with those previously reported for

$[Ru(bpy)_3]^{3+}$.[20] No changes were observed in the absorption profile of $[Ru(bpy)_3]Cl_2$ upon addition of $HClO_4$ in the absence of **1a**. These results highlight the strongly oxidant character of **1a** upon protonation, which is capable to oxidize of $[Ru(bpy)_3]^{2+}$ ($E_{1/2}^{ox}$ = 1.29 V vs SCE).[19]

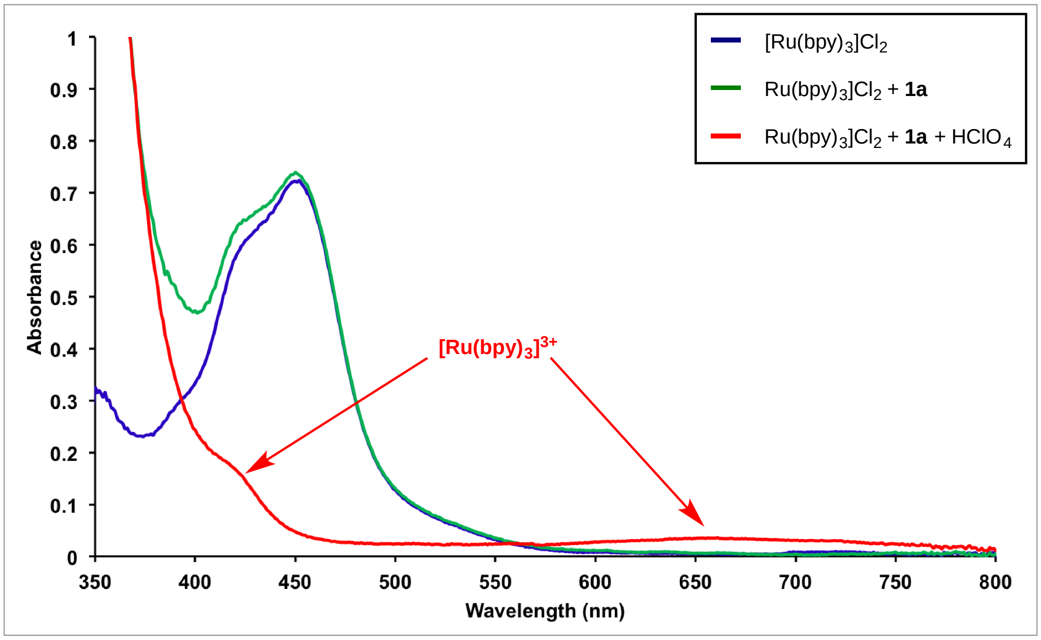

Scheme S7.

4.2.2 Further Studies to Support the Electron Relay Mode

As this reactivity was not envisaged we have performed further studies in order to evaluate its feasibility. As shown in Table S3 we have evaluated several other electron relay catalysts in the reaction between **1a** and *t*-Bu-benzene in the presence of $HClO_4$ in the dark. In general, **3a** was obtained in low conversion, which supports the feasibility of reactivity (entries 2–9). In the case of $Fe(bpy)_3Cl_2$, which has been reported in the literature to be a competent electron relay catalyst,[21] **3a** was obtained in 40% yield but no improvement was observed under blue LEDs irradiation (entries 10 and 11). Furthermore, in the absence of $HClO_4$ no product was observed which supports protonated **1a** to act as a strong oxidant (entry 12).

Table S3.

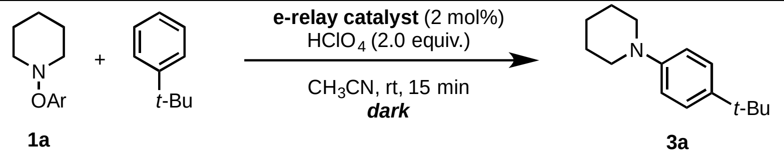

Entry	e-relay catalyst	Yield (%)
**1**	$Ru(bpy)_3Cl_2$	51
**2**	$AgBF_4$	traces
**3**	$Co(BF_4){\bullet}6H_2O$	traces
**4**	$[Cu(CH_3CN)_4](PF_6)$	3%
**5**	CuCl	traces
**6**	$FeCl_2$	4%
**7**	$MnCl_2$	10%
**8**	ferrocene	6%
**9**	$Fe(CO)_5$	traces
**10**	$[Fe(bpy)_3](PF_6)_2$	40%
**11**[a]	$[Fe(bpy)_3](PF_6)_2$	41%
**12**[b]	$[Fe(bpy)_3](PF_6)_2$	–

[a] The reaction was run under blue LEDs irradiation.

[b] The reaction was run without $HClO_4$.

This reactivity is however not general as, for example, reaction with 1-CN-naphtalene di not provide the desired product **3u** with or without blue LEDs irradiation (Scheme S8).

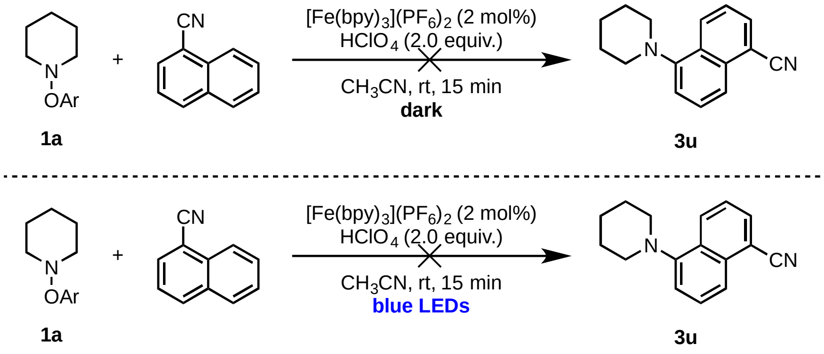

Scheme S8.

We have then evaluated this dark electron relay reactivity with $Ru(bpy)_3Cl_2$ in the presence of other aromatics and found that the yields were consistently lower when compared to the ones obtained upon blue LEDs irradiation (Table S4). In the case of cyano-naphtalene and strychnine (entries 3 and 4) the reactions were also run for 1h and overnight with no changes in the reaction yield.

Table S4.

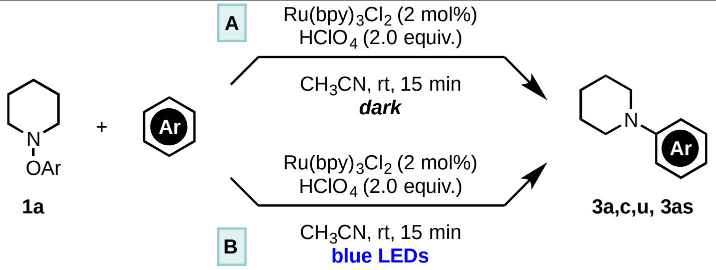

Entry	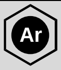	Yield (%) A	Yield (%) B
**1**	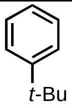	51	61
**2**	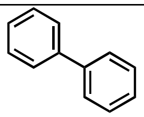	47	75
**3**	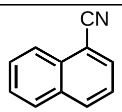	9	57
**4**	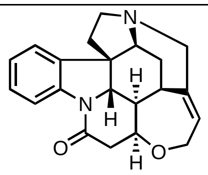	7	42

These study showed a significant difference in the reaction efficiency depending on blue LEDs irradiation. *As a result, we believe that the electron-relay mechanism might be involved as part of a chain-propagation process but it does not account alone for the full formation of the reaction products.*

4.3 Mechanism Based on the Radical Chain Propagation

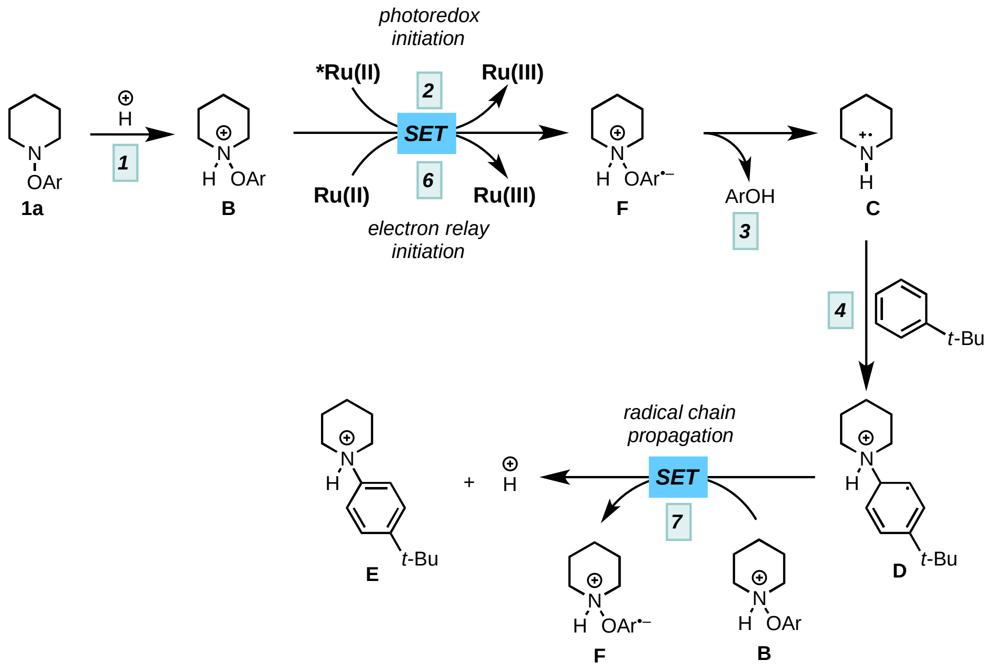

Scheme S9.

Steps 2, 3, 4 and 6 are identical to the ones in the **Photoredox catalysis** and **Ru(II)-Catalysed Electron Relay** (see above).

STEP 7. We have studied by DFT the feasibility of the chain-propagation and found that a SET between **B** and **D/D'** is thermodynamically feasible (Scheme S10). We believe that the strong ability of **B** to act as an oxidant is responsible for the success of this step.[ii]

[ii] We have calculated the quantum yield for the process to be Φ = 44.

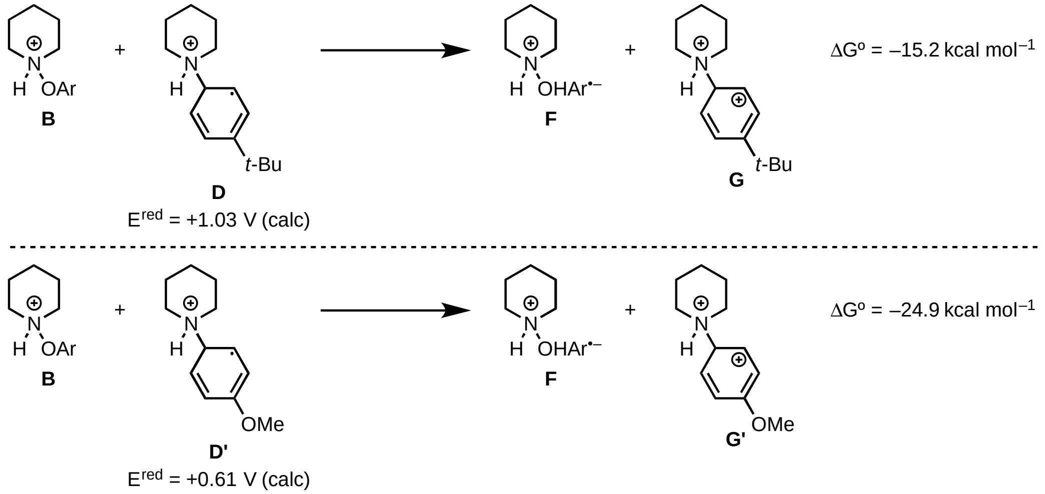
H OAr
B
+
H
t-Bu
D
E^red = +1.03 V (calc)
H OHAr•–
F
+
H
t-Bu
G
ΔGº = –15.2 kcal mol–1
H OAr
B
+
H
OMe
D'
E^red = +0.61 V (calc)
H OHAr•–
F
+
H
OMe
G'
ΔGº = –24.9 kcal mol–1

Scheme S10.

Conclusions

Overall, these mechanistic studies, revealed a complex interplay of three main mechanistic pathways all contributing to the success of the reaction. It is difficult at this stage to rule out any of these possible mechanisms as they all have supporting evidences. From these studies we also believe that, changing the nature of the aromatic coupling partner, can change the extent by which each mechanistic pathway contributes to the productive formation of the final product. This is visually represented in Scheme S11 with a generic benzene aromatic partner. The exact role of the blue LEDs irradiation in the improvement of the reaction performance depending on the aromatic partners remains unclear.

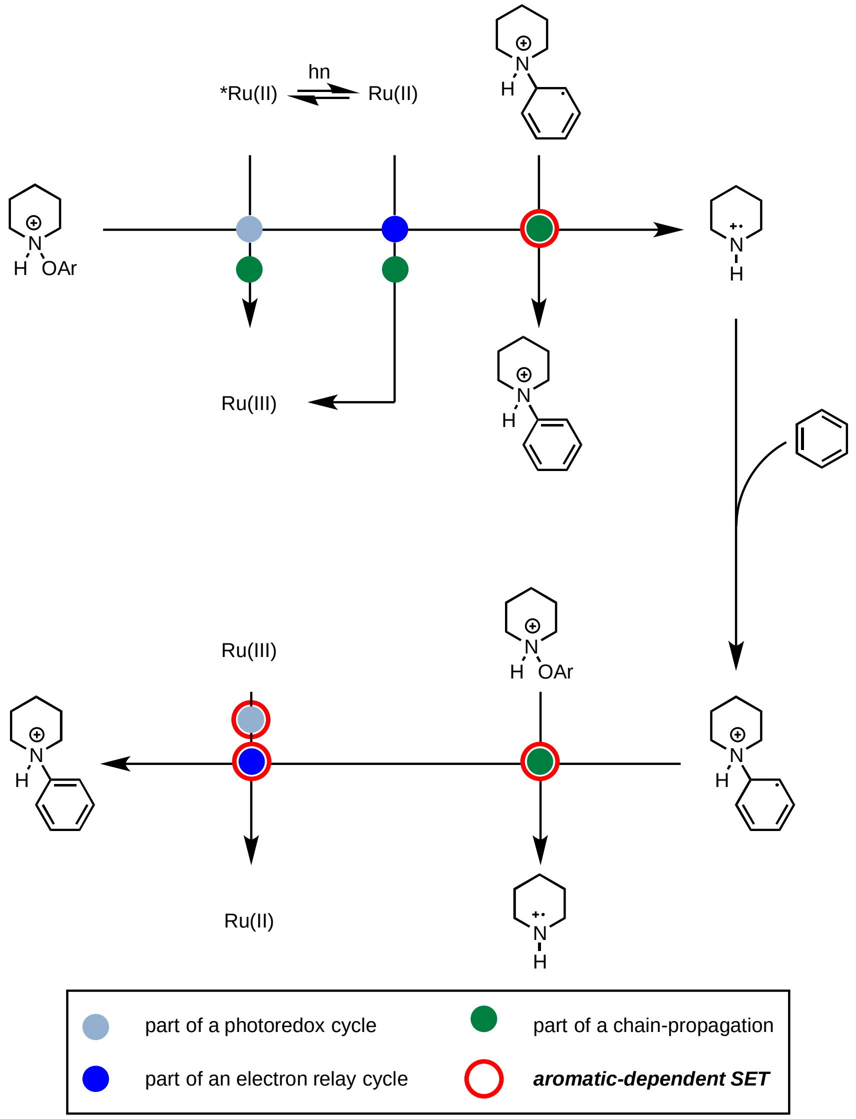

Scheme S11.

4.4 Stability of $Ru(bpy)_3Cl_2$ in the presence of $HClO_4$

The ability of $*Ru(bpy)_3Cl_2$ to perform reductive SET in the presence of large amounts of $HClO_4$ has been already demonstrated in the literature whereby the rate of SET increases linearly with the $HClO_4$ concentration.[16, 22] This means that decomposition pathways are not likely to happen. Nevertheless, we have evaluated $Ru(bpy)_3Cl_2$ stability by monitoring its luminescence profile upon addition of $HClO_4$. This study revealed no change in the emission profile of the catalyst (see Section 4.8).

4.5 Arylation of Aminium Radicals

In all the possible productive mechanisms, the formation of the reaction product relies on the ability of the aminium radical to undergo polarised radical reaction with the aromatic partner. We have performed DFT studies to understand better this step.

4.5.1 Electrophilicity of Aminium Radicals

Aminium radicals are isoelectronic to alkyl radicals but carry a formal positive charge, which makes them powerful electrophilic species. According to our calculations, upon protonation of the aminyl radical there is a remarkable increase in electrophilicity (Scheme S12). *This is in agreement with the radical reaction of aminium radicals with aromatics to be a highly polarized process.*

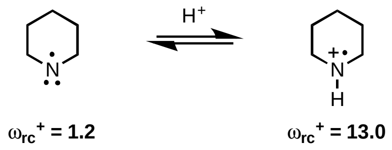

Scheme S12.

4.5.2 Reaction Selectivity – Fukui's Indices

In general our reactions lead to the formation of the *para*-aminated products as the major product. To rationalise this selectivity we have calculated the Fukui indices on several aromatics (Scheme S13). Our calculations show that the *para*-carbon is generally the most reactive and this is in accordance with our experimental results. *This is in agreement with the radical reaction of aminium radicals with aromatics to be a highly polarized process.*

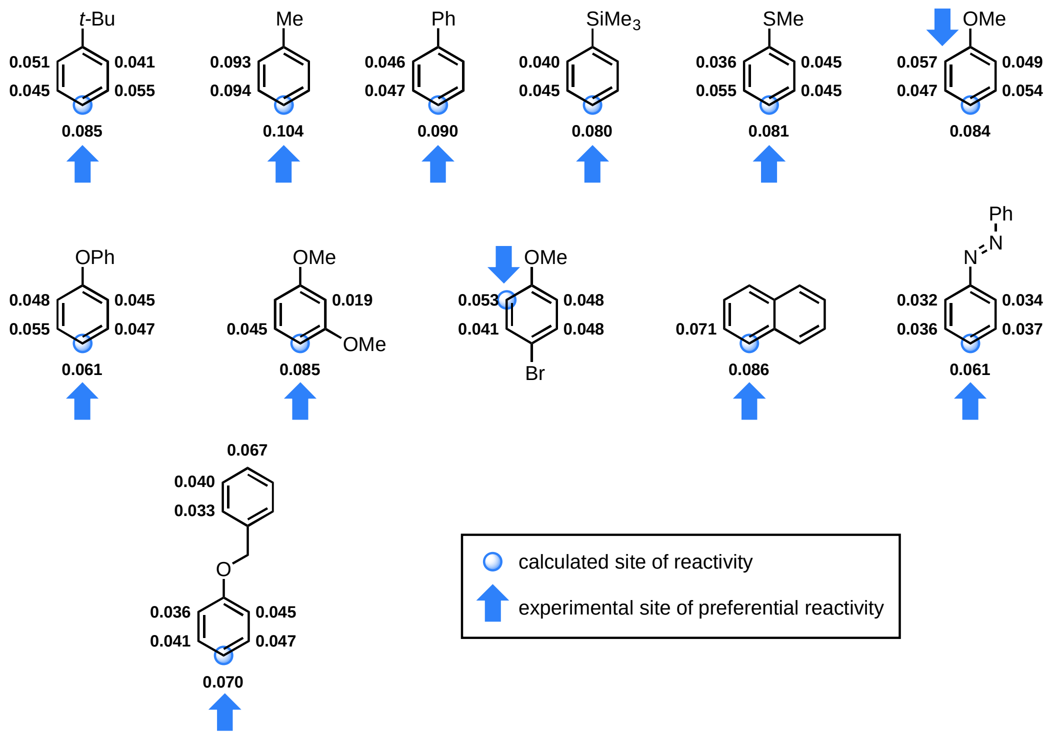

t-Bu
0.051
0.041
0.045
0.055
0.085
Me
0.093
0.094
0.104
Ph
0.046
0.047
0.090
SiMe3
0.040
0.045
0.080
SMe
0.036
0.045
0.055
0.045
0.081
OMe
0.057
0.049
0.047
0.054
0.084
OPh
0.048
0.045
0.055
0.047
0.061
OMe
0.019
0.045
OMe
0.085
OMe
0.053
0.048
0.041
0.048
Br
0.071
0.086
Ph
N
N
0.032
0.034
0.036
0.037
0.061
0.067
0.040
0.033
O
0.036
0.045
0.041
0.047
0.070
calculated site of reactivity
experimental site of preferential reactivity

Scheme S13.

4.6 Protonation Studies

In order to evaluate the ability of our nitrogen radical precursors to undergo protonation, we have performed ^{1}H NMR spectroscopy studies on the following aminyl radical precursors and Brönsted acids.

Aminyl radical precursors:

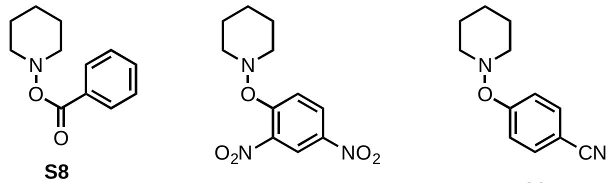

Brönsted acids:

**Brönsted acid**	AcOH	TFA	*p*TsOH	$HClO_4$
**p*K*a (H_2O)**	4.8	–0.25	–2.8	–10.0

General Procedure for the Protonation Studies – GP5

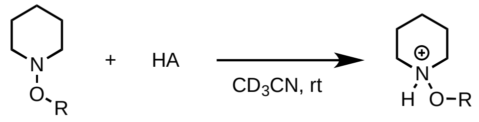

A dry NMR was charge with a solution of **1a**–**S8,9** (0.06 mmol, 1.0 equiv.) in CD_3CN (0.6 mL). The Brönsted acid (2.0 equiv.) was added, the NMR tube was shaken and the ^{1}H NMR spectrum was recorded.

The results of this study are collected in Table S6.

Table S6.

Substrate	Protonation (%)			
	Brönsted acid			
	AcOH	TFA	*p*TsOH	$HClO_4$
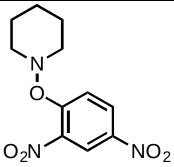 **1a**	–	–	20	100
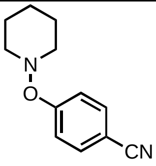 **S9**	–	10	100	100
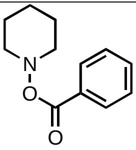 **S8**	–	100	100	100

4.7 Electrochemical Studies

4.7.1 General Experimental Details

Cyclic voltammetry was conducted on an EmStat (PalmSens) potentiostat using a 3-electrode cell configuration. A glassy carbon working electrode was employed alongside a platinum flag counter electrode and a silver pseudo-reference electrode. All the solutions were degassed by bubbling N_2 prior to measurements. 5 mM solutions of the desired compounds were freshly prepared in dry acetonitrile along with 0.1 M of tetrabutylammonium hexafluorophosphate as supporting electrolyte and were examined at a scan rate of 0.1 V s^{-1}. Ferrocene ($E_{1/2}$ = +0.42 V vs SCE)[17, 23] was added at the end of the measurements as an internal standard to determine the precise potential scale. Potential values are given versus the saturated calomel electrode (SCE). Irreversible reduction waves were obtained in all cases; therefore, the reduction potentials were estimated at half the maximum current, as previously described by Nicewicz.[17]

4.7.2 Electrochemical Potentials

Table S7.

Substrate	$E_{1/2}$ (V vs SCE)	Cyclic Voltammogram
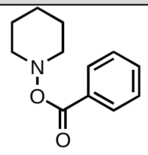 **S8**	–1.88	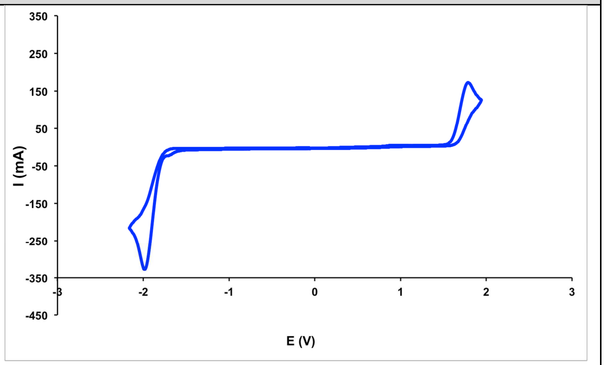
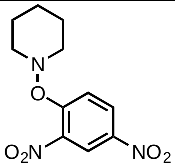 **1a**	–0.90	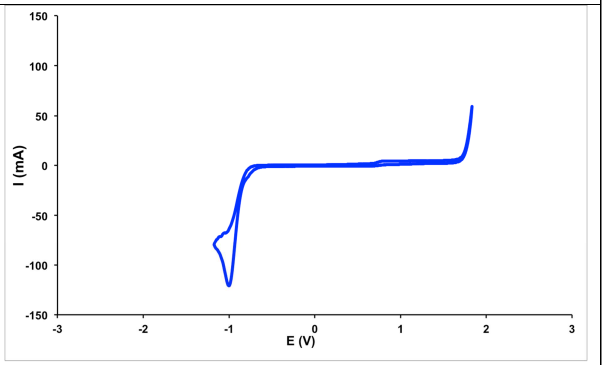
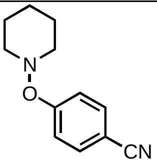 **S9**	–2.20	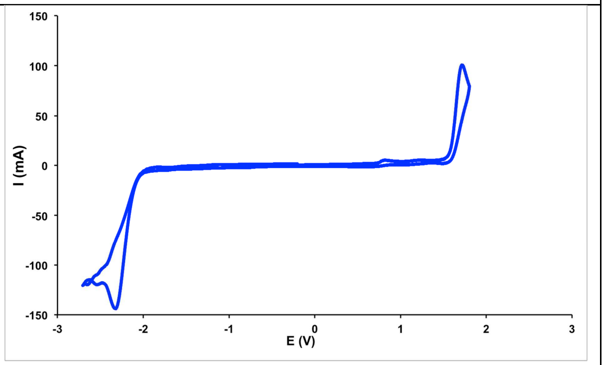
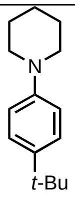 **3a**	+0.77	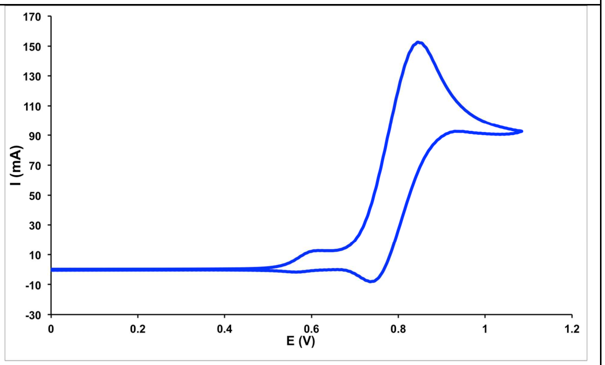

4.8 Emission Quenching Experiments

Stern-Volmer experiments for all the components of the reaction mixture were carried out monitoring the emission intensity of argon-degassed solutions of Ru(bpy)$_3$Cl$_2$ (1 x 10^{-5} M) containing variable amounts of the quencher in dry acetonitrile (Table S8 and Scheme S15). The reported excited-state lifetime for Ru(bpy)$_3$Cl$_2$ in MeCN (0.855 μs)[24] was used for k_q calculations (Table S9).

Table S8.

<table>
<tr><th>Substrate</th><th>Stern-Volmer Plot</th></tr>
<tr><td>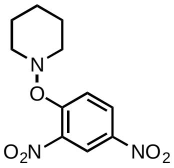

1a</td><td>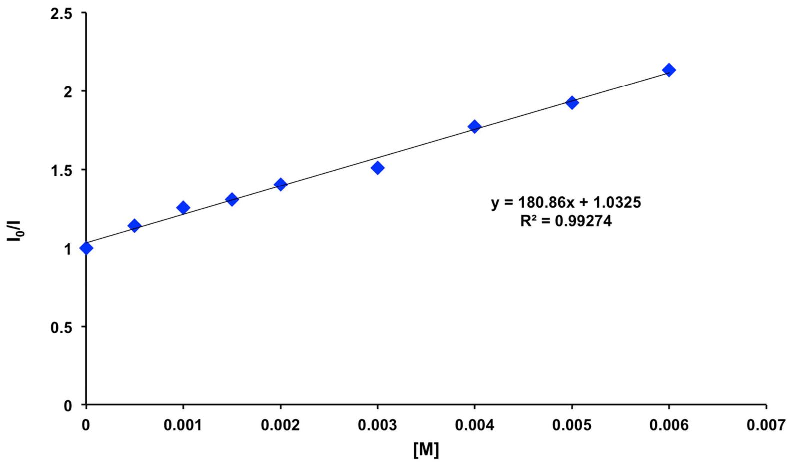
</td></tr>
<tr><td>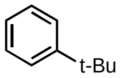
</td><td>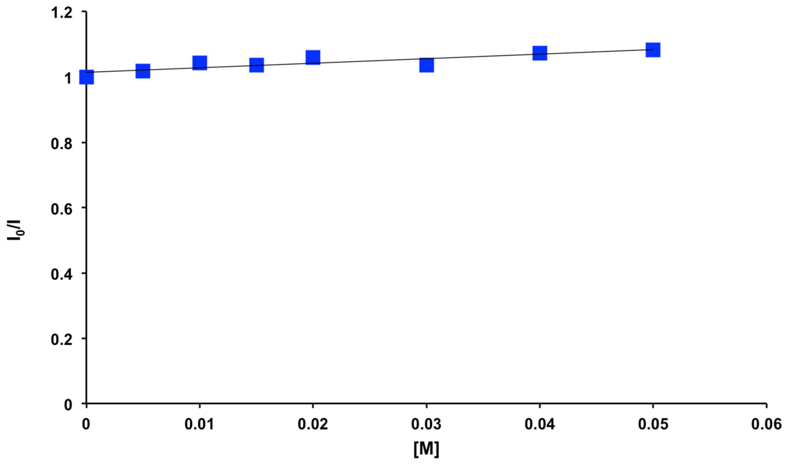
</td></tr>
</table>

<table>
<tr><th>Substrate</th><th>Stern-Volmer Plot</th></tr>
<tr><td>$HClO_4$</td><td>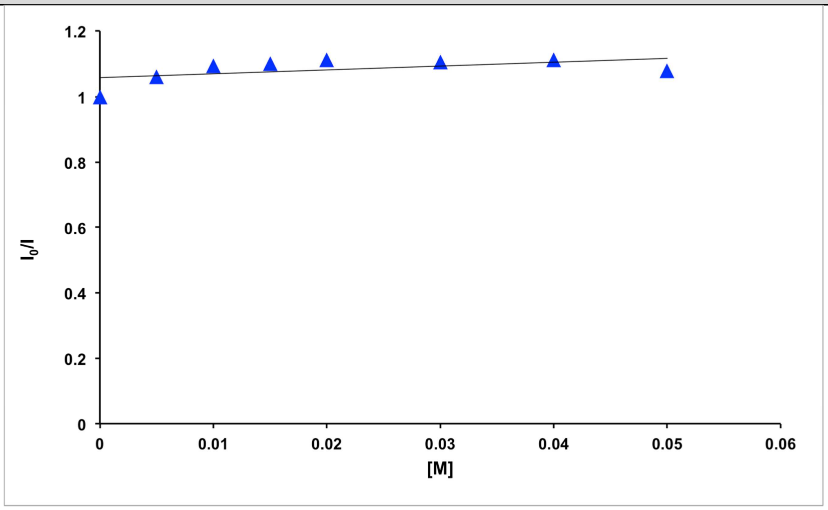
</td></tr>
<tr><td>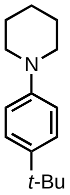

3a</td><td>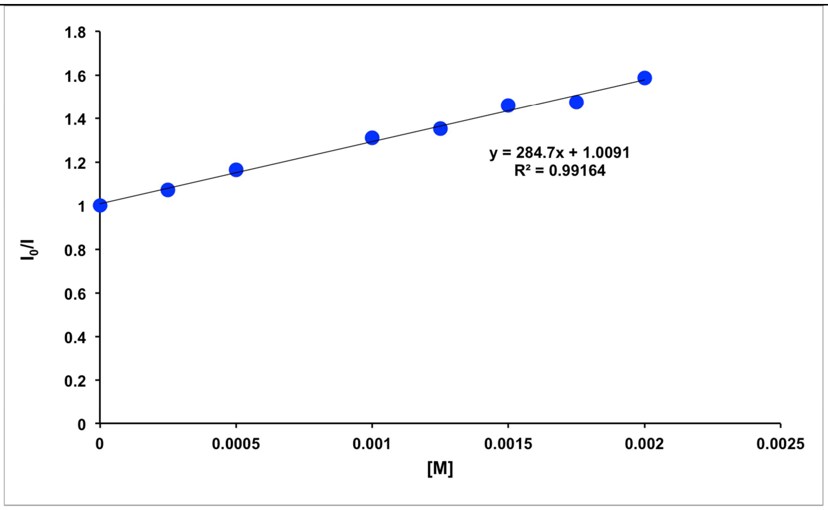
</td></tr>
</table>

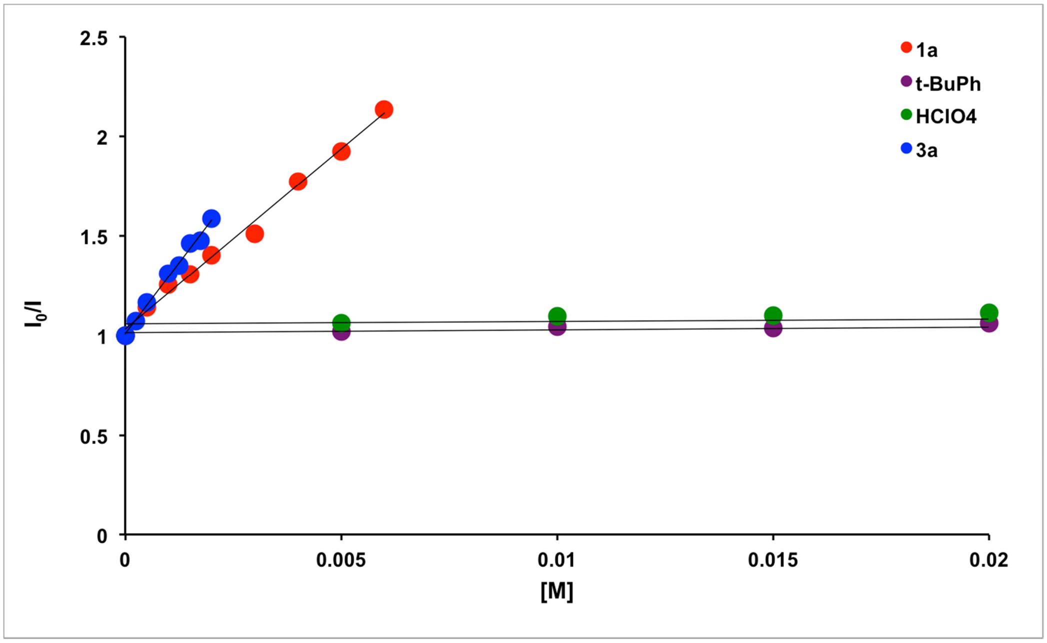

Scheme S15.

Table S9.

Substrate	k_q (M^{-1} s^{-1})	Substrate	k_q (M^{-1} s^{-1})
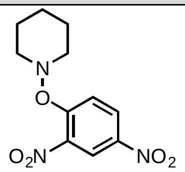 **1a**	2.1×10^8	$HClO_4$	–
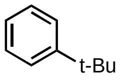	–	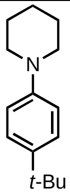 **3a**	3.3×10^8

5 Computational Data

5.1 Computational Studies

5.1.1 Computational methods

DFT calculations were carried out with the Gaussian 09 package at the B3LYP/6-311+g(d,p) level.[25] All geometry optimizations were followed by vibrational frequency calculations to verify that the obtained geometries were true minima on the potential energy surface. The solvent effects (when applicable) were computed by application of the SCRF model of solvation. The electronic properties such as adiabatic electron affinities and ionization potentials, electrophilicity index and reaction energies were calculated from the Gibbs free energies of relaxed molecules at the same level of theory including solvent effects (acetonitrile). Redox potentials (*vs* SCE) in acetonitrile were calculated according to the method described by Nicewicz.[17] Fukui indices[26] were calculated in the following way: The neutral arene (with N electrons) was subjected to a geometry optimization in the gas phase, and Hirshfeld populations were determined with Multiwfn 3.3.9.[27] Hirshfeld populations of the corresponding cationic (N–1 electrons) and anionic (N+1) arenes were calculated without further optimization. Fukui indices for radical attack were calculated for each atom as: $f_k^0 = [q_k(N-1) - q_k(N+1)]/2$.

5.2 Computational Data

5.2.1 Optimized structures for Fukui indices calculations.

B3LYP/6-311+g(d,p) in the gas phase

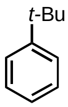

Atom	No.	x	y	z	Neutral pop	Cation pop	Anion pop	Fukui radical
C	1	2.215596	1.201819	-0.000013	-0.042817	0.037558	-0.073625	0.055592
C	2	2.908006	-0.004197	-0.000016	-0.045983	0.093704	-0.076888	0.085296
C	3	2.187713	-1.198346	-0.000055	-0.042269	0.015719	-0.074627	0.045173
C	4	0.796433	-1.177990	-0.000089	-0.041938	0.040131	-0.060977	0.050554
C	5	0.078173	0.028522	-0.000091	0.008291	0.119345	0.003626	0.057860
C	6	0.819204	1.215669	-0.000061	-0.045946	0.017128	-0.065606	0.041367
H	7	2.758524	2.140882	0.000055				
H	8	3.992103	-0.016603	0.000026				
H	9	2.710412	-2.148801	-0.000067				
H	10	0.264654	-2.122831	-0.000148				
H	11	0.313998	2.172604	0.000059				
C	12	-1.461372	0.005511	-0.000005				
C	13	-1.965602	-0.733195	-1.261760				
H	14	-3.059759	-0.759501	-1.274941				
H	15	-1.626669	-0.228301	-2.170590				
H	16	-1.607229	-1.764379	-1.299603				
C	17	-2.069557	1.420170	-0.000123				
H	18	-3.160556	1.348003	0.000297				
H	19	-1.776743	1.990286	0.885840				
H	20	-1.777469	1.989754	-0.886683				
C	21	-1.965150	-0.732945	1.262103				
H	22	-1.607009	-1.764217	1.299865				
H	23	-1.625609	-0.228024	2.170694				
H	24	-3.059305	-0.758981	1.275866				

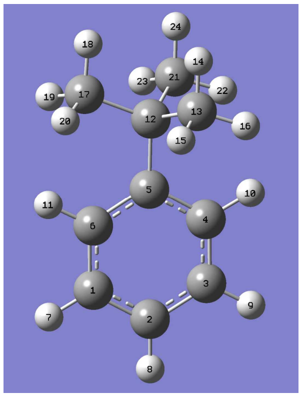

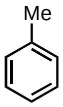

Atom	No.	x	y	z	Neutral pop	Cation pop	Anion pop	Fukui radical
C	1	-0.004273	0.913135	0.000000	0.007710	0.131904	-0.035517	0.083711
C	2	-0.007555	0.194230	1.200484	-0.046352	0.032601	-0.153311	0.092956
C	3	-0.007555	-1.199548	1.203415	-0.041410	0.033086	-0.155552	0.094319
C	4	-0.006636	-1.902681	0.000000	-0.046134	0.104356	-0.104221	0.104289
C	5	-0.007555	-1.199548	-1.203415	-0.041410	0.033086	-0.155552	0.094319
C	6	-0.007555	0.194230	-1.200484	-0.046352	0.032601	-0.153311	0.092956
H	7	-0.012602	0.732057	2.143638				
H	8	-0.012150	-1.736113	2.146085				
H	9	-0.009469	-2.986956	0.000000				
H	10	-0.012150	-1.736113	-2.146085				
H	11	-0.012602	0.732057	-2.143638				
C	12	0.029022	2.423022	0.000000				
H	13	-0.464553	2.832953	0.884499				
H	14	1.060711	2.792130	0.000000				
H	15	-0.464553	2.832953	-0.884499				

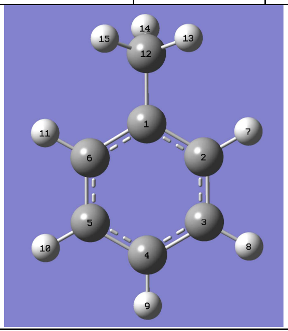

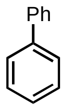

Atom	No.	x	y	z	Neutral pop	Cation pop	Anion pop	Fukui radical
**C**	1	0.000000	0.000000	-3.558930	-0.041397	0.052530	-0.126530	0.089530
**C**	2	-0.422636	1.127252	-2.856084	-0.039525	0.007747	-0.087211	0.047479
**C**	3	-0.422504	1.126861	-1.463621	-0.039758	0.008327	-0.084663	0.046495
**C**	4	0.000000	0.000000	-0.742734	0.002549	0.055149	-0.044860	0.050005
**C**	5	0.422504	-1.126861	-1.463621	-0.039758	0.008327	-0.084663	0.046495
**C**	6	0.422636	-1.127252	-2.856084	-0.039525	0.007747	-0.087211	0.047479
**H**	7	0.000000	0.000000	-4.643067				
**H**	8	-0.762195	2.006341	-3.392736				
**H**	9	-0.776173	2.000742	-0.928032				
**H**	10	0.776173	-2.000742	-0.928032				
**H**	11	0.762195	-2.006341	-3.392736				
**C**	12	0.000000	0.000000	0.742734				
**C**	13	-0.422504	-1.126861	1.463621				
**C**	14	0.422504	1.126861	1.463621				
**C**	15	-0.422636	-1.127252	2.856084				
**H**	16	-0.776173	-2.000742	0.928032				
**C**	17	0.422636	1.127252	2.856084				
**H**	18	0.776173	2.000742	0.928032				
**C**	19	0.000000	0.000000	3.558930				
**H**	20	-0.762195	-2.006341	3.392736				
**H**	21	0.762195	2.006341	3.392736				
**H**	22	0.000000	0.000000	4.643067				

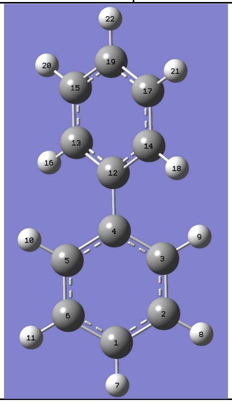

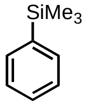

Atom	No.	x	y	z	Neutral pop	Cation pop	Anion pop	Fukui radical
**C**	1	0.030540	0.429292	0.000000	-0.093126	0.010727	-0.101154	0.055941
**C**	2	0.031973	1.160673	-1.199770	-0.038116	0.024201	-0.055726	0.039964
**C**	3	0.031973	2.554979	-1.204164	-0.041460	0.021626	-0.068006	0.044816
**C**	4	0.031593	3.256561	0.000000	-0.038791	0.088928	-0.070848	0.079888
**C**	5	0.031973	2.554979	1.204164	-0.041460	0.021626	-0.068006	0.044816
**C**	6	0.031973	1.160673	1.199770	-0.038116	0.024201	-0.055726	0.039964
**H**	7	0.037492	0.639474	-2.152094				
**H**	8	0.034903	3.092937	-2.146211				
**H**	9	0.033367	4.341053	0.000000				
**H**	10	0.034903	3.092937	2.146211				
**H**	11	0.037492	0.639474	2.152094				
**Si**	12	-0.017874	-1.467737	0.000000				
**C**	13	0.851507	-2.127053	-1.542679				
**H**	14	1.890338	-1.788096	-1.593414				
**H**	15	0.857884	-3.221724	-1.537126				
**H**	16	0.351545	-1.810330	-2.462734				
**C**	17	0.851507	-2.127053	1.542679				
**H**	18	0.351545	-1.810330	2.462734				
**H**	19	0.857884	-3.221724	1.537126				
**H**	20	1.890338	-1.788096	1.593414				
**C**	21	-1.817359	-2.048325	0.000000				
**H**	22	-2.350479	-1.682135	-0.882477				
**H**	23	-1.880584	-3.141336	0.000000				
**H**	24	-2.350479	-1.682135	0.882477				

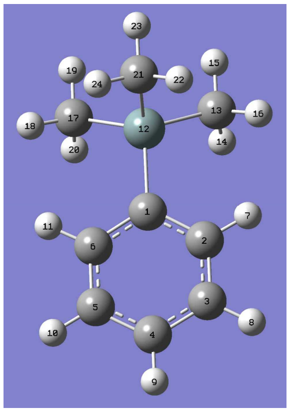

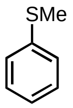

Atom	No.	x	y	z	Neutral pop	Cation pop	Anion pop	Fukui radical
**C**	1	0.123122	-0.042112	-0.267626	-0.014527	0.008036	-0.068402	0.038219
**C**	2	-0.542600	1.186866	-0.213619	-0.039117	0.000637	-0.089571	0.045104
**C**	3	-1.911925	1.230959	0.041971	-0.037349	0.003586	-0.086354	0.044970
**C**	4	-2.627078	0.049560	0.235031	-0.035861	0.033744	-0.128586	0.081165
**C**	5	-1.969159	-1.177464	0.170769	-0.037256	0.019831	-0.091207	0.055519
**C**	6	-0.598262	-1.225061	-0.079480	-0.039255	-0.013059	-0.085231	0.036086
**H**	7	0.012952	2.102429	-0.379975				
**H**	8	-2.420778	2.187675	0.082432				
**H**	9	-3.693175	0.085251	0.428957				
**H**	10	-2.521594	-2.099528	0.313745				
**H**	11	-0.083422	-2.176983	-0.134393				
**S**	12	1.885024	-0.107748	-0.628299				
**C**	13	2.580453	0.149083	1.050533				
**H**	14	2.270257	-0.649032	1.725215				
**H**	15	3.665527	0.125295	0.939252				
**H**	16	2.282536	1.117873	1.452077				

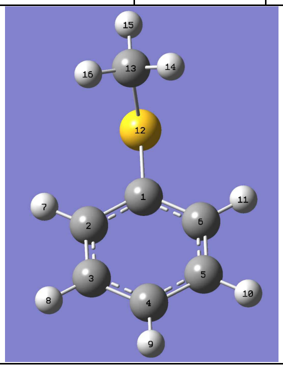

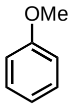

Atom	No.	x	y	z	Neutral pop	Cation pop	Anion pop	Fukui radical
**C**	1	-0.452231	-0.271665	-0.000037	0.071638	0.155008	0.059814	0.047597
**C**	2	-0.031524	1.060850	-0.000047	-0.068425	0.002788	-0.094752	0.048770
**C**	3	1.335900	1.349926	-0.000020	-0.040064	0.032557	-0.076165	0.054361
**C**	4	2.281945	0.331394	0.000014	-0.056129	0.076013	-0.093085	0.084549
**C**	5	1.851001	-0.998337	0.000047	-0.037820	0.019489	-0.075089	0.047289
**C**	6	0.496771	-1.301942	0.000035	-0.056346	0.031377	-0.083510	0.057444
**H**	7	-0.746871	1.872292	-0.000055				
**H**	8	1.654152	2.386683	-0.000024				
**H**	9	3.339939	0.565004	0.000041				
**H**	10	2.576570	-1.804251	0.000111				
**H**	11	0.147482	-2.327534	0.000084				
**O**	12	-1.758926	-0.669722	-0.000198				
**C**	13	-2.773238	0.324797	0.000136				
**H**	14	-2.716569	0.955236	-0.894072				
**H**	15	-3.718917	-0.214890	0.000320				
**H**	16	-2.716124	0.955097	0.894416				

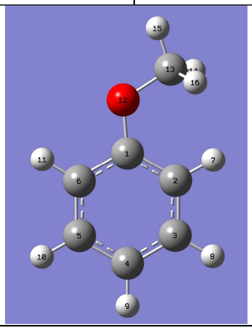

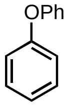

Atom	No.	x	y	z	Neutral pop	Cation pop	Anion pop	Fukui radical
**C**	1	-0.242257	1.177455	0.500783	0.074527	0.114670	0.056690	0.028990
**C**	2	-1.037505	1.230347	-0.644410	-0.053986	-0.011029	-0.101759	0.045365
**C**	3	-1.299950	2.465036	-1.234591	-0.036446	0.003180	-0.091359	0.047270
**C**	4	-0.784729	3.639149	-0.686719	-0.049235	0.034720	-0.088003	0.061362
**C**	5	0.000000	3.572527	0.463944	-0.036130	0.010365	-0.100081	0.055223
**C**	6	0.276017	2.344262	1.059734	-0.051691	-0.005254	-0.101255	0.048001
**H**	7	-1.446837	0.319633	-1.063671				
**H**	8	-1.919273	2.506728	-2.123647				
**H**	9	-0.995262	4.595806	-1.150054				
**H**	10	0.403928	4.479180	0.900226				
**H**	11	0.883496	2.272694	1.953751				
**O**	12	0.000000	0.000000	1.182737				
**C**	13	0.242257	-1.177455	0.500783				
**C**	14	-0.276017	-2.344262	1.059734				
**C**	15	1.037505	-1.230347	-0.644410				
**C**	16	0.000000	-3.572527	0.463944				
**H**	17	-0.883496	-2.272694	1.953751				
**C**	18	1.299950	-2.465036	-1.234591				
**H**	19	1.446837	-0.319633	-1.063671				
**C**	20	0.784729	-3.639149	-0.686719				
**H**	21	-0.403928	-4.479180	0.900226				
**H**	22	1.919273	-2.506728	-2.123647				
**H**	23	0.995262	-4.595806	-1.150054				

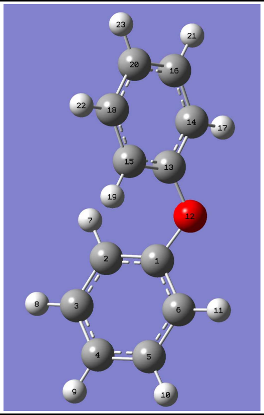

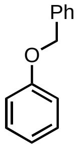

Atom	No.	x	y	z	Neutral pop	Cation pop	Anion pop	Fukui radical
**C1**	1	-1.774097	0.000349	-0.230948	0.068207	0.130140	0.043512	0.043314
**C2**	2	-2.465172	1.210201	-0.168721	-0.050847	-0.005187	-0.077585	0.036199
**C3**	3	-3.853467	1.205776	-0.042866	-0.037908	0.009414	-0.071734	0.040574
**C4**	4	-4.550375	-0.000366	0.020571	-0.046103	0.047388	-0.092120	0.069754
**C5**	5	-3.853244	-1.206140	-0.047268	-0.037905	0.009352	-0.072398	0.040875
**C6**	6	-2.464949	-1.209849	-0.173137	-0.050841	-0.005106	-0.076938	0.035916
**H**	7	-1.910875	2.139478	-0.234971				
**H**	8	-4.391341	2.146679	-0.001122				
**H**	9	-5.630266	-0.000639	0.115426				
**H**	10	-4.390945	-2.147288	-0.008958				
**H**	11	-1.910481	-2.138777	-0.242778				
**O**	12	-0.404320	0.000796	-0.406584				
**C13**	13	0.354621	-0.001343	0.803827				
**H**	14	0.143045	-0.895550	1.401808				
**H**	15	0.142880	0.890638	1.405066				
**C16**	16	1.837623	-0.000449	0.388731	-0.002025	0.047582	-0.033134	0.040358
**C17**	17	2.510436	-1.208320	0.202076	-0.037812	-0.005408	-0.070162	0.032377
**C18**	18	2.508175	1.207860	0.199261	-0.037768	-0.004912	-0.070575	0.032832
**C19**	19	3.853682	-1.207788	-0.173308	-0.038881	0.002607	-0.076892	0.039750
**H**	20	1.981713	-2.160756	0.352278				
**C21**	21	3.851562	1.208565	-0.177198	-0.038876	0.002201	-0.076537	0.039369
**H**	22	1.977997	2.159943	0.346158				
**C23**	23	4.524408	0.001023	-0.363365	-0.039246	0.039138	-0.095923	0.067531
**H**	24	4.384238	-2.159804	-0.319840				
**H**	25	4.379893	2.161416	-0.326915				
**H**	26	5.583492	0.001193	-0.659399				

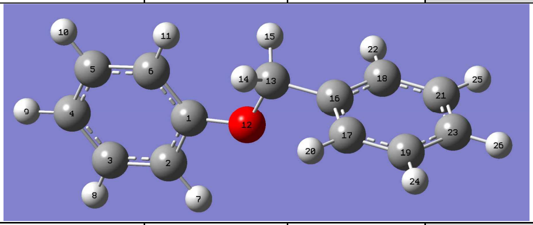

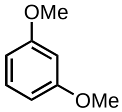

Atom	No.	x	y	z	Neutral pop	Cation pop	Anion pop	Fukui radical
**C**	1	-0.000319	1.207498	0.242213	0.071720	0.136242	0.064330	0.035956
**C**	2	0.000000	0.000000	-0.467481	-0.093988	-0.062637	-0.100164	0.018764
**C**	3	0.000319	-1.207498	0.242213	0.071720	0.136242	0.064330	0.035956
**C**	4	0.000385	-1.212535	1.638358	-0.073554	0.049209	-0.095022	0.072116
**C**	5	0.000000	0.000000	2.319521	-0.036500	0.012242	-0.062307	0.037275
**C**	6	-0.000385	1.212535	1.638358	-0.073554	0.049209	-0.095022	0.072116
**H**	7	0.000000	0.000000	-1.546363				
**H**	8	0.000583	-2.160699	2.160592				
**H**	9	0.000000	0.000000	3.403809				
**H**	10	-0.000583	2.160699	2.160592				
**O**	11	-0.000866	2.435633	-0.357405				
**O**	12	0.000866	-2.435633	-0.357405				
**C**	13	0.000000	2.510937	-1.774797				
**H**	14	-0.000221	3.572853	-2.015907				
**H**	15	0.895058	2.044807	-2.201781				
**H**	16	-0.894312	2.044426	-2.203148				
**C**	17	0.000000	-2.510937	-1.774797				
**H**	18	0.894312	-2.044426	-2.203148				
**H**	19	0.000221	-3.572853	-2.015907				
**H**	20	-0.895058	-2.044807	-2.201781				

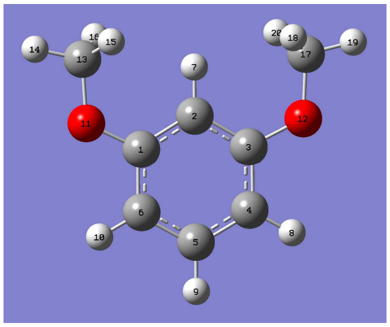

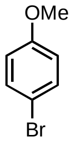

Atom	No.	x	y	z	Neutral pop	Cation pop	Anion pop	Fukui radical
**C**	1	0.727837	0.044683	0.000007	-0.008733	0.069382	-0.037998	0.053690
**C**	2	0.141670	1.310483	0.000033	-0.039054	0.007874	-0.075311	0.041593
**C**	3	-1.241042	1.423210	0.000011	-0.047973	0.020957	-0.085985	0.053471
**C**	4	-2.047114	0.277767	-0.000064	0.073571	0.147878	0.056176	0.045851
**C**	5	-1.450302	-0.985336	-0.000062	-0.060822	0.000881	-0.094506	0.047694
**C**	6	-0.057677	-1.098239	-0.000031	-0.041690	0.016883	-0.080185	0.048534
**H**	7	0.759077	2.199586	0.000068				
**H**	8	-1.718103	2.395720	0.000010				
**H**	9	-2.046623	-1.887590	-0.000032				
**H**	10	0.401272	-2.078506	-0.000026				
**Br**	11	2.640701	-0.114857	0.000008				
**O**	12	-3.392272	0.498336	-0.000100				
**C**	13	-4.266232	-0.623246	0.000108				
**H**	14	-5.274736	-0.213523	-0.000028				
**H**	15	-4.124998	-1.239356	-0.894429				
**H**	16	-4.125078	-1.238941	0.894946				

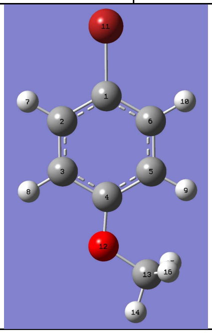

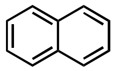

Atom	No.	x	y	z	Neutral pop	Cation pop	Anion pop	Fukui radical
**C**	1	0.000000	2.430636	0.707533	-0.041548	0.029009	-0.112420	0.070715
**C**	2	0.000000	1.243878	1.400696	-0.039022	0.051638	-0.119466	0.085552
**C**	3	0.000000	0.000000	0.715961	0.000506	0.015830	-0.017030	0.016430
**C**	4	0.000000	0.000000	-0.715961	0.000506	0.015830	-0.017030	0.016430
**C**	5	0.000000	1.243878	-1.400696	-0.039022	0.051638	-0.119466	0.085552
**C**	6	0.000000	2.430636	-0.707533	-0.041548	0.029009	-0.112420	0.070715
**H**	7	0.000000	-1.243077	2.485974				
**H**	8	0.000000	3.373015	1.243930				
**H**	9	0.000000	1.243077	2.485974				
**C**	10	0.000000	-1.243878	1.400696				
**C**	11	0.000000	-1.243878	-1.400696				
**H**	12	0.000000	1.243077	-2.485974				
**H**	13	0.000000	3.373015	-1.243930				
**C**	14	0.000000	-2.430636	-0.707533				
**C**	15	0.000000	-2.430636	0.707533				
**H**	16	0.000000	-1.243077	-2.485974				
**H**	17	0.000000	-3.373015	-1.243930				
**H**	18	0.000000	-3.373015	1.243930				

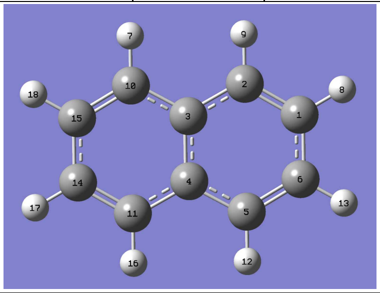

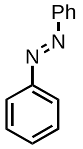

Atom	No.	x	y	z	Neutral pop	Cation pop	Anion pop	Fukui radical
**C**	1	-0.002856	1.779960	-0.000001	0.021954	0.042534	0.003933	0.019301
**C**	2	1.003050	2.752140	0.000002	-0.033787	-0.004889	-0.073495	0.034303
**C**	3	0.669677	4.103968	0.000003	-0.037666	-0.002891	-0.077024	0.037067
**C**	4	-0.669677	4.488544	0.000002	-0.031231	0.021957	-0.099870	0.060914
**C**	5	-1.676123	3.516962	-0.000001	-0.035045	0.002278	-0.069534	0.035906
**C**	6	-1.351753	2.167444	-0.000002	-0.036635	-0.014829	-0.078878	0.032025
**H**	7	2.036241	2.425532	0.000003				
**H**	8	1.451923	4.854323	0.000005				
**H**	9	-0.933060	5.540248	0.000002				
**H**	10	-2.717430	3.819696	-0.000002				
**H**	11	-2.118627	1.404177	-0.000004				
**N**	12	0.450376	0.434912	-0.000002				
**N**	13	-0.450376	-0.434912	-0.000002				
**C**	14	0.002856	-1.779960	-0.000001				
**C**	15	-1.003050	-2.752140	0.000002				
**C**	16	1.351753	-2.167444	-0.000002				
**C**	17	-0.669677	-4.103968	0.000003				
**H**	18	-2.036241	-2.425532	0.000003				
**C**	19	1.676123	-3.516962	-0.000001				
**H**	20	2.118627	-1.404177	-0.000004				
**C**	21	0.669677	-4.488544	0.000002				
**H**	22	-1.451923	-4.854323	0.000005				
**H**	23	2.717430	-3.819696	-0.000002				
**H**	24	0.933060	-5.540248	0.000002				

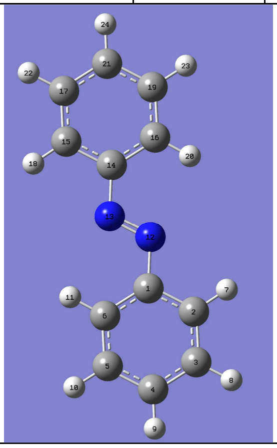

5.2.2 Electrophilicity of Aminyl and Aminium Radicals

NCR	Ionization Potential (I, eV)[a]	Electron Affinity (A, eV)[a]	Electronic Chemical Potential (μ, eV)	Chemical Hardness (η, eV)	Fukui Nucleoph. attack in N (f^+)	Global Electrophilicity (ω, eV)	Electrophilicity (ω^{+}_{rc}, eV)
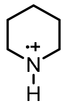	6.016621	5.649503	-5.833062	0.367117	0.281500	46.340246	**13.044779**
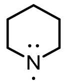	6.733577	2.976937	-4.855257	3.756640	0.380200	3.137581	**1.192908**

[a] Calculated ΔG. All the involved species were fully optimized.

5.2.3 Optimized Energies (in Hartrees) and Geometries

B3LYP/6-311+g(d,p) in acetonitrile

Molecule	Total electronic energy	Sum of electronic + ZPE	Gibbs Free Energy
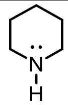	-251.981446	-251.823227	-251.851925
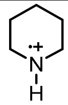	-251.772642	-251.614791	-251.644299
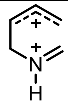	-251.544701	-251.391204	-251.423181
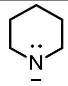	-251.432300	-251.290615	-251.319093
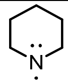	-251.324342	-251.180275	-251.209687
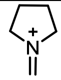	-251.196369	-251.048974	-251.078189
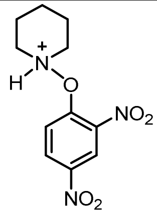	-967.826691	-967.565655	-967.612042
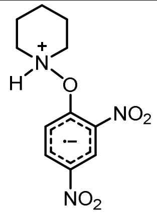	-968.039982	-967.786199	-967.836813
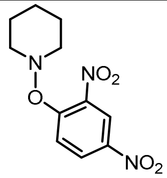	-967.410766	-967.164787	-967.211792
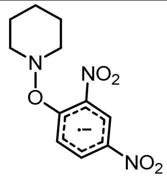	-967.539955	-967.296814	-967.344743

Molecule	Total electronic energy	Sum of electronic + ZPE	Gibbs Free Energy
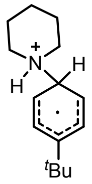	-641.373183	-640.998485	-641.042552
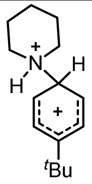	-641.175713	-640.799040	-640.842082
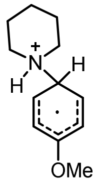	-598.637063	-598.341595	-598.382485
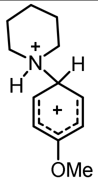	-598.455586	-598.157566	-598.197375
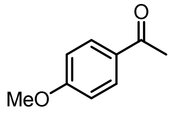	-499.570026	-499.400703	-499.437183
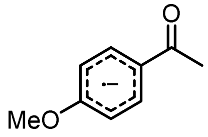	-499.648857	-499.483925	-499.522918
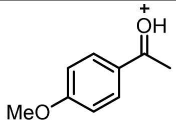	-499.987702	-499.804971	-499.841538
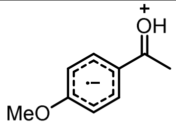	-500.130206	-499.951421	-499.989409

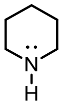

	X	Y	Z
C	0.0084070	-0.7897380	1.2215320
C	0.0084070	0.7464120	1.2641290
C	-0.6415390	1.3280350	0.0000000
C	0.0084070	0.7464120	-1.2641290
C	0.0084070	-0.7897380	-1.2215320
H	-0.5147470	1.0929610	2.1623230
H	-1.0253910	-1.1533960	1.2813850
H	0.5402200	-1.1999990	2.0846610
H	-1.7117910	1.0826780	0.0000000
H	-0.5710830	2.4203440	0.0000000
H	-0.5147470	1.0929610	-2.1623230
H	1.0440100	1.1021990	-1.3410500
H	-1.0253910	-1.1533960	-1.2813850
H	0.5402200	-1.1999990	-2.0846610
H	1.0440100	1.1021990	1.3410500
N	0.6055560	-1.3541330	0.0000000
H	1.6032590	-1.1559210	0.0000000

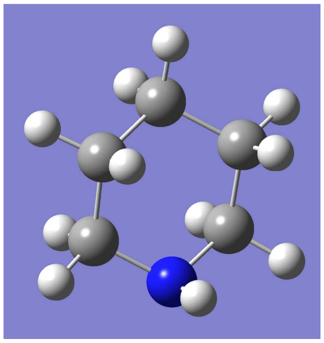

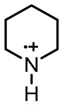

	X	Y	Z
C	0.0095700	-0.8154740	1.2378010
C	0.0095700	0.7564420	1.2668080
C	-0.6409190	1.3104280	0.0000000
C	0.0095700	0.7564420	-1.2668080
C	0.0095700	-0.8154740	-1.2378010
H	-0.5302650	1.0490150	2.1692570
H	-1.0278690	-1.1615690	1.2605270
H	0.5689060	-1.2330730	2.0710500
H	-1.7121930	1.0857620	0.0000000
H	-0.5438160	2.4010730	0.0000000
H	-0.5302650	1.0490150	-2.1692570
H	1.0423820	1.0993300	-1.3625910
H	-1.0278690	-1.1615690	-1.2605270
H	0.5689060	-1.2330730	-2.0710500
H	1.0423820	1.0993300	1.3625910
N	0.6005320	-1.2265270	0.0000000
H	1.5618110	-1.5627320	0.0000000

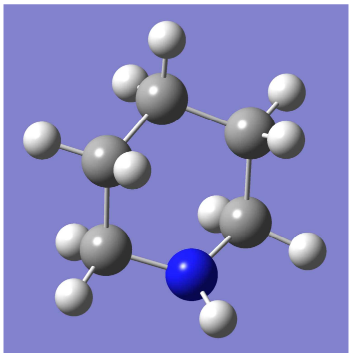

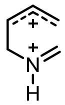

	X	Y	Z
C	2.4100050	0.9213340	0.1279260
C	-2.6928910	0.6114100	-0.1680330
C	-1.4415640	0.2001970	0.3898820
C	-0.6286940	-0.8782790	-0.1307470
C	0.8465760	-0.9528850	0.2725330
H	-2.4003470	1.5839370	-0.6363740
H	2.4511540	0.9832210	1.2091090
H	2.9964350	1.5919270	-0.4878320
H	-1.0945130	0.7292920	1.2784410
H	-3.4116480	0.9182820	0.5972510
H	-1.1442570	-1.7378950	0.3636160
H	-0.8177790	-1.0652570	-1.1920100
H	0.9835830	-0.8069200	1.3420780
H	1.2406980	-1.9316440	-0.0009370
H	-3.0999710	-0.0330540	-0.9435000
N	1.6672550	0.0498970	-0.4323830
H	1.6652660	0.0081670	-1.4525210

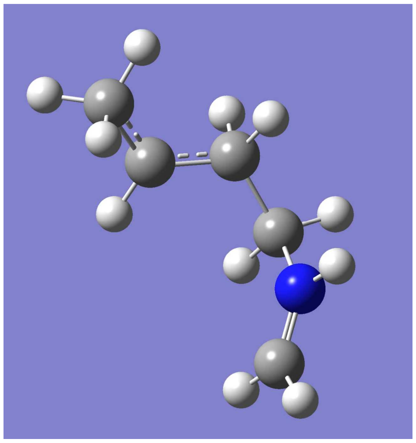

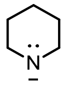

	X	Y	Z
C	0.0350280	-0.8319340	1.1705720
C	0.0350280	0.7143730	1.2666470
C	-0.5854340	1.3234120	0.0000000
C	0.0350280	0.7143730	-1.2666470
C	0.0350280	-0.8319340	-1.1705720
H	-0.5139170	1.0524640	2.1585260
H	-1.0503770	-1.1336640	1.2274140
H	0.5011450	-1.2416280	2.0794160
H	-1.6633580	1.1056250	0.0000000
H	-0.4934150	2.4164560	0.0000000
H	-0.5139170	1.0524640	-2.1585260
H	1.0724510	1.0586890	-1.3732660
H	-1.0503770	-1.1336640	-1.2274140
H	0.5011450	-1.2416280	-2.0794160
H	1.0724510	1.0586890	1.3732660
N	0.6871580	-1.3605050	0.0000000

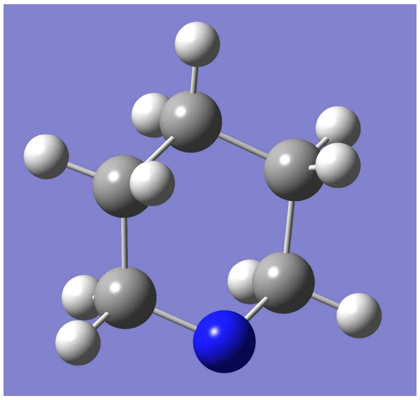

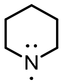

	X	Y	Z
C	0.0325820	-0.8348430	1.1950740
C	0.0325820	0.7126270	1.2594620
C	-0.6072010	1.3060020	0.0000000
C	0.0325820	0.7126270	-1.2594620
C	0.0325820	-0.8348430	-1.1950740
H	-0.4971670	1.0285930	2.1627770
H	-1.0146230	-1.1813210	1.2003070
H	0.5285830	-1.2523470	2.0748510
H	-1.6821270	1.0883720	0.0000000
H	-0.5035300	2.3955960	0.0000000
H	-0.4971670	1.0285930	-2.1627770
H	1.0668690	1.0613440	-1.3532780
H	-1.0146230	-1.1813210	-1.2003070
H	0.5285830	-1.2523470	-2.0748510
H	1.0668690	1.0613440	1.3532780
N	0.6970790	-1.3094180	0.0000000

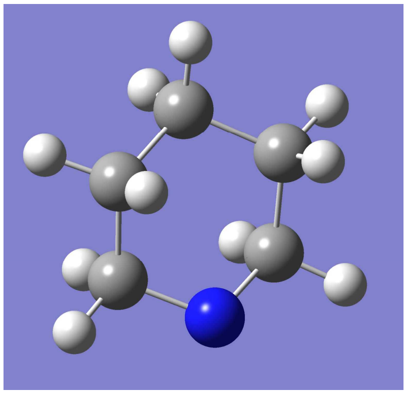

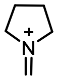

	X	Y	Z
C	2.1088160	0.0000010	0.0000030
C	-0.0163610	1.2243140	-0.1265120
C	-1.4144760	0.7297070	0.2422870
C	-1.4144790	-0.7297010	-0.2422780
C	-0.0163630	-1.2243190	0.1265020
H	0.3840740	2.0066160	0.5146040
H	2.6478840	-0.9364160	0.0823470
H	2.6478750	0.9364230	-0.0823270
H	-1.5591600	0.7752960	1.3241030
H	-2.1858350	1.3349040	-0.2321960
H	-2.1858350	-1.3348980	0.2322110
H	-1.5591770	-0.7752860	-1.3240930
H	0.0398900	-1.5428520	1.1689940
H	0.3840580	-2.0066180	-0.5146280
H	0.0398820	1.5428350	-1.1690100
N	0.8376460	-0.0000020	-0.0000010

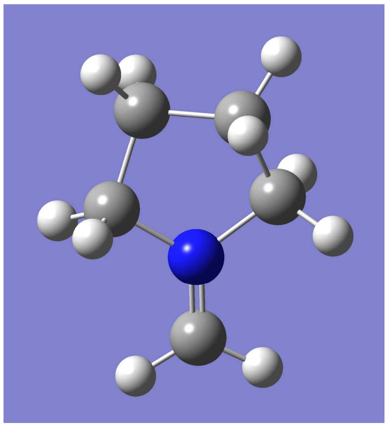

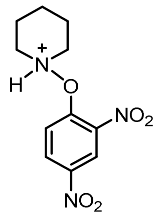

	X	Y	Z
C	-1.0723270	0.9985610	-0.1332340
C	-0.5745510	-1.2675800	-0.8018270
C	-2.3875700	0.6535670	0.1324480
C	-1.8913860	-1.6254610	-0.5449720
H	0.1048610	-2.0231870	-1.1748260
C	-2.7771260	-0.6588360	-0.0844900
H	-3.0857060	1.3932930	0.4966900
H	-2.2275440	-2.6387150	-0.7122280
N	-0.6843160	2.4039280	0.0799510
O	-1.1755980	2.9734530	1.0450270
O	0.0778450	2.9144700	-0.7265960
N	-4.1798310	-1.0338220	0.1823910
O	-4.4952940	-2.2043190	0.0157330
O	-4.9434860	-0.1531240	0.5551330
C	-0.1531510	0.0427890	-0.5946290
O	1.1360340	0.4685990	-0.8688450
C	3.4476890	0.1178220	-1.0802810
C	2.3007650	-0.3660060	1.1091920
C	4.6408410	-0.7291580	-0.6423890
H	3.5414670	1.1603170	-0.7776460
H	3.2548320	0.0609140	-2.1495170
C	3.4939750	-1.2141450	1.5488610
H	2.4174850	0.6848120	1.3738790
H	1.3588480	-0.7430680	1.5038130
C	4.7940460	-0.7523370	0.8823670
H	5.5227660	-0.2999580	-1.1224940
H	4.5337650	-1.7459990	-1.0337060
H	3.5537510	-1.1279220	2.6359640
H	3.2992120	-2.2684850	1.3285410
H	5.6102950	-1.4208010	1.1621010
H	5.0586190	0.2468430	1.2434340
N	2.1964770	-0.3880720	-0.3984470
H	2.0077890	-1.3402100	-0.7311660

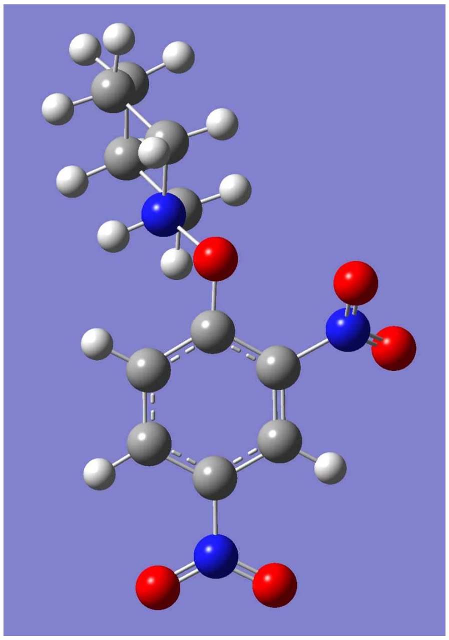

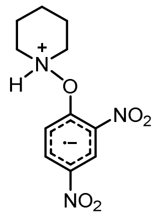

	X	Y	Z
C	-1.1566670	0.3682150	-0.0572940
C	-1.8160250	-1.9736590	0.0973540
C	-2.5000070	0.7295220	-0.0825520
C	-3.1314570	-1.6129210	0.0874700
H	-1.5304530	-3.0166440	0.1608010
C	-3.4795870	-0.2435330	-0.0030200
H	-2.7706610	1.7720970	-0.1538220
H	-3.9135330	-2.3574940	0.1467810
N	-0.2034500	1.4488080	-0.1417400
O	-0.5942170	2.5587080	-0.5229990
O	0.9724750	1.2401080	0.1752270
N	-4.8651280	0.1476650	-0.0071310
O	-5.7244020	-0.7387720	0.0685130
O	-5.1495890	1.3484730	-0.0871570
C	-0.7294120	-1.0206930	0.0286000
O	0.4622900	-1.4321660	0.0169350
C	3.5786200	0.0783050	1.3851940
C	3.8513010	-1.4997880	-0.5030770
C	4.4698130	1.1330070	0.6653560
H	4.2162500	-0.5235590	2.0454740
H	2.7860450	0.5434360	1.9661080
C	4.7444630	-0.4765810	-1.2642470
H	4.5017250	-2.1659380	0.0782910
H	3.2392600	-2.0933960	-1.1793170
C	5.4605730	0.4525320	-0.2815480
H	4.9817000	1.7092580	1.4387040
H	3.8167580	1.8158270	0.1163700
H	5.4514510	-1.0472060	-1.8697740
H	4.1109630	0.0980600	-1.9453610
H	6.0120550	1.2151630	-0.8392710
H	6.1981620	-0.1149840	0.2964670
N	2.9934470	-0.7970410	0.4096960
H	1.9440600	-0.8478660	0.2752050

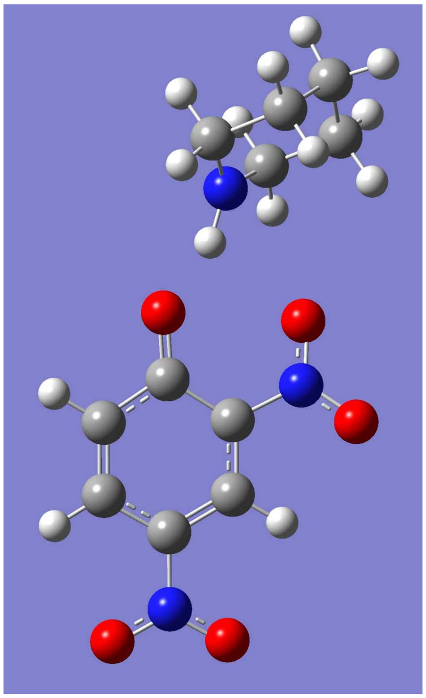

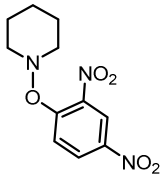

	X	Y	Z
C	-1.2085570	1.0353860	-0.0287290
C	-0.3488980	-1.2237850	-0.0927510
C	-2.5079340	0.5552960	0.0147590
C	-1.6425060	-1.7070030	-0.0731940
H	0.4950810	-1.8952400	-0.1178320
C	-2.7154950	-0.8141700	-0.0191970
H	-3.3382990	1.2438270	0.0643800
H	-1.8287990	-2.7715870	-0.0962530
N	-1.0457050	2.4937780	-0.0164680
O	-1.8837060	3.1491080	0.5979790
O	-0.1076520	2.9826920	-0.6321010
N	-4.0838570	-1.3244410	0.0028290
O	-4.2455970	-2.5421450	-0.0261480
O	-5.0063010	-0.5138560	0.0503450
C	-0.0953890	0.1590920	-0.0770980
O	1.1327690	0.6928860	-0.0722780
C	3.0522600	-0.0169210	-1.1993600
C	2.9439830	0.0073910	1.2387390
C	4.2670340	-0.9508950	-1.1748290
H	3.3765800	1.0338890	-1.2093020
H	2.4395980	-0.1973100	-2.0843390
C	4.1551910	-0.9258150	1.3408570
H	3.2668600	1.0585710	1.2560980
H	2.2554350	-0.1561690	2.0696090
C	5.0705400	-0.7781850	0.1195970
H	4.8842510	-0.7362490	-2.0512970
H	3.9247360	-1.9871250	-1.2667300
H	4.6923670	-0.6930550	2.2641860
H	3.8050650	-1.9602480	1.4227280
H	5.8827700	-1.5089310	0.1631030
H	5.5343370	0.2153440	0.1302520
N	2.2256390	-0.2889860	-0.0120380

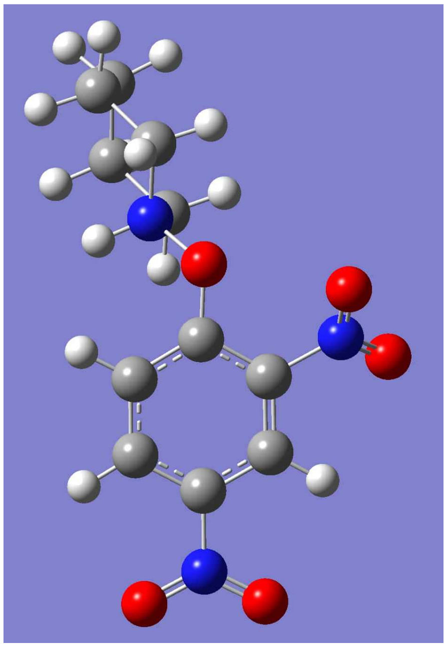

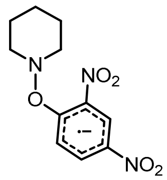

	X	Y	Z
C	-1.2043690	1.0371060	-0.0695630
C	-0.3561110	-1.2078590	-0.1810250
C	-2.5095030	0.5584680	0.0265260
C	-1.6472760	-1.6968120	-0.1058720
H	0.4835220	-1.8842450	-0.2389950
C	-2.7515840	-0.8210830	-0.0030710
H	-3.3321420	1.2508750	0.1080090
H	-1.8285710	-2.7619640	-0.1257370
N	-1.0394060	2.4879870	-0.0497380
O	-1.8682160	3.1545450	0.5739410
O	-0.1016310	2.9881640	-0.6664120
N	-4.0546270	-1.3155100	0.0632060
O	-4.2502930	-2.5965060	0.0178500
O	-5.0448970	-0.4851490	0.1644770
C	-0.0928580	0.1706870	-0.1750270
O	1.1613630	0.7020930	-0.2407890
C	3.0918180	-0.1662040	-1.2210230
C	2.9243320	0.1520580	1.1882780
C	4.2870490	-1.1103000	-1.0546880
H	3.4392900	0.8701880	-1.3524440
H	2.4977920	-0.4398610	-2.0951110
C	4.1141350	-0.7809160	1.4367000
H	3.2685120	1.1933620	1.0926130
H	2.2116120	0.1021300	2.0137940
C	5.0622250	-0.7985560	0.2313520
H	4.9315110	-1.0144650	-1.9329050
H	3.9264250	-2.1443540	-1.0304210
H	4.6348450	-0.4485610	2.3390780
H	3.7419300	-1.7922580	1.6327670
H	5.8583720	-1.5328030	0.3836660
H	5.5463510	0.1808460	0.1354170
N	2.2287170	-0.2729910	-0.0360090

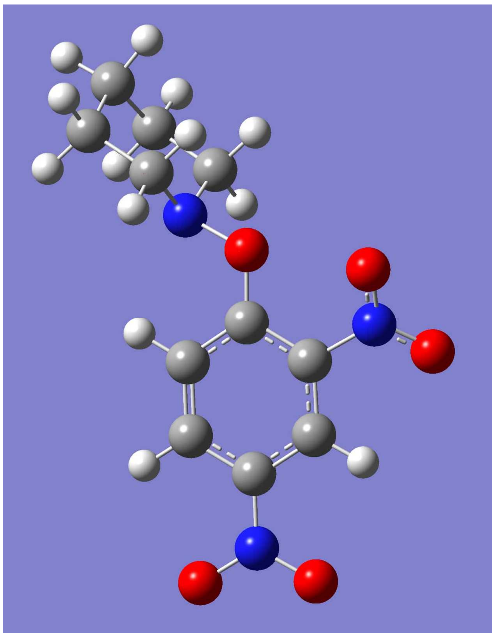

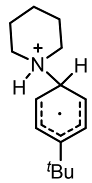

	X	Y	Z
C	-3.4762110	-0.8508860	0.4070030
C	-3.7469690	0.5621490	0.9112340
C	-3.6351640	1.5943680	-0.2150500
C	-2.2629320	1.4969210	-0.8883820
C	-1.9848330	0.0912380	-1.4108870
H	-4.7542800	0.5569890	1.3356560
H	-4.2565070	-1.1517190	-0.2951110
H	-3.4480280	-1.5823020	1.2142690
H	-4.4252650	1.4199590	-0.9544930
H	-3.7869300	2.6001970	0.1826700
H	-2.2029260	2.1706640	-1.7472310
H	-1.4793140	1.8034750	-0.1924520
H	-2.6808640	-0.1592280	-2.2138420
H	-0.9713170	-0.0165350	-1.7947990
H	-3.0691990	0.8194180	1.7283160
N	-2.1706700	-0.9943100	-0.3618470
C	-0.8811230	-1.3157950	0.5283170
C	0.1406020	-1.9438730	-0.3581410
C	-0.3570770	-0.1409230	1.2791510
C	1.3896110	-1.4163970	-0.5236550
H	-0.1325250	-2.8611780	-0.8697250
C	0.9030280	0.3340120	1.0807590
H	-0.9874190	0.3063150	2.0362410
C	1.8234590	-0.2481680	0.1573210
H	2.0700160	-1.9304240	-1.1899110
H	1.2192550	1.1851270	1.6725910
H	-2.2754810	-1.8615820	-0.8904500
C	4.0477360	-0.3829420	-1.0819030
H	5.0198590	0.1053360	-1.1857550
H	4.2308500	-1.4222170	-0.7962160
H	3.5665890	-0.3745630	-2.0637550
H	-1.3019640	-2.0580930	1.2175540
C	3.2114430	0.3655930	-0.0280360
C	3.0618830	1.8388640	-0.4826230
H	2.5125950	2.4385790	0.2466410
H	4.0504770	2.2898210	-0.6080990
H	2.5357970	1.9006500	-1.4392490
C	3.9734740	0.3228350	1.3205780
H	4.1012280	-0.7072490	1.6642620
H	4.9657440	0.7669120	1.1989710
H	3.4527310	0.8798430	2.1027430

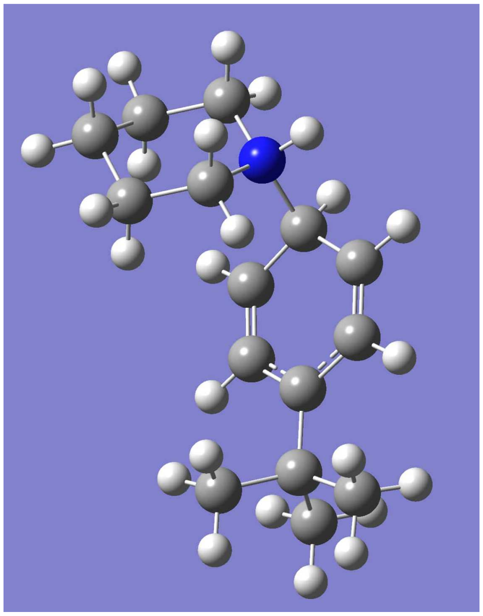

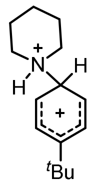

	X	Y	Z
C	-3.3336060	-0.6064120	1.0455600
C	-3.7005620	0.8686390	0.9171500
C	-3.9130700	1.2998830	-0.5378490
C	-2.7023720	0.9232090	-1.3966230
C	-2.4300020	-0.5711160	-1.3377010
H	-4.6194360	0.9979450	1.4945390
H	-4.1826080	-1.2315530	0.7679120
H	-3.0246560	-0.8790490	2.0536420
H	-4.8093290	0.8168290	-0.9418690
H	-4.0837130	2.3769170	-0.5804840
H	-2.8779580	1.1666570	-2.4473150
H	-1.8170060	1.4932810	-1.0982760
H	-3.2896920	-1.1343770	-1.7024920
H	-1.5544610	-0.8680510	-1.9130780
H	-2.9570570	1.5063030	1.3991070
N	-2.2124600	-1.0748350	0.0968300
C	-0.7925520	-0.9155330	0.6005240
C	0.1629330	-1.7769450	-0.1401980
C	-0.3110530	0.4591150	0.8799660
C	1.4573010	-1.4083340	-0.3090730
H	-0.1726680	-2.7531750	-0.4753840
C	0.9895620	0.7888130	0.6871220
H	-0.9792150	1.1635850	1.3531780
C	1.9205330	-0.1221480	0.0994180
H	2.1392620	-2.0975220	-0.7837040
H	1.3212630	1.7744010	0.9810230
H	-2.3411810	-2.0884340	0.0521160
C	4.2333870	-0.7751720	-0.7385310
H	5.2528630	-0.3945100	-0.8180830
H	4.2725870	-1.6959620	-0.1519360
H	3.8923940	-1.0131060	-1.7489110
H	-0.7888450	-1.3802720	1.6115280
C	3.3572780	0.2980940	-0.0715260
C	3.3963280	1.5936210	-0.9351870
H	2.8569920	2.4227640	-0.4764120
H	4.4409660	1.8920860	-1.0442960
H	2.9836240	1.4138200	-1.9298720
C	3.9272250	0.6080280	1.3473760
H	3.8946200	-0.2763470	1.9867120
H	4.9699880	0.9099600	1.2293170
H	3.3925020	1.4192090	1.8426540

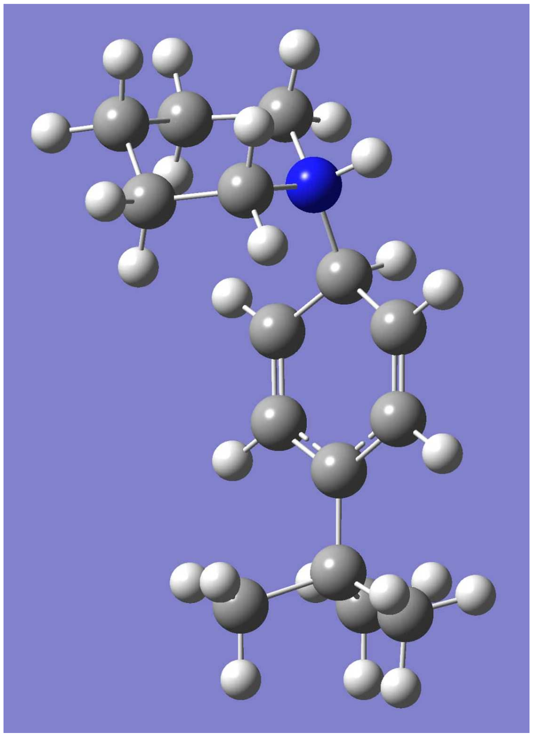

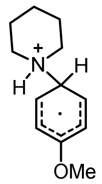

	X	Y	Z
C	2.8298260	1.0500960	0.1958830
C	3.2984630	-0.2678140	0.8018750
C	3.2389730	-1.4083020	-0.2193110
C	1.8238910	-1.5328000	-0.7924240
C	1.3410420	-0.2220410	-1.4066960
H	4.3223390	-0.1106390	1.1508400
H	3.5195140	1.3657680	-0.5900810
H	2.7709690	1.8499710	0.9340260
H	3.9525810	-1.2153330	-1.0287420
H	3.5364360	-2.3483050	0.2508080
H	1.7907160	-2.2852940	-1.5848300
H	1.1320040	-1.8634190	-0.0151720
H	1.9424980	0.0286910	-2.2832410
H	0.2974790	-0.2681600	-1.7155940
H	2.7057830	-0.5195200	1.6845230
N	1.4679530	0.9672500	-0.4717910
C	0.1997240	1.2250930	0.5097100
C	-0.9355770	1.6812070	-0.3394600
C	-0.1361640	0.0572980	1.3688490
C	-2.1125490	0.9895620	-0.4322610
H	-0.8123650	2.6044310	-0.8950050
C	-1.3217590	-0.5965160	1.2577970
H	0.5746020	-0.2467570	2.1256280
C	-2.3231050	-0.1777460	0.3375410
H	-2.8917020	1.3683680	-1.0805780
H	-1.5429510	-1.4428230	1.8981680
H	1.4197680	1.7918370	-1.0720330
O	-3.4428080	-0.9298100	0.3119200
C	-4.5156680	-0.5662790	-0.5700580
H	-5.2814490	-1.3241660	-0.4245950
H	-4.9162960	0.4161360	-0.3096520
H	-4.1818160	-0.5736130	-1.6101520
H	0.6060920	2.0500030	1.1042040

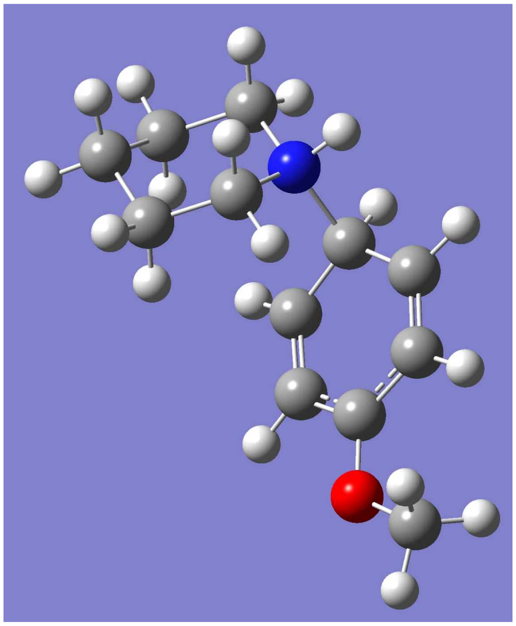

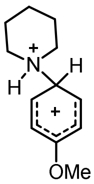

	X	Y	Z
C	2.7416550	0.8575460	0.8382770
C	3.2455320	-0.5815630	0.8564650
C	3.3904980	-1.1671650	-0.5522790
C	2.0823830	-1.0240570	-1.3360290
C	1.6355640	0.4281220	-1.4155840
H	4.2135330	-0.5537440	1.3630240
H	3.4937490	1.5120260	0.3965990
H	2.4997640	1.2362870	1.8302110
H	4.1951930	-0.6507670	-1.0867810
H	3.6743590	-2.2189890	-0.4866360
H	2.2070380	-1.3665080	-2.3663450
H	1.2968940	-1.6493580	-0.9019670
H	2.3792170	1.0324400	-1.9362800
H	0.6800980	0.5486310	-1.9243790
H	2.6073600	-1.2196030	1.4708420
N	1.4965180	1.0871460	-0.0365130
C	0.1501510	0.8693250	0.6439220
C	-0.9353540	1.5584910	-0.1185190
C	-0.1623450	-0.5314480	1.0544590
C	-2.1631870	1.0274970	-0.2658800
H	-0.7331840	2.5533680	-0.5017700
C	-1.3947690	-1.0468420	0.9043550
H	0.5935780	-1.0960750	1.5801920
C	-2.4233360	-0.2943020	0.2378180
H	-2.9411330	1.5819100	-0.7705630
H	-1.6475020	-2.0346090	1.2665410
H	1.5115690	2.0931400	-0.2180180
O	-3.5575640	-0.8915190	0.1327920
C	-4.7171560	-0.2886880	-0.5293810
H	-5.4938850	-1.0403450	-0.4424170
H	-4.9987230	0.6217940	-0.0041120
H	-4.4749820	-0.0995280	-1.5731700
H	0.2241200	1.4305680	1.5925450

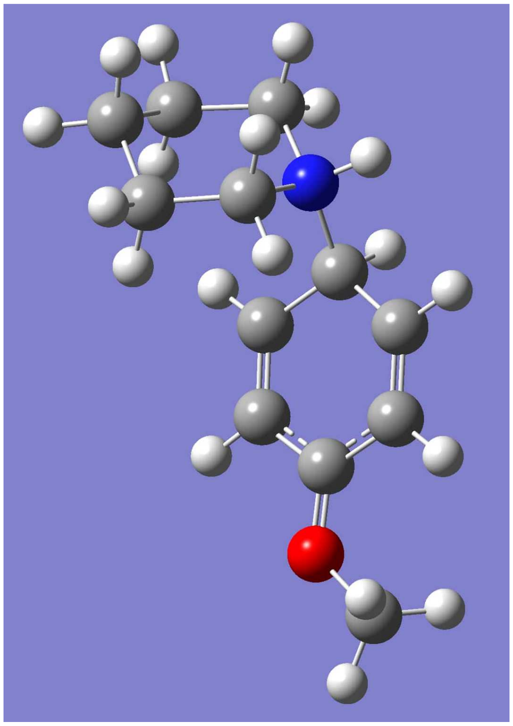

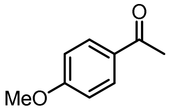

	X	Y	Z
C	-1.6820620	-0.2996820	-0.0000010
C	-0.8263860	-1.4121530	0.0000020
C	0.5462580	-1.2333820	0.0000030
C	1.1087640	0.0563920	0.0000000
C	0.2394320	1.1559610	-0.0000020
C	-1.1399070	0.9940260	-0.0000020
H	-1.2601630	-2.4048080	0.0000050
H	1.1816120	-2.1100380	0.0000040
H	0.6612440	2.1534840	-0.0000040
H	-1.7789880	1.8661540	-0.0000020
O	-3.0089850	-0.5732720	0.0000000
C	-3.9433440	0.5131910	0.0000000
H	-3.8272130	1.1296900	-0.8950090
H	-4.9283240	0.0519580	0.0000050
H	-3.8272070	1.1296960	0.8950030
C	2.5778710	0.2896680	0.0000010
C	3.5097860	-0.9041540	-0.0000040
H	3.3415520	-1.5298390	0.8808630
H	3.3415590	-1.5298260	-0.8808830
H	4.5403140	-0.5526780	0.0000030
O	3.0356270	1.4278960	0.0000050

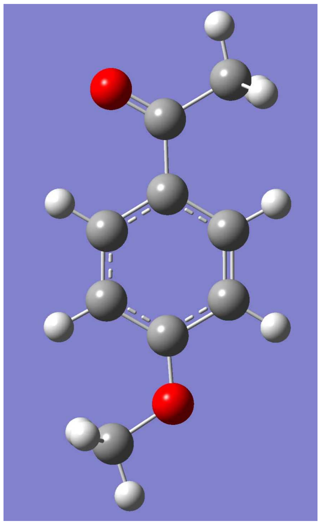

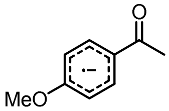

	X	Y	Z
C	-1.7047660	-0.0088450	-0.3040460
C	-0.9595140	-1.1982560	-0.2519360
C	0.4201340	-1.1641840	-0.1241420
C	1.1411480	0.0752730	-0.0365120
C	0.3440960	1.2698280	-0.1010020
C	-1.0333410	1.2229810	-0.2290490
H	-1.4782670	-2.1499480	-0.3218860
H	0.9596670	-2.1039510	-0.0955680
H	0.8515730	2.2257010	-0.0506590
H	-1.6107170	2.1418660	-0.2813810
O	-3.0886350	-0.0503660	-0.4839620
C	-3.8421250	-0.0792820	0.7327260
H	-3.6511740	0.8160980	1.3348250
H	-4.8966890	-0.1092380	0.4544570
H	-3.5979250	-0.9681160	1.3252900
C	2.5629030	0.1510450	0.0984740
C	3.3736600	-1.1376550	0.1582040
H	3.0647540	-1.7927710	0.9831650
H	3.2850250	-1.7321480	-0.7612400
H	4.4267610	-0.8876340	0.2989190
O	3.1928610	1.2722050	0.1661840

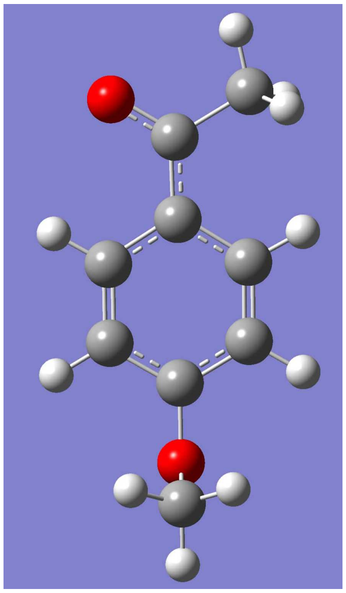

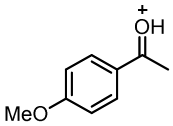

	X	Y	Z
C	1.7178770	0.2953920	0.0009900
C	0.8789130	1.4334930	0.0011120
C	-0.4827530	1.2832870	-0.0097360
C	-1.0700010	-0.0125930	-0.0156300
C	-0.2115000	-1.1442470	-0.0136450
C	1.1556620	-1.0008020	-0.0069710
H	1.3364950	2.4141590	0.0077640
H	-1.1055100	2.1674270	-0.0069460
H	-0.6403670	-2.1372780	-0.0182430
H	1.7859550	-1.8782020	-0.0075230
O	3.0234390	0.5441140	0.0076000
C	3.9720920	-0.5434270	0.0065990
H	3.8564540	-1.1488900	-0.8934910
H	4.9487880	-0.0679770	0.0117790
H	3.8502540	-1.1556500	0.9012650
C	-2.4739850	-0.1764550	-0.0104100
C	-3.4574530	0.9401660	0.0029640
H	-3.5232680	1.3401760	1.0209380
H	-3.1533660	1.7517350	-0.6559090
H	-4.4491020	0.5961520	-0.2927980
O	-2.9220030	-1.4044210	0.0163670
H	-3.8909290	-1.4480800	0.0097910

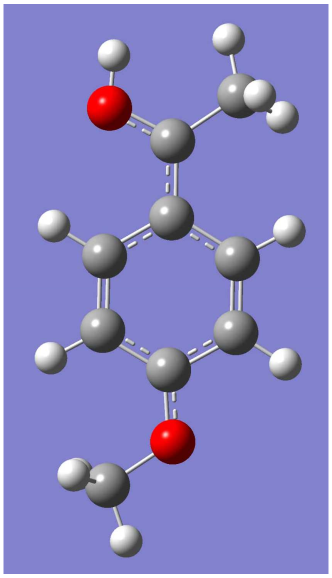

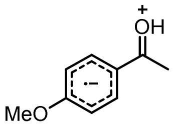

	X	Y	Z
C	1.7460910	0.2963610	-0.0004190
C	0.9112640	1.4281830	-0.0071650
C	-0.4619090	1.2954070	-0.0047240
C	-1.0884730	0.0117030	0.0090550
C	-0.2179350	-1.1161170	0.0107760
C	1.1649500	-0.9774610	0.0078000
H	1.3676340	2.4116560	-0.0176710
H	-1.0652180	2.1942930	-0.0169120
H	-0.6444110	-2.1104270	0.0183220
H	1.7794900	-1.8681930	0.0115990
O	3.0929780	0.5426330	-0.0045880
C	3.9866920	-0.5705640	0.0003920
H	3.8504040	-1.1925160	-0.8895380
H	4.9894740	-0.1475670	-0.0038950
H	3.8535810	-1.1819260	0.8981060
C	-2.4929390	-0.1461000	0.0168690
C	-3.5259720	0.9288370	0.0075160
H	-4.3075380	0.7214670	0.7493780
H	-3.1064610	1.9057140	0.2416270
H	-4.0217520	1.0033640	-0.9705520
O	-2.9565400	-1.4414650	-0.0335750
H	-3.9173320	-1.4466990	0.0442360

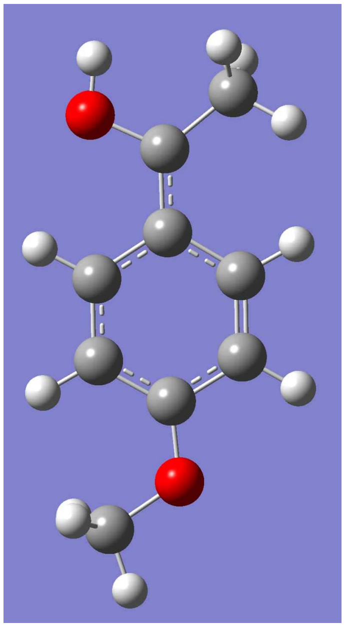

6 NMR Spectra

1a – ^{1}H NMR (400 MHz, $CDCl_3$)

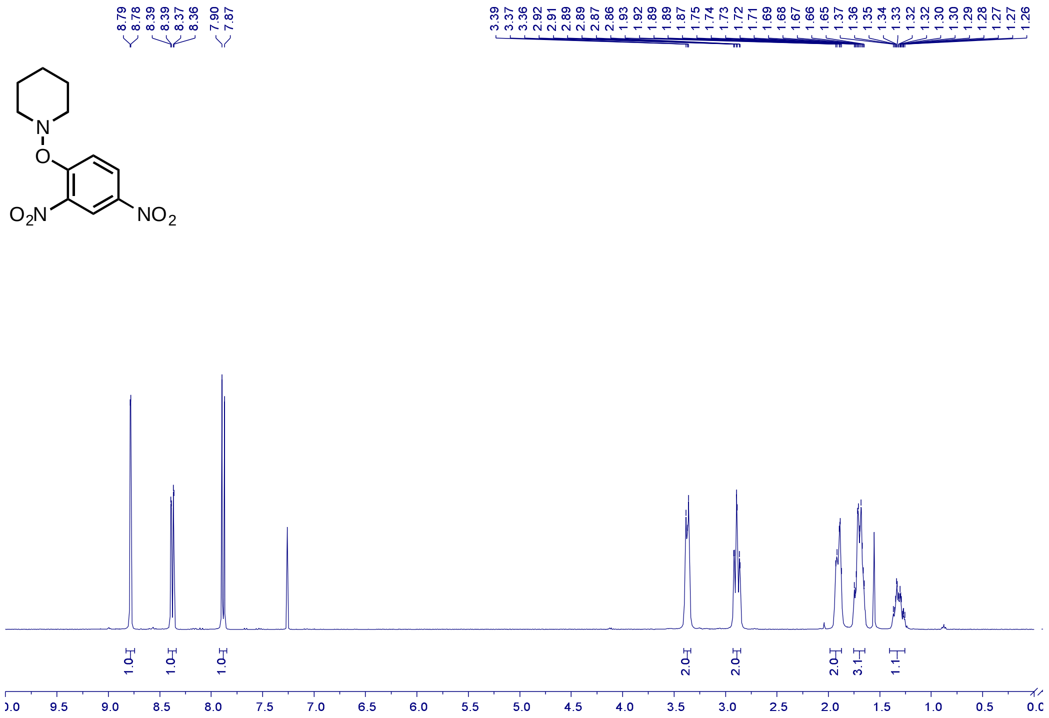

1a – ^{13}C NMR (101 MHz, $CDCl_3$)

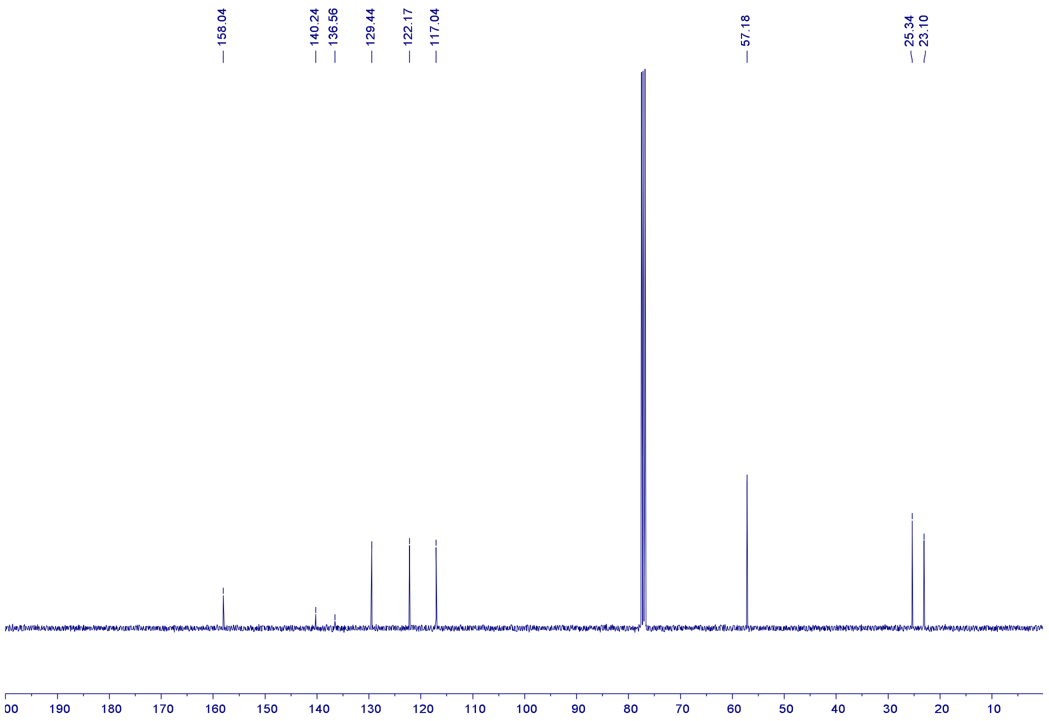

1b – ^{1}H NMR (500 MHz, $CDCl_3$)

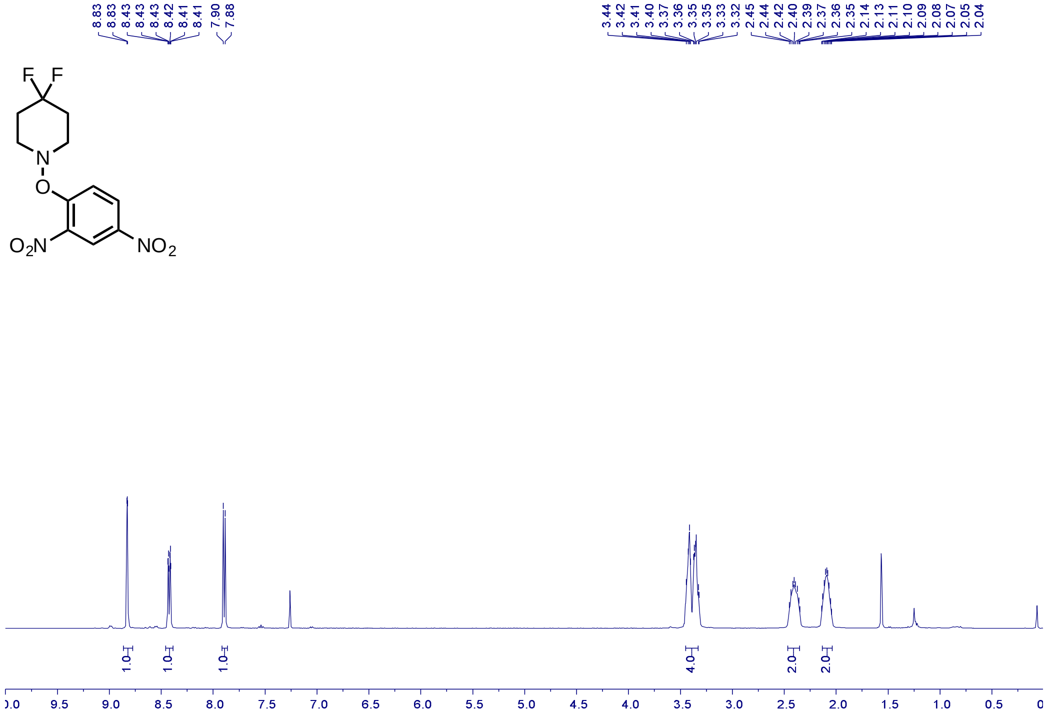

1b – ^{13}C NMR (126 MHz, $CDCl_3$)

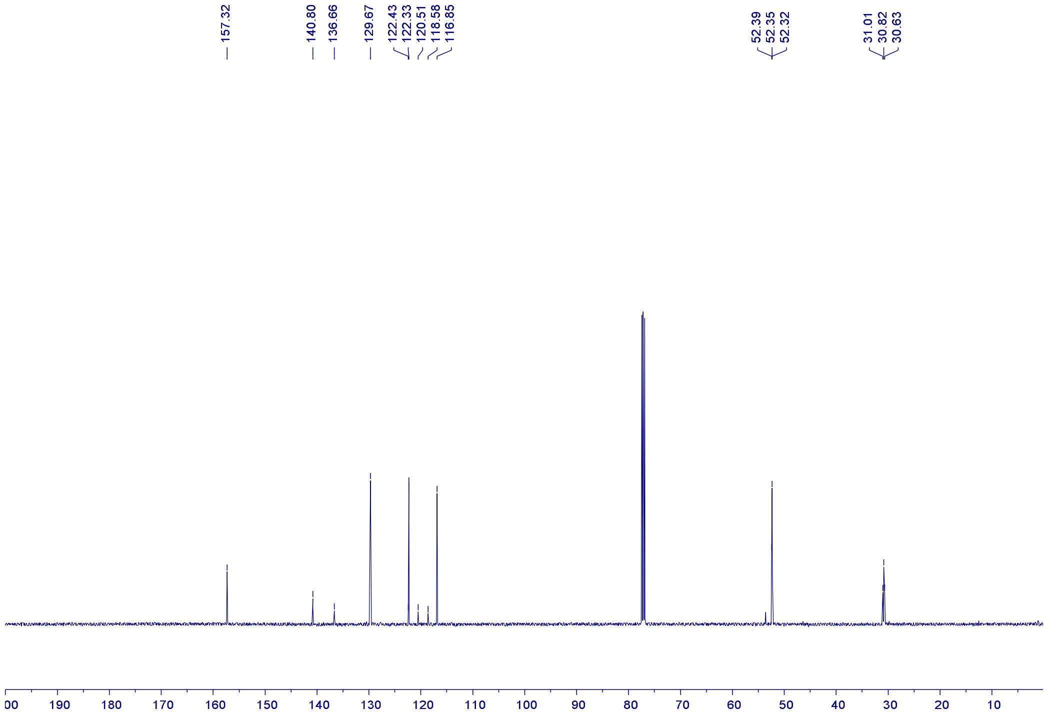

1b – ^{19}F NMR (376 MHz, $CDCl_3$)

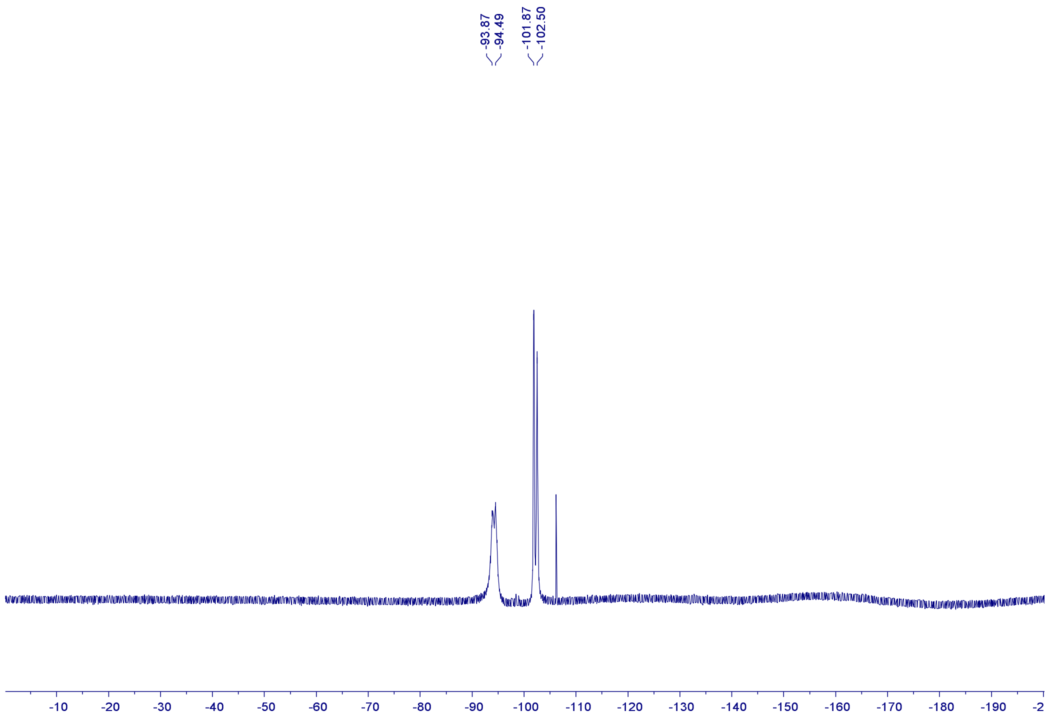

1c – ^{1}H NMR (500 MHz, $CDCl_3$)

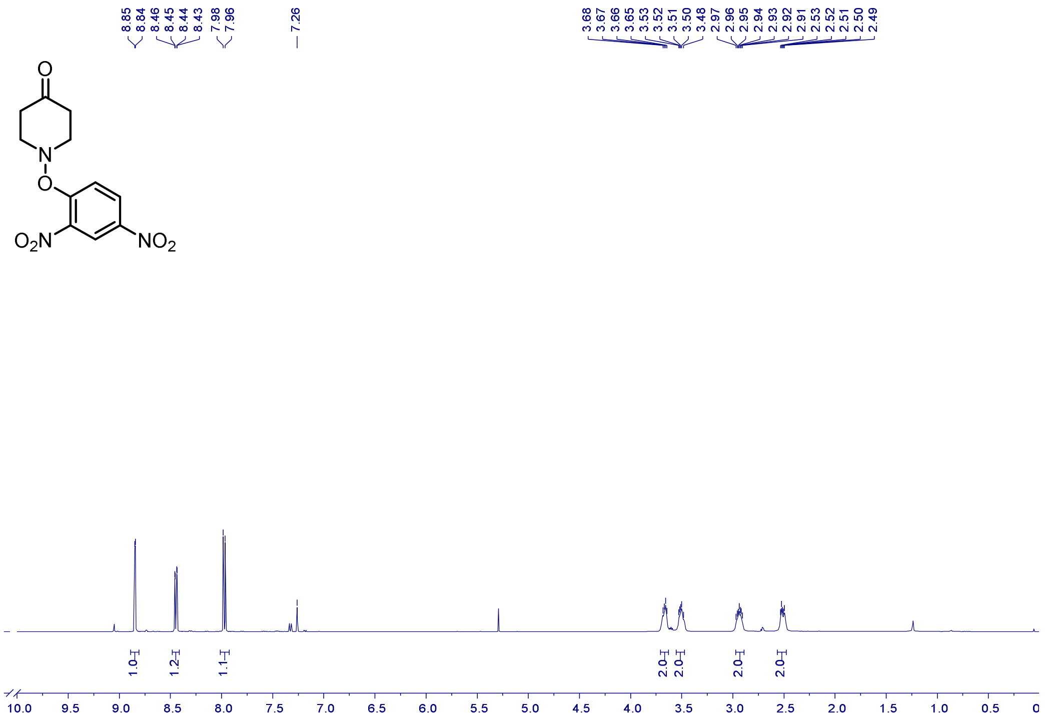

1c – ^{13}C NMR (126 MHz, $CDCl_3$)

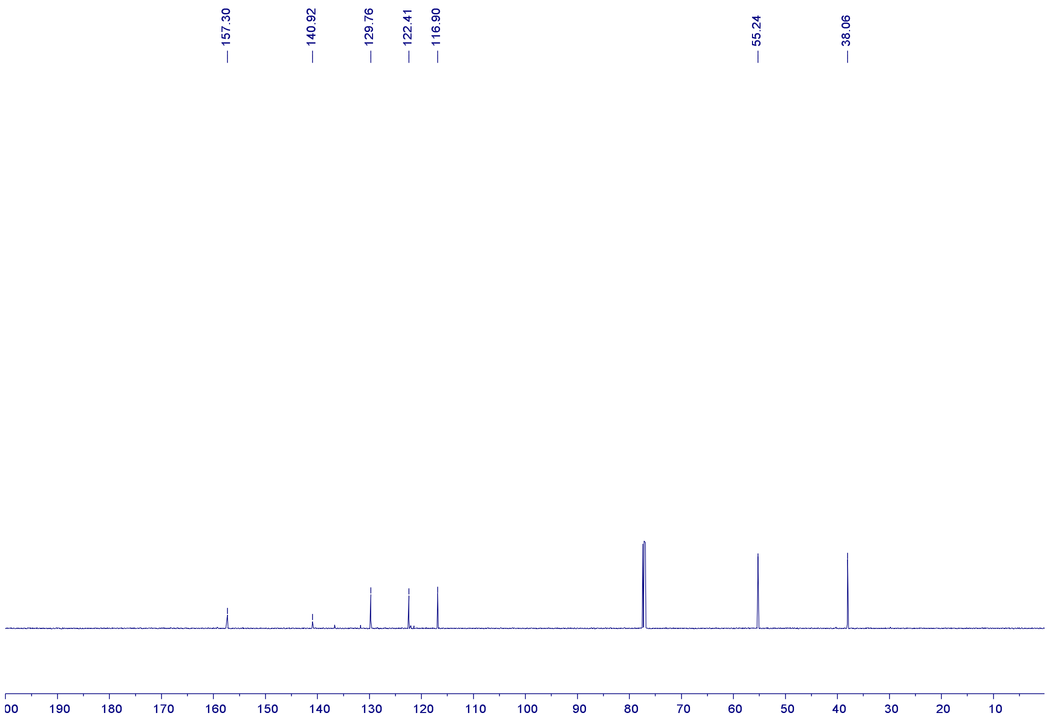

1d – ^{1}H NMR (400 MHz, $CDCl_3$)

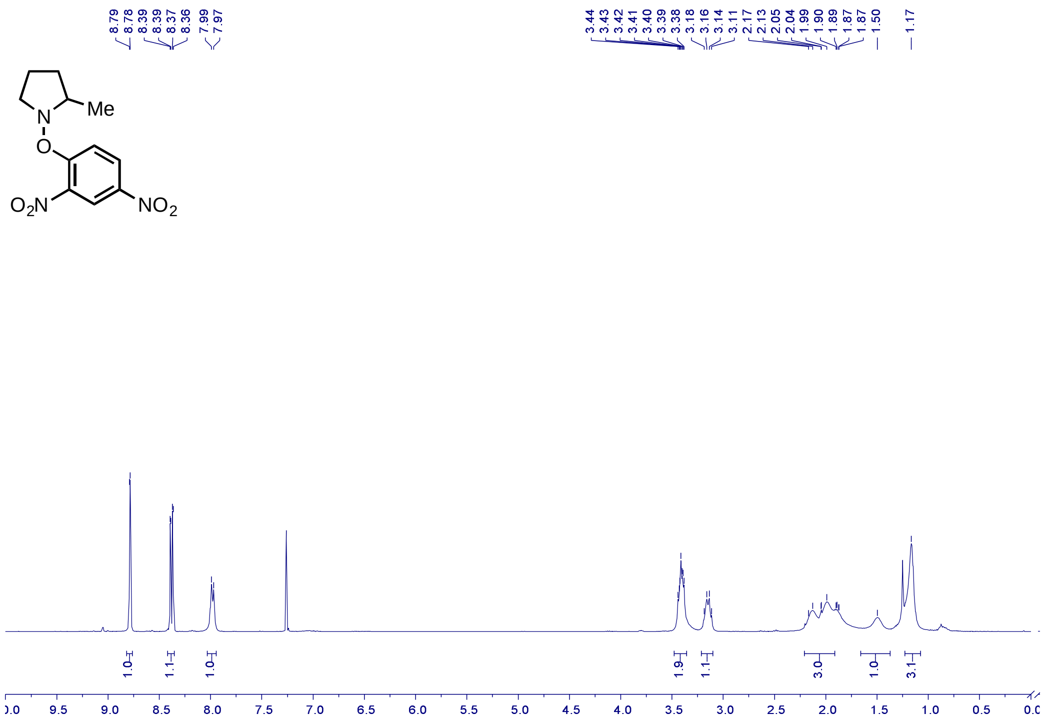

1d – ^{13}C NMR (101 MHz, $CDCl_3$)

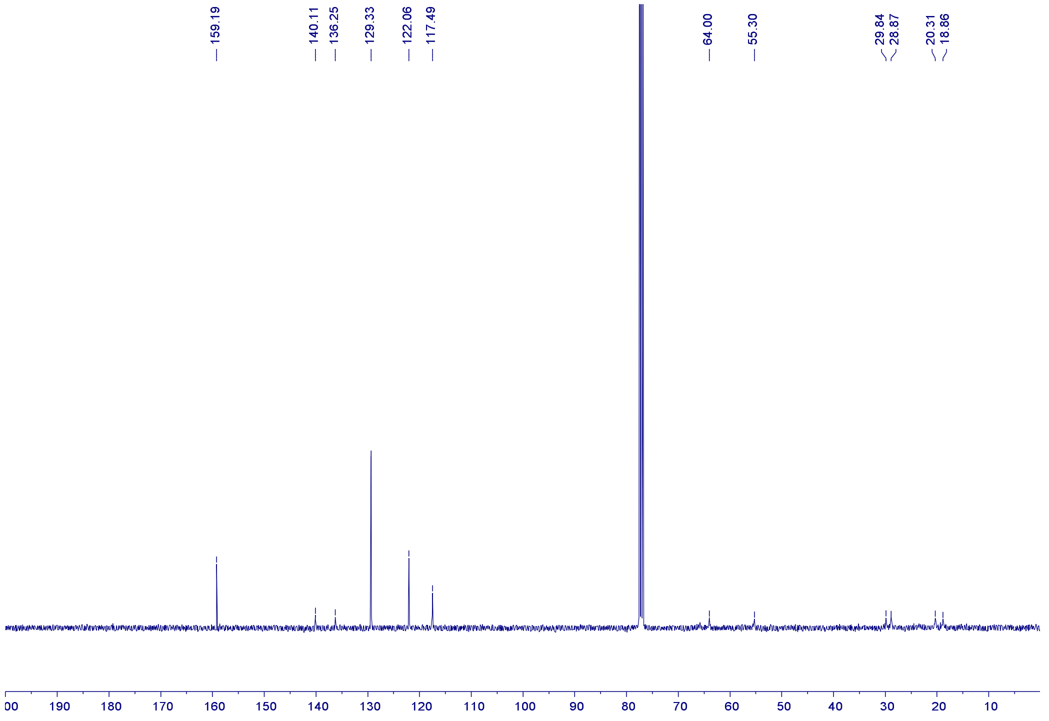

1e – ^{1}H NMR (400 MHz, $CDCl_3$)

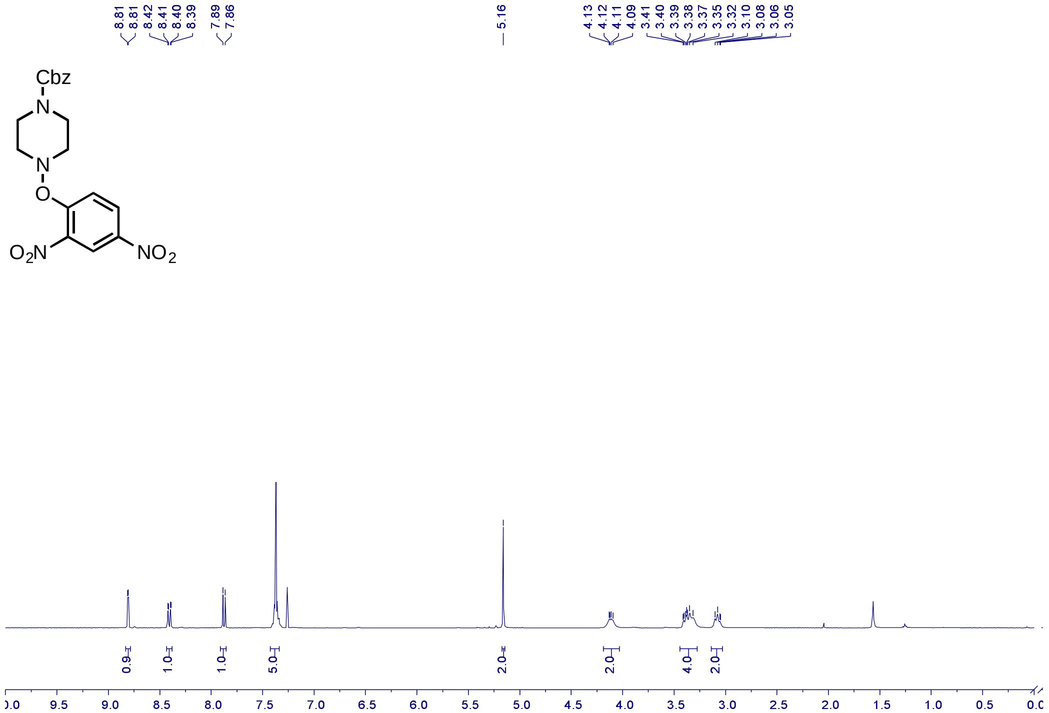

1e – ^{13}C NMR (101 MHz, $CDCl_3$)

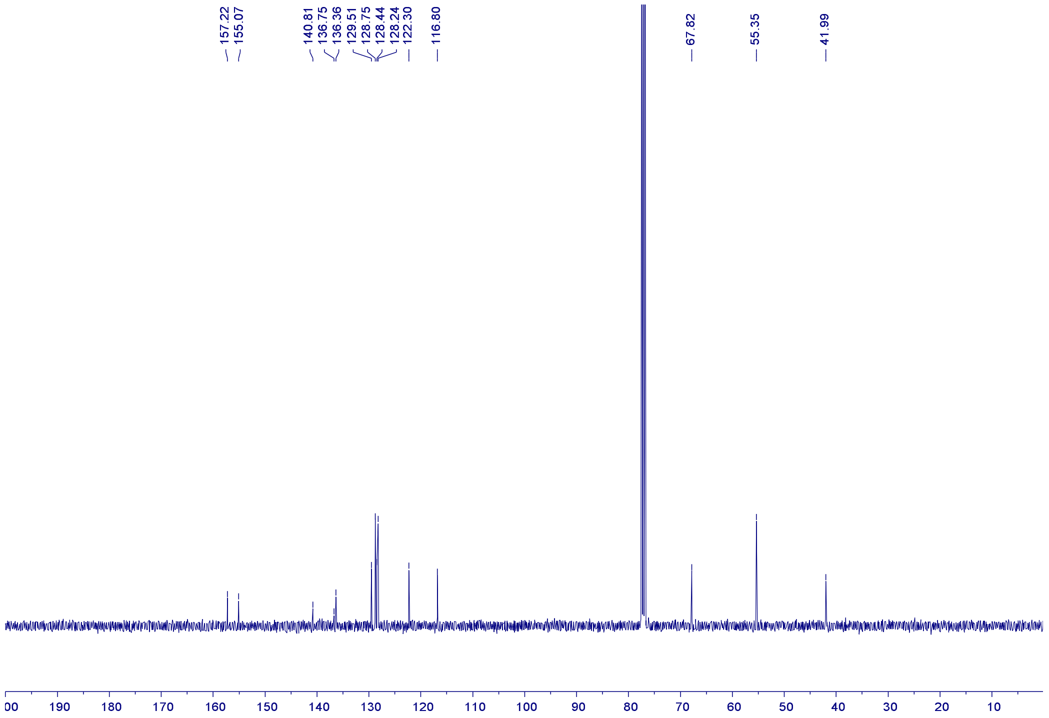

1f – ^{1}H NMR (400 MHz, $CDCl_3$)

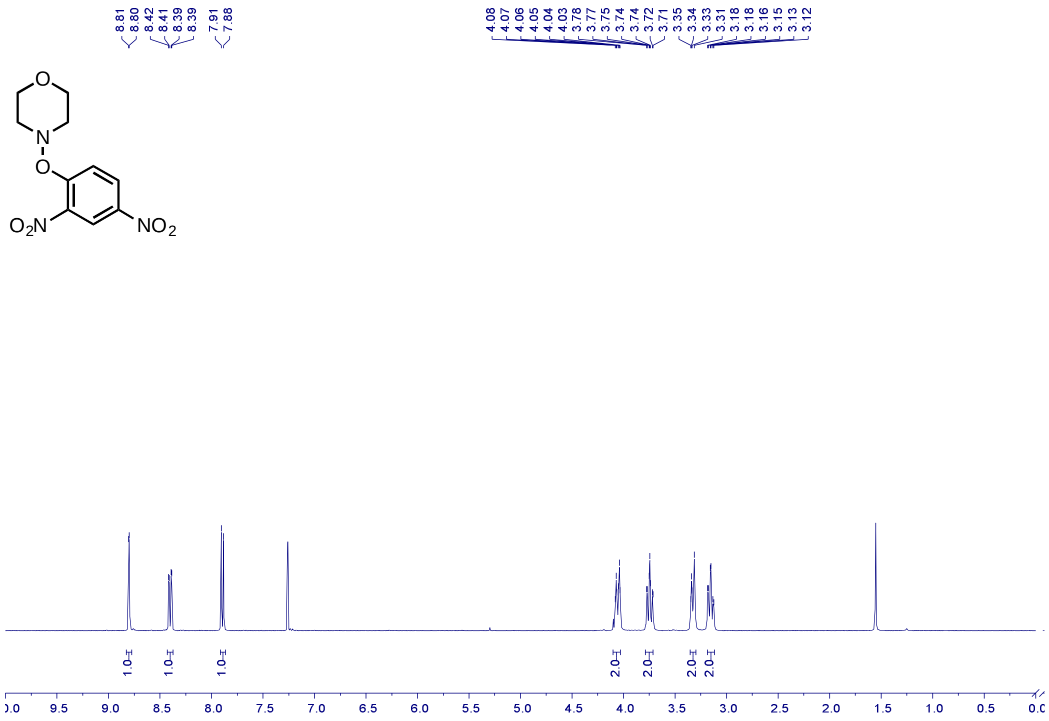

1f – ^{13}C NMR (101 MHz, $CDCl_3$)

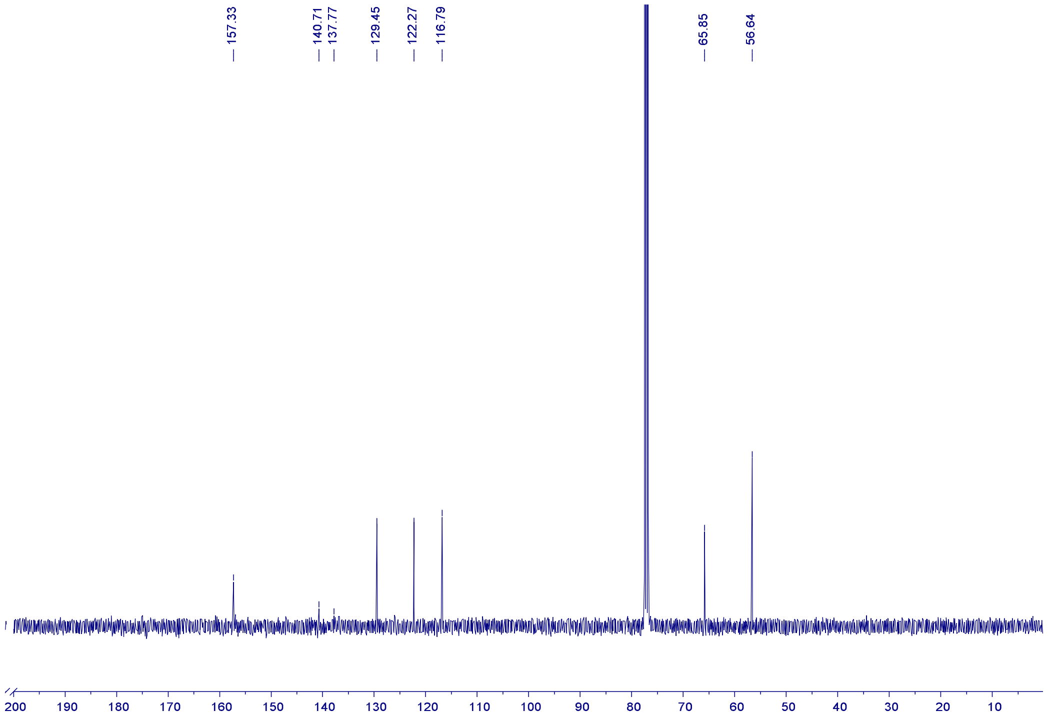

1g – ^{1}H NMR (400 MHz, $CDCl_3$)

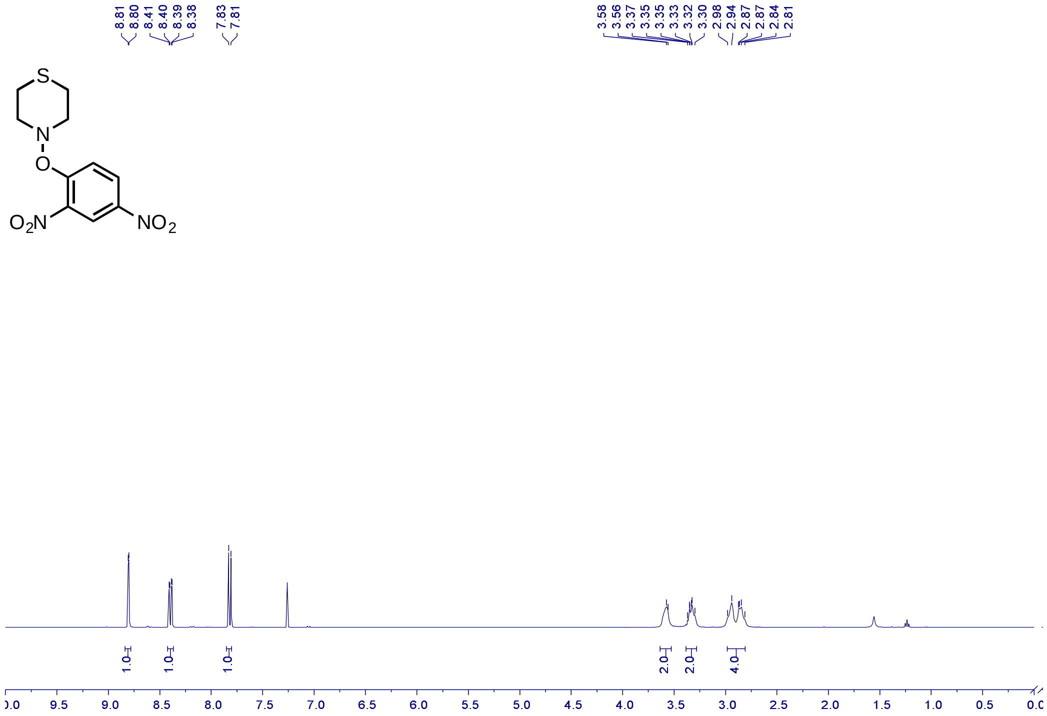

1g – ^{13}C NMR (101 MHz, $CDCl_3$)

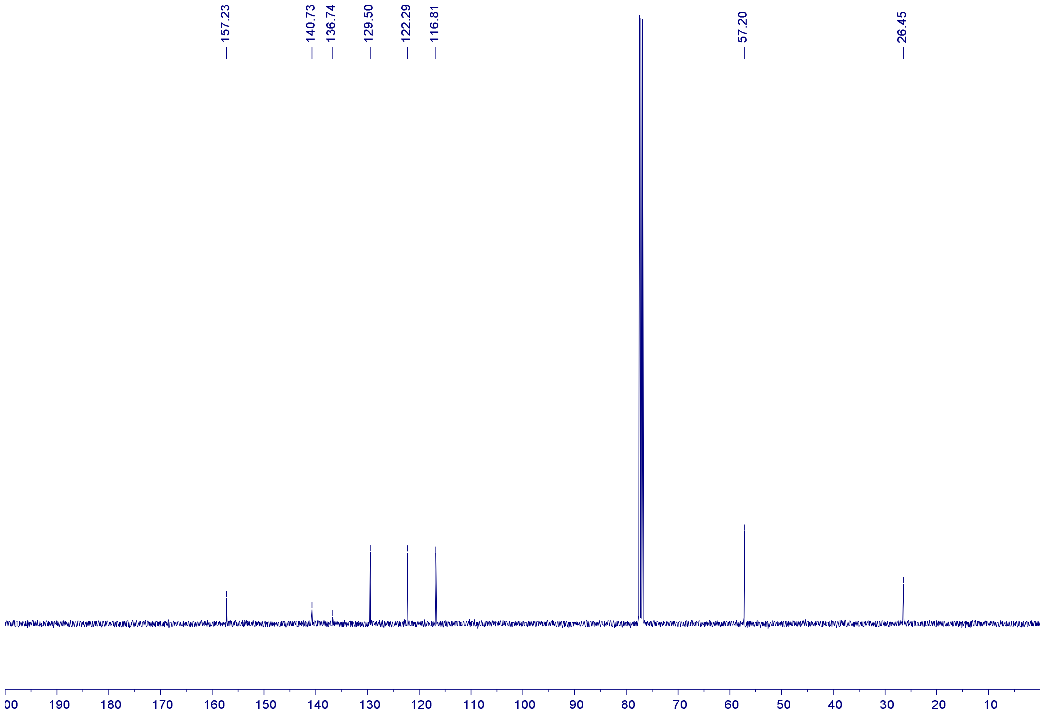

1h – ^{1}H NMR (400 MHz, $CDCl_3$)

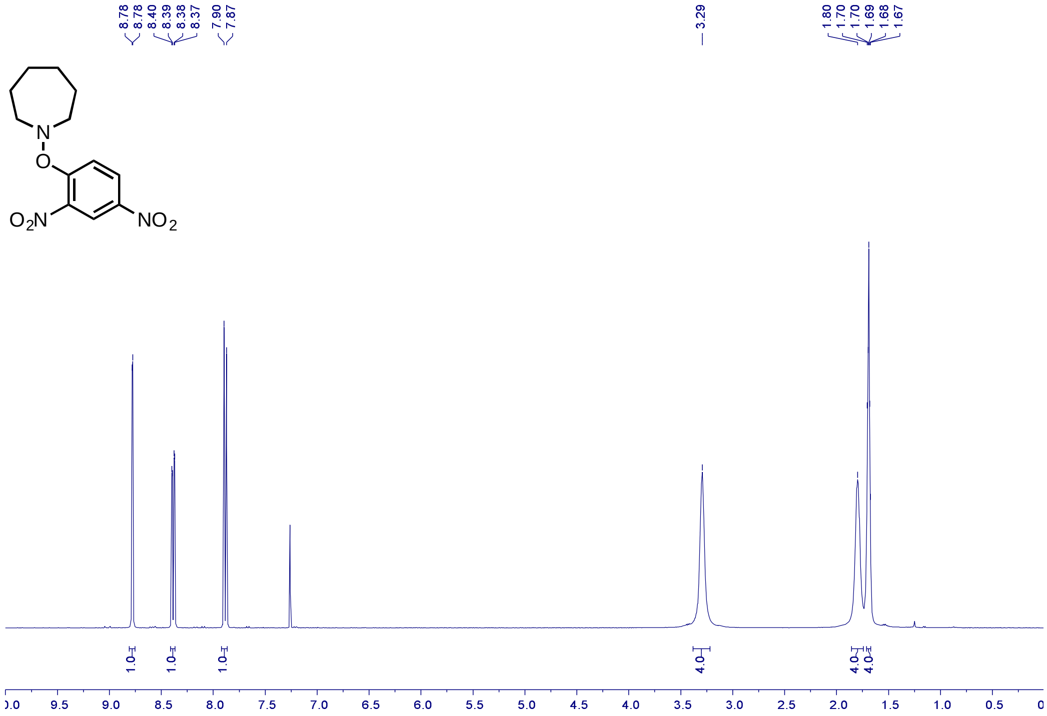

1h – ^{13}C NMR (101 MHz, $CDCl_3$)

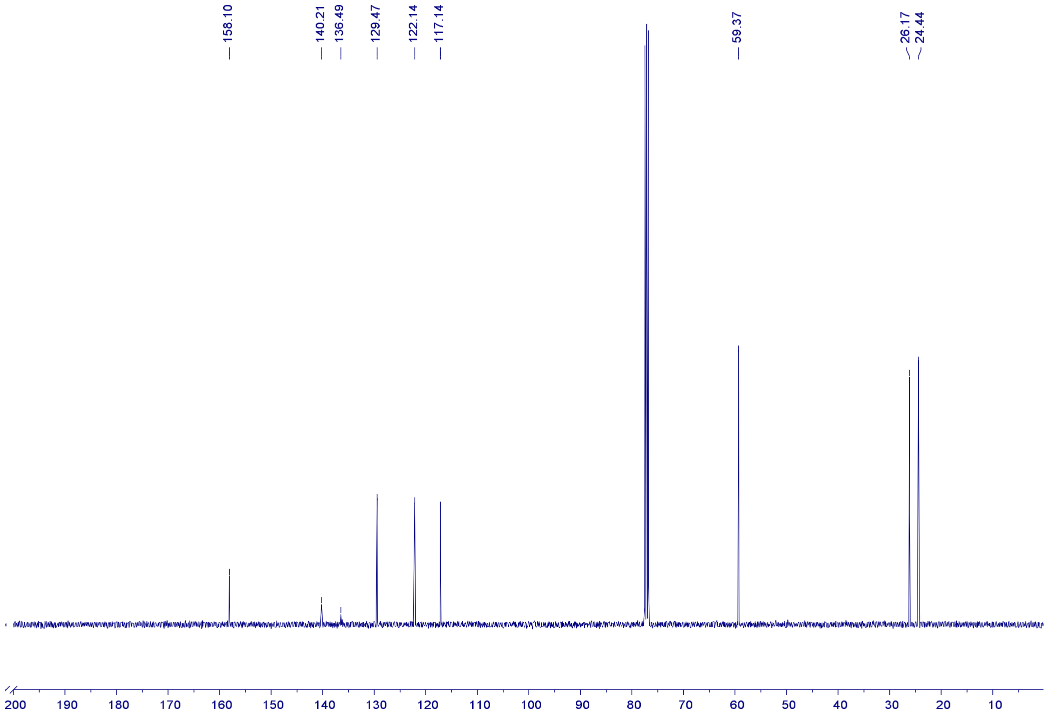

1i – ^{1}H NMR (400 MHz, $CDCl_3$)

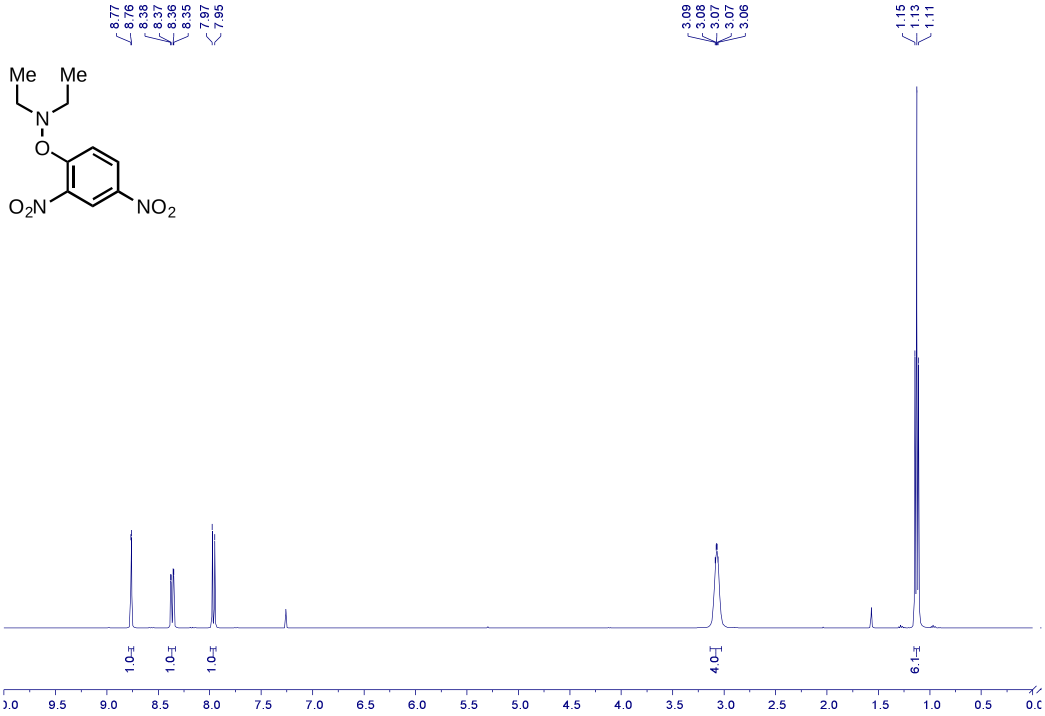

1i – ^{13}C NMR (101 MHz, $CDCl_3$)

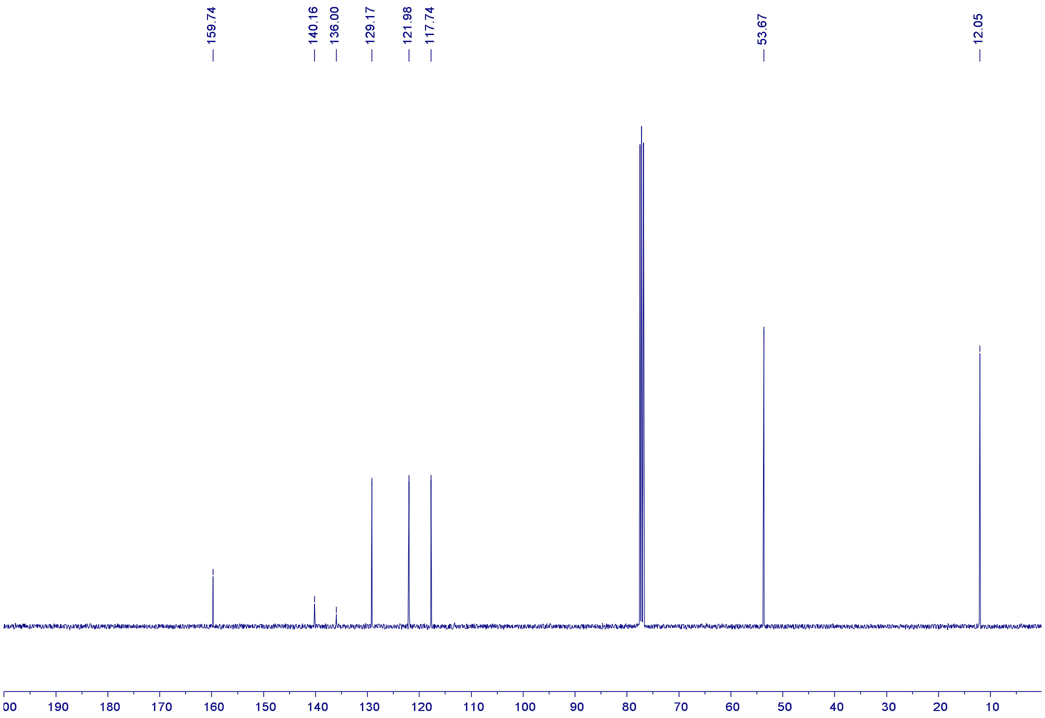

1j – ^{1}H NMR (400 MHz, $CDCl_3$)

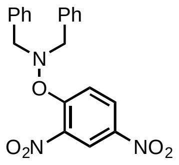

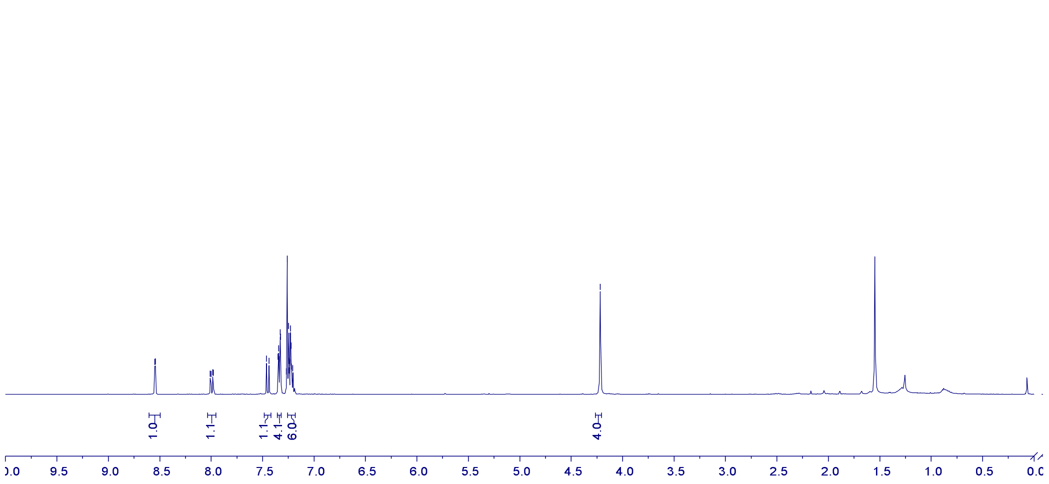

1k– ^{13}C NMR (101 MHz, $CDCl_3$)

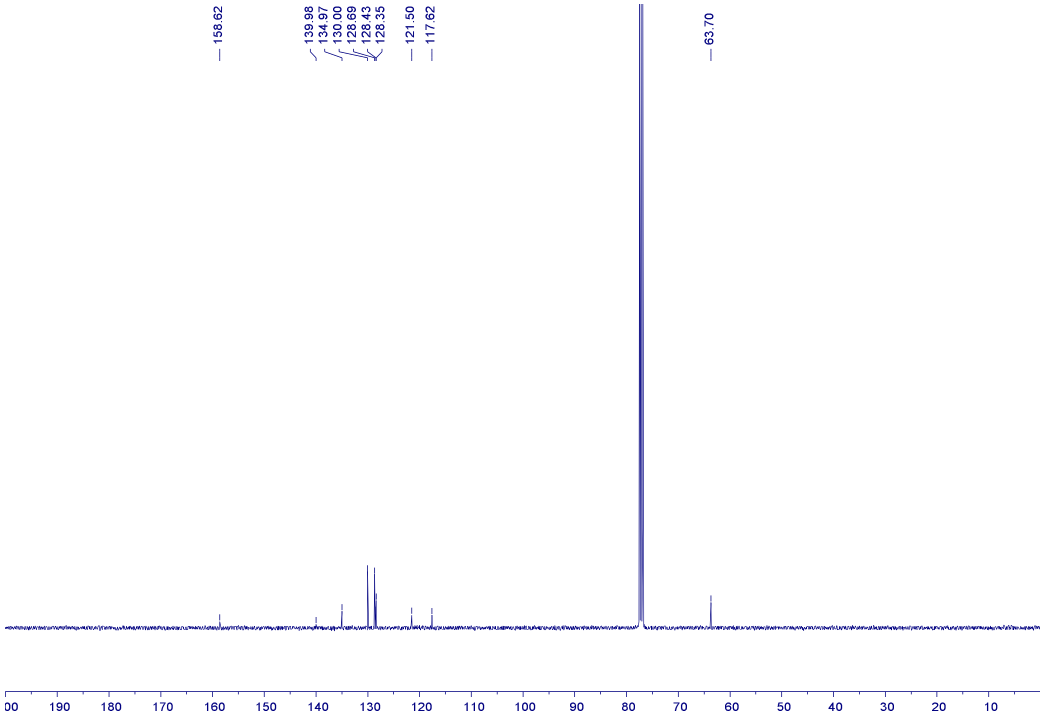

1k – ^{1}H NMR (500 MHz, $CDCl_3$)

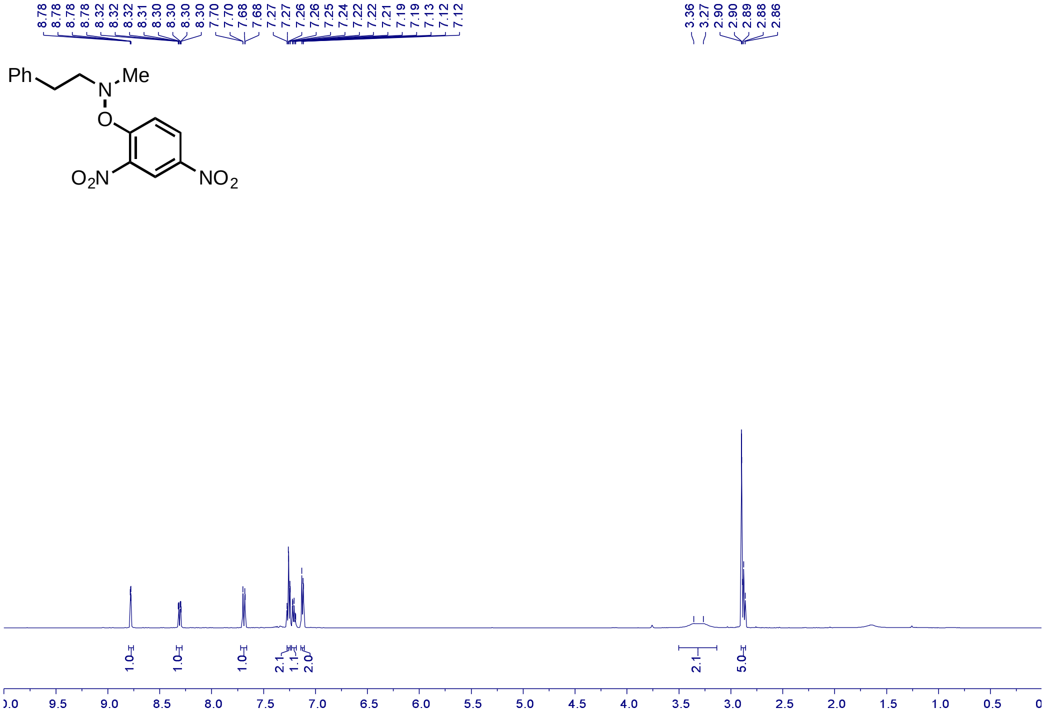

1k – ^{13}C NMR (126 MHz, $CDCl_3$)

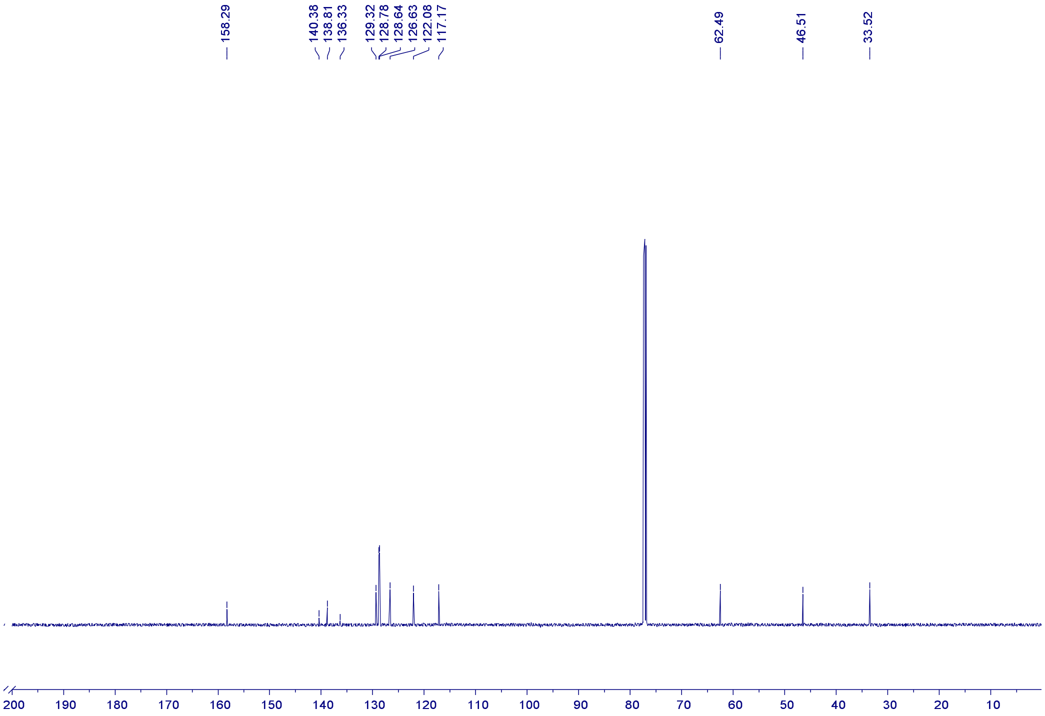

1l – ^{1}H NMR (500 MHz, $CDCl_3$)

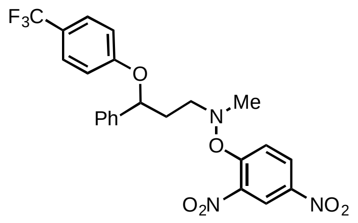

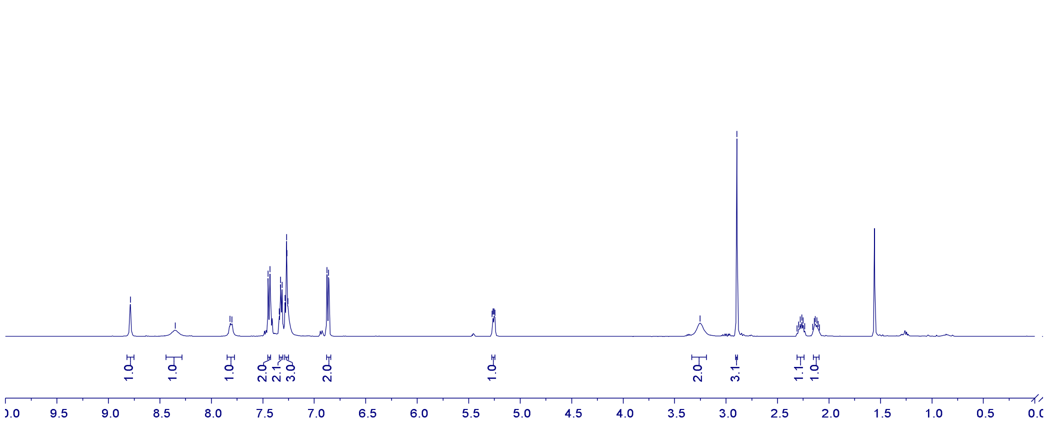

1l – ^{13}C NMR (126 MHz, $CDCl_3$)

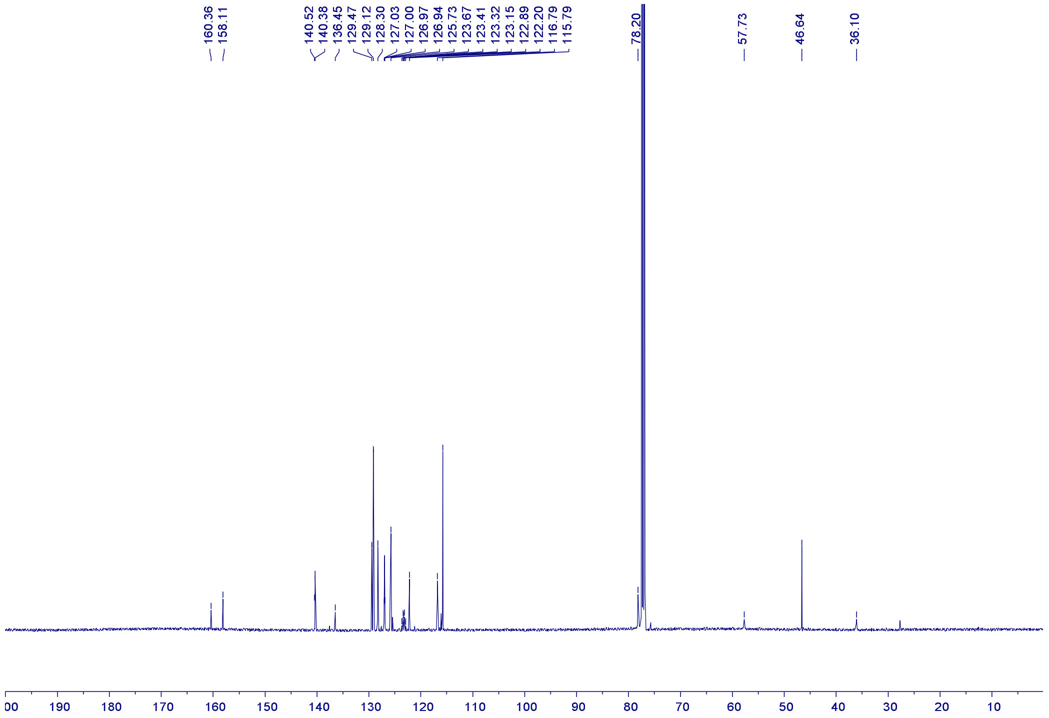

1l – ^{19}F NMR (376 MHz, $CDCl_3$)

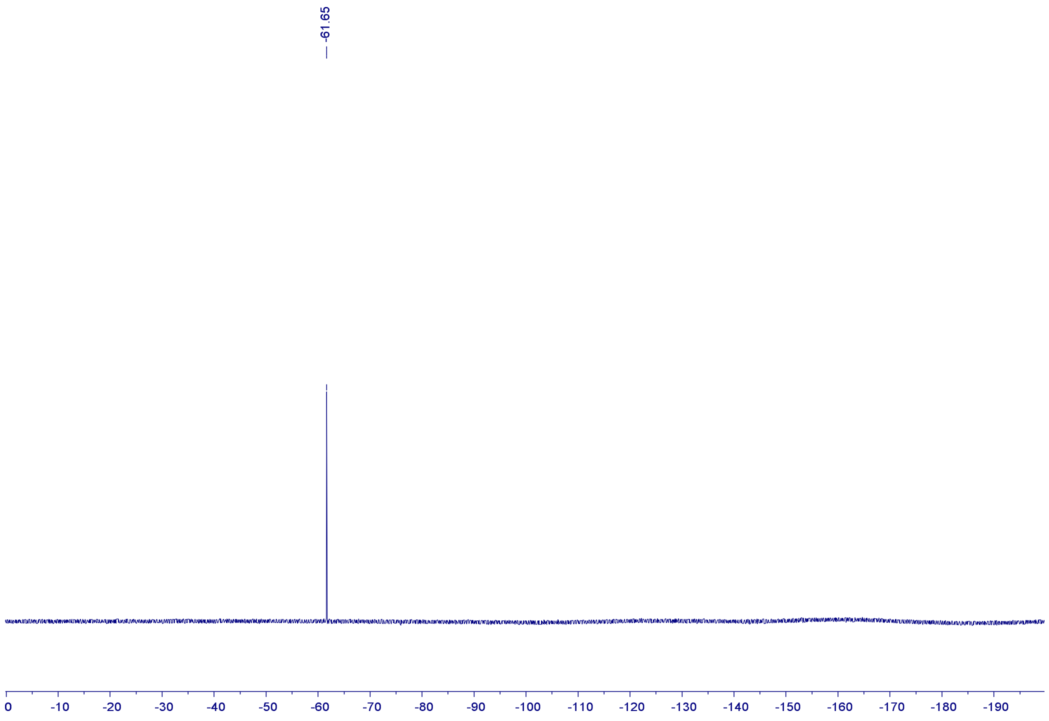

1m – ^{1}H NMR (500 MHz, $CDCl_3$)

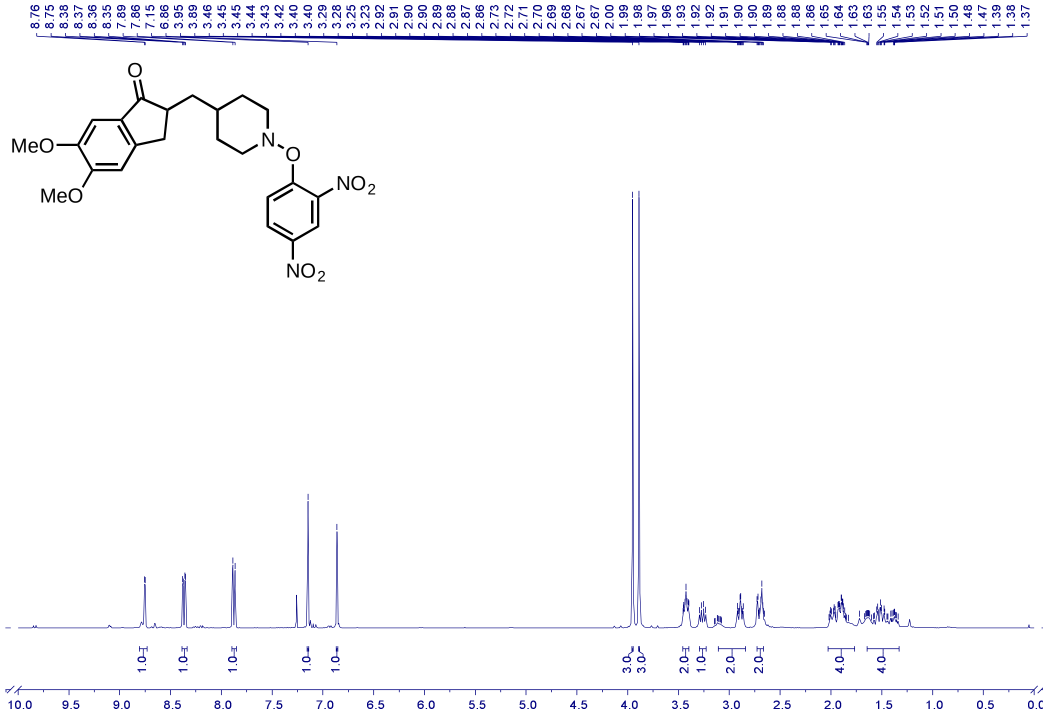

1m – ^{13}C NMR (126 MHz, $CDCl_3$)

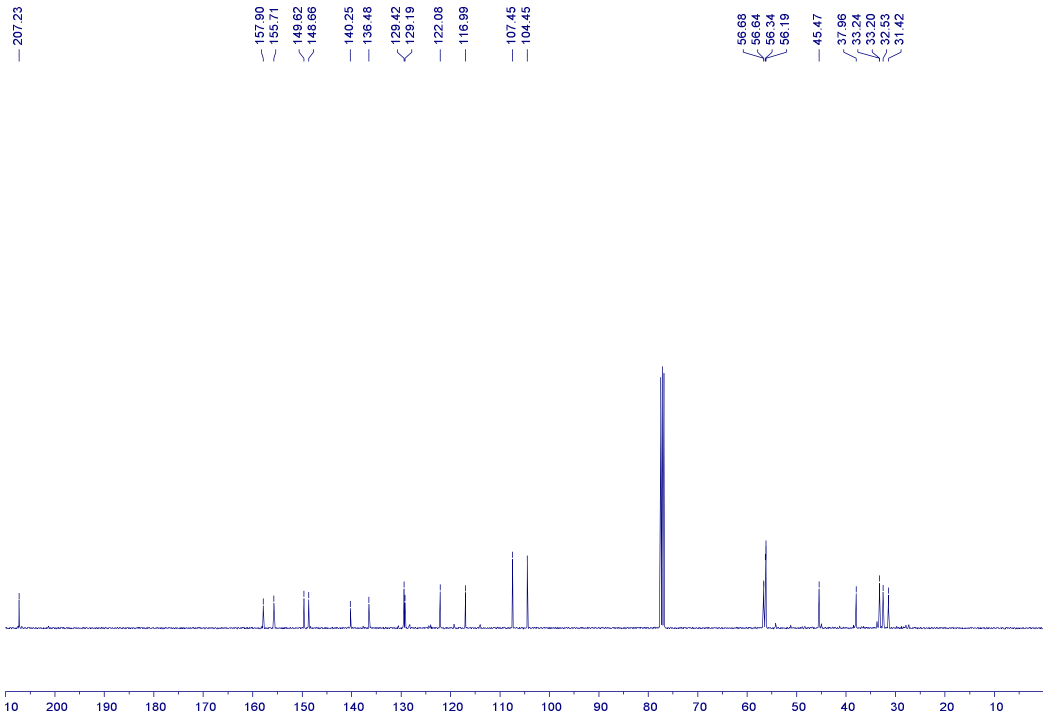

S8 – ^{1}H NMR (500 MHz, $CDCl_3$)

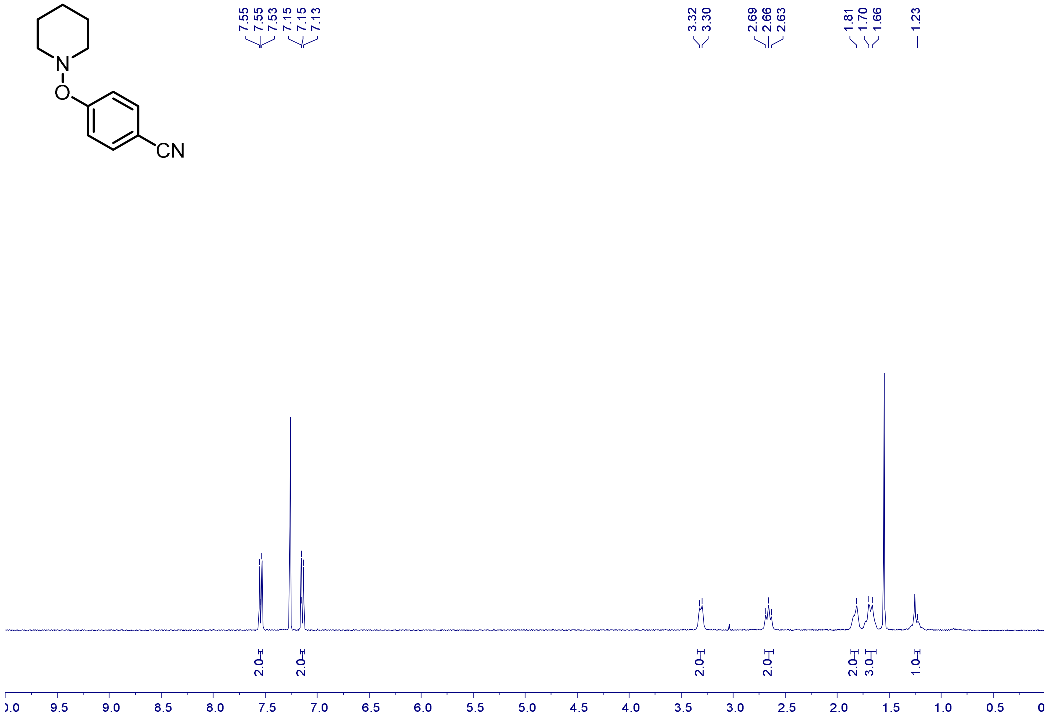

S8 – ^{13}C NMR (126 MHz, $CDCl_3$)

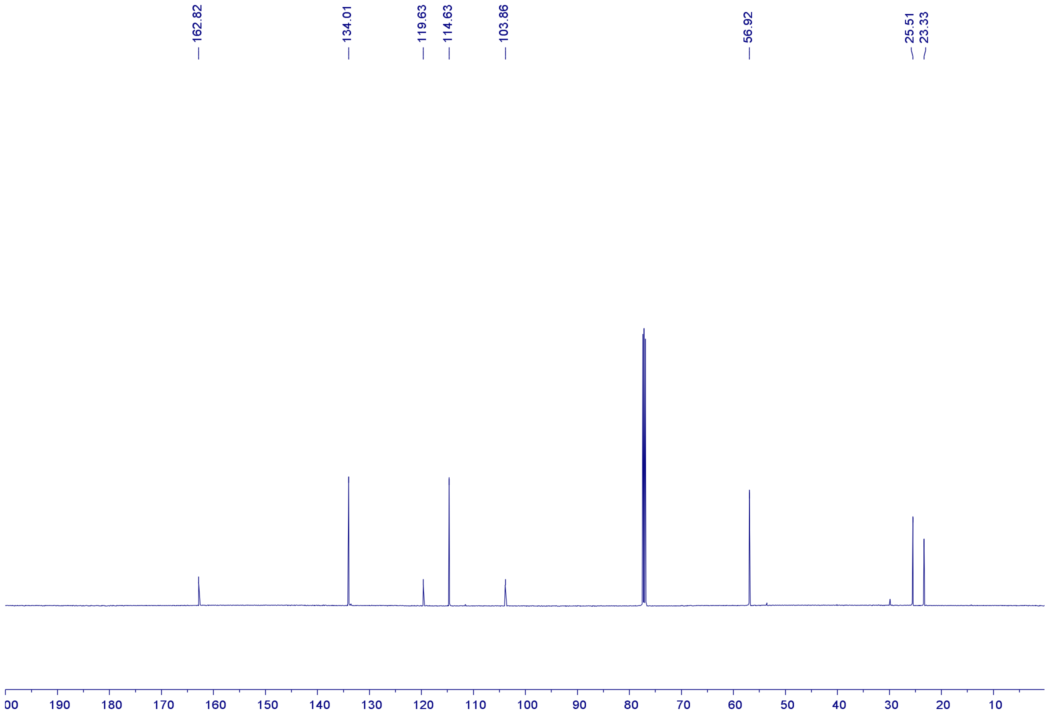

3d and 3d' – ^{1}H NMR (500 MHz, $CDCl_3$)

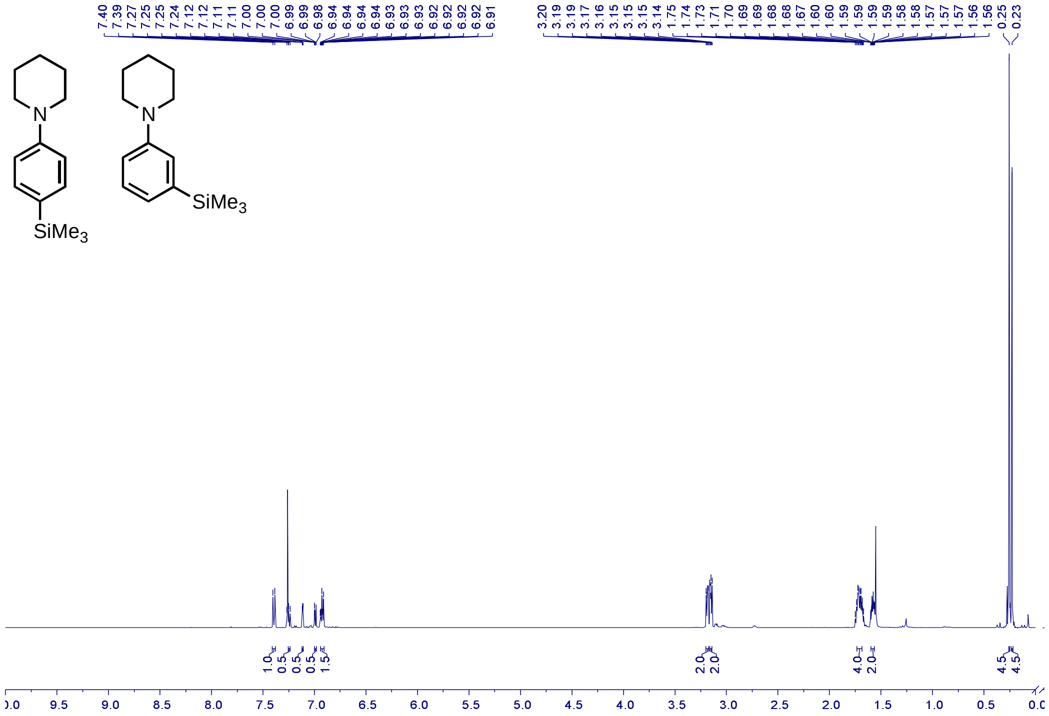

3d and 3d' – ^{13}C NMR (126 MHz, $CDCl_3$)

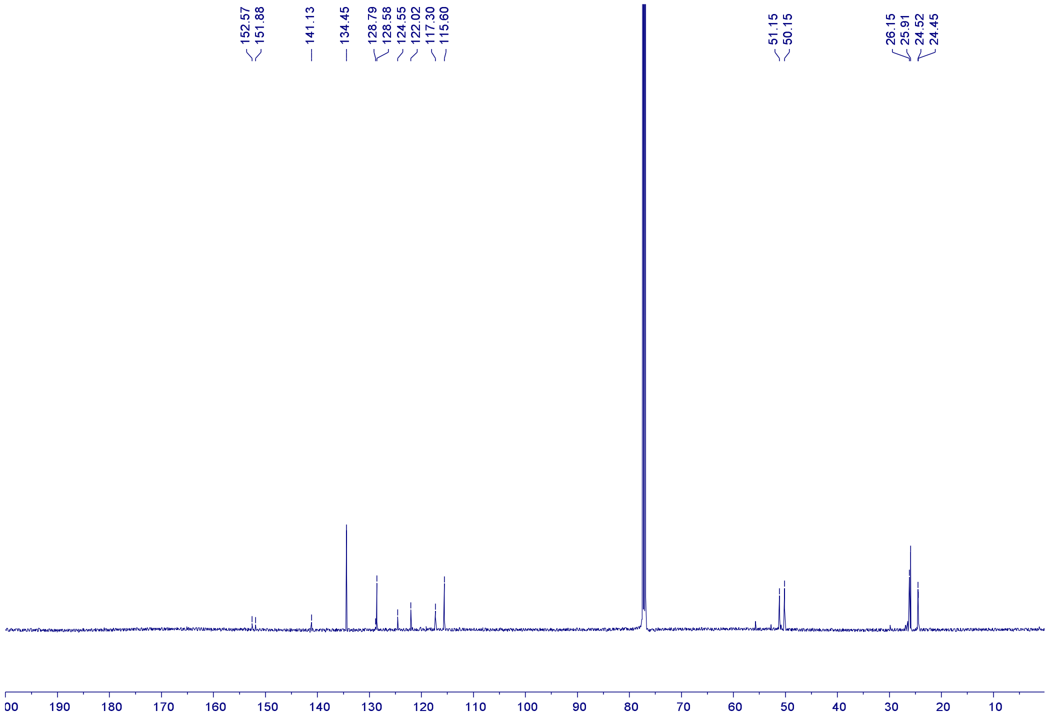

3e – ^{1}H NMR (500 MHz, $CDCl_3$)

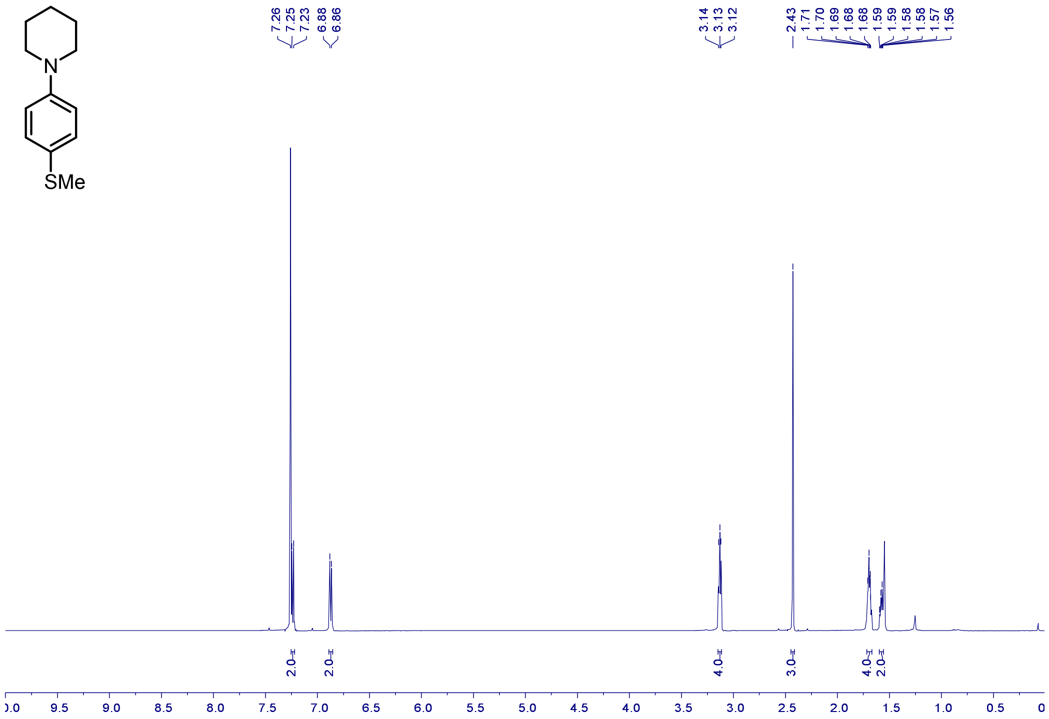

3e – ^{13}C NMR (126 MHz, $CDCl_3$)

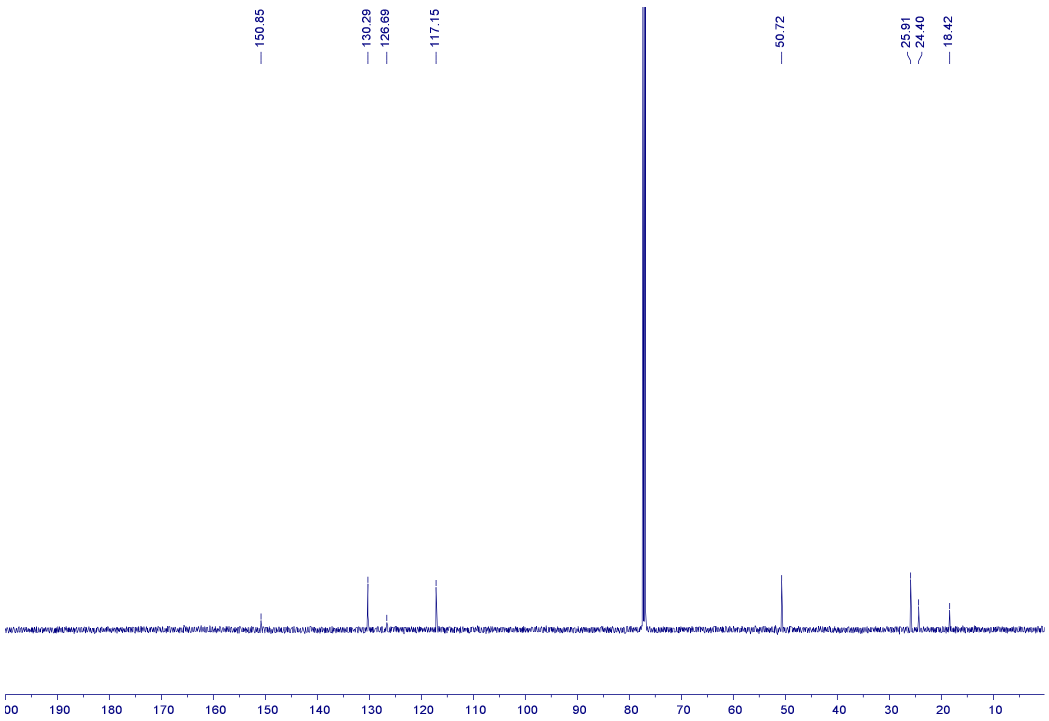

3g – ^{1}H NMR (500 MHz, $CDCl_3$)

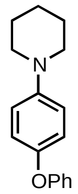

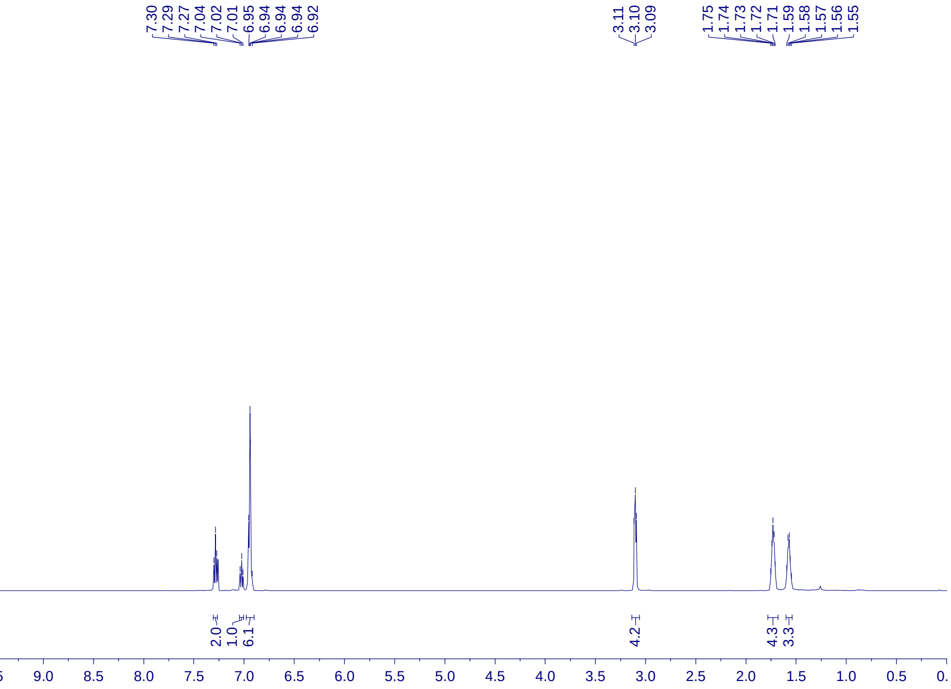

3g – ^{13}C NMR (126 MHz, $CDCl_3$)

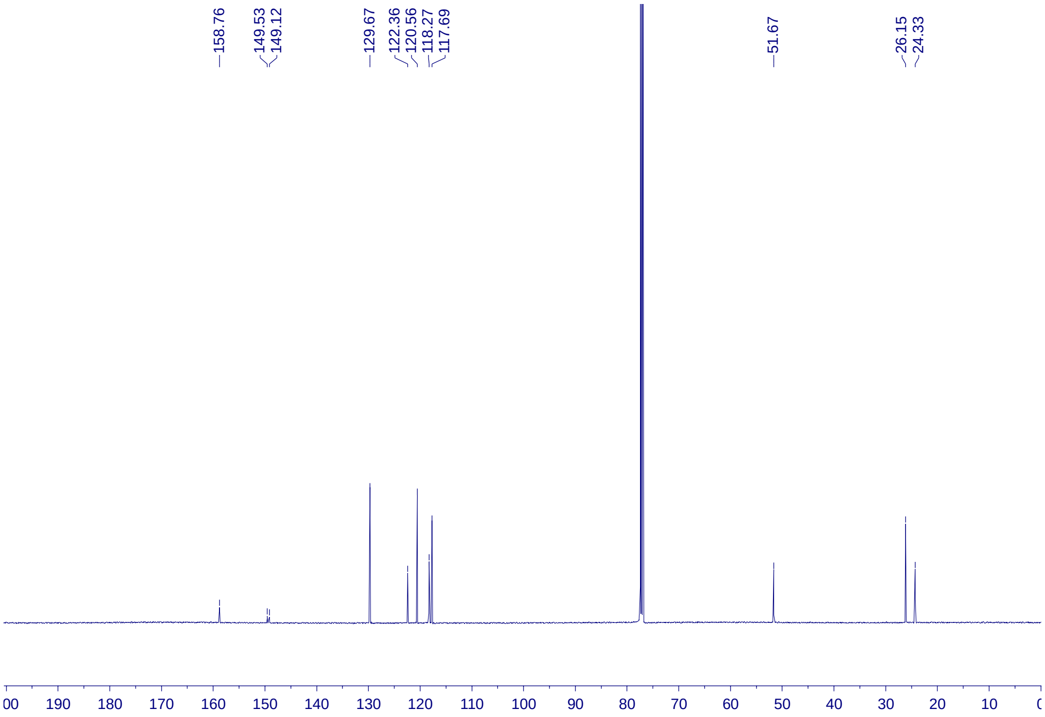

3g’ – ^{1}H NMR (500 MHz, CD_3OD)

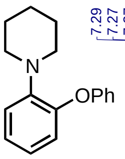

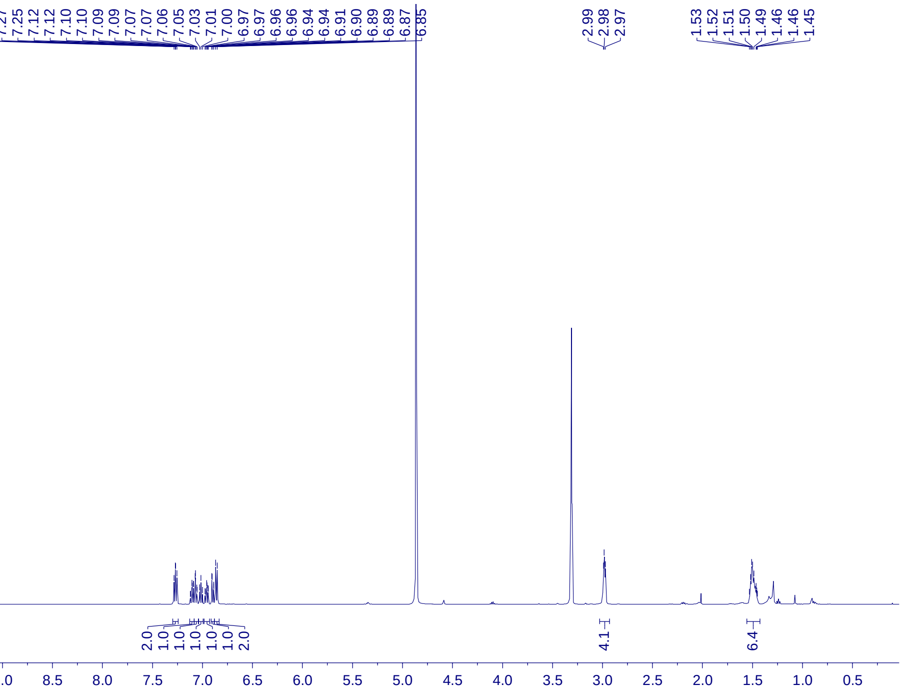

3g’ – ^{13}C NMR (126 MHz, CD_3OD)

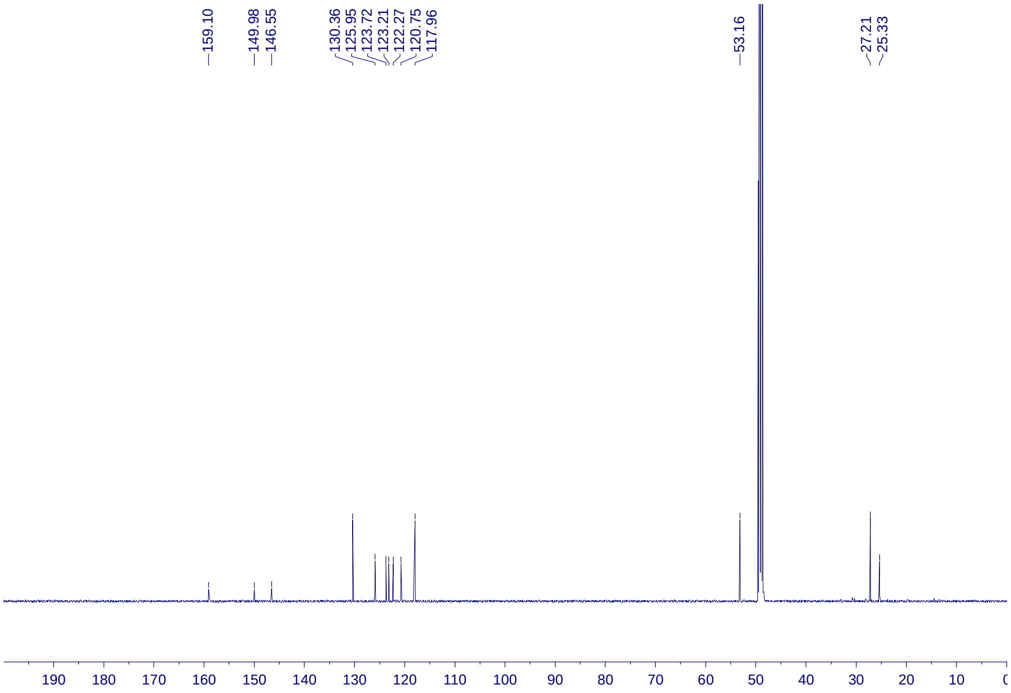

3h – ^{1}H NMR (500 MHz, $CDCl_3$)

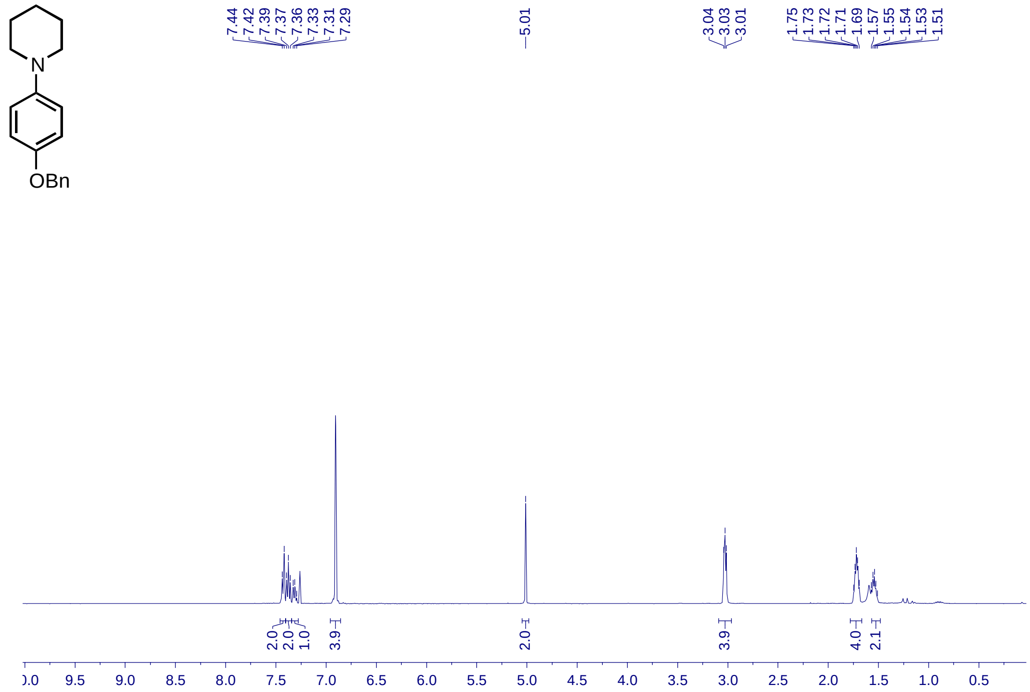

3h – ^{13}C NMR (126 MHz, $CDCl_3$)

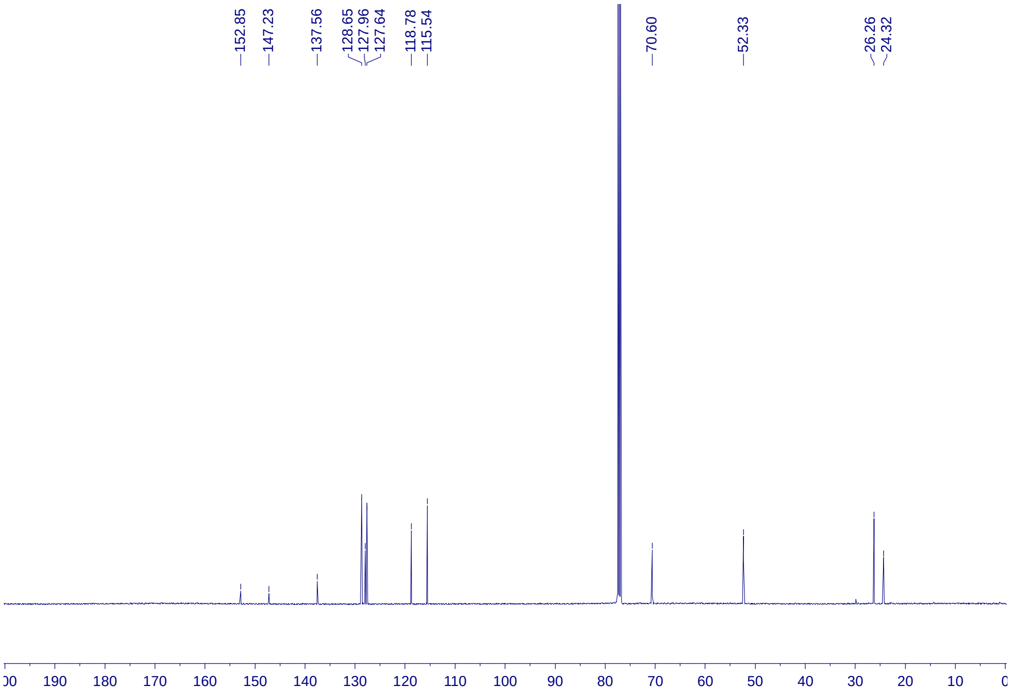

3h' – ^{1}H NMR (500 MHz, CD_3OD)

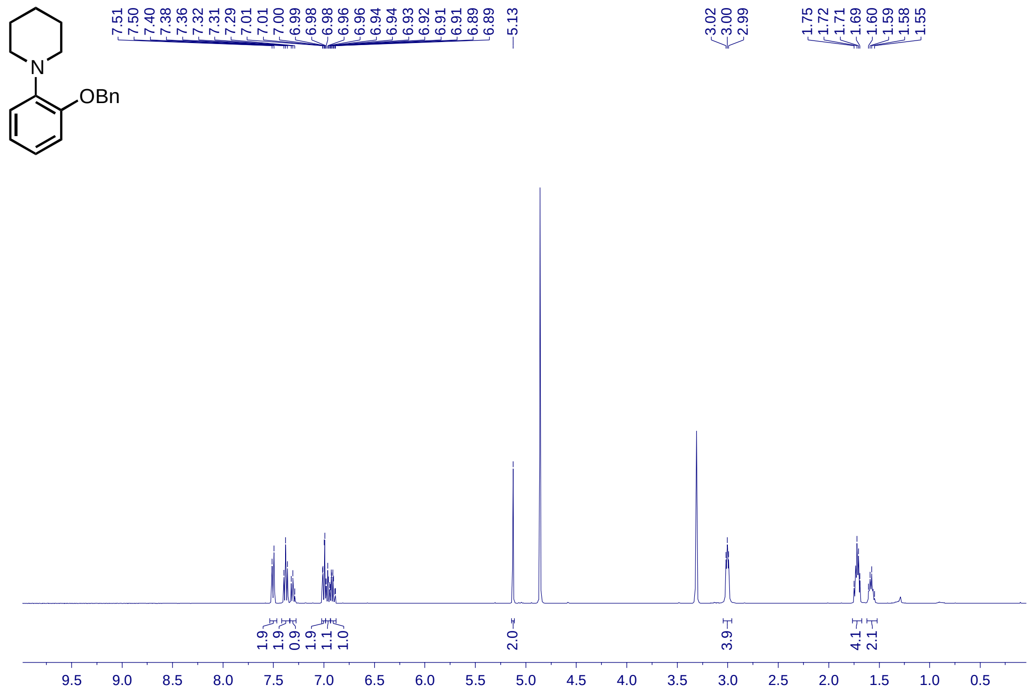

3h' – ^{13}C NMR (126 MHz, CD_3OD)

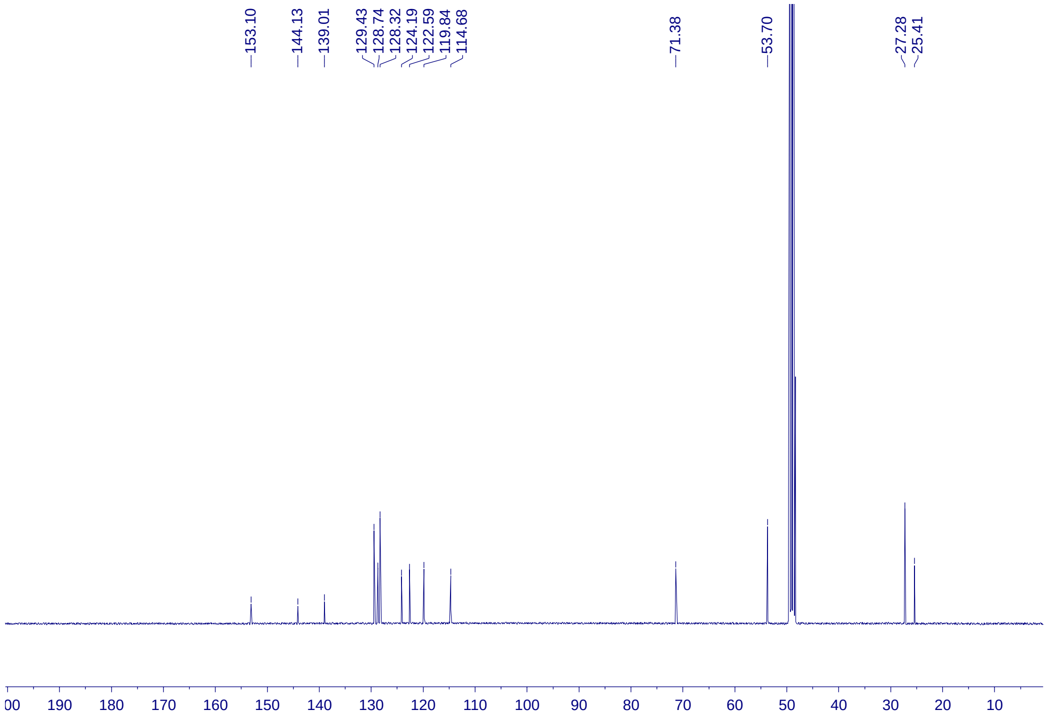

3k – ^{1}H NMR (500 MHz, $CDCl_3$)

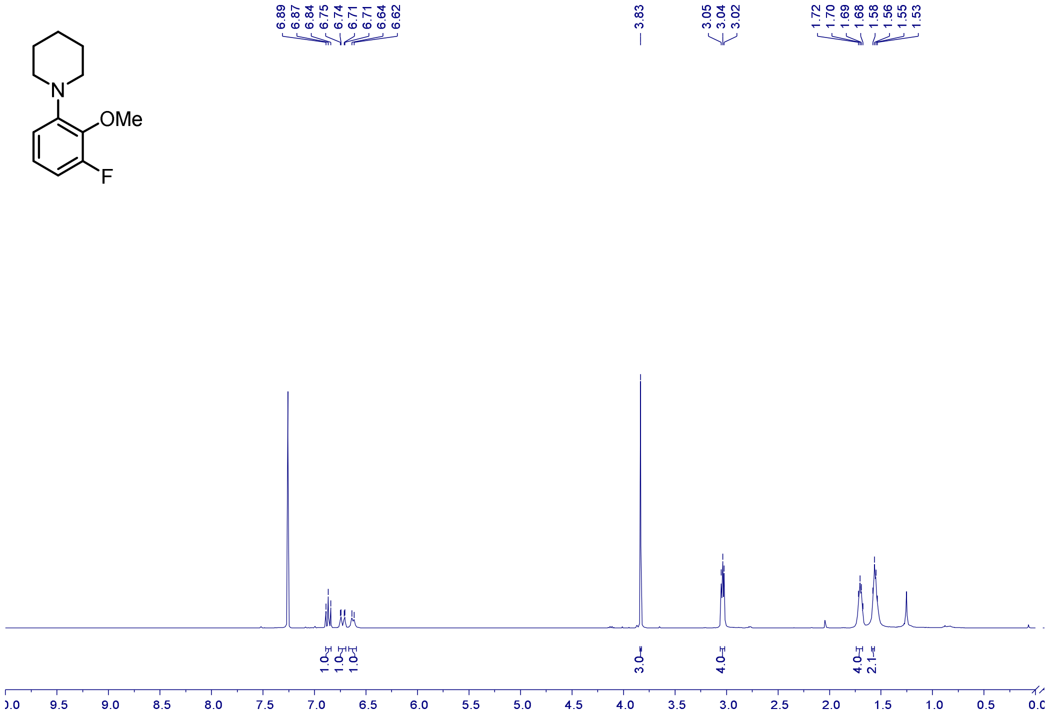

3k – ^{13}C NMR (126 MHz, $CDCl_3$)

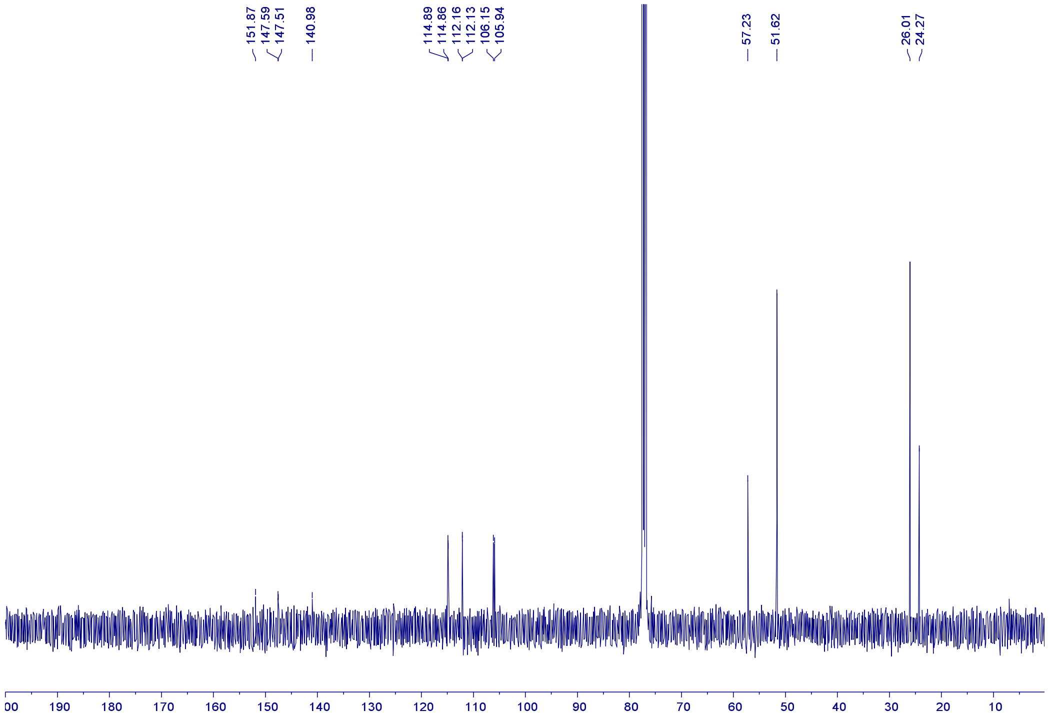

3k – ^{19}F NMR (376 MHz, $CDCl_3$)

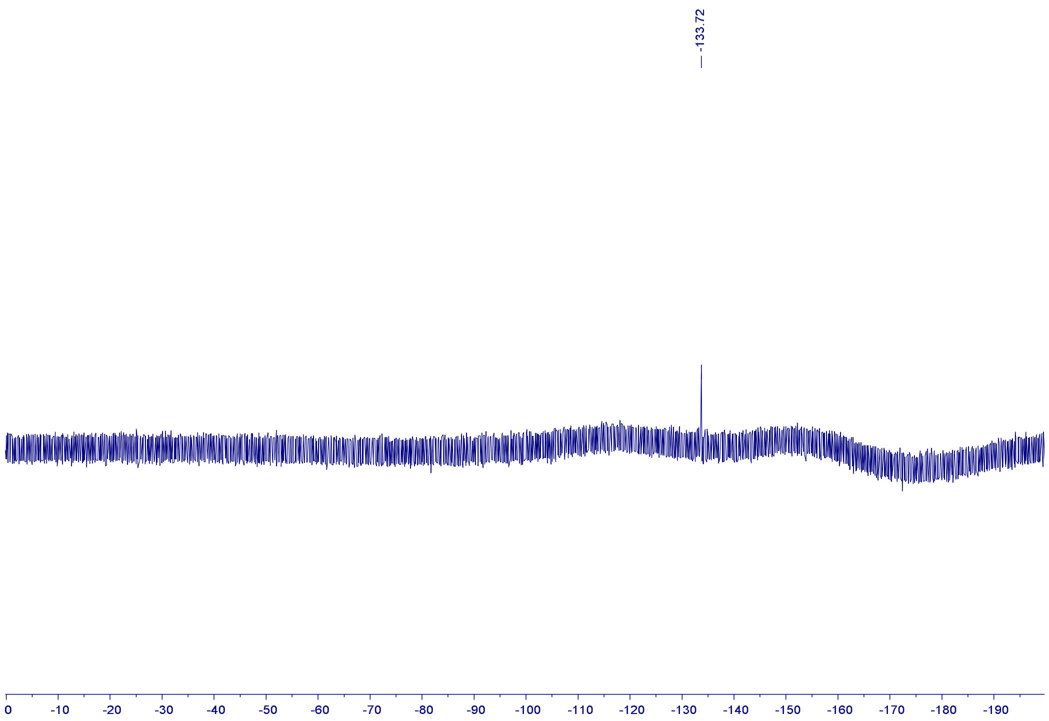

3k – COSY ($CDCl_3$)

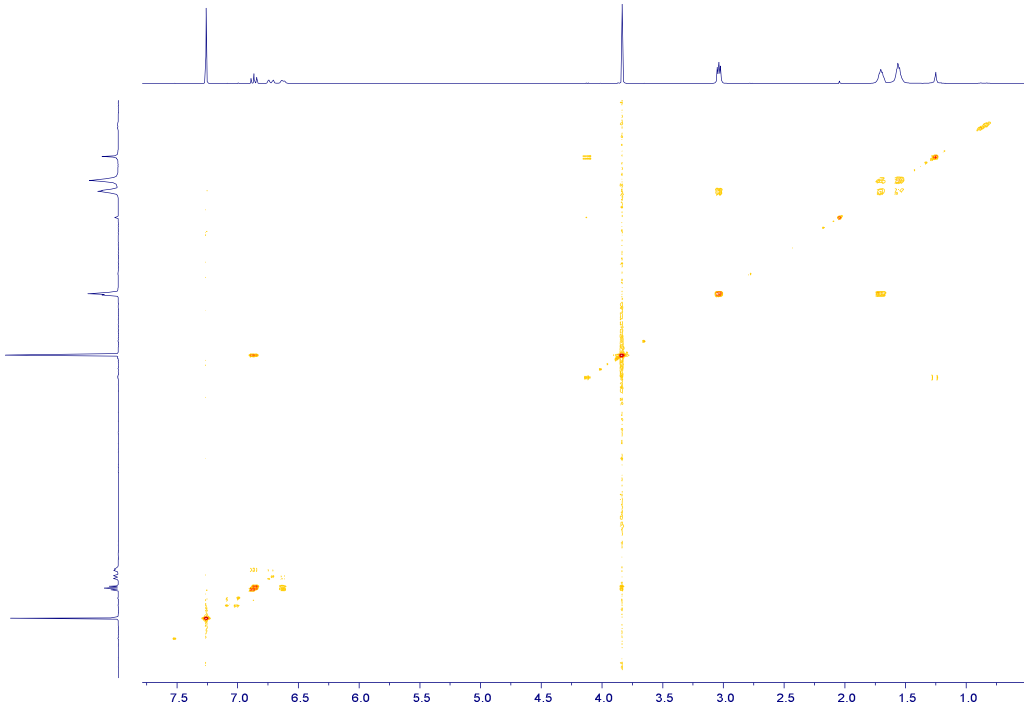

3k – HSQC ($CDCl_3$)

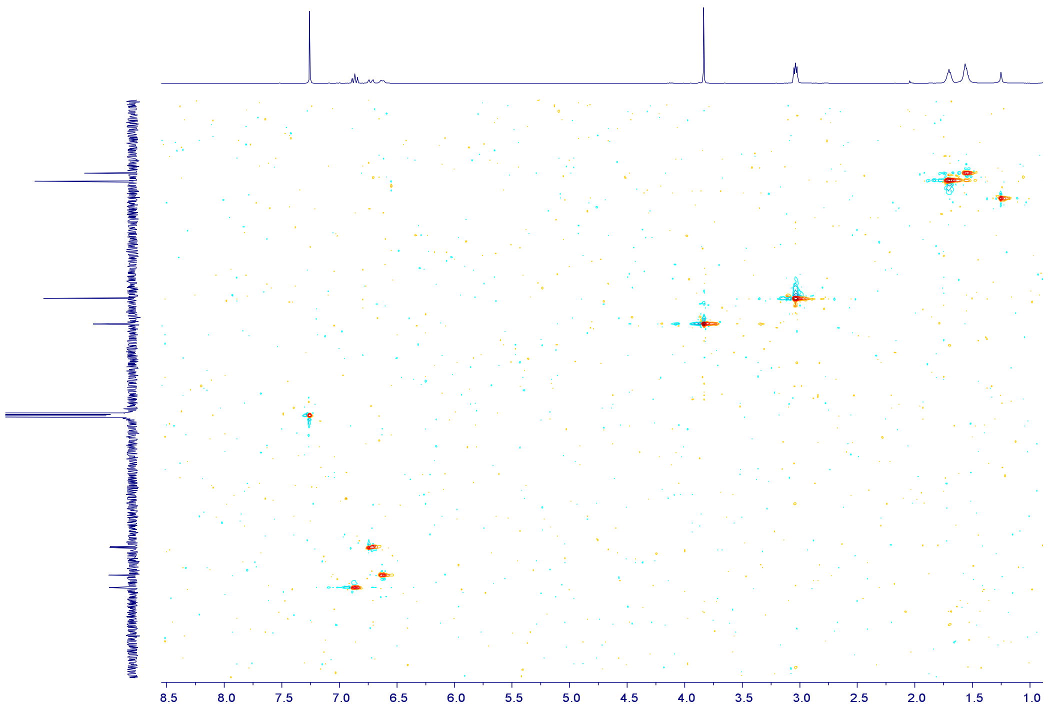

3k – HMBC ($CDCl_3$)

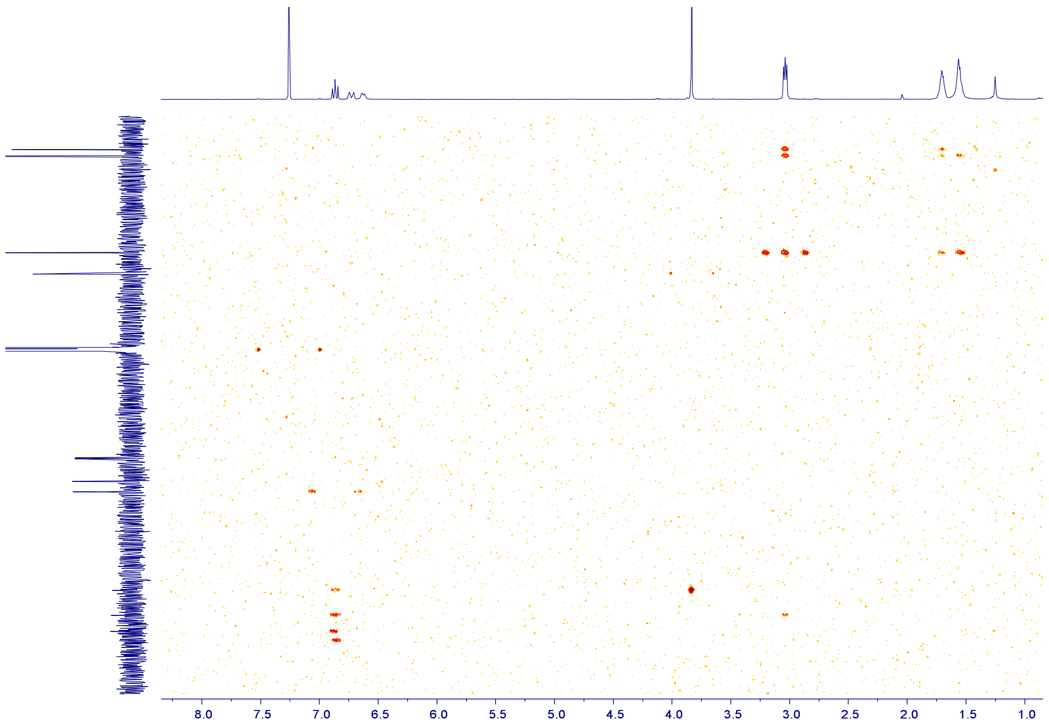

3l – ^{1}H NMR (400 MHz, $CDCl_3$)

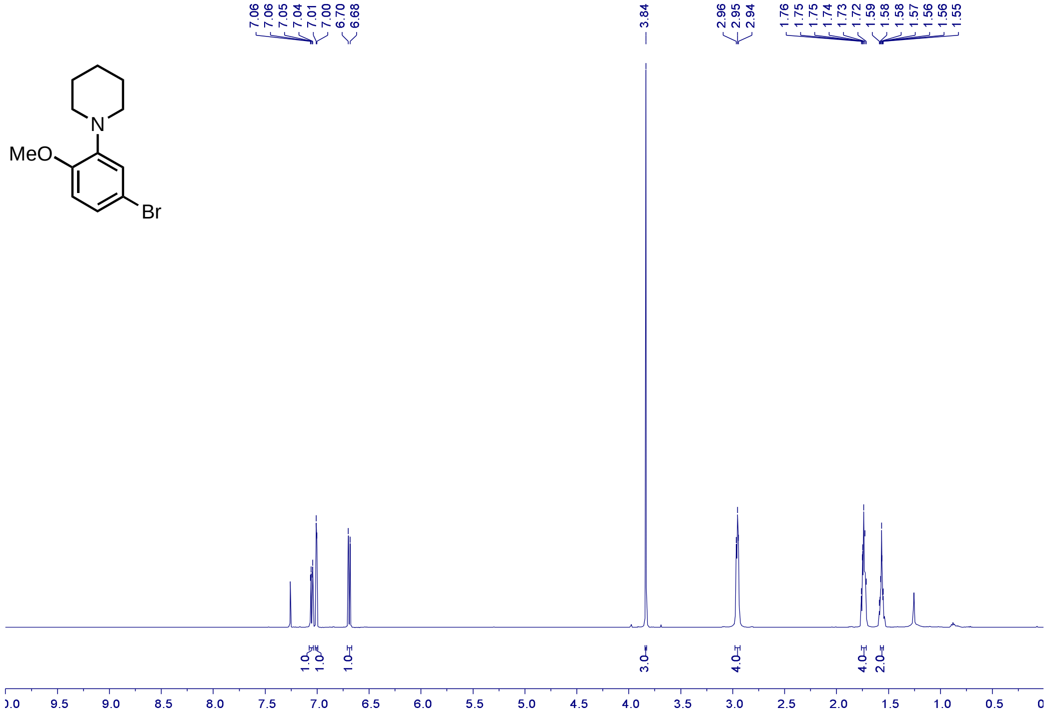

3l – ^{13}C NMR (101 MHz, $CDCl_3$)

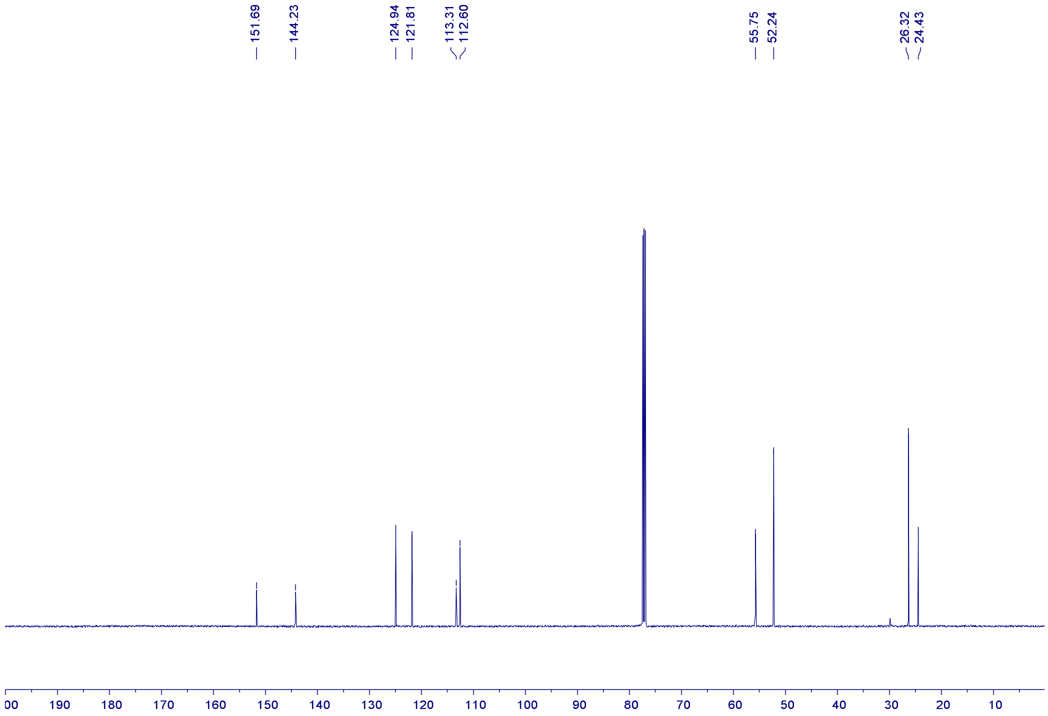

3l – COSY ($CDCl_3$)

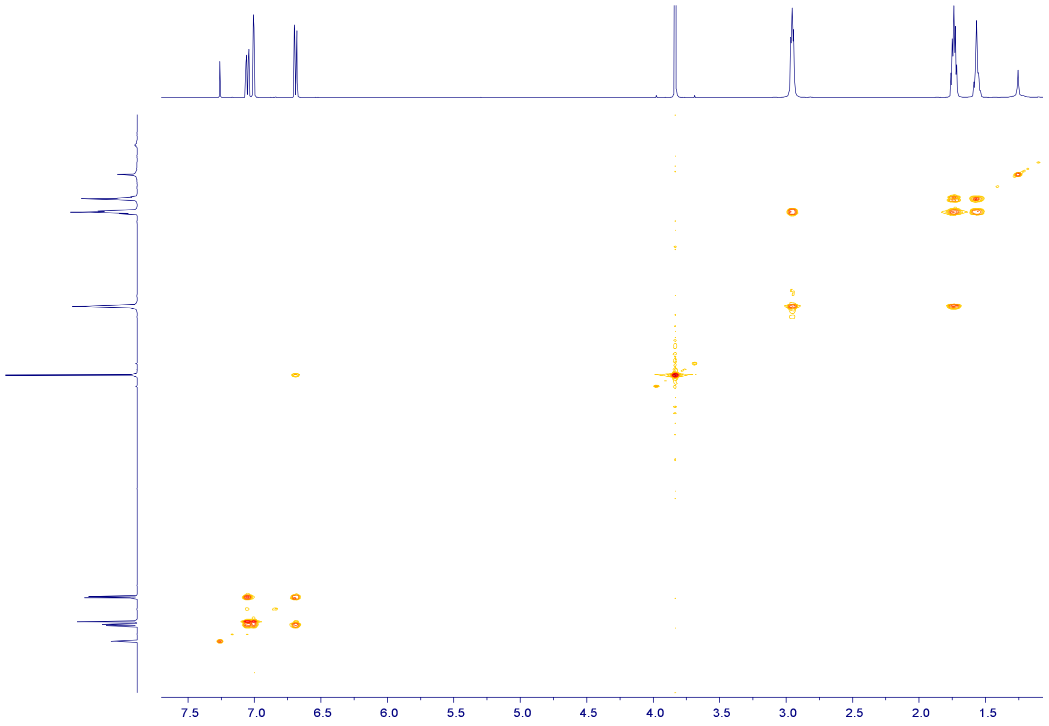

3l – HSQC ($CDCl_3$)

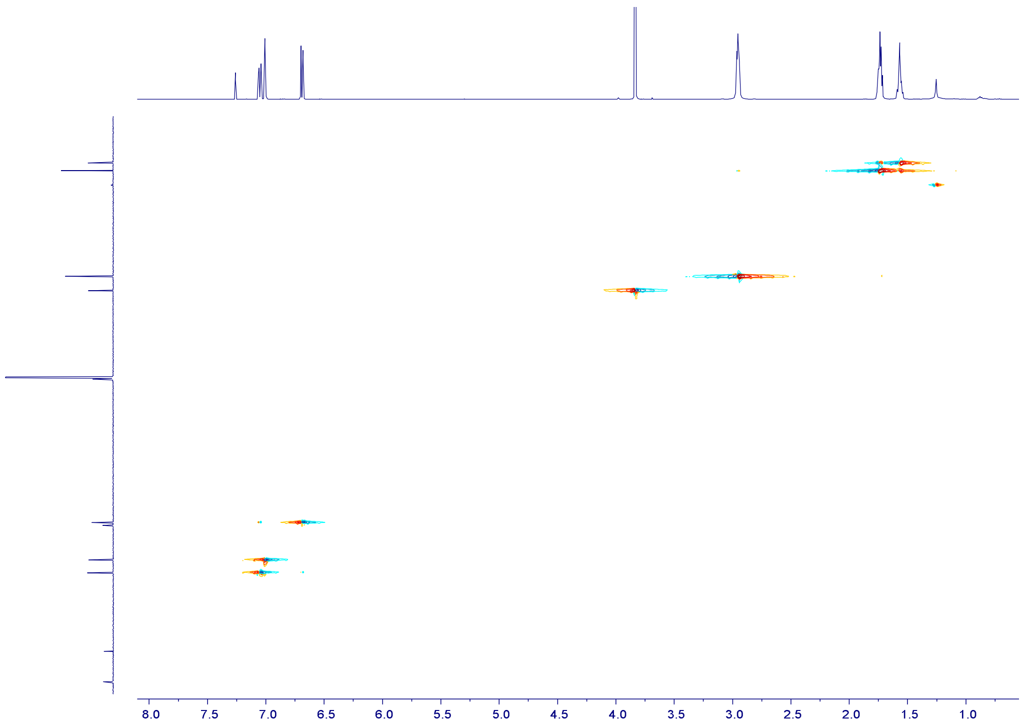

3l – HMBC ($CDCl_3$)

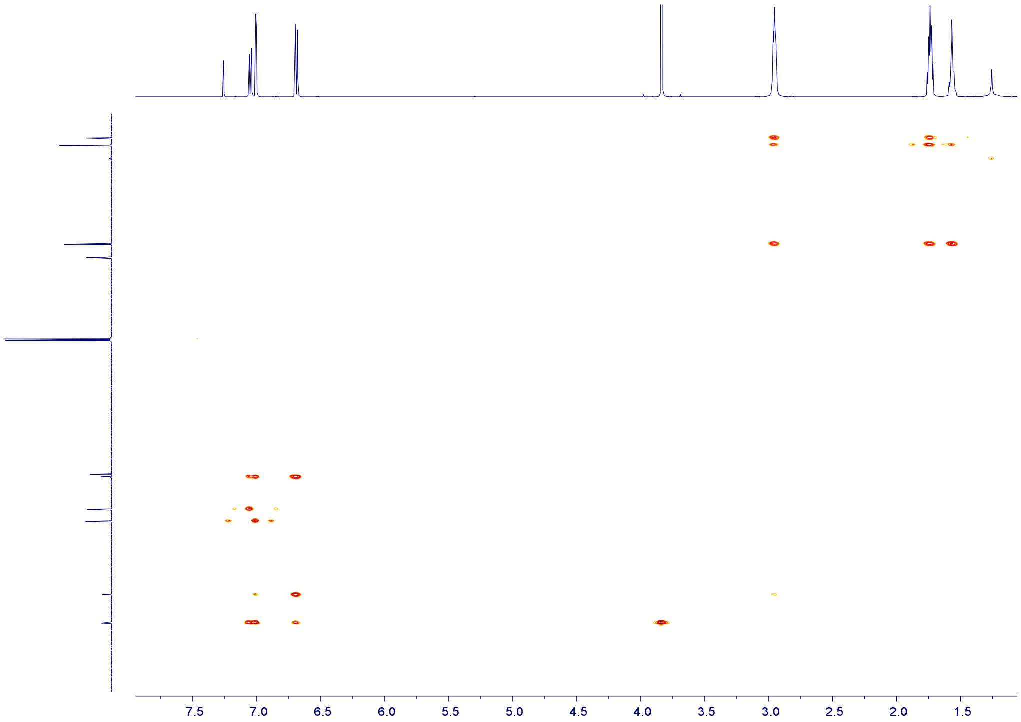

3n – ^{1}H NMR (400 MHz, $CDCl_3$)

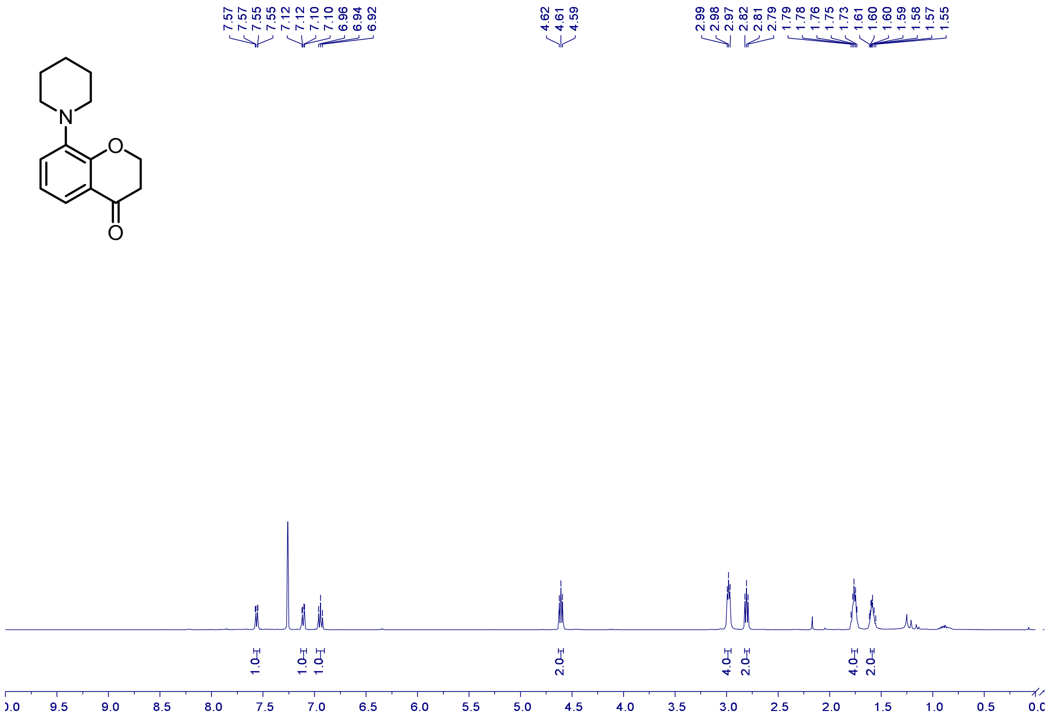

3n – ^{13}C NMR (101 MHz, $CDCl_3$)

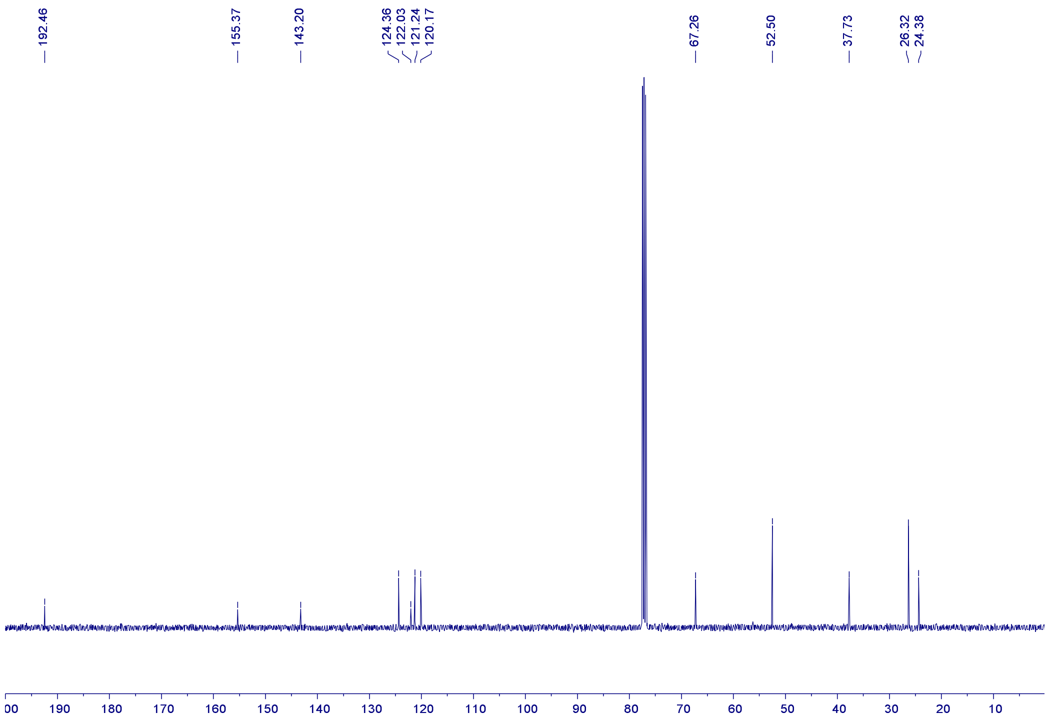

3p – ^{1}H NMR (500 MHz, $CDCl_3$)

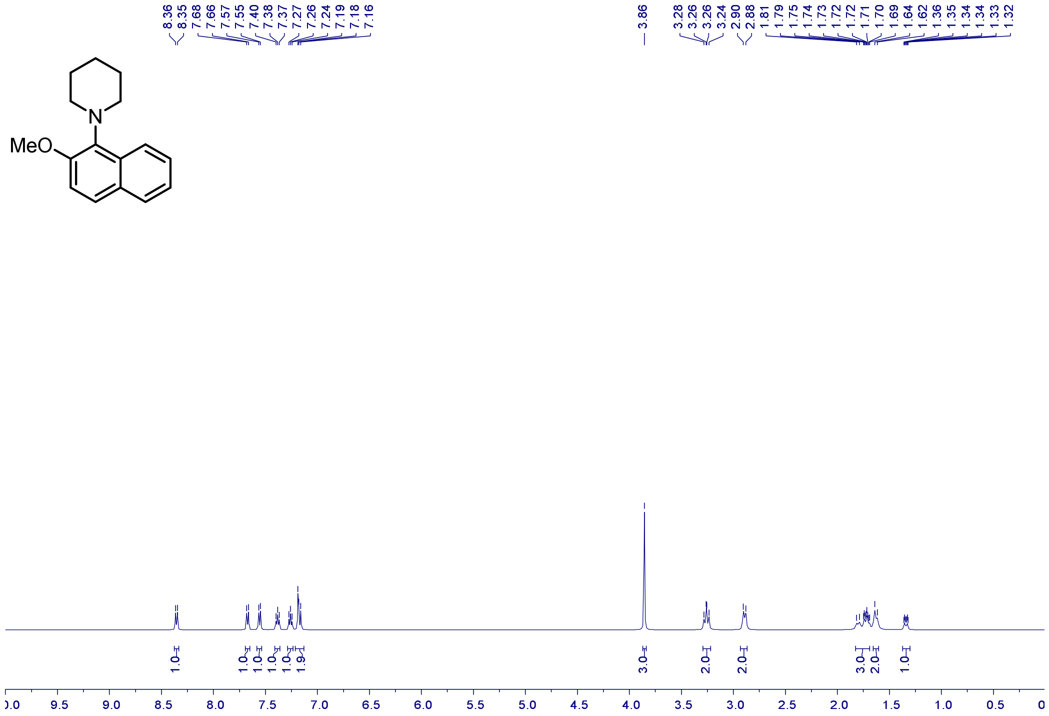

3p – ^{13}C NMR (126 MHz, $CDCl_3$)

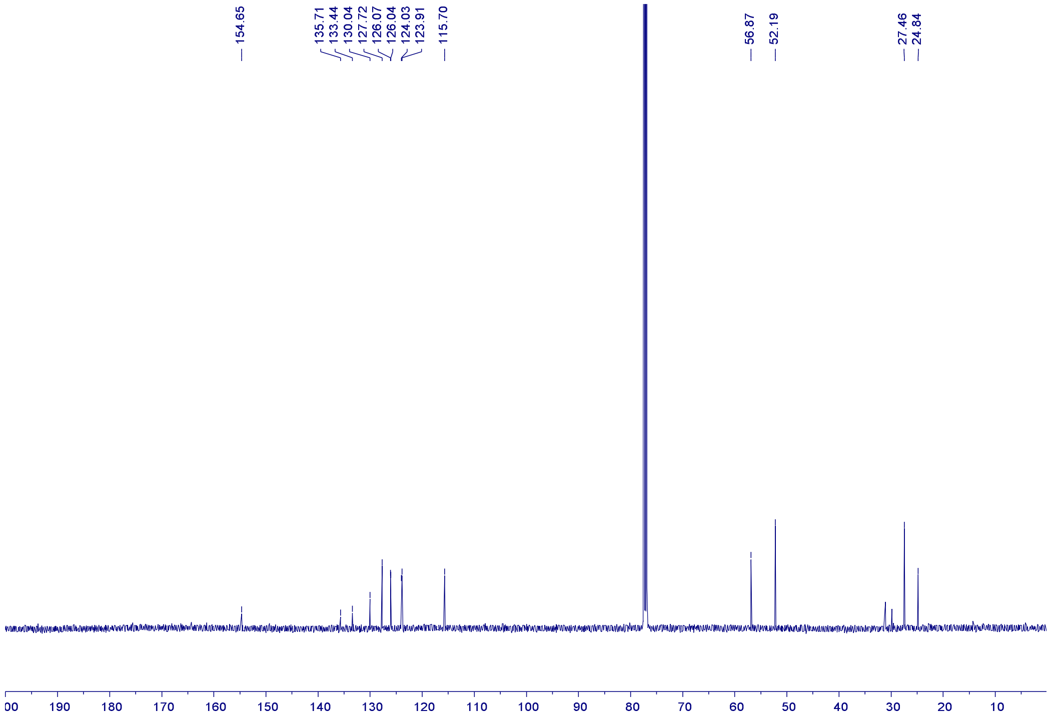

3p – COSY ($CDCl_3$)

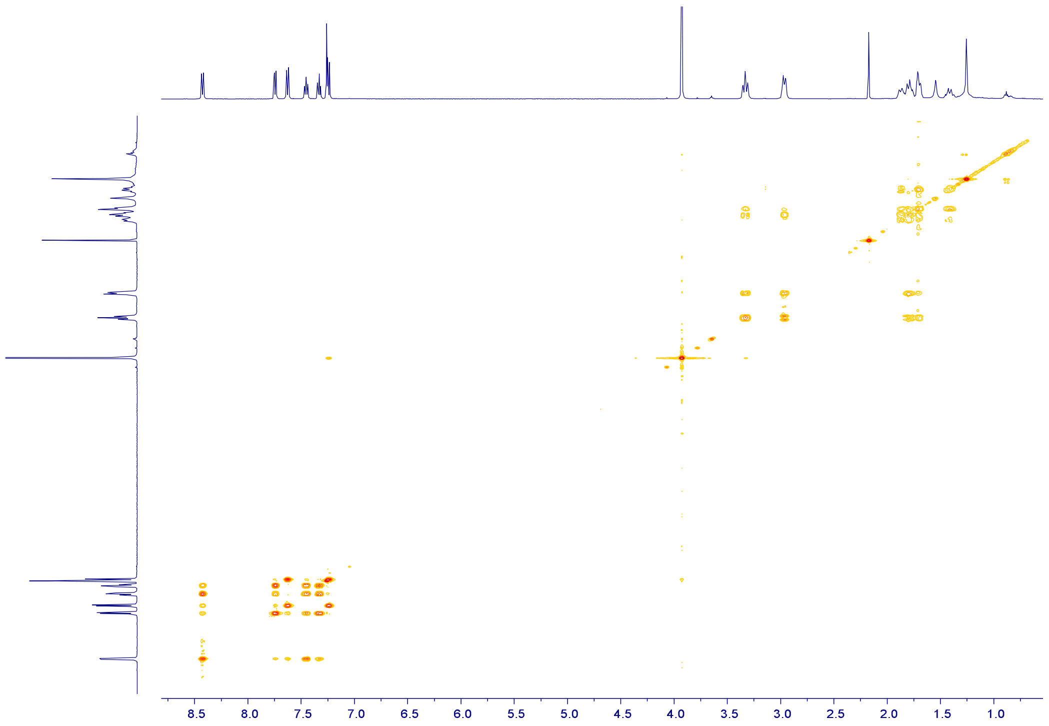

3p – HSQC ($CDCl_3$)

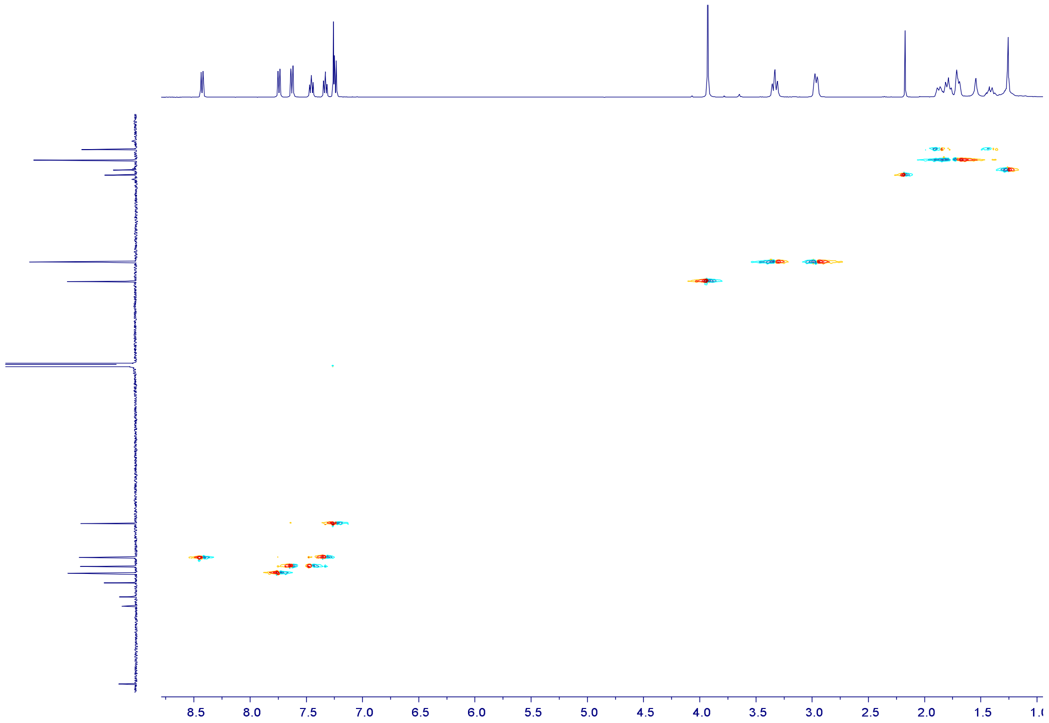

3p – HMBC ($CDCl_3$)

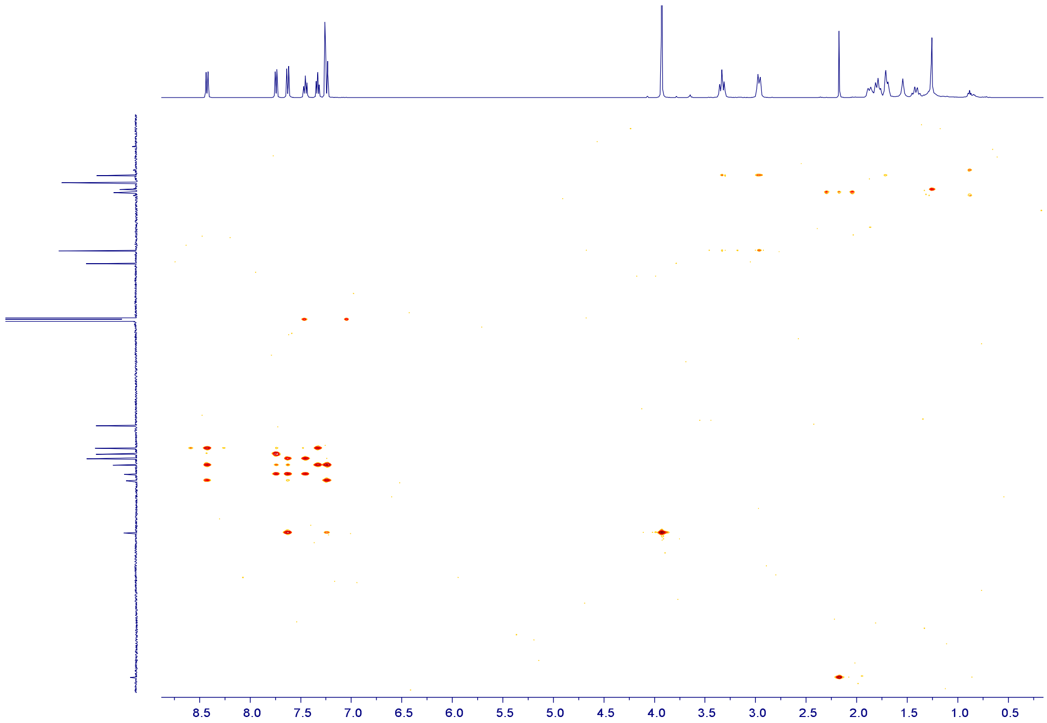

3q – ^{1}H NMR (400 MHz, $CDCl_3$)

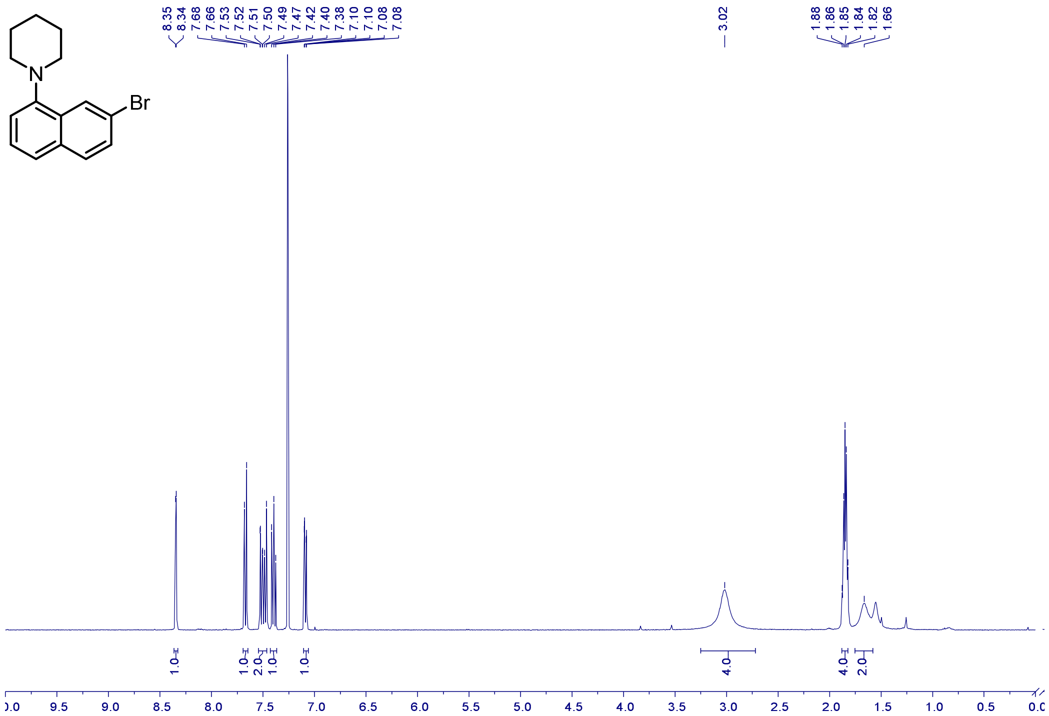

3q – ^{13}C NMR (101 MHz, $CDCl_3$)

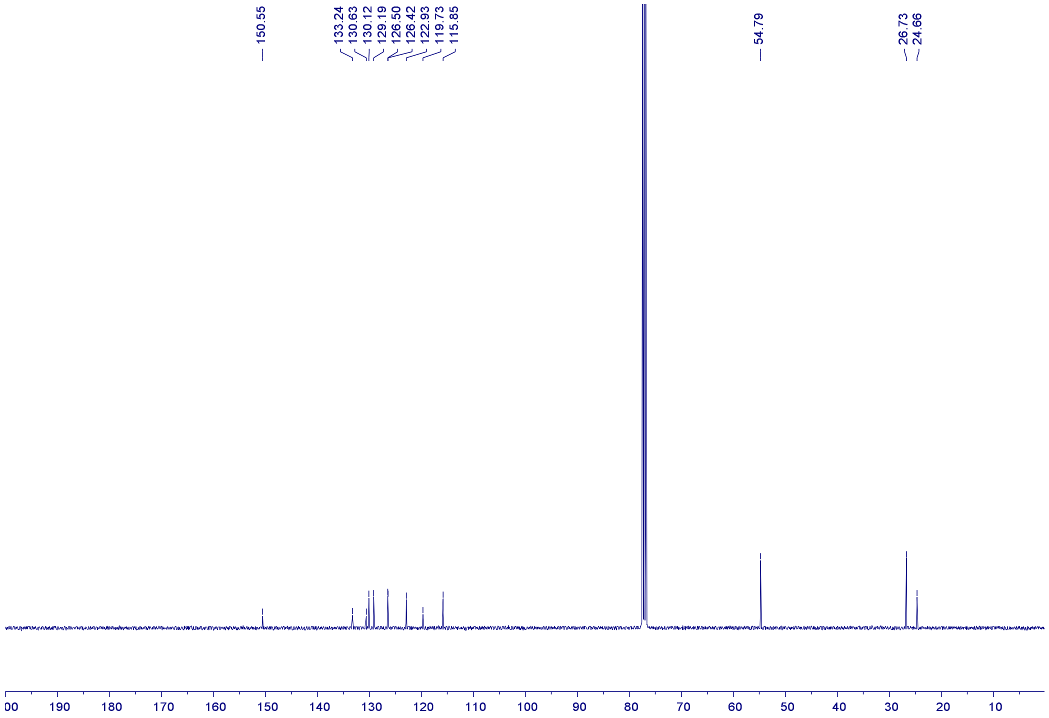

3q – COSY ($CDCl_3$)

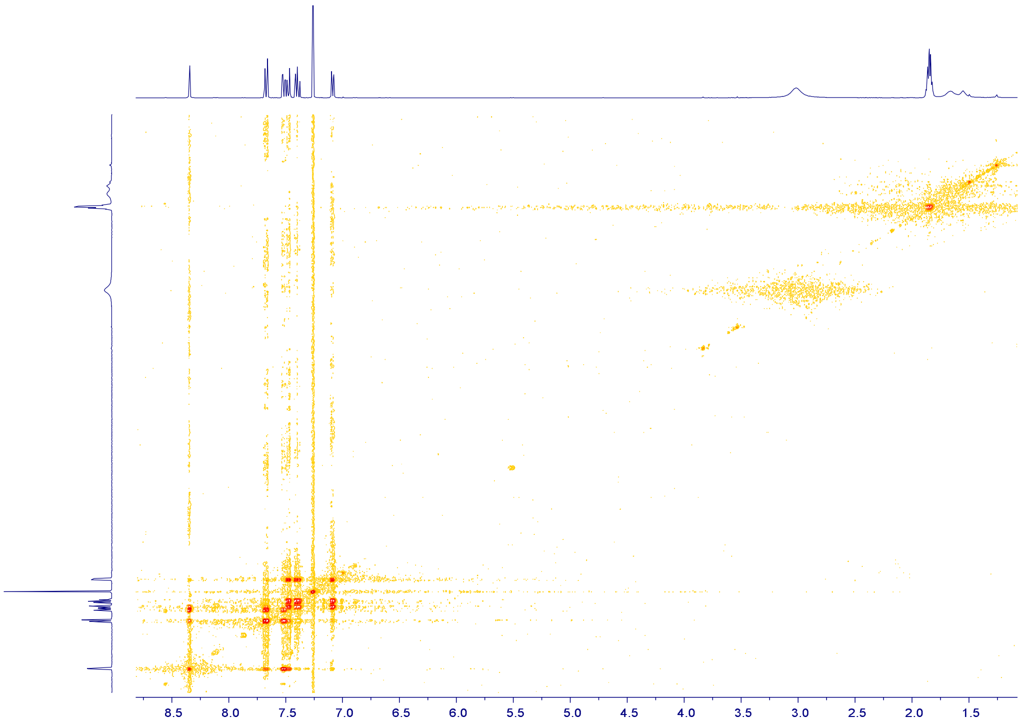

3q – HSQC ($CDCl_3$)

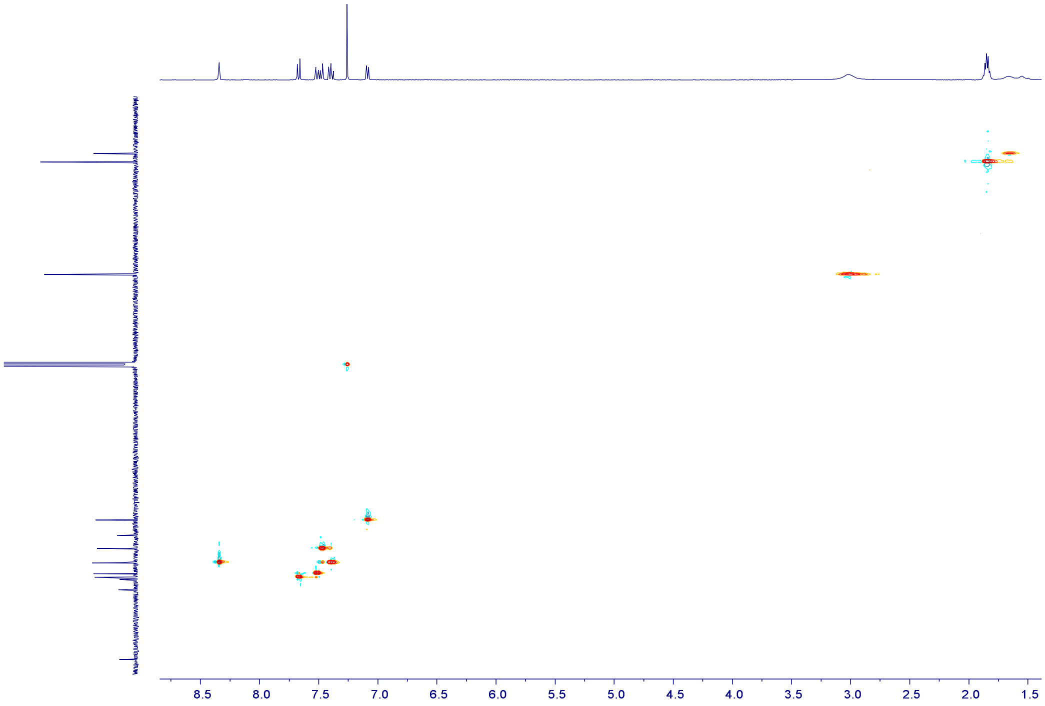

3q – HMBC ($CDCl_3$)

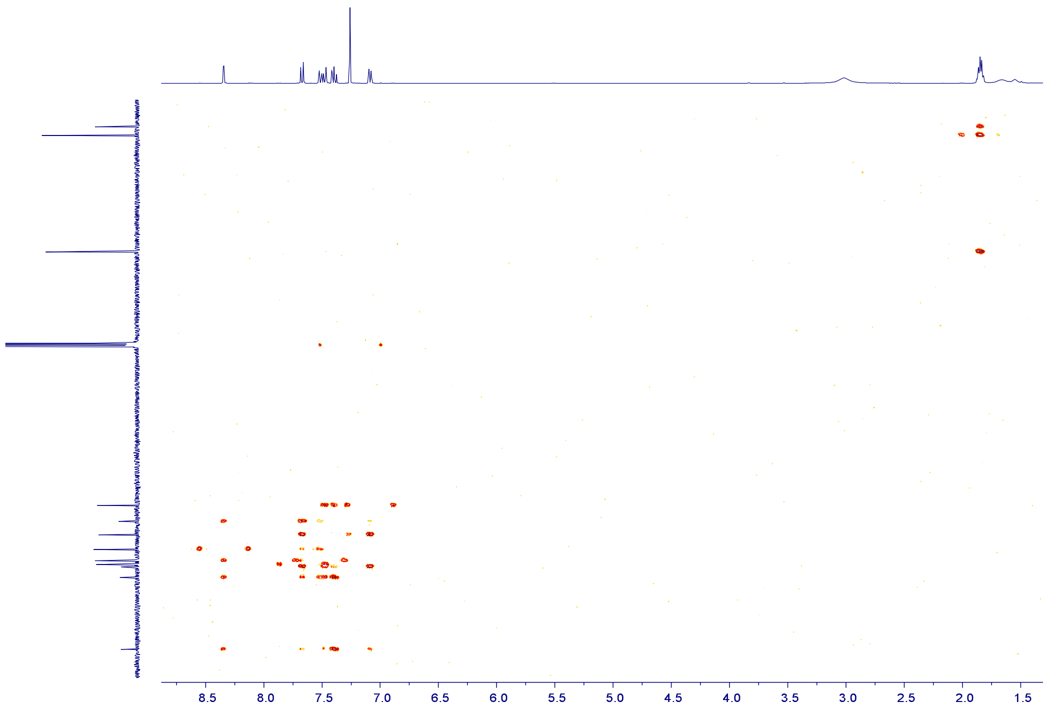

3q' – ^{1}H NMR (400 MHz, $CDCl_3$)

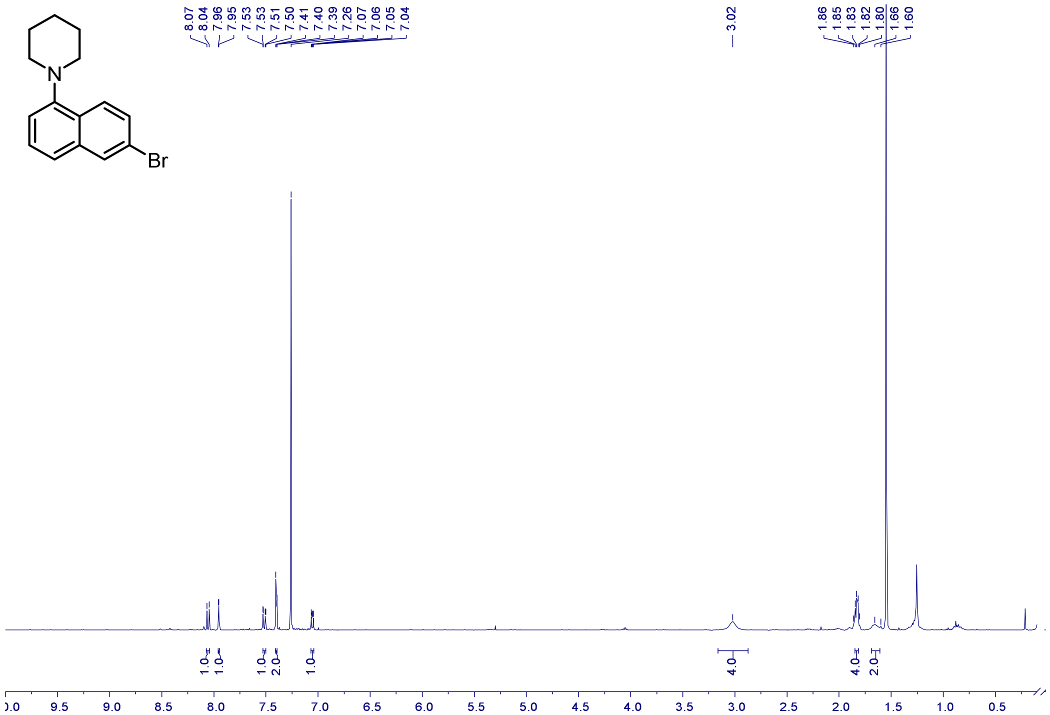

3q' – ^{13}C NMR (101 MHz, $CDCl_3$)

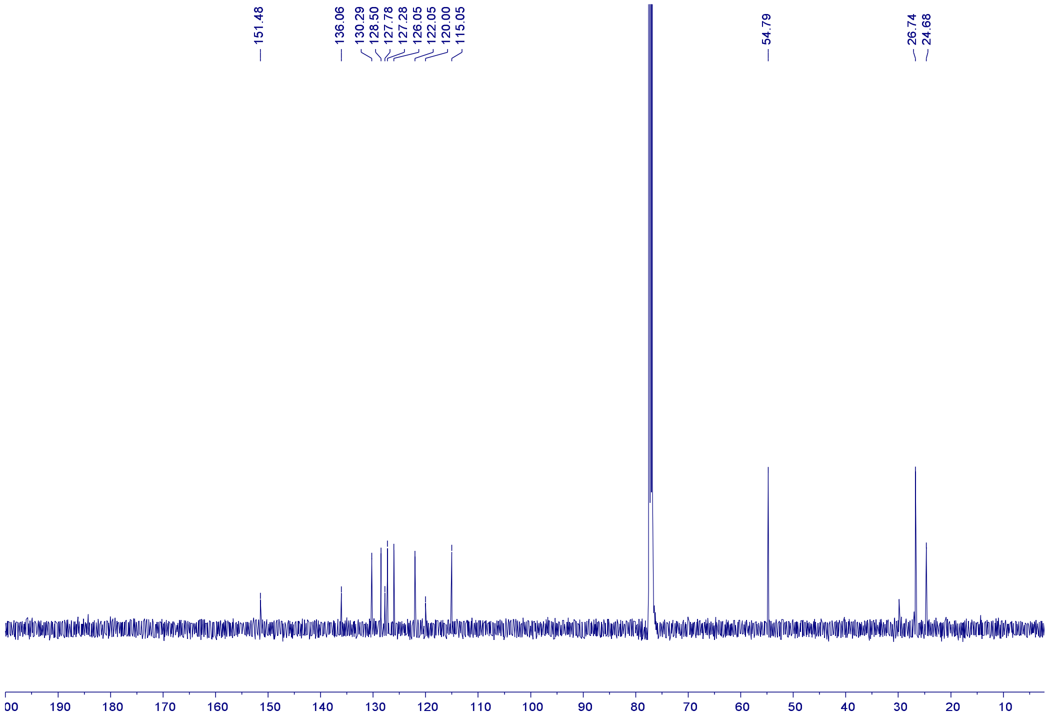

3r – ^{1}H NMR (500 MHz, $CDCl_3$)

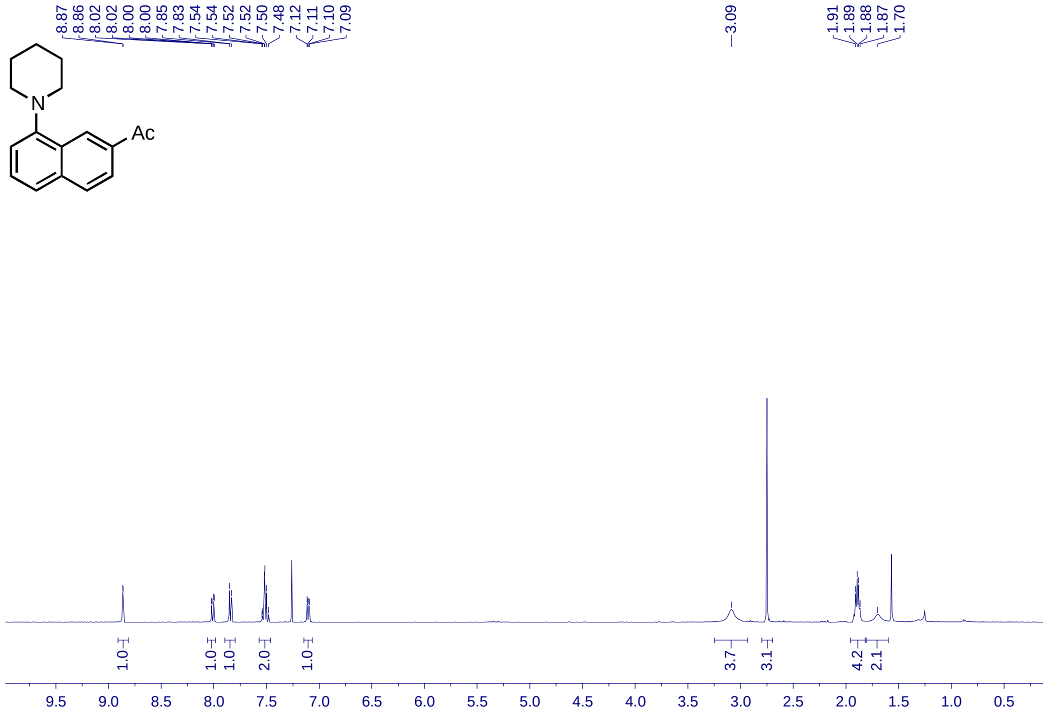

3r – ^{13}C NMR (126 MHz, $CDCl_3$)

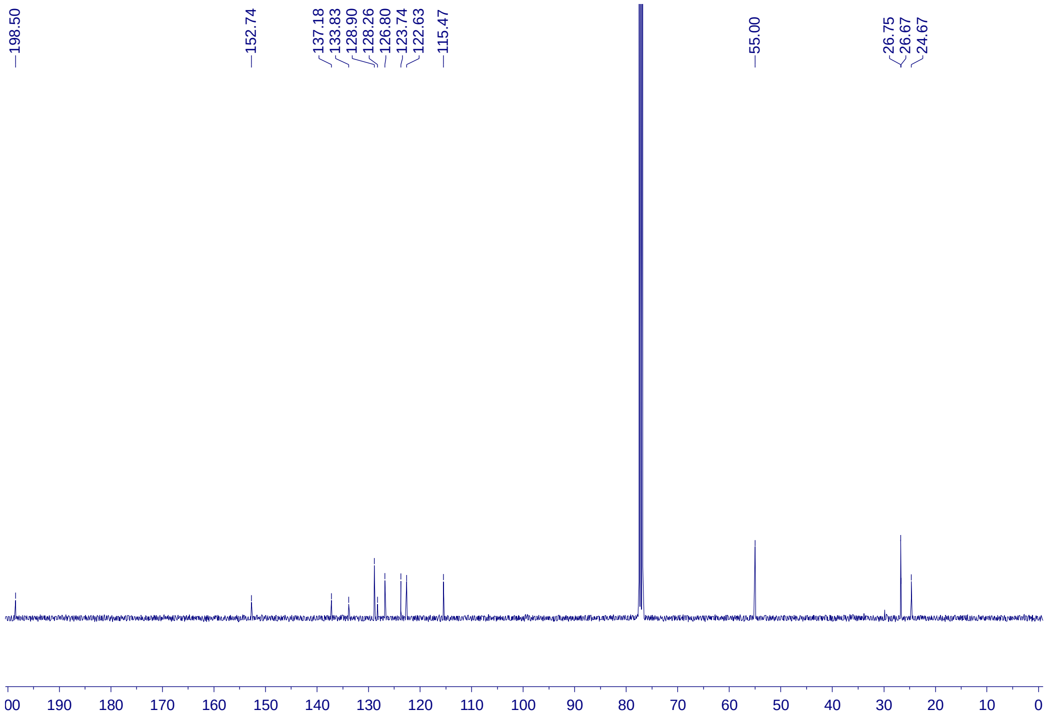

3r' – ^{1}H NMR (500 MHz, $CDCl_3$)

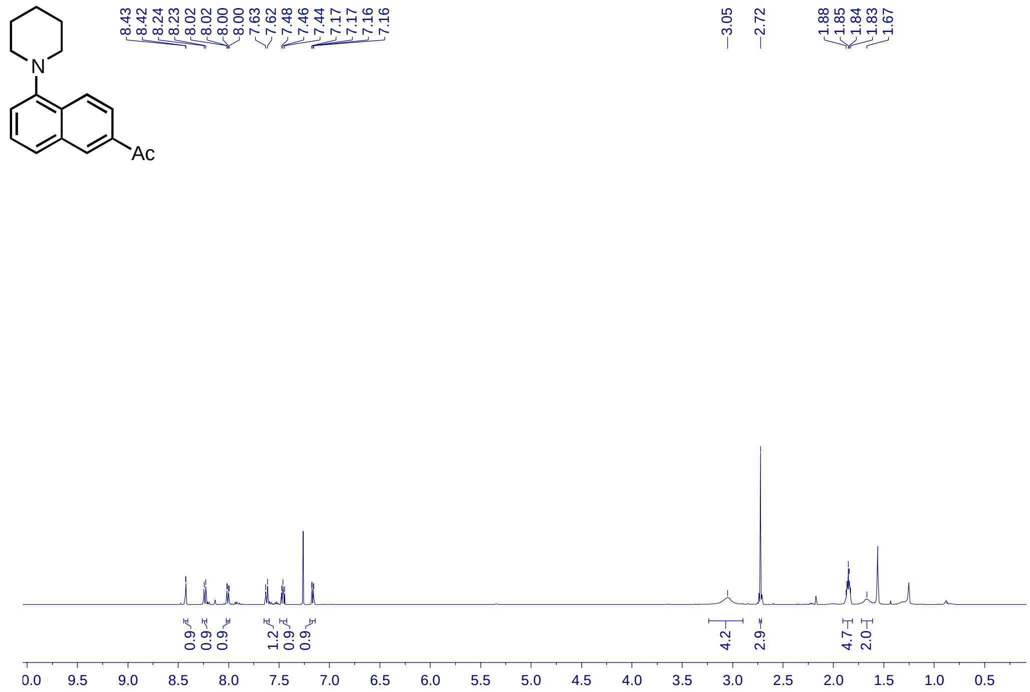

3r' – ^{13}C NMR (126 MHz, $CDCl_3$)

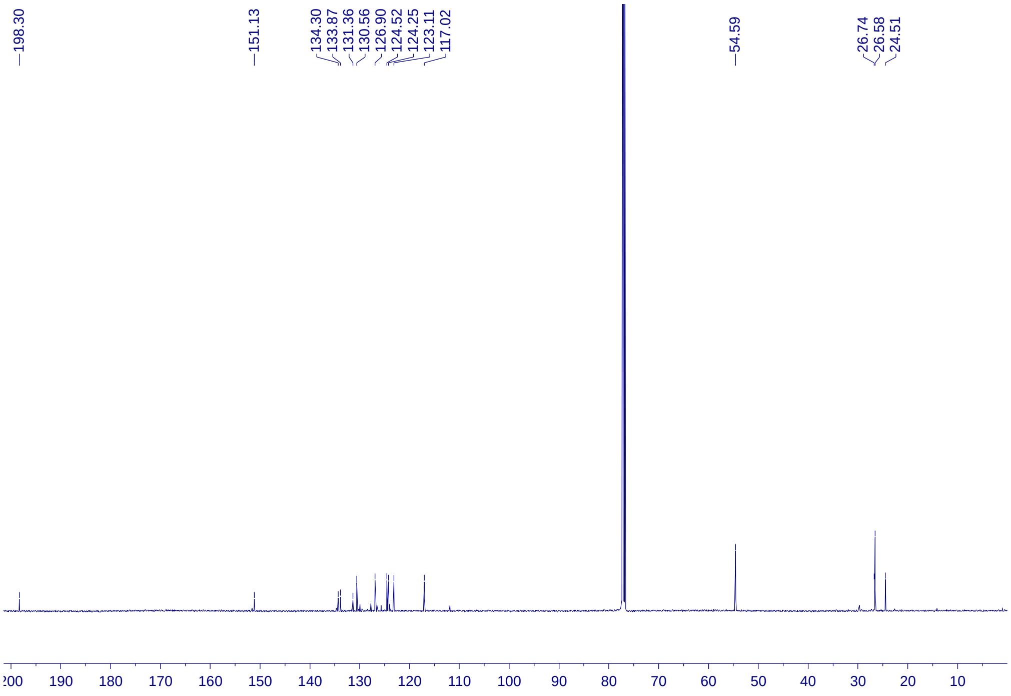

3t – ^{1}H NMR (400 MHz, $CDCl_3$)

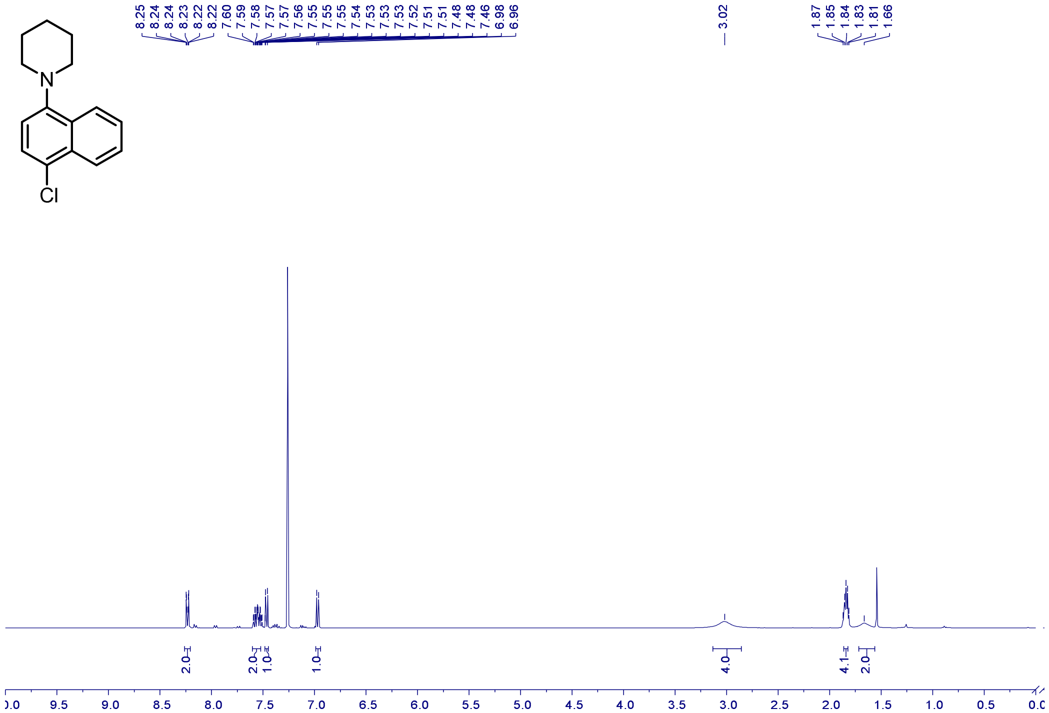

3t – ^{13}C NMR (101 MHz, $CDCl_3$)

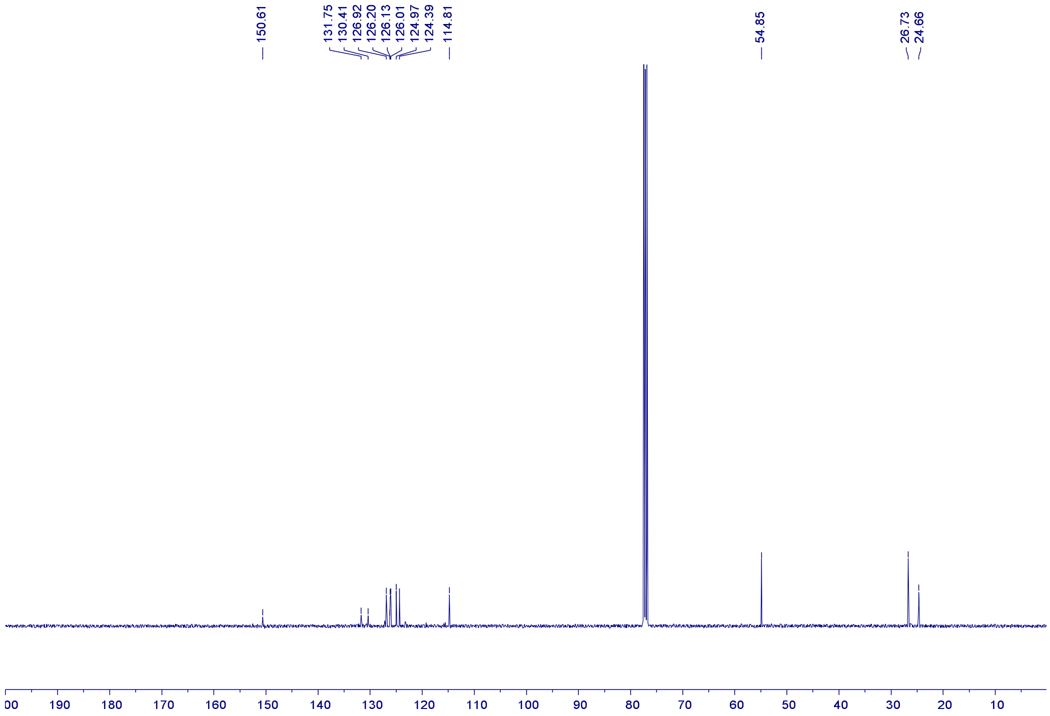

3u – ^{1}H NMR (500 MHz, $CDCl_3$)

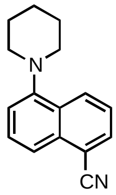

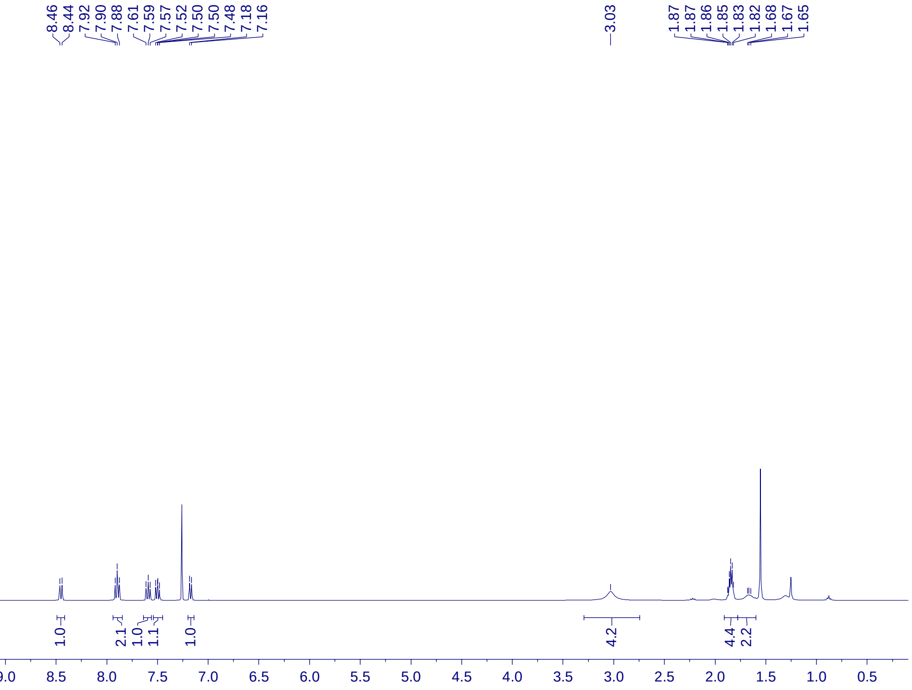

3u – ^{13}C NMR (126 MHz, $CDCl_3$)

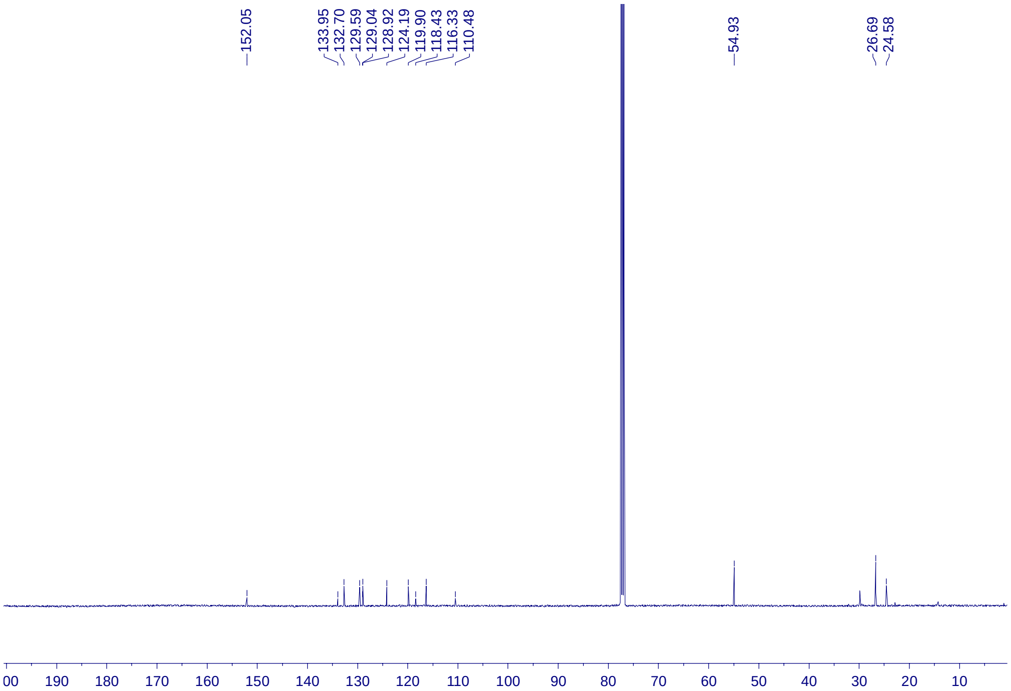

3v – ^{1}H NMR (500 MHz, $CDCl_3$)

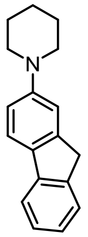

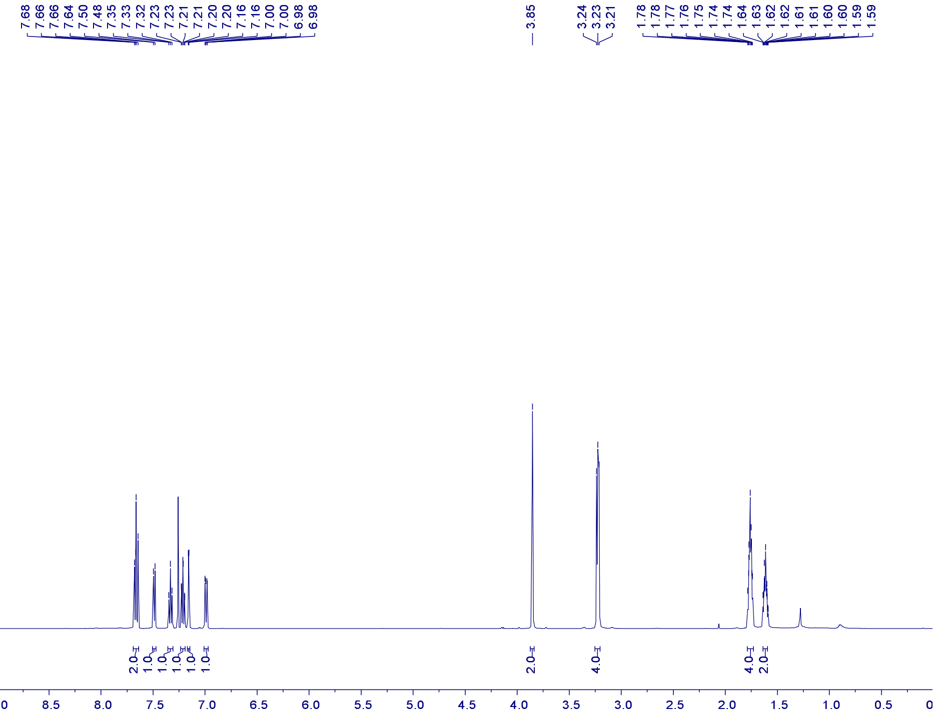

3v – ^{13}C NMR (126 MHz, $CDCl_3$)

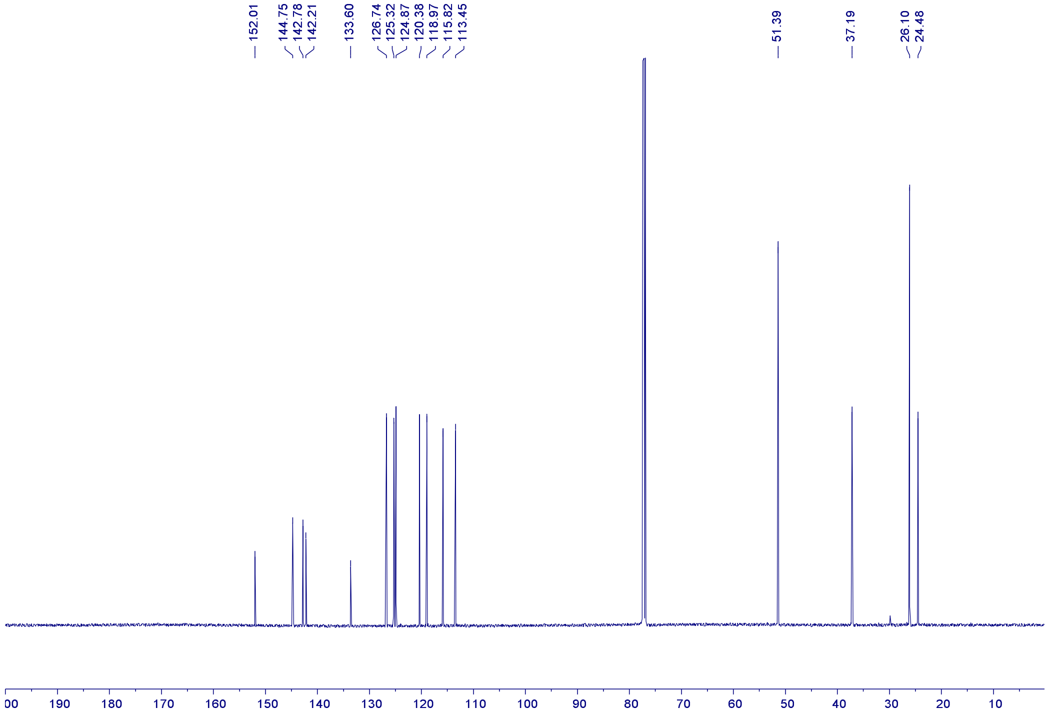

3v – COSY ($CDCl_3$)

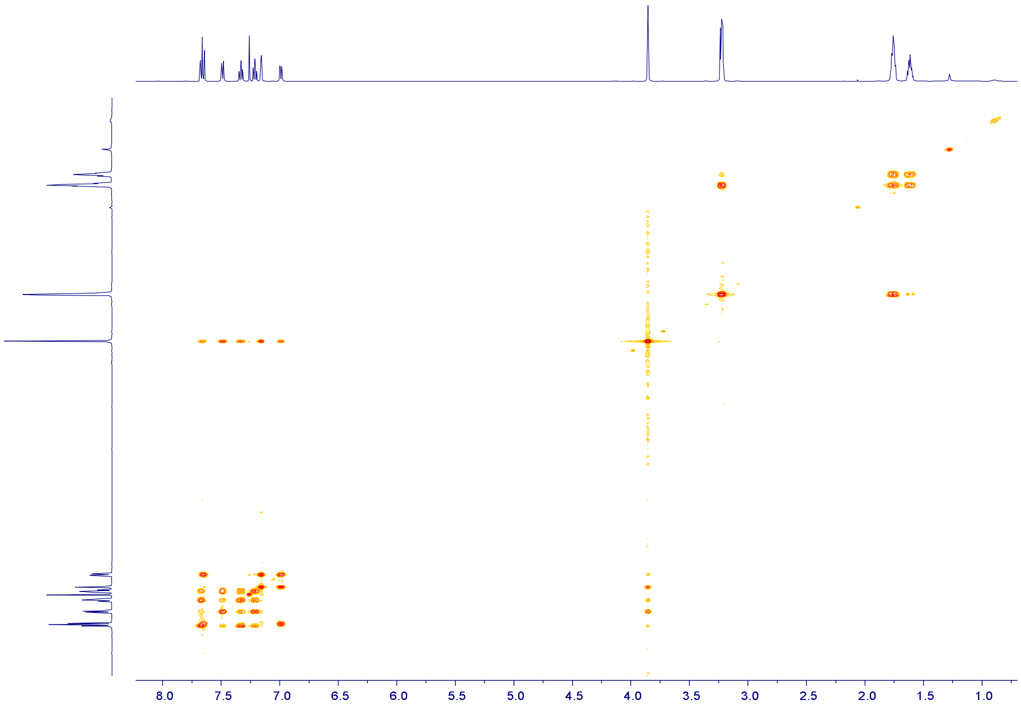

3v – HSQC ($CDCl_3$)

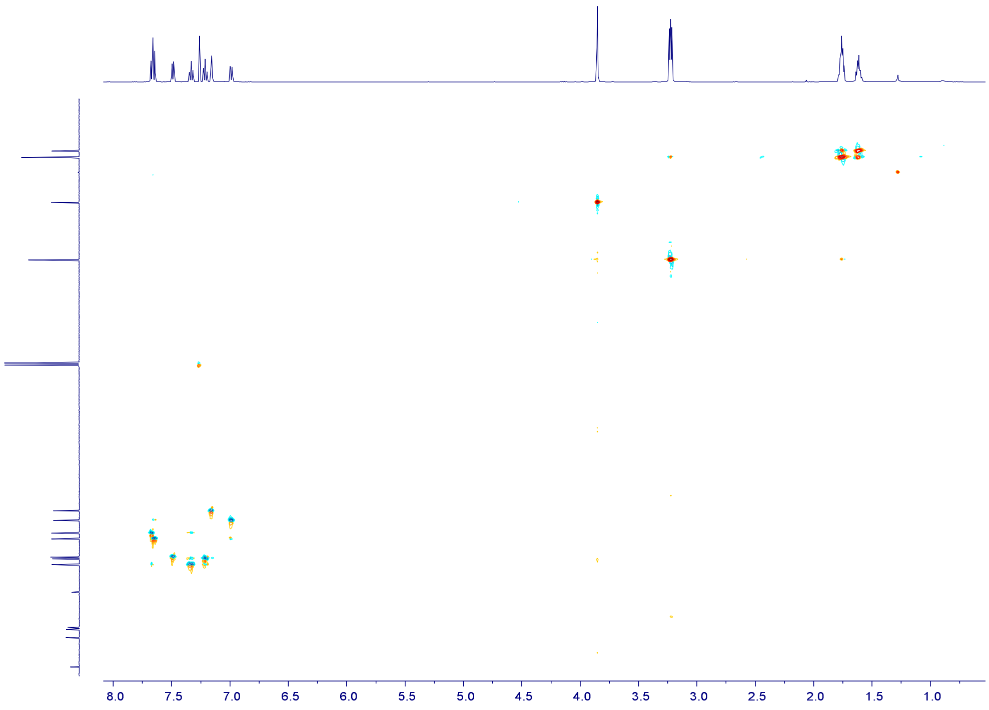

3v – HMBC ($CDCl_3$)

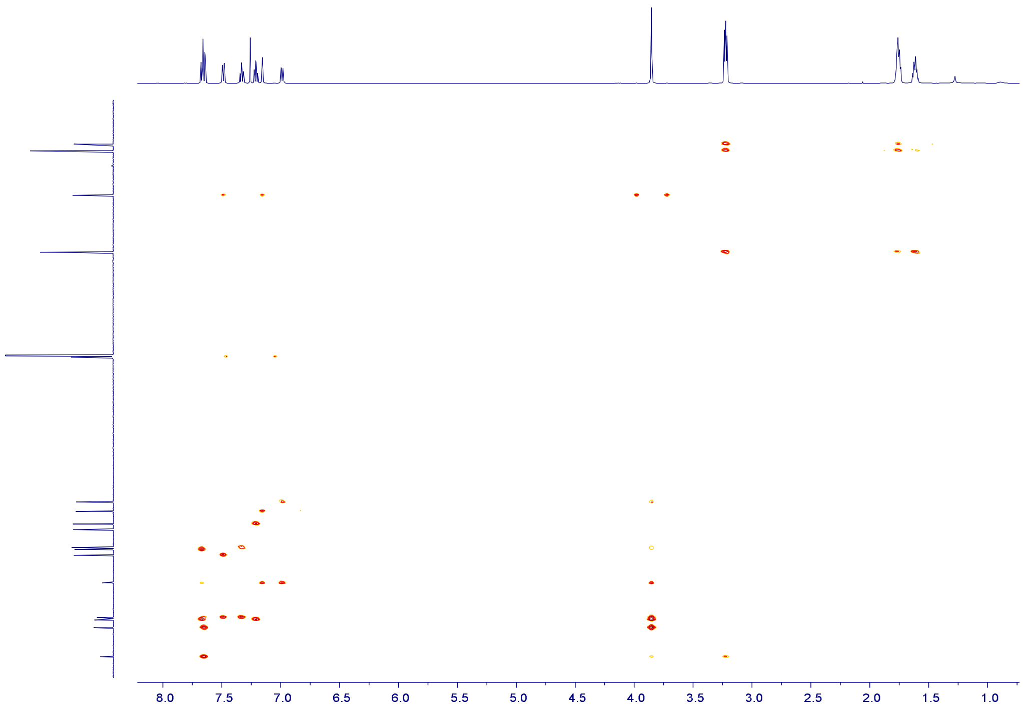

3w – ^{1}H NMR (500 MHz, $CDCl_3$)

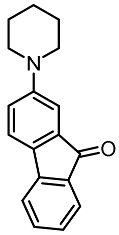

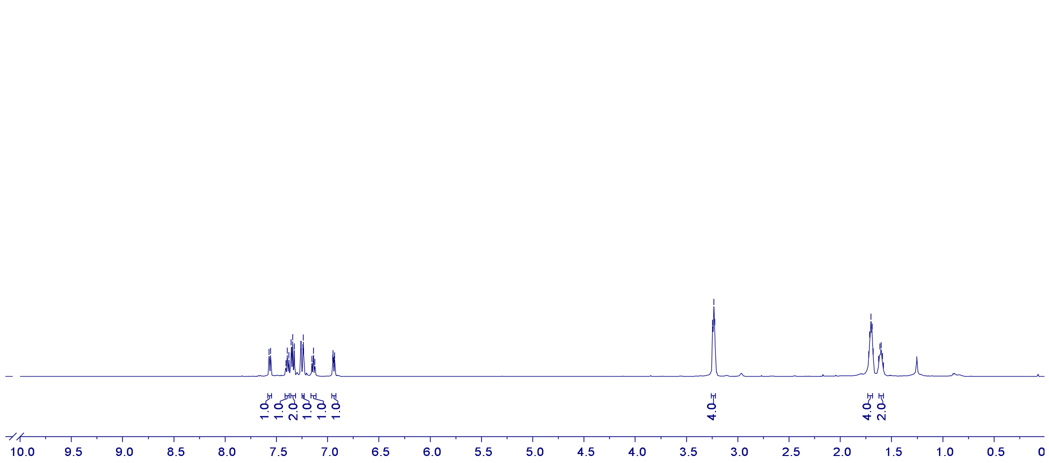

3w – ^{13}C NMR (126 MHz, $CDCl_3$)

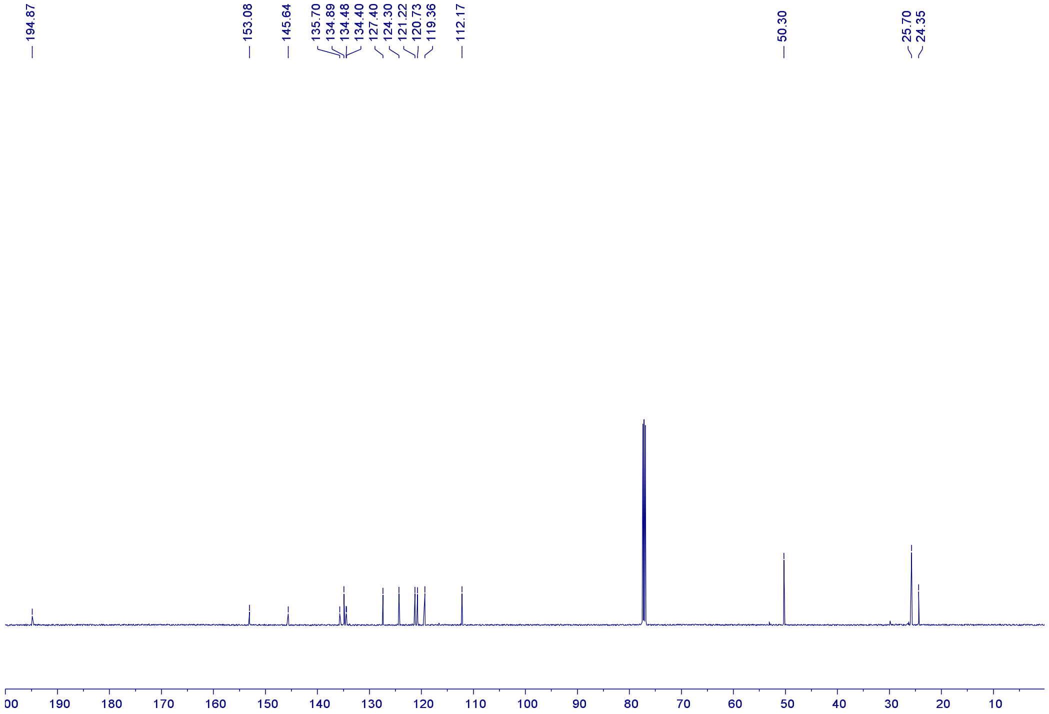

3w – COSY ($CDCl_3$)

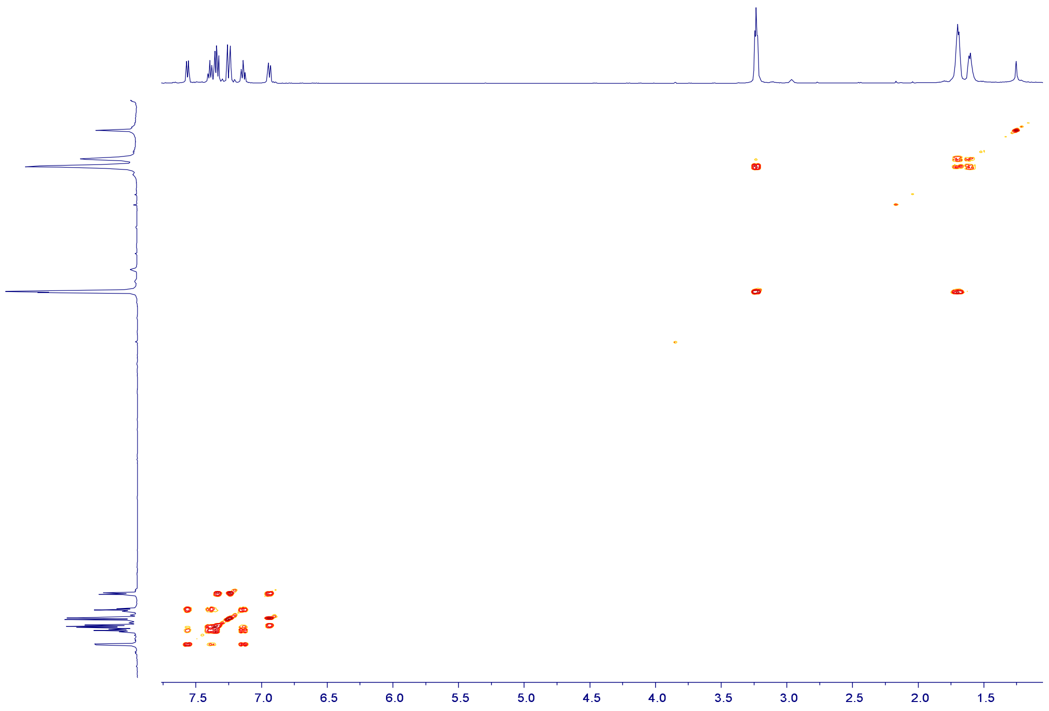

3w – HSQC ($CDCl_3$)

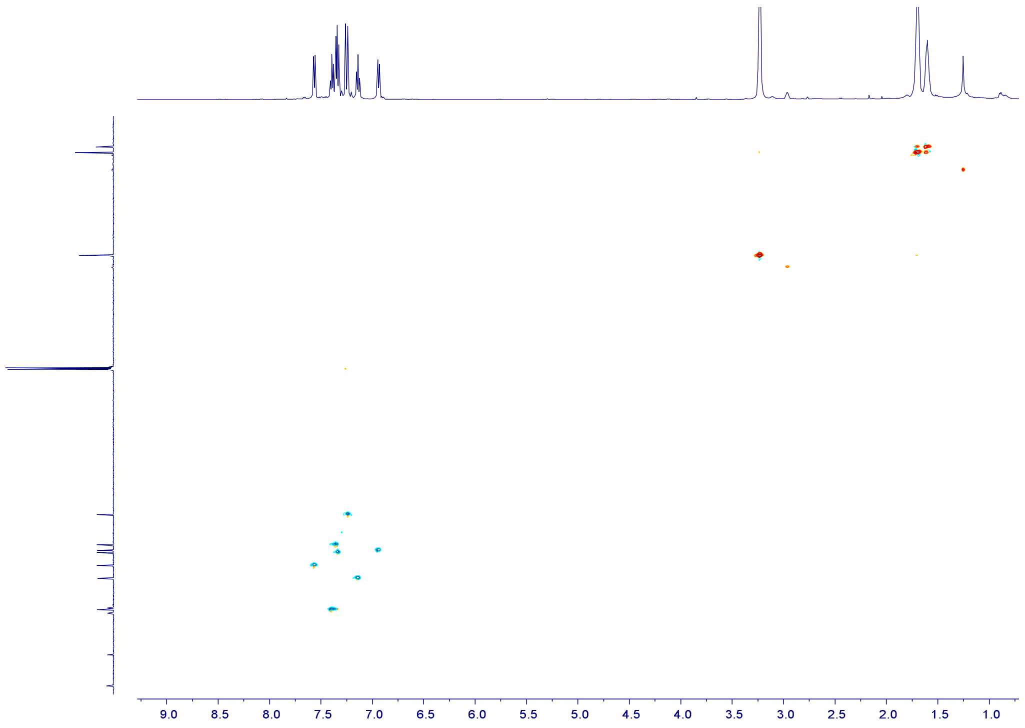

3w – HMBC ($CDCl_3$)

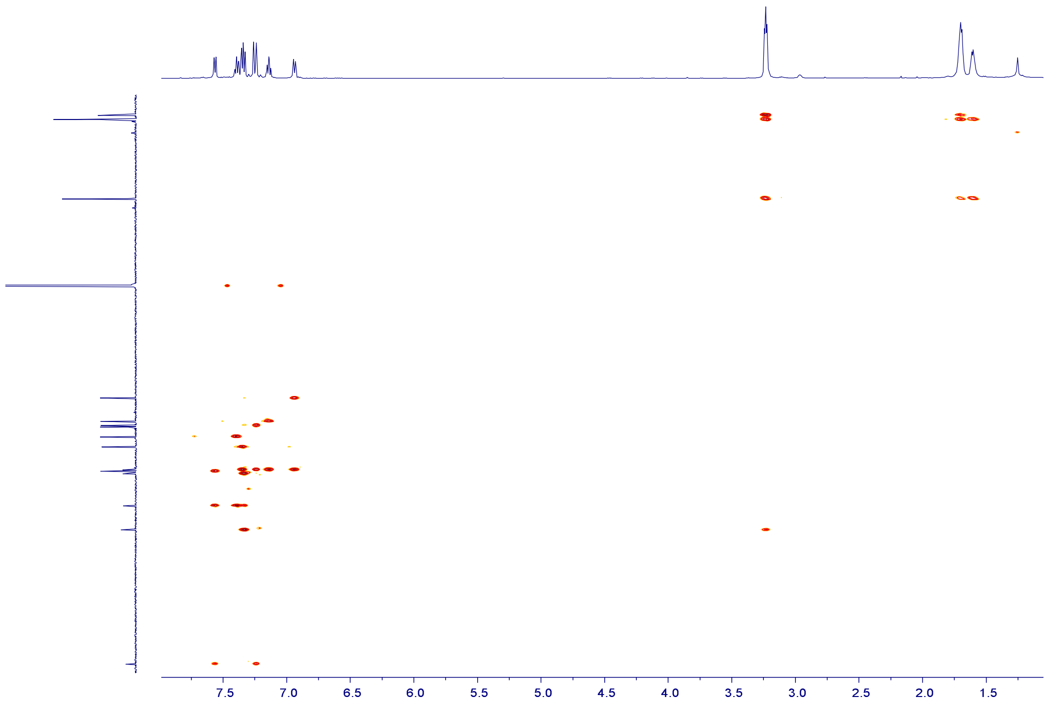

3x – ^{1}H NMR (400 MHz, $CDCl_3$)

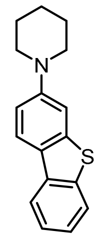

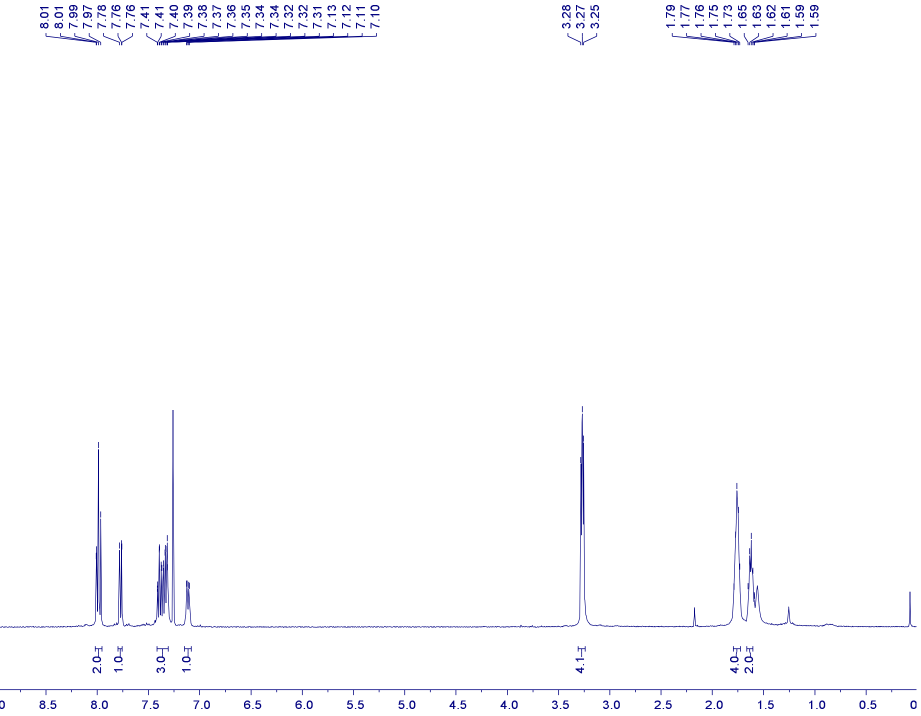

3x – ^{13}C NMR (101 MHz, $CDCl_3$)

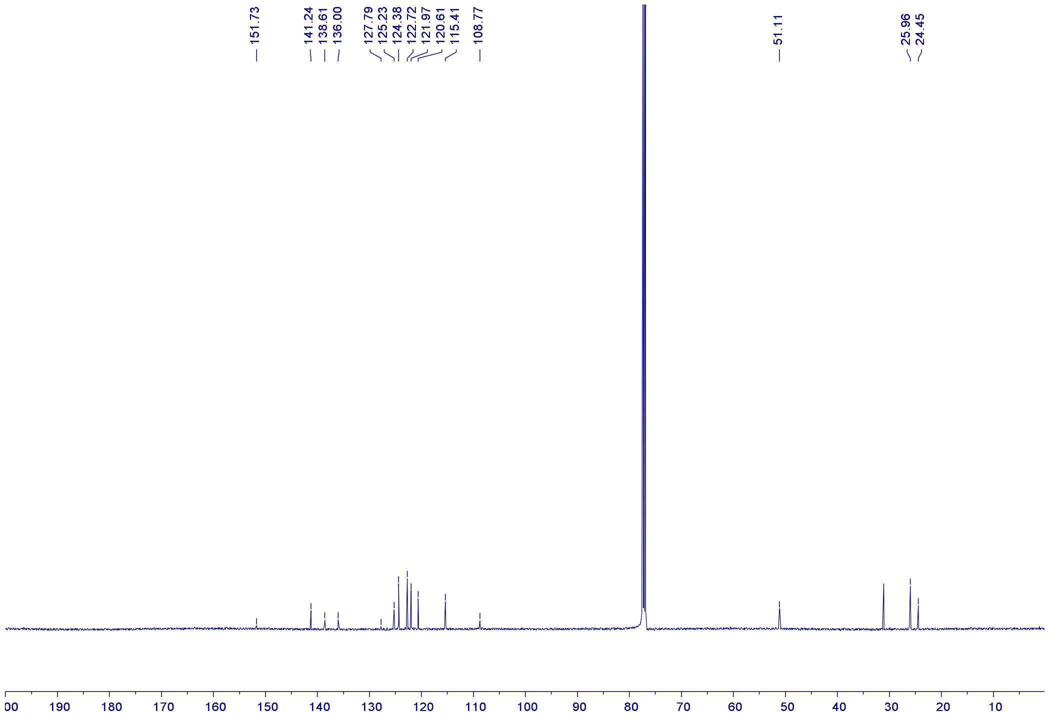

3x – COSY ($CDCl_3$)

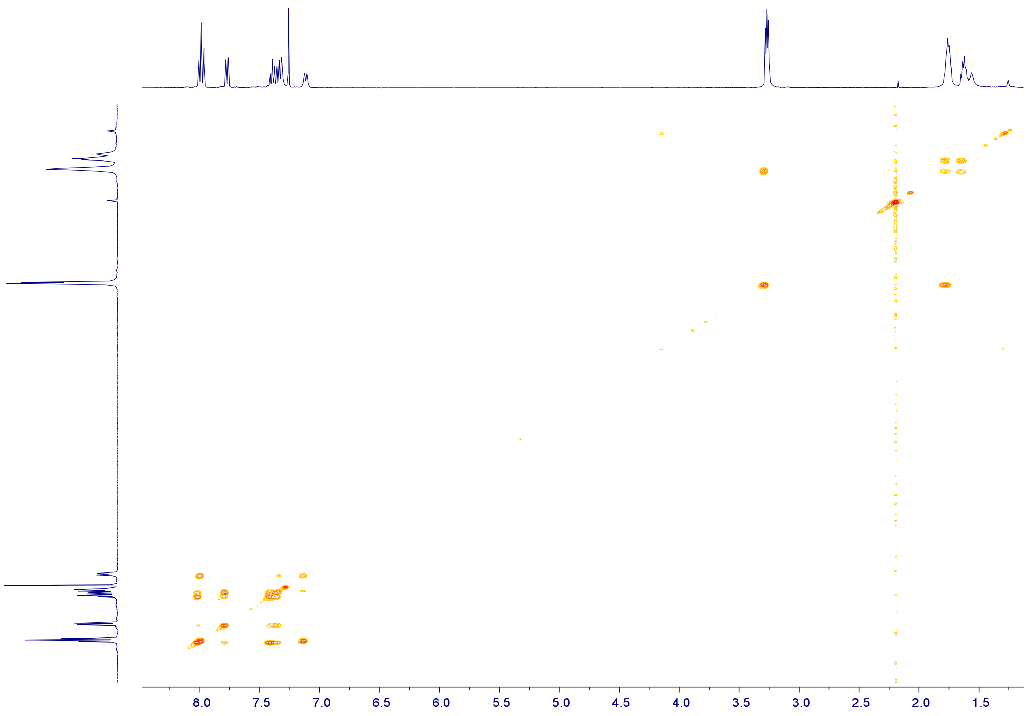

3x – HSQC ($CDCl_3$)

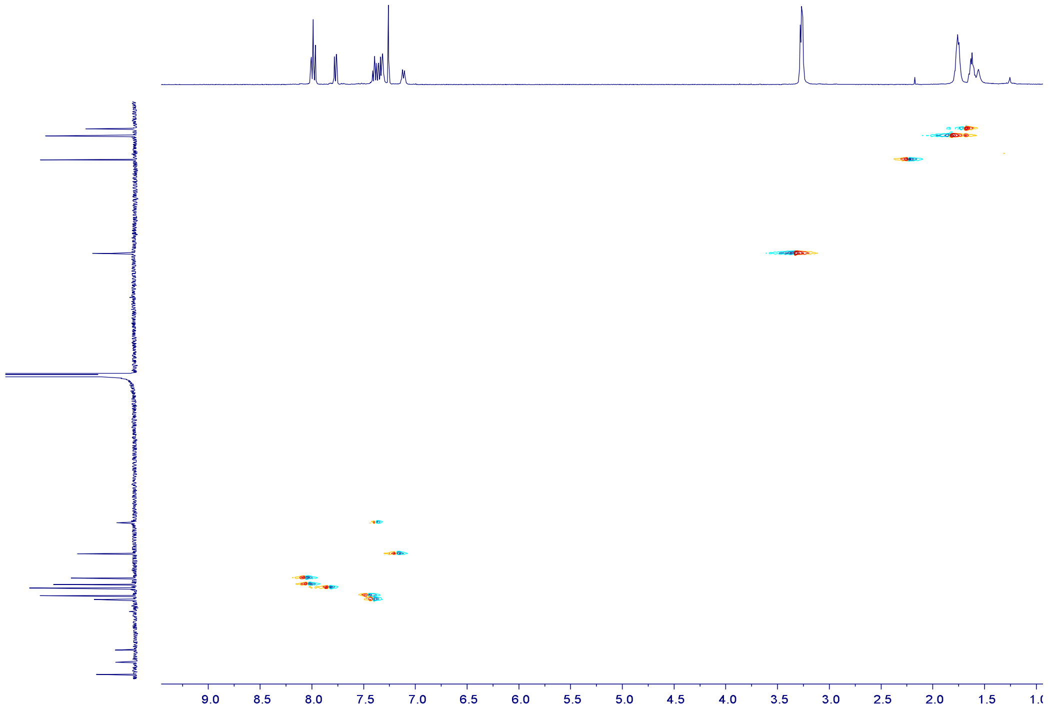

3x – HMBC ($CDCl_3$)

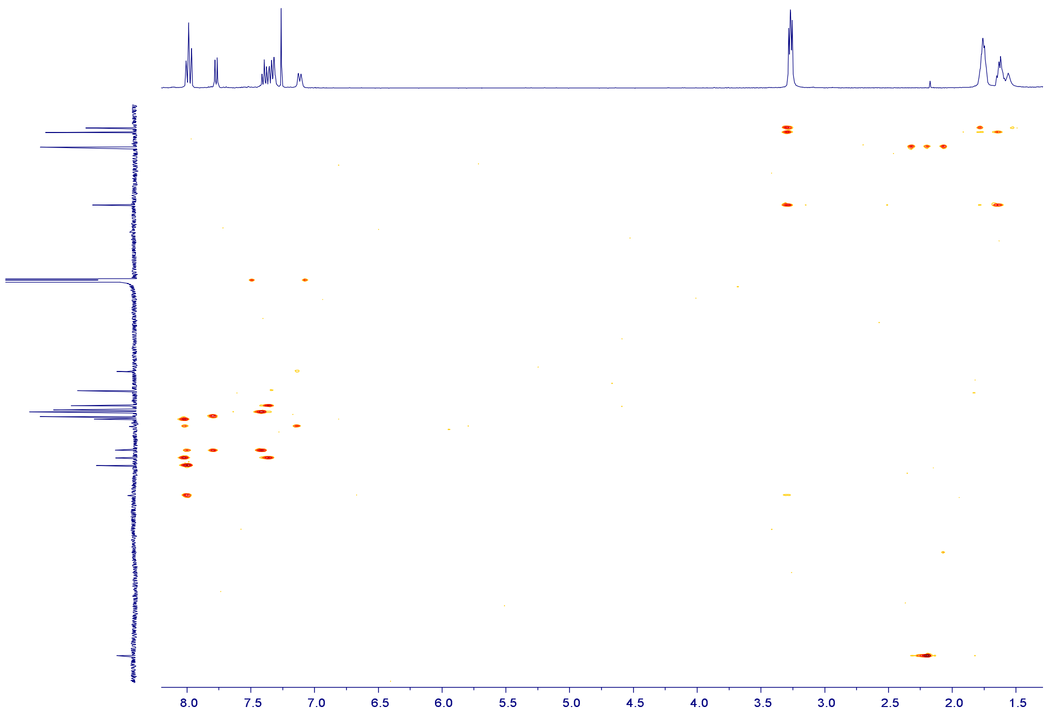

3y – ^{1}H NMR (400 MHz, $CDCl_3$)

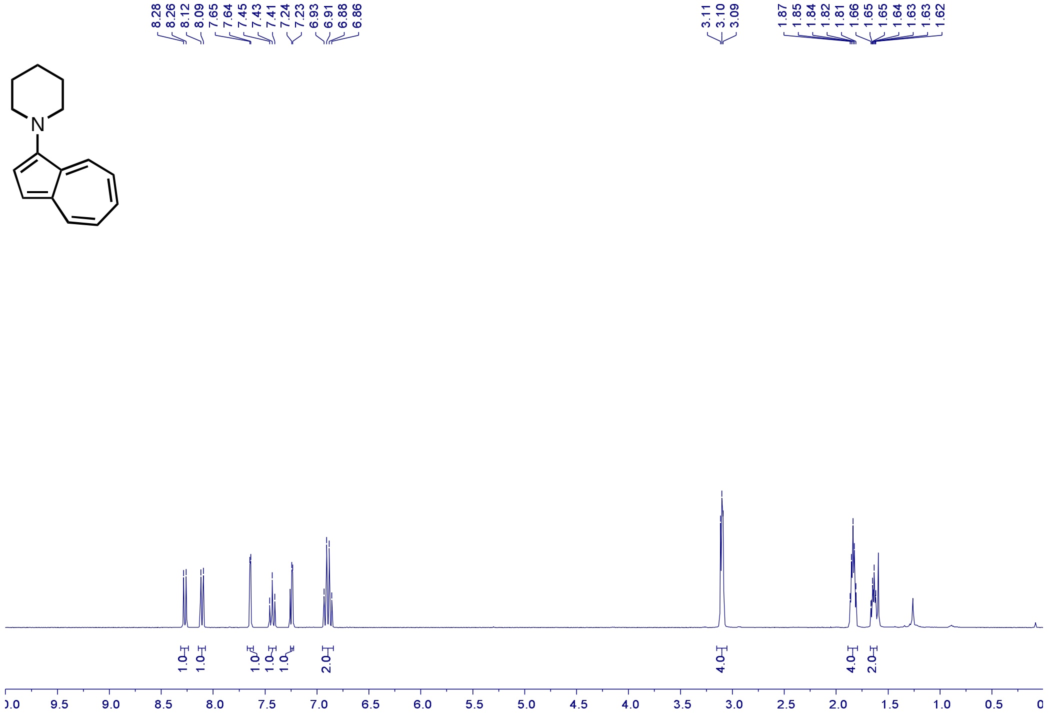

3y – ^{13}C NMR (101 MHz, $CDCl_3$)

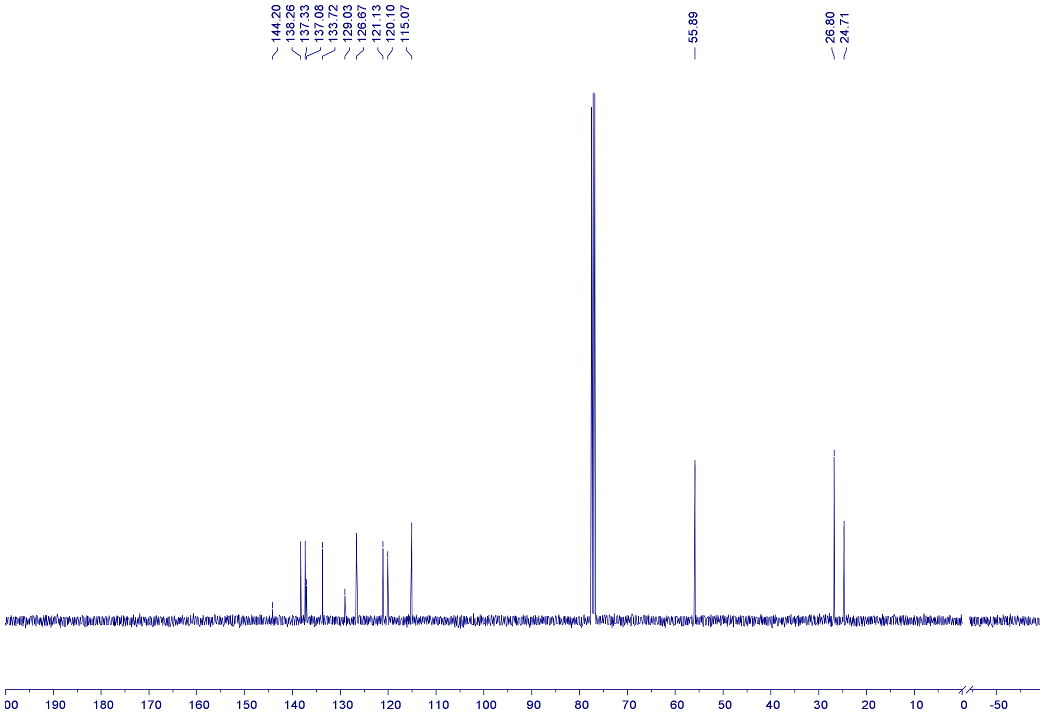

3z – ^{1}H NMR (500 MHz, $CDCl_3$)

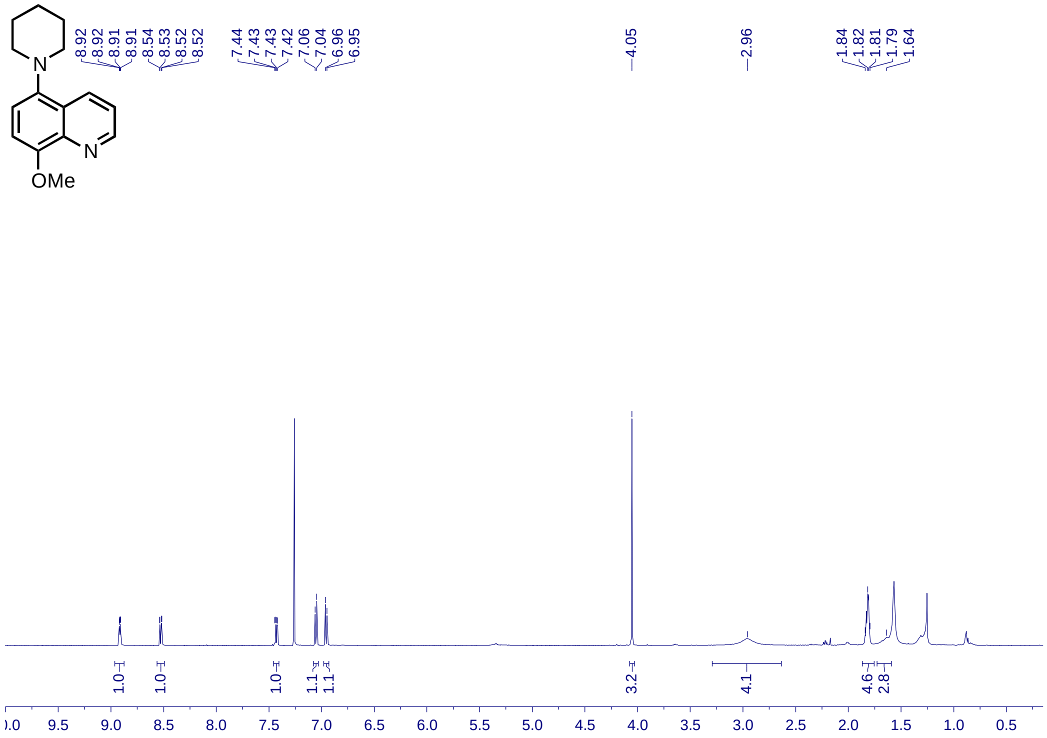

3z – ^{13}C NMR (126 MHz, $CDCl_3$)

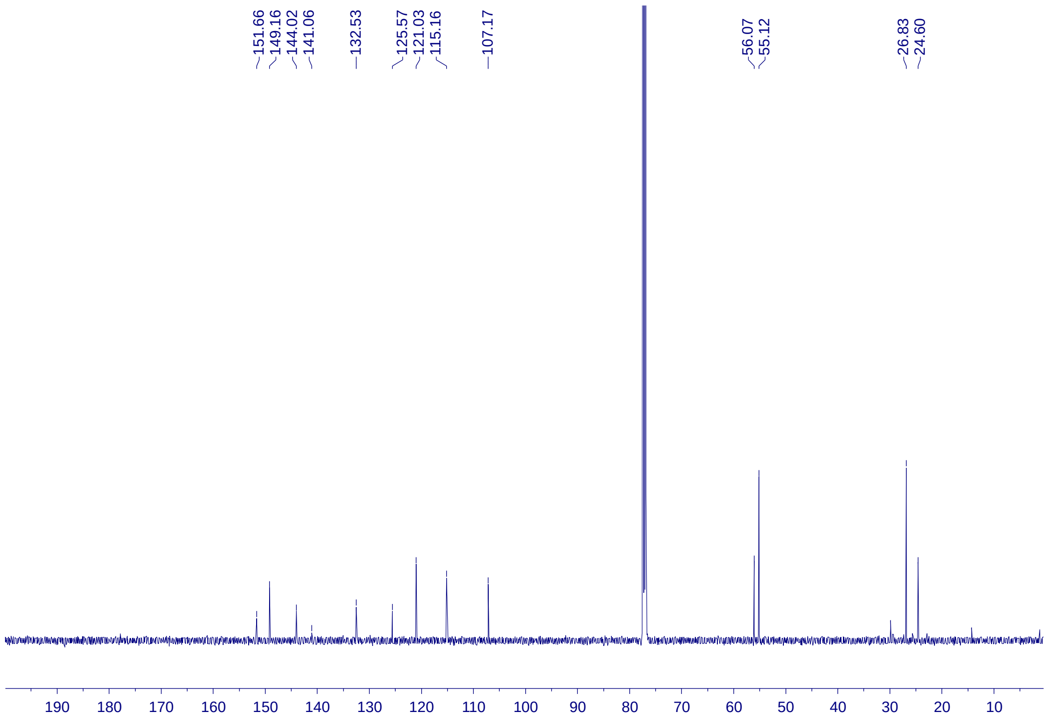

3aa – ^{1}H NMR (500 MHz, $CDCl_3$)

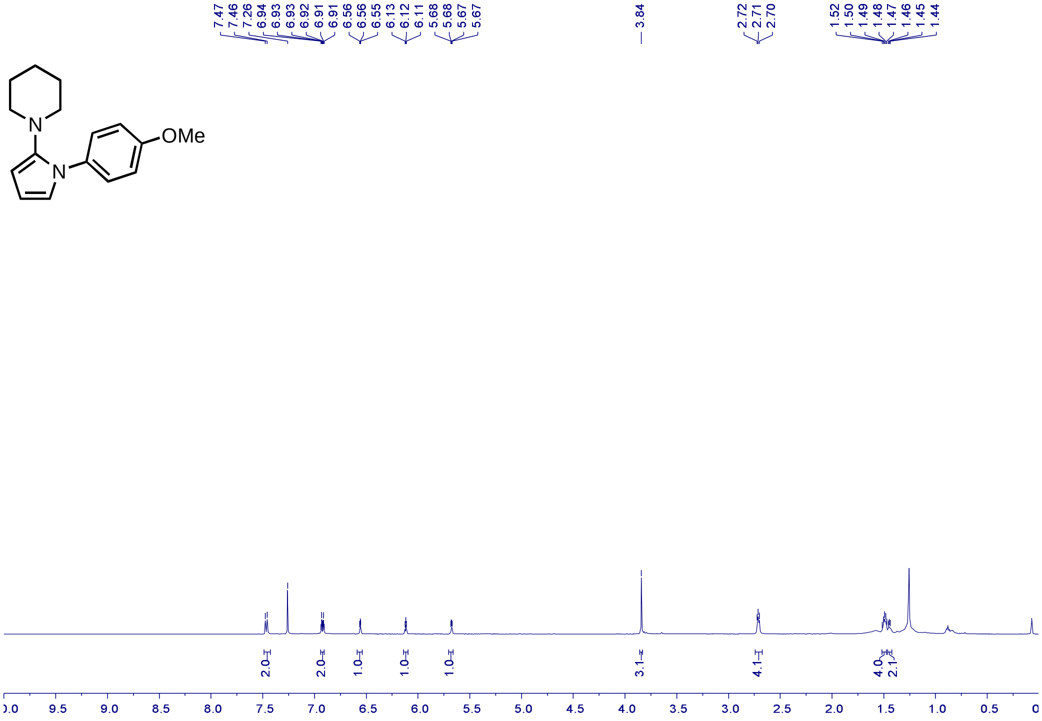

3aa – ^{13}C NMR (126 MHz, $CDCl_3$)

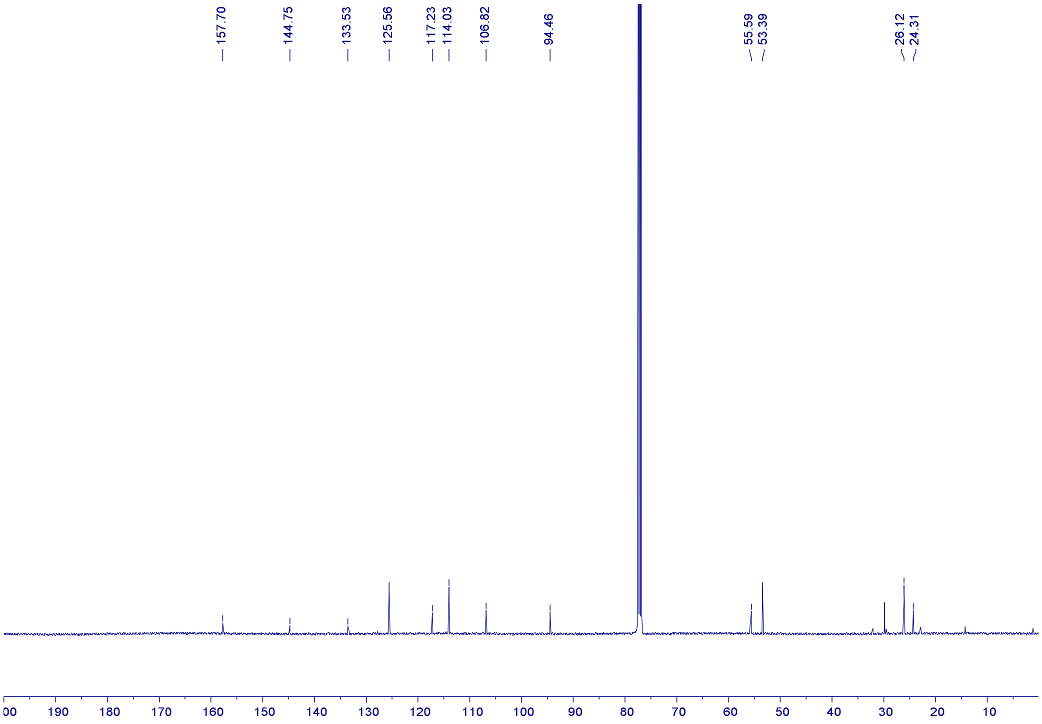

3ac – ^{1}H NMR (500 MHz, $CDCl_3$)

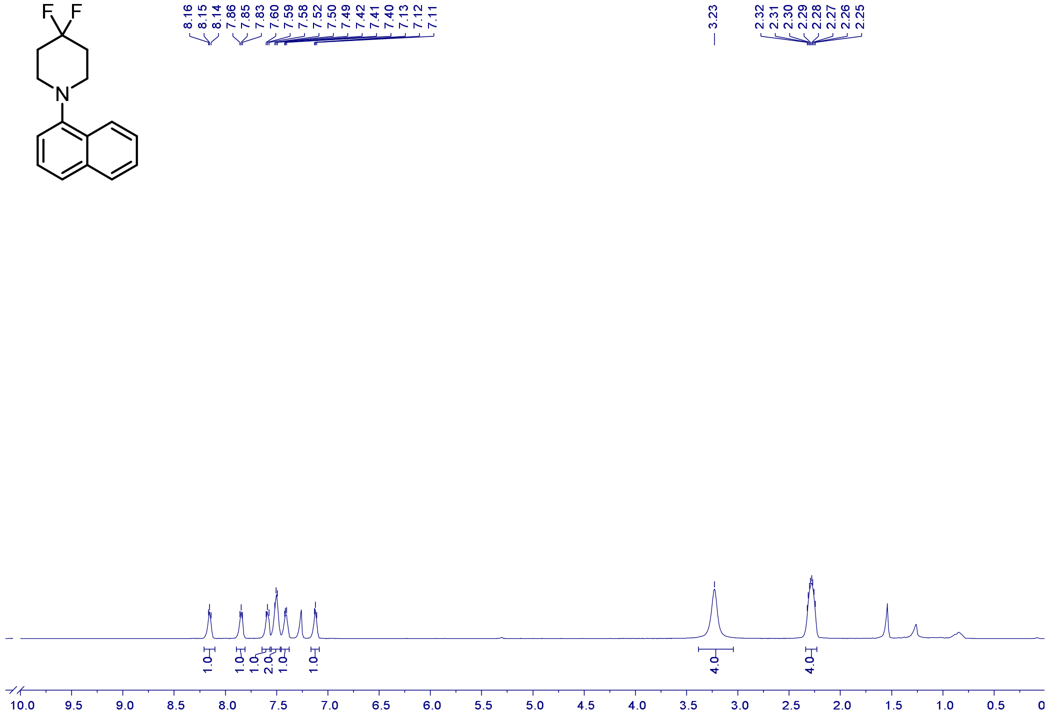

3ac – ^{13}C NMR (126 MHz, $CDCl_3$)

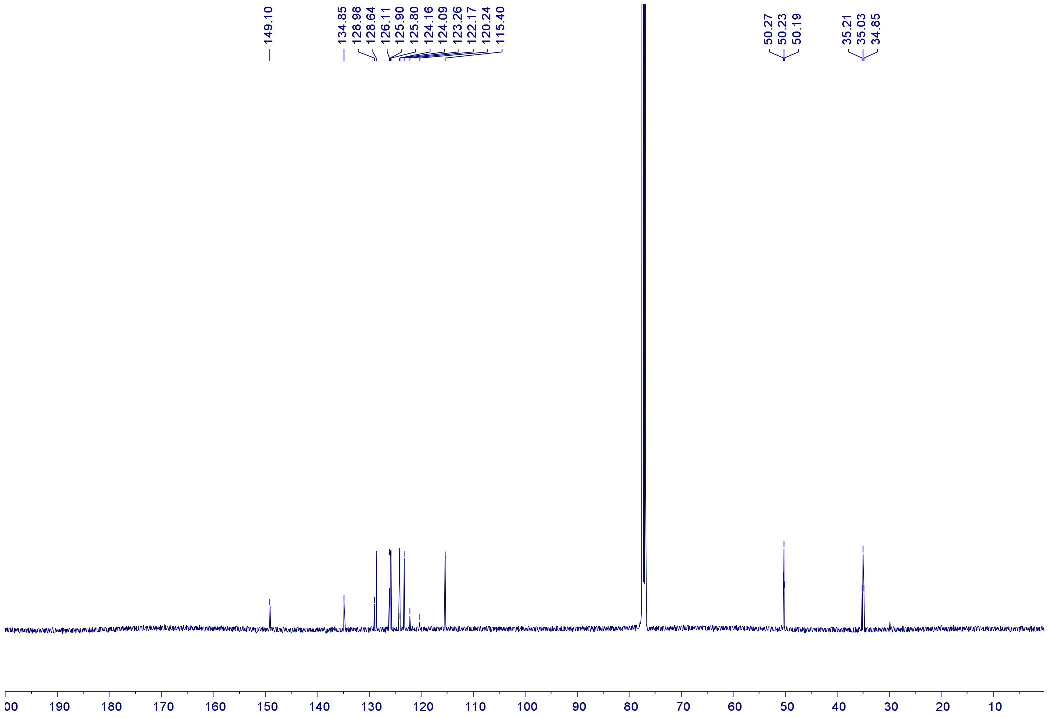

3ac – ^{19}F NMR (375 MHz, $CDCl_3$)

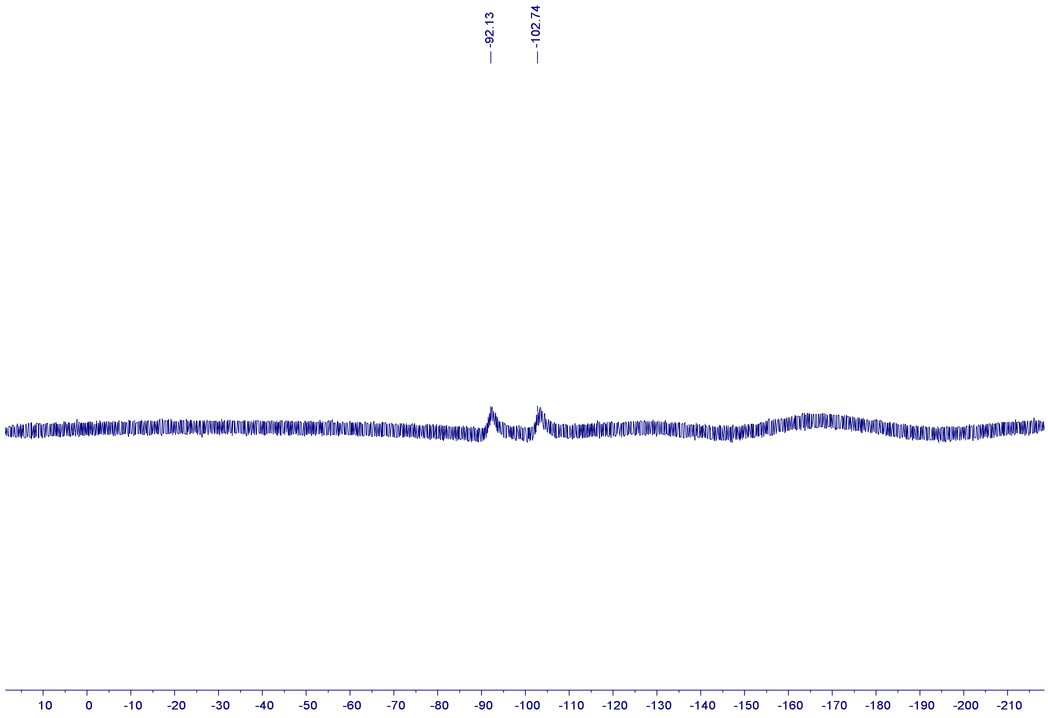

3ad – ^{1}H NMR (500 MHz, $CDCl_3$)

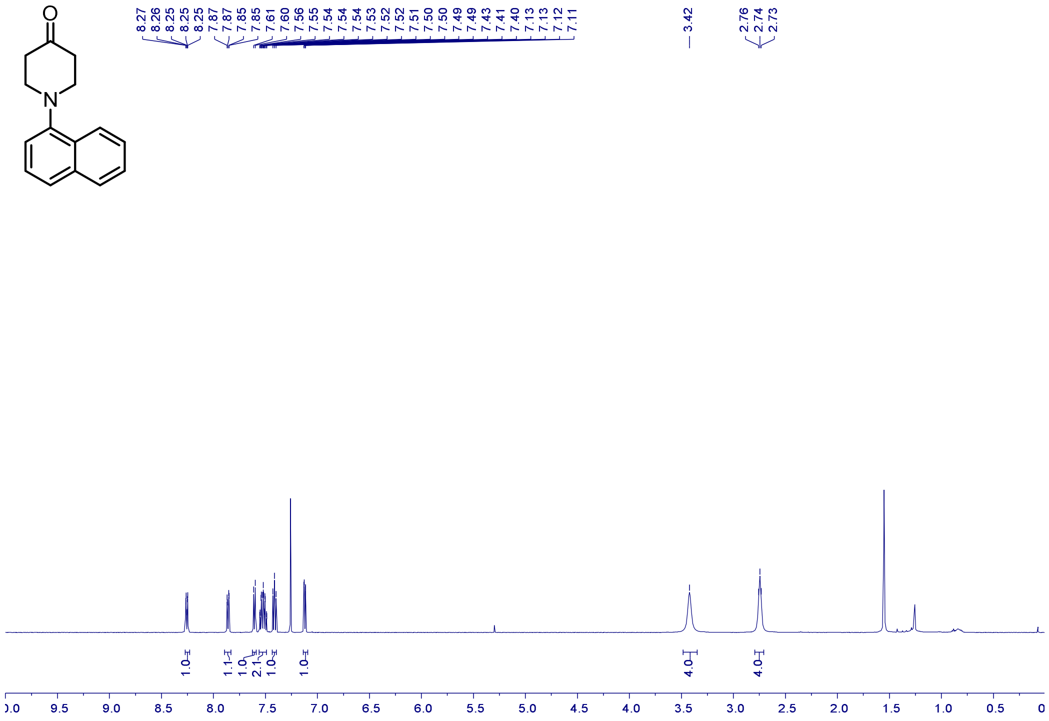

3ad – ^{13}C NMR (126 MHz, $CDCl_3$)

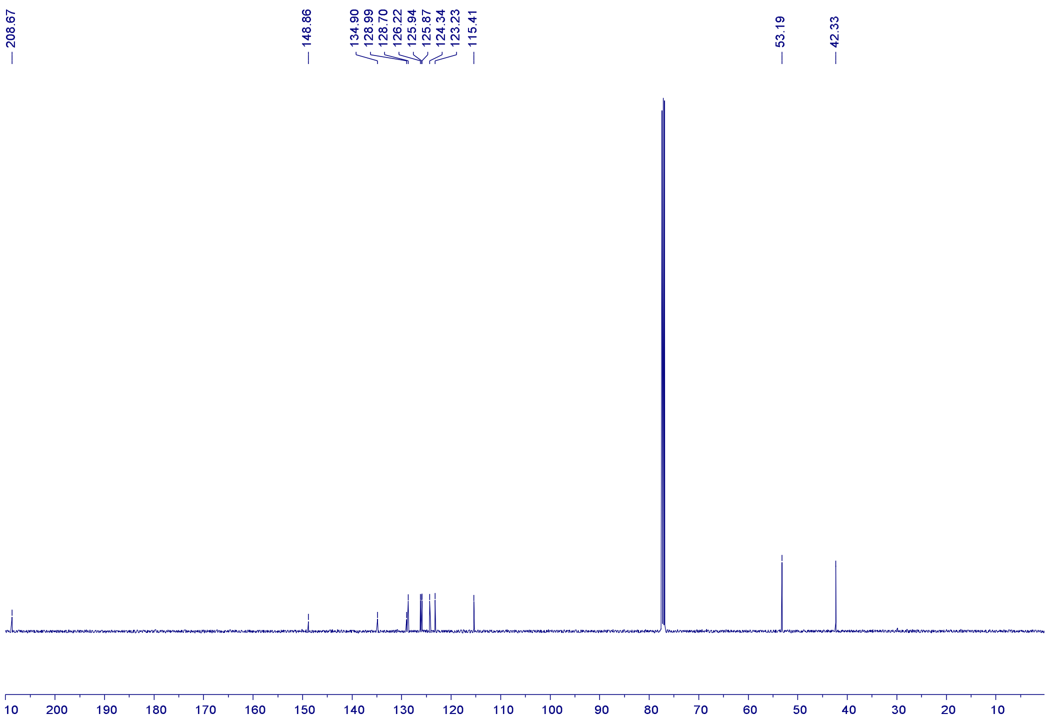

3ag – ^{1}H NMR (500 MHz, $CDCl_3$)

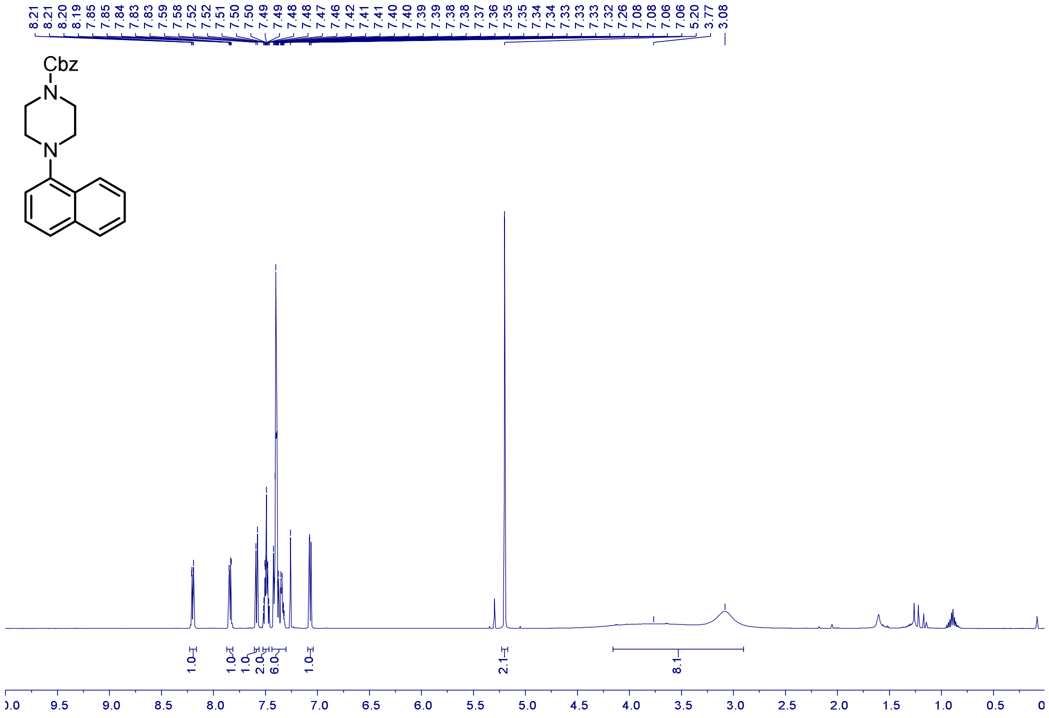

3ag – ^{13}C NMR (126 MHz, $CDCl_3$)

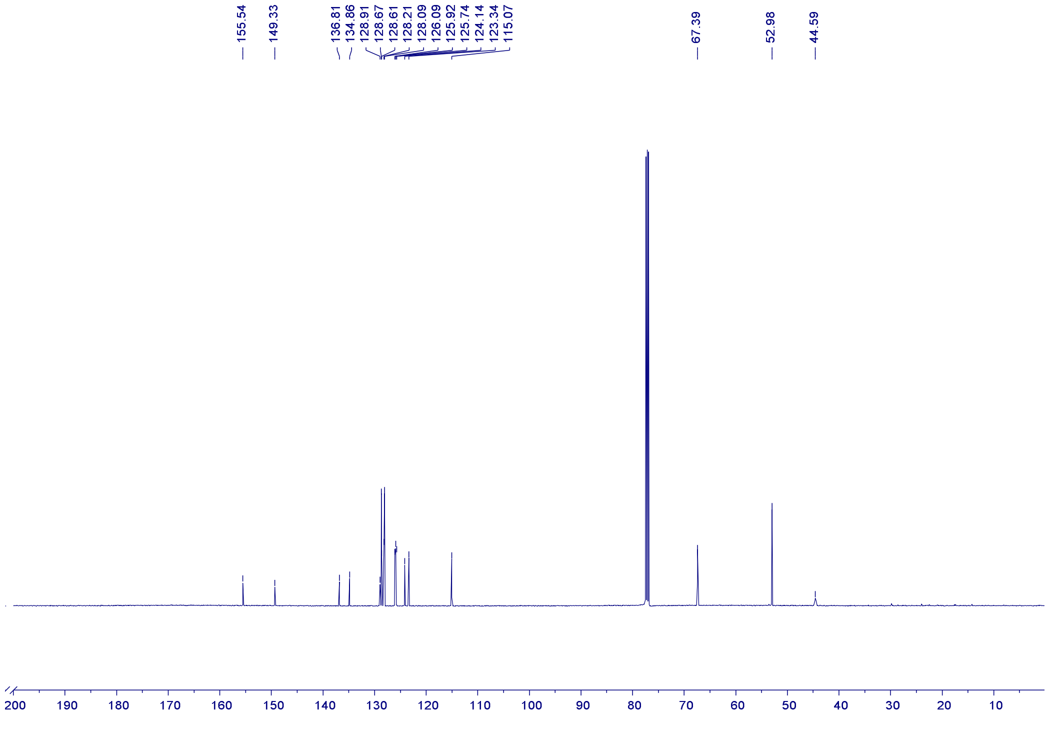

3ai – ^{1}H NMR (400 MHz, $CDCl_3$)

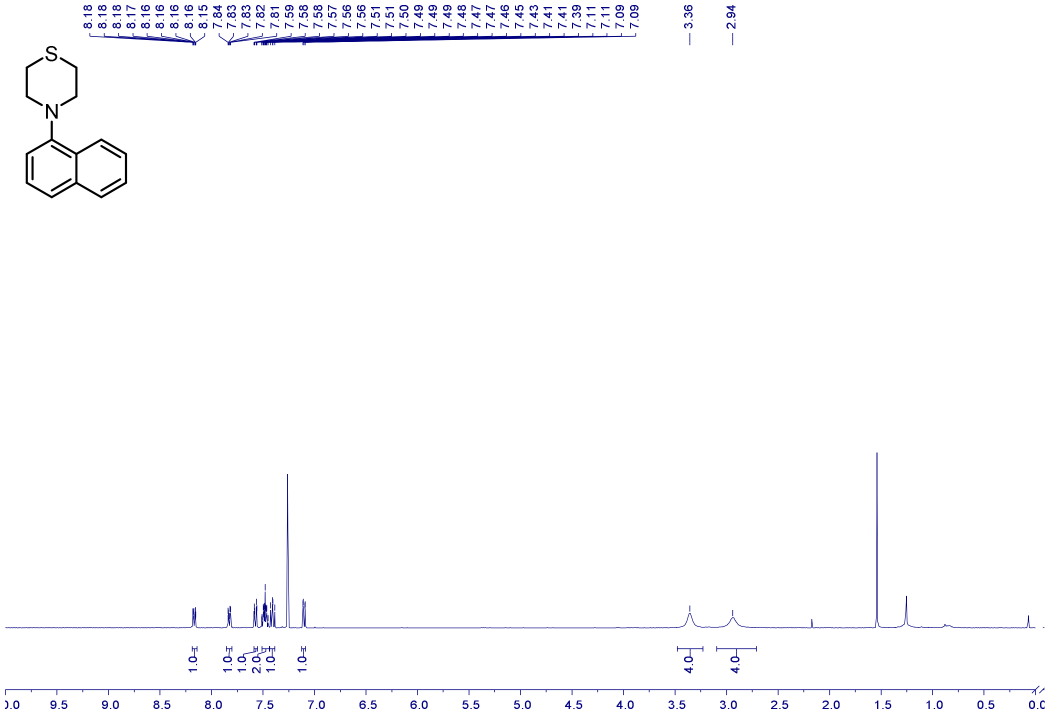

3ai – ^{13}C NMR (126 MHz, $CDCl_3$)

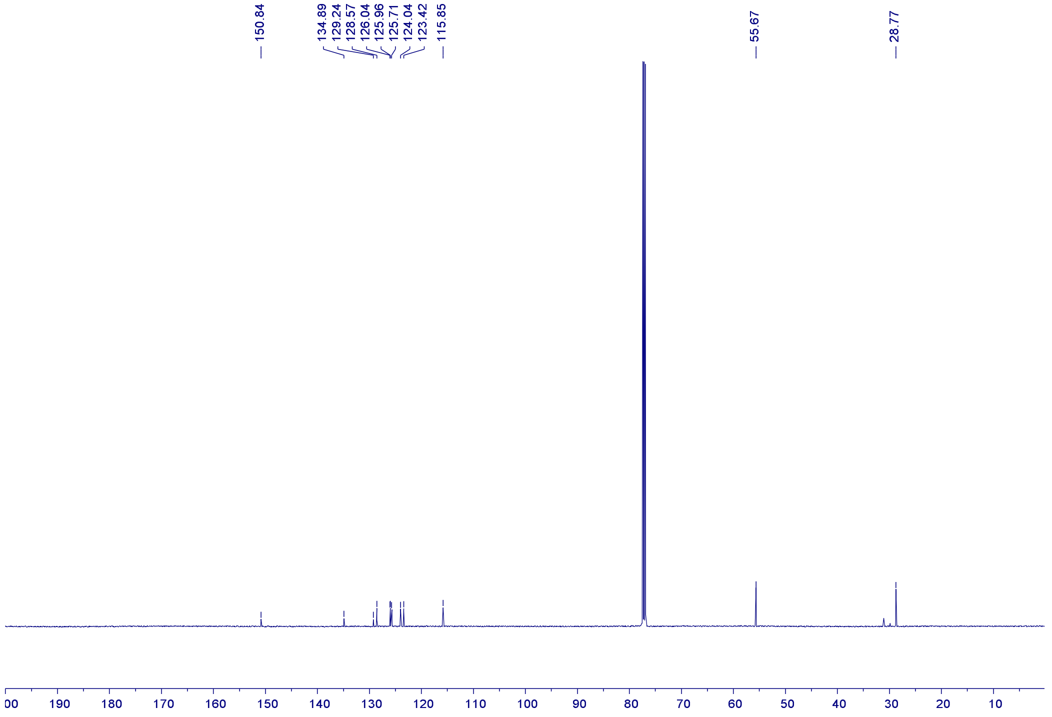

3aj – ^{1}H NMR (400 MHz, $CDCl_3$)

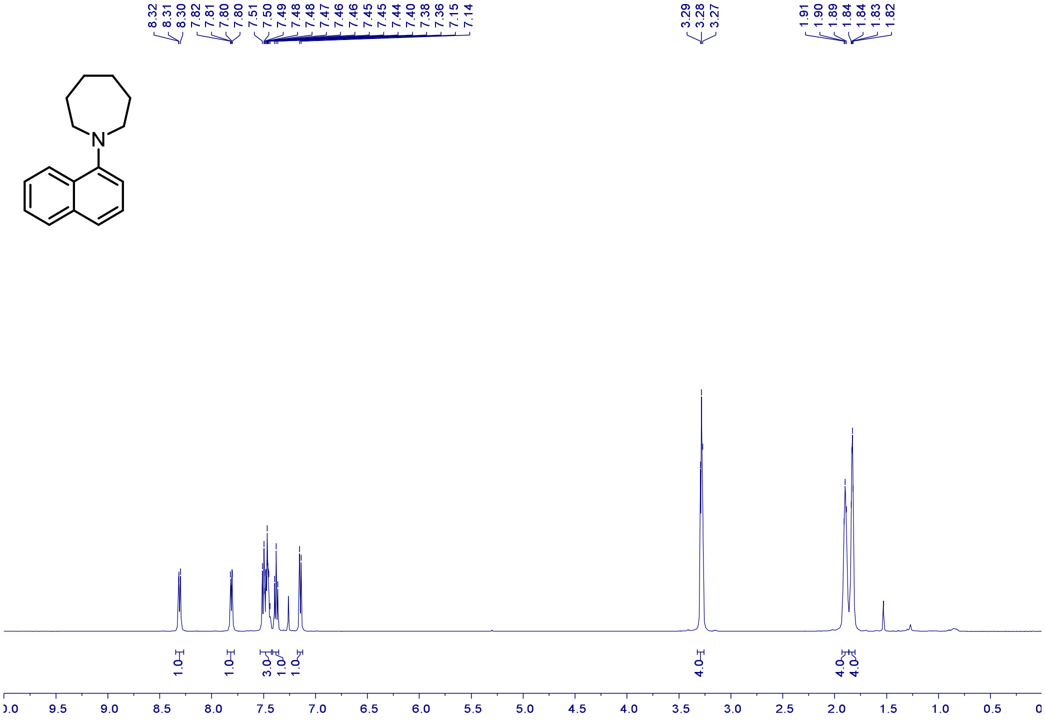

3aj – ^{13}C NMR (101 MHz, $CDCl_3$)

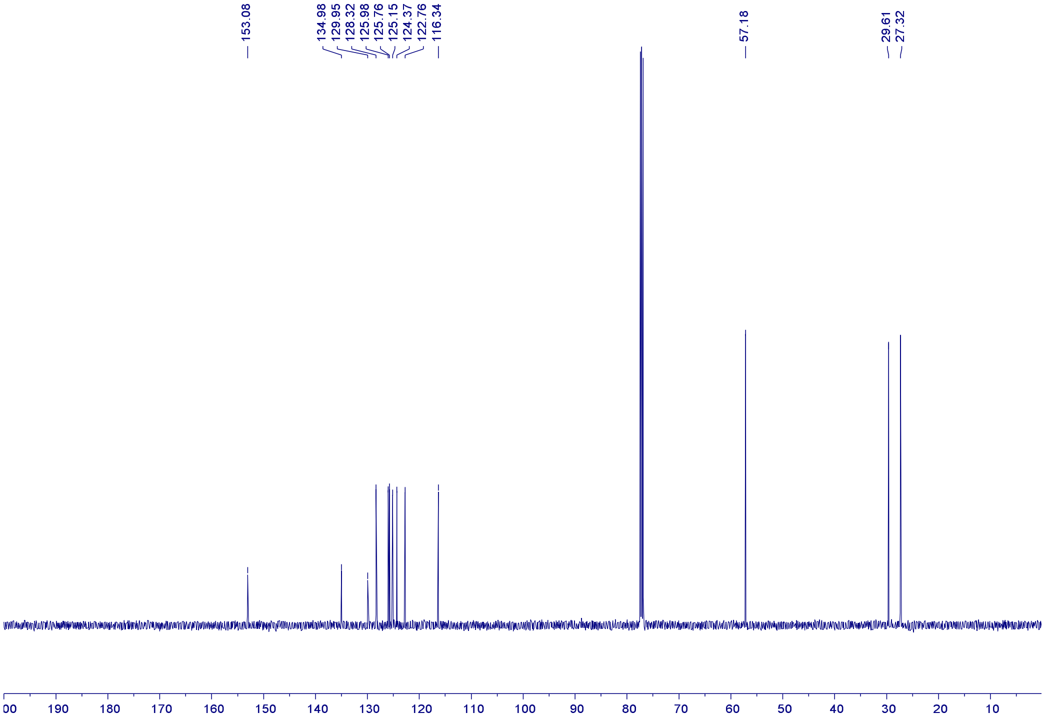

3an – ^{1}H NMR (500 MHz, $CDCl_3$)

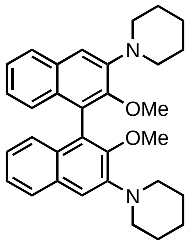

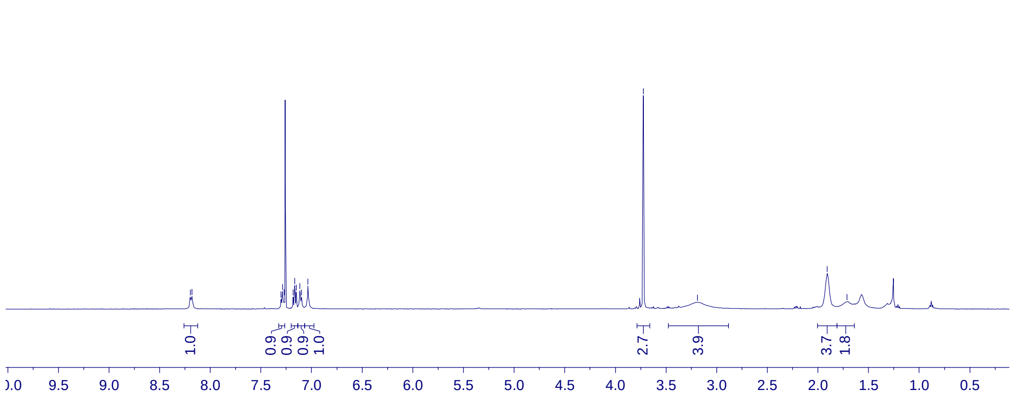

3an – ^{13}C NMR (126 MHz, $CDCl_3$)

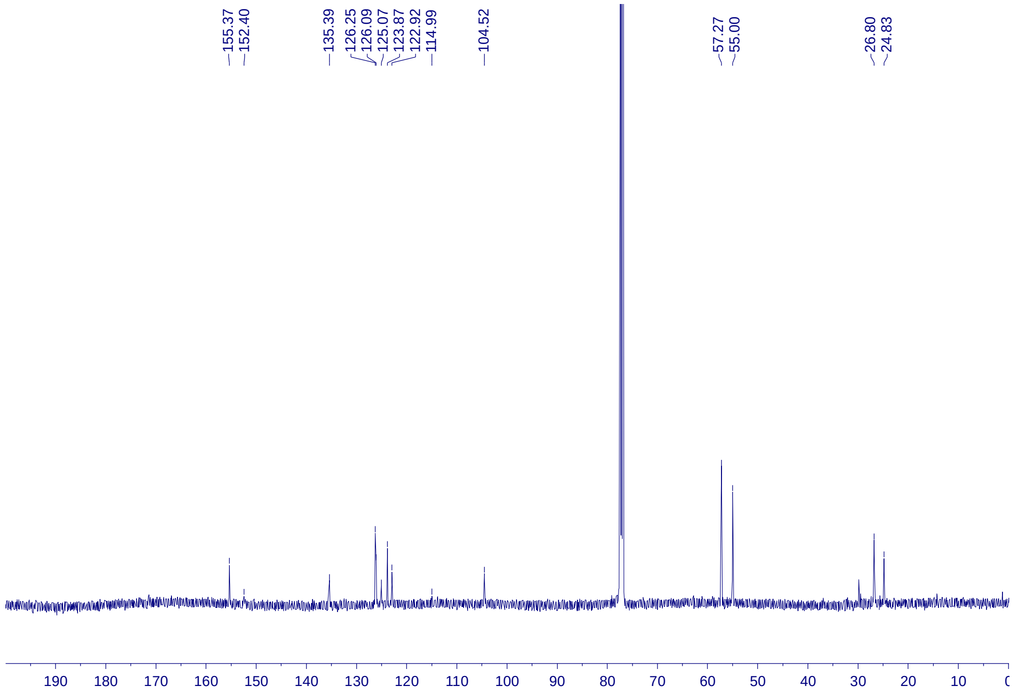

3ao – ^{1}H NMR (500 MHz, $CDCl_3$)

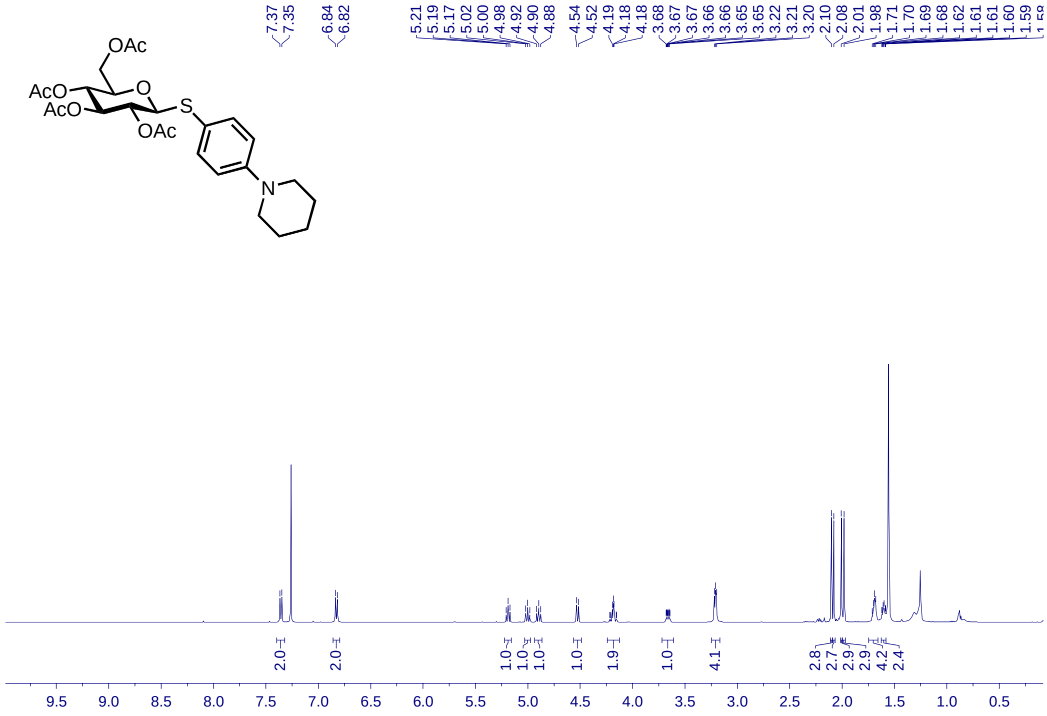

3ao – ^{13}C NMR (126 MHz, $CDCl_3$)

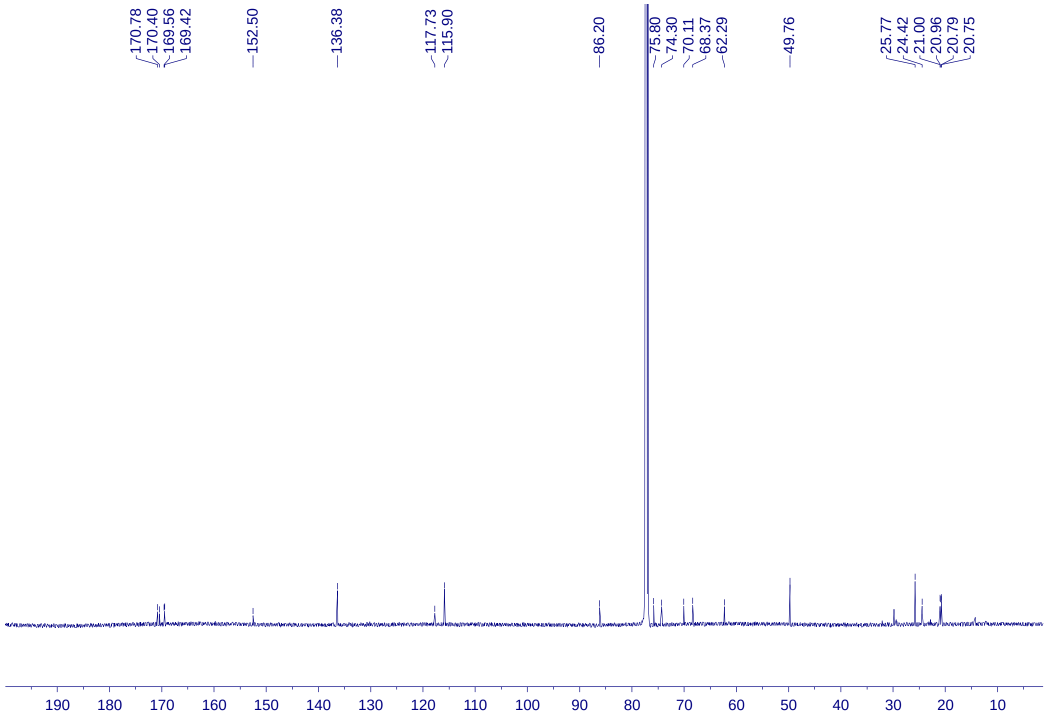

3ap – ^{1}H NMR (400 MHz, $CDCl_3$)

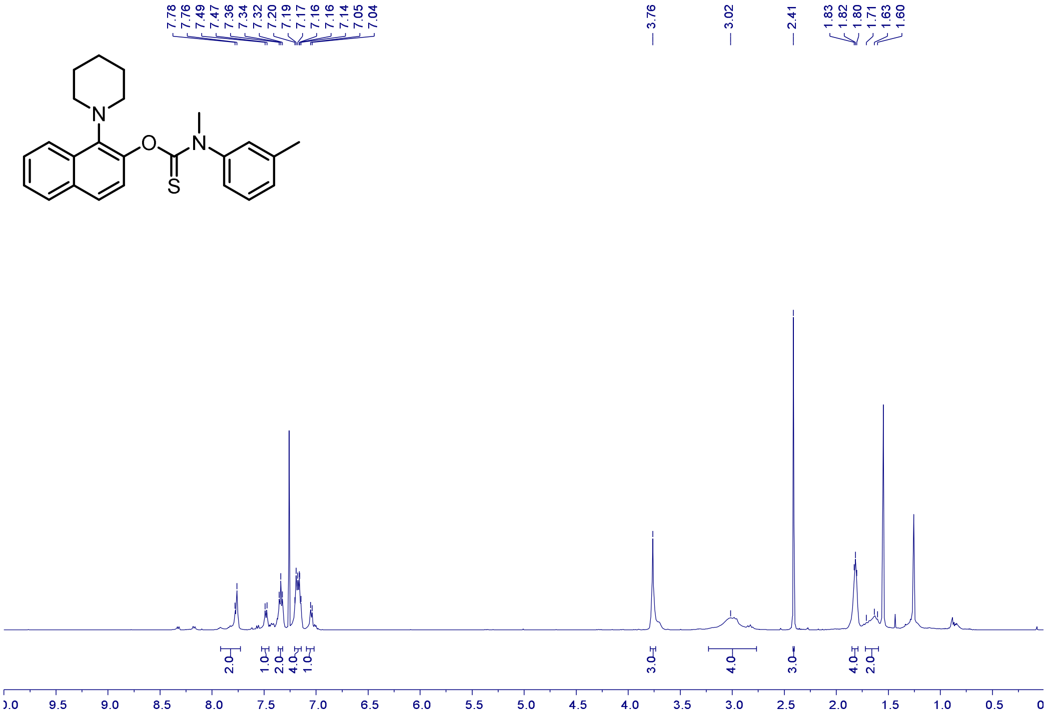

3ap – ^{13}C NMR (101 MHz, $CDCl_3$)

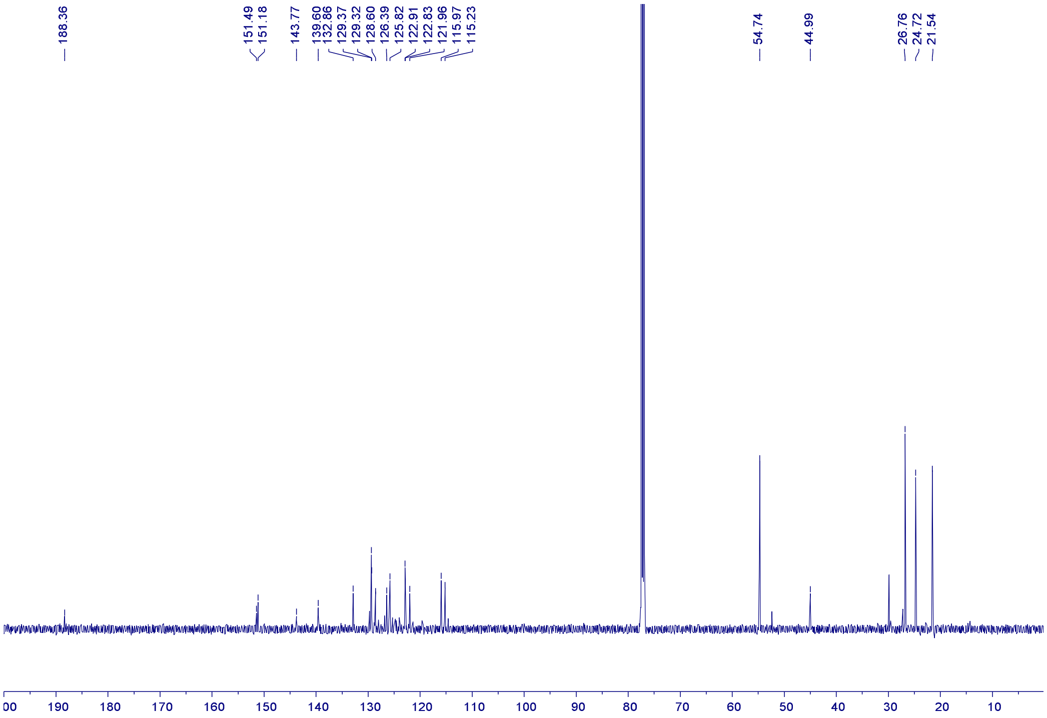

3aq and 3aq' – ^{1}H NMR (400 MHz, $CDCl_3$)

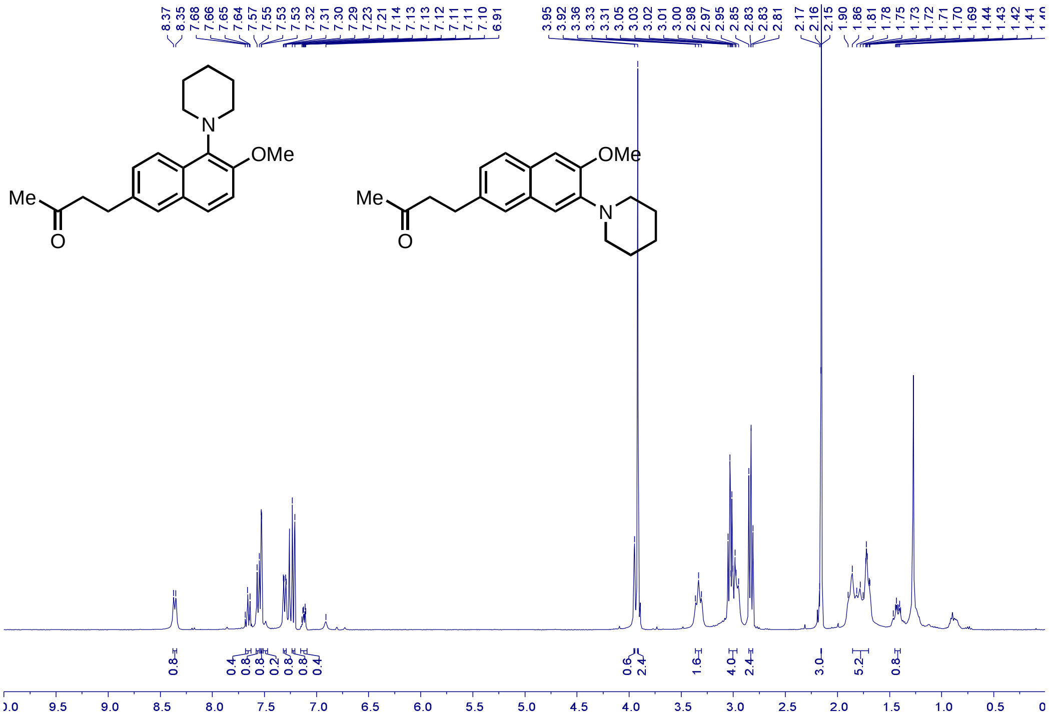

3aq and 3aq' – ^{13}C NMR (101 MHz, $CDCl_3$)

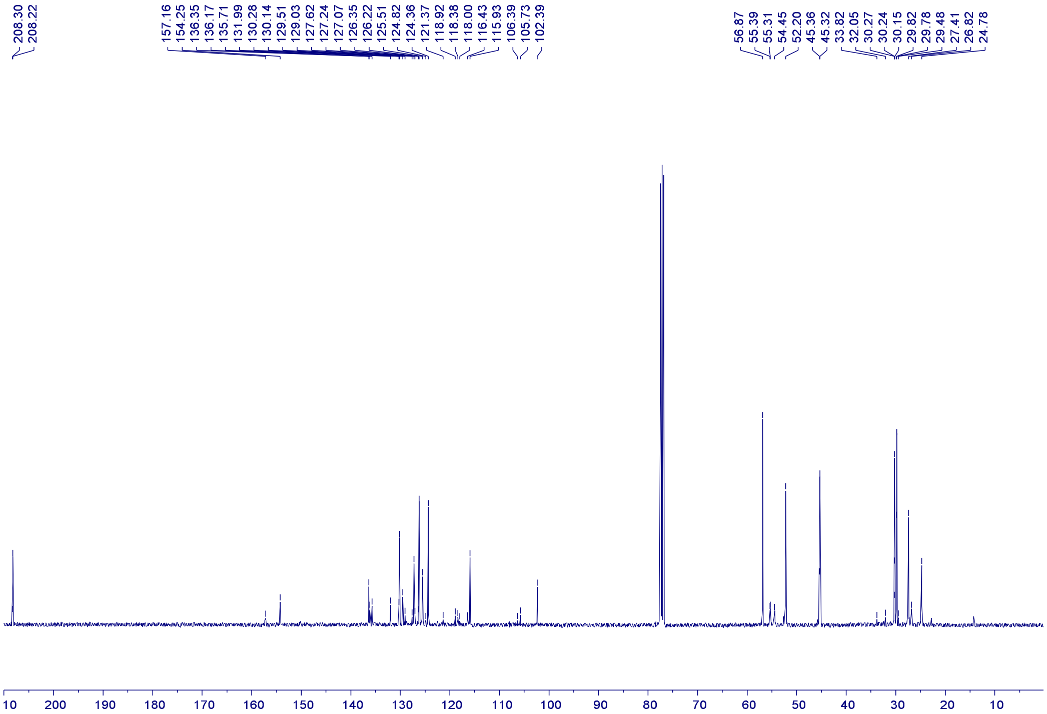

3aq and 3aq' – COSY ($CDCl_3$)

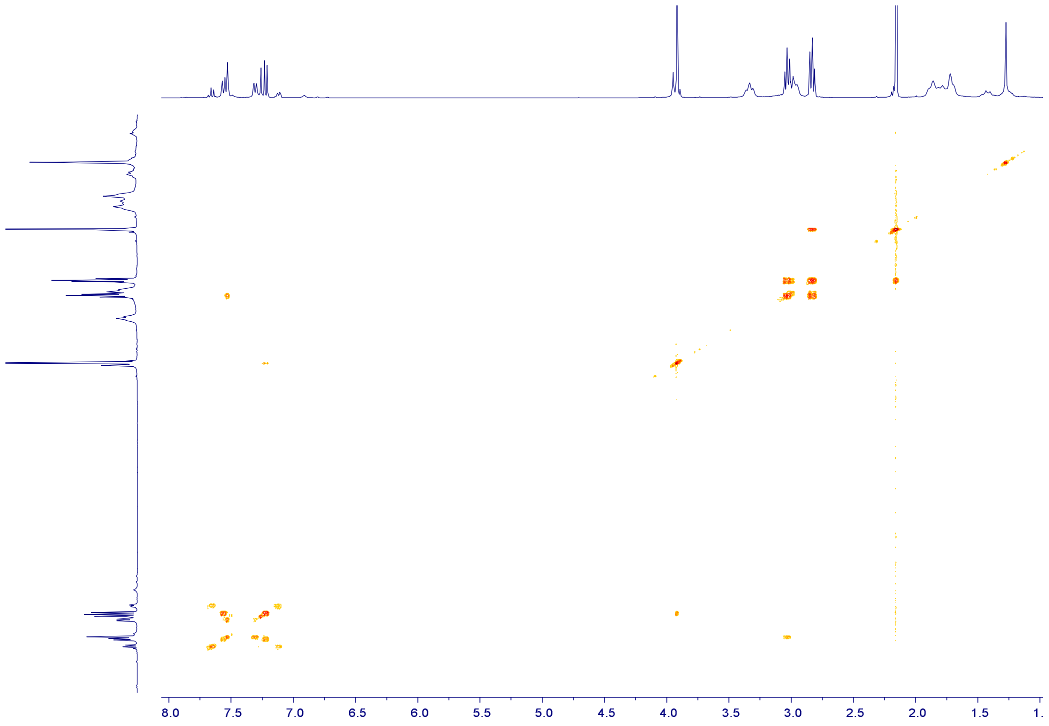

3aq and 3aq' – HSQC ($CDCl_3$)

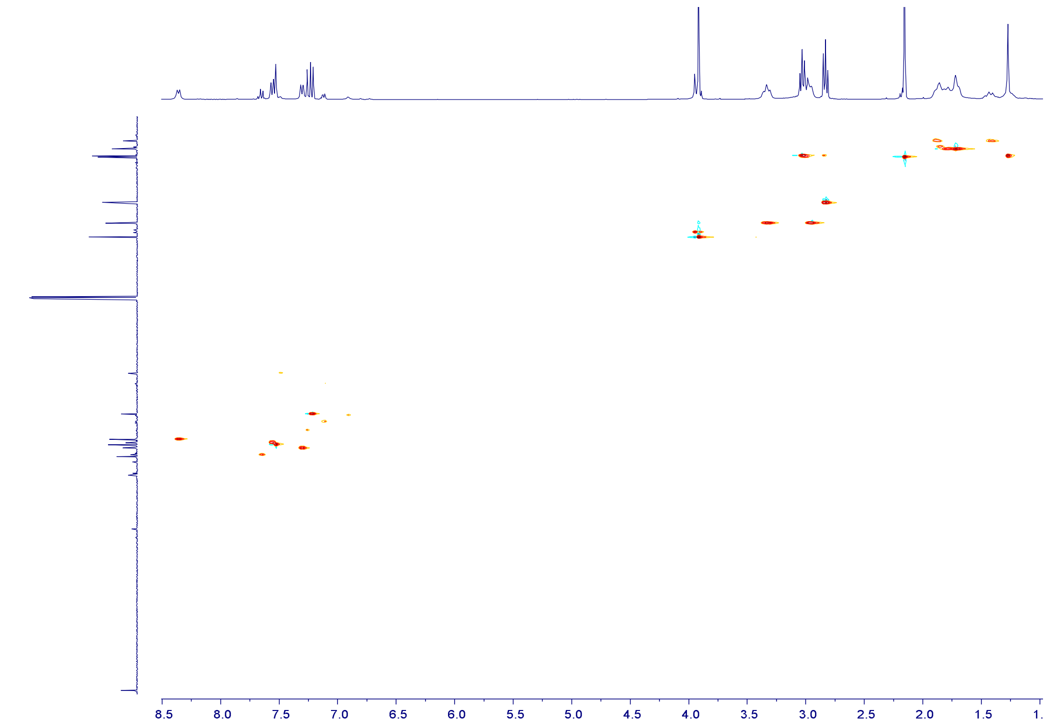

3aq and 3aq' – HMBC ($CDCl_3$)

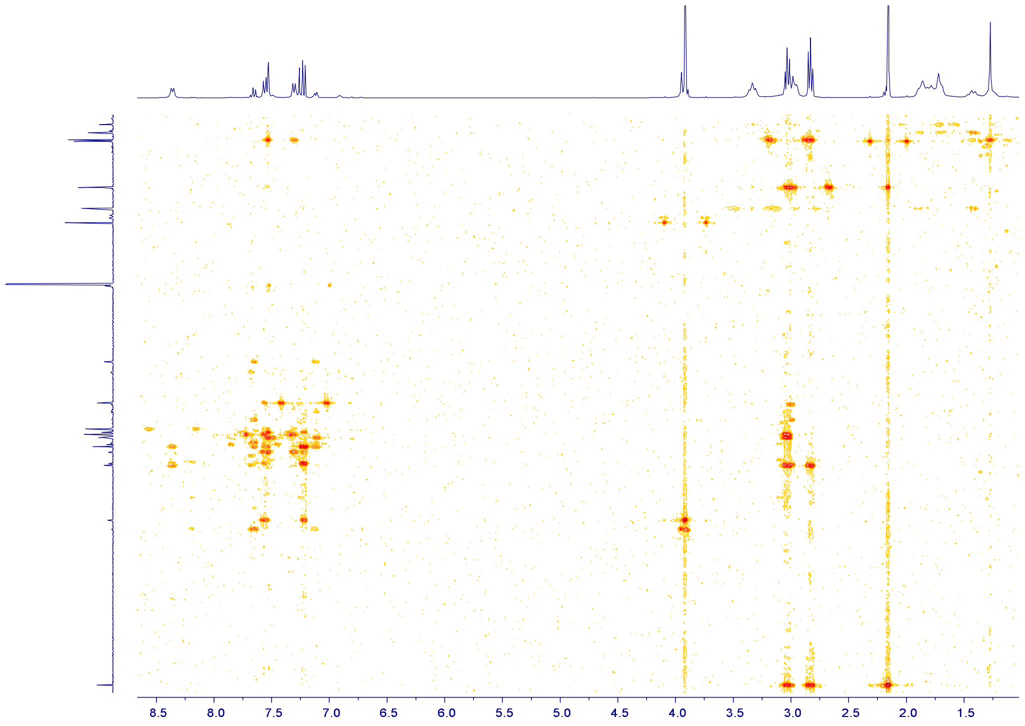

3ar – ^{1}H NMR (400 MHz, $CDCl_3$)

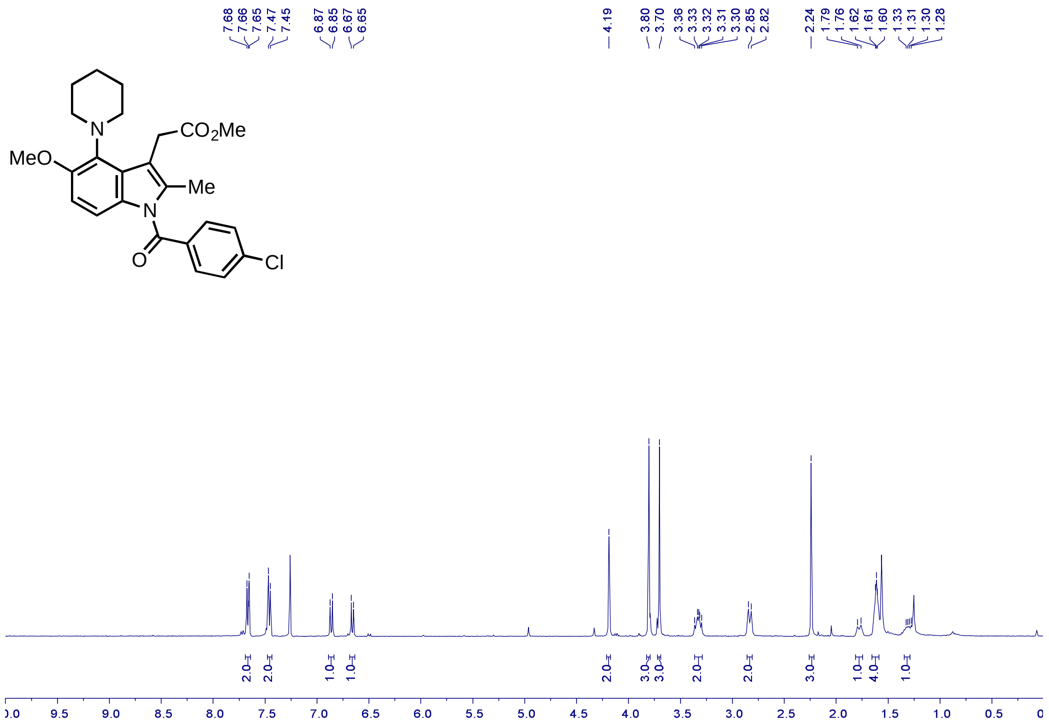

3ar – ^{13}C NMR (101 MHz, $CDCl_3$)

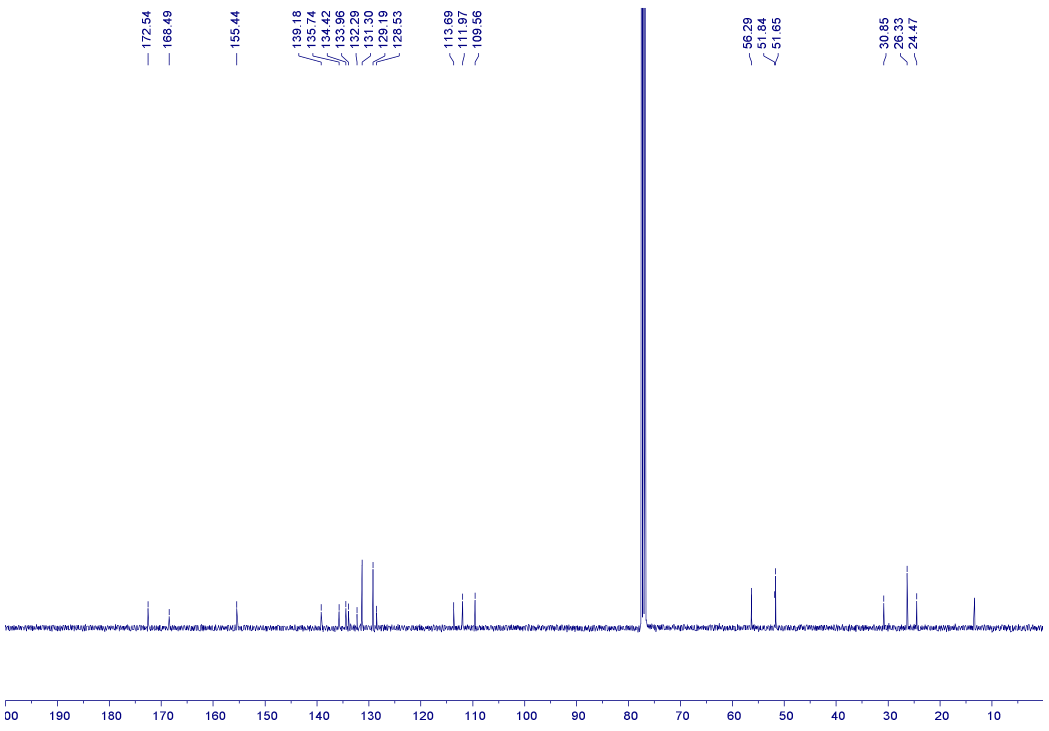

3as – ^{1}H NMR (400 MHz, $CDCl_3$)

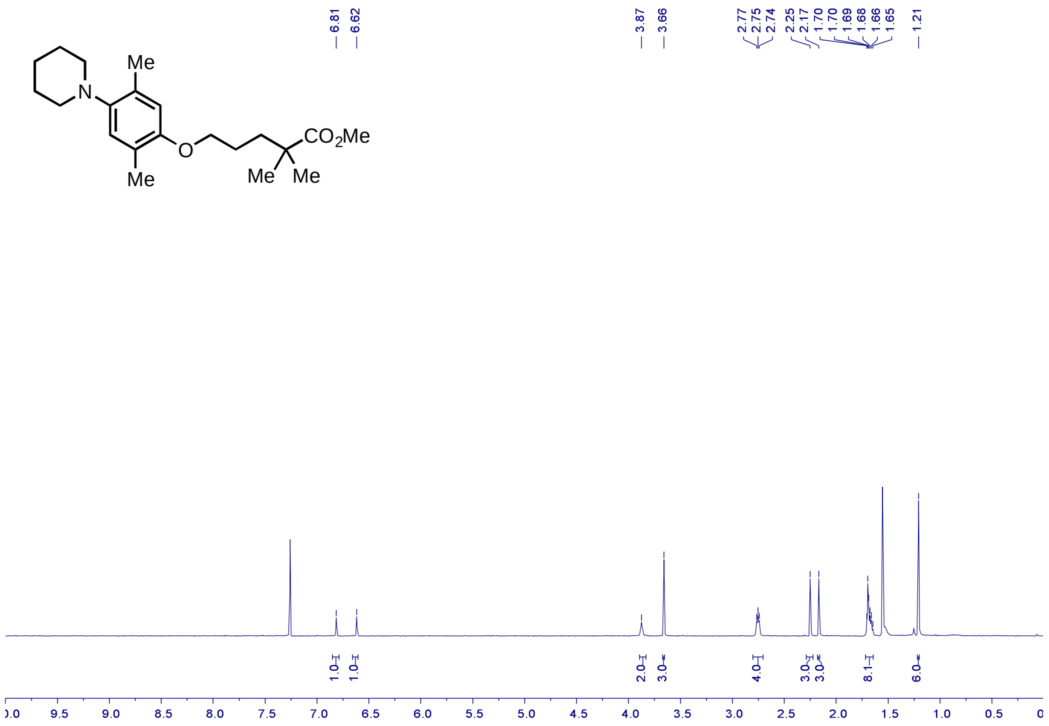

3as – ^{13}C NMR (126 MHz, $CDCl_3$)

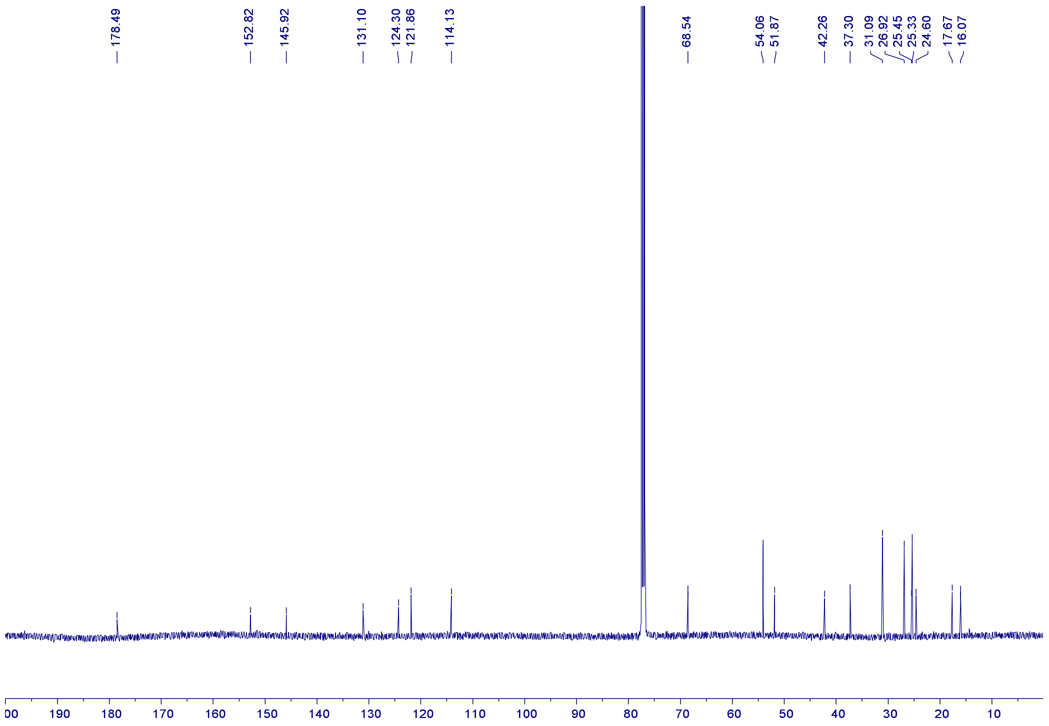

3at – ^{1}H NMR (500 MHz, $CDCl_3$)

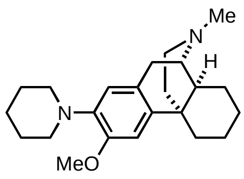

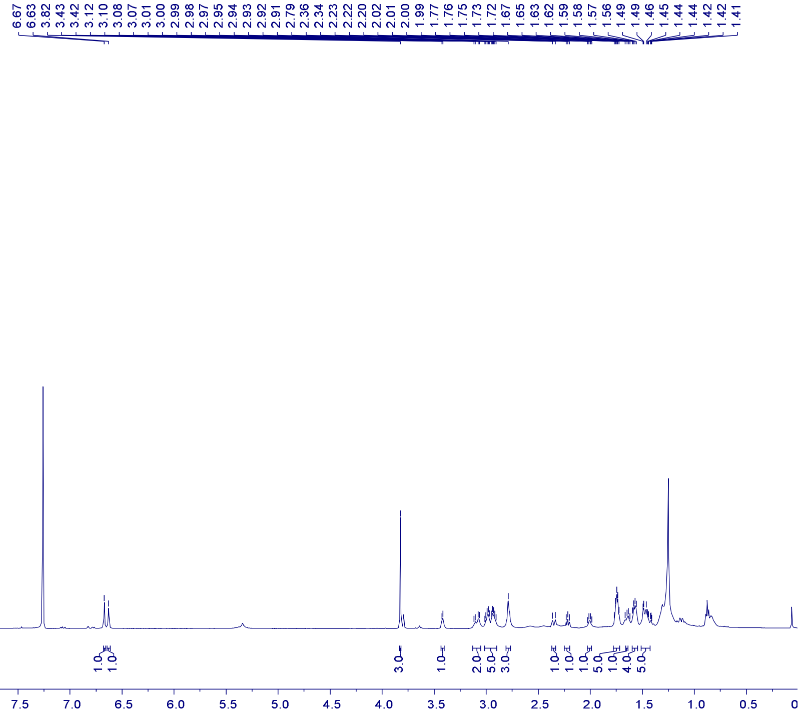

3at – ^{13}C NMR (150 MHz, $CDCl_3$)

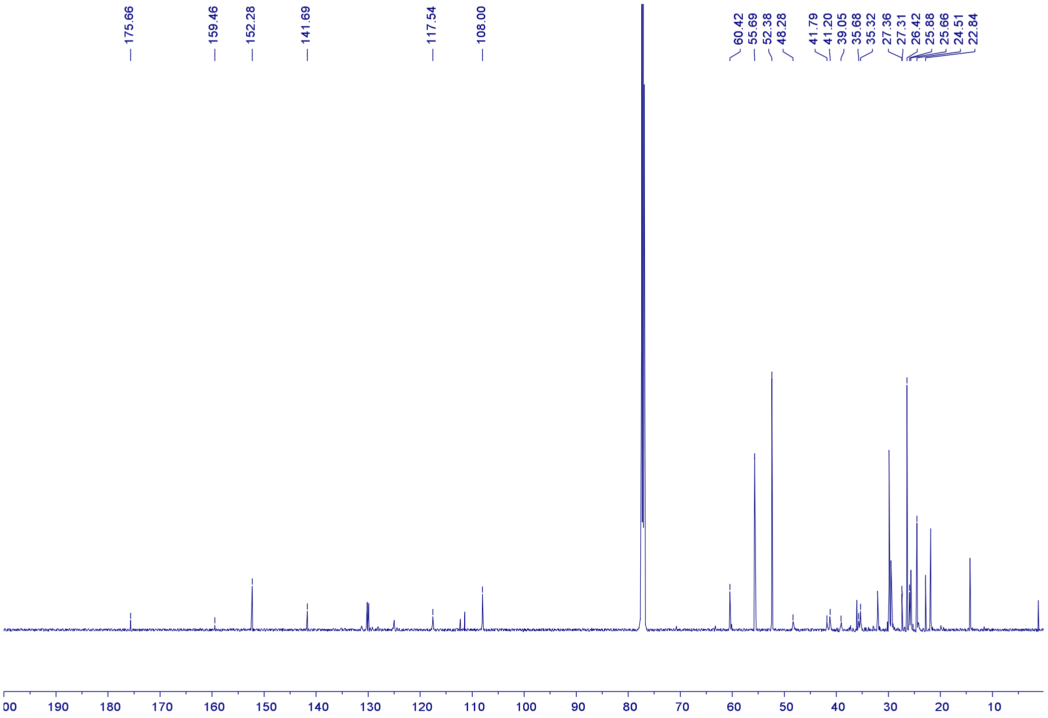

3au – ^{1}H NMR (500 MHz, $CDCl_3$)

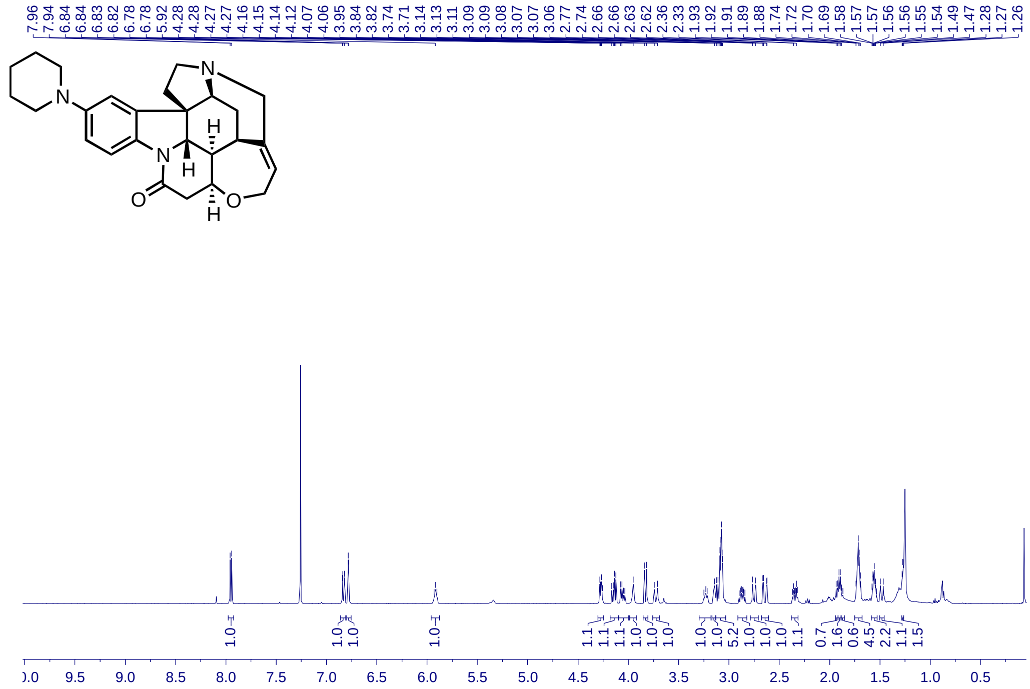

3au – ^{13}C NMR (126 MHz, $CDCl_3$)

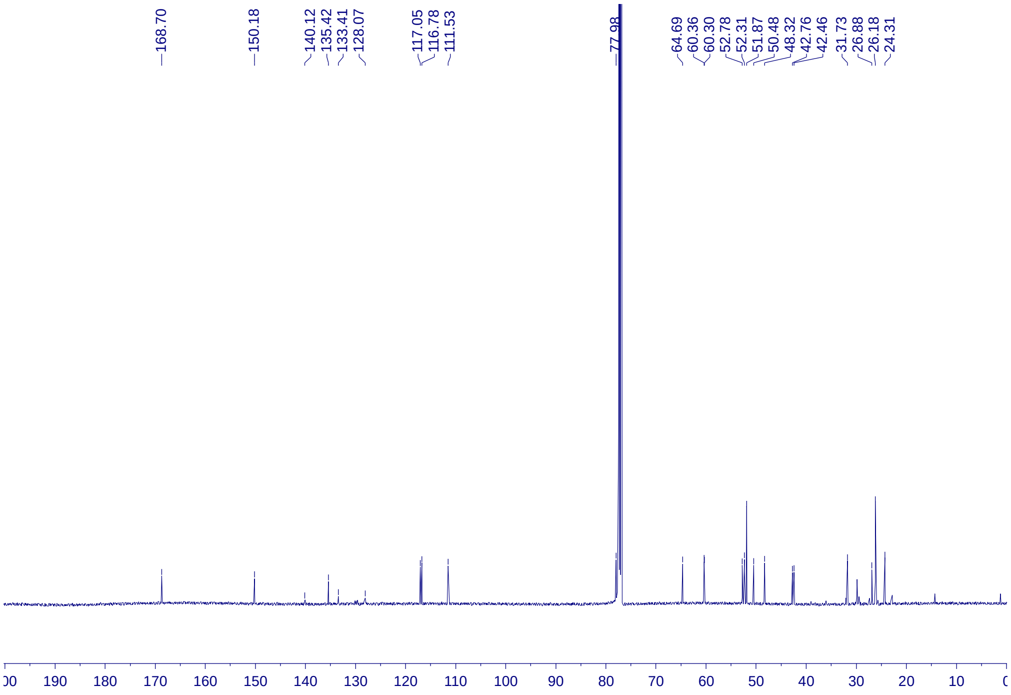

3av – ^{1}H NMR (500 MHz, $CDCl_3$)

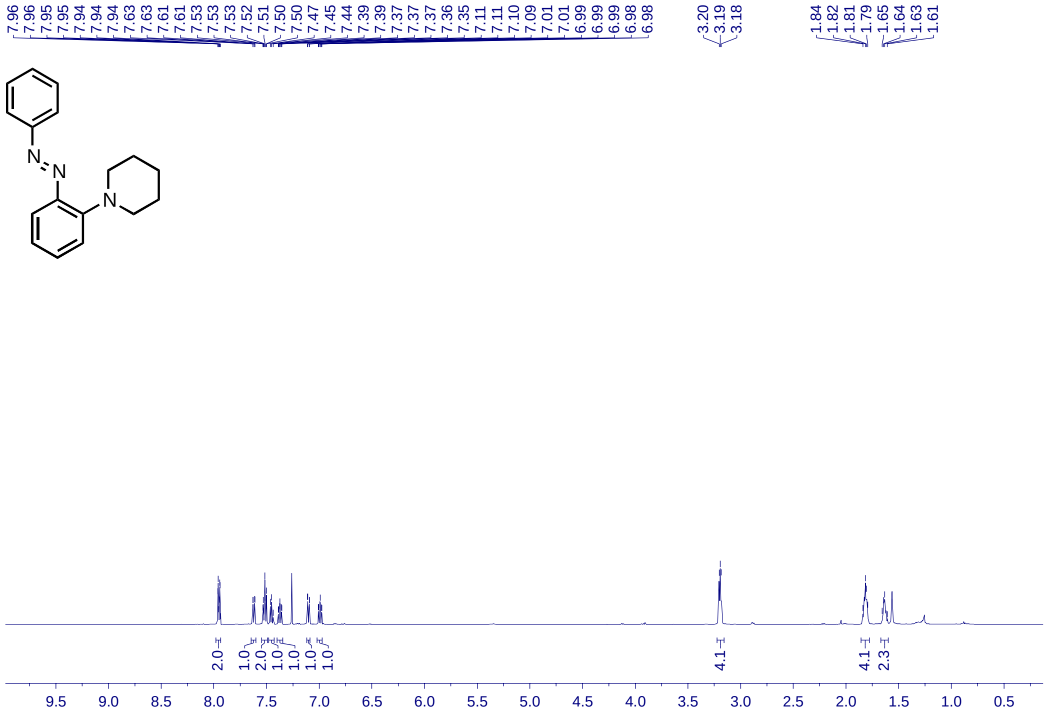

3av – ^{13}C NMR (126 MHz, $CDCl_3$)

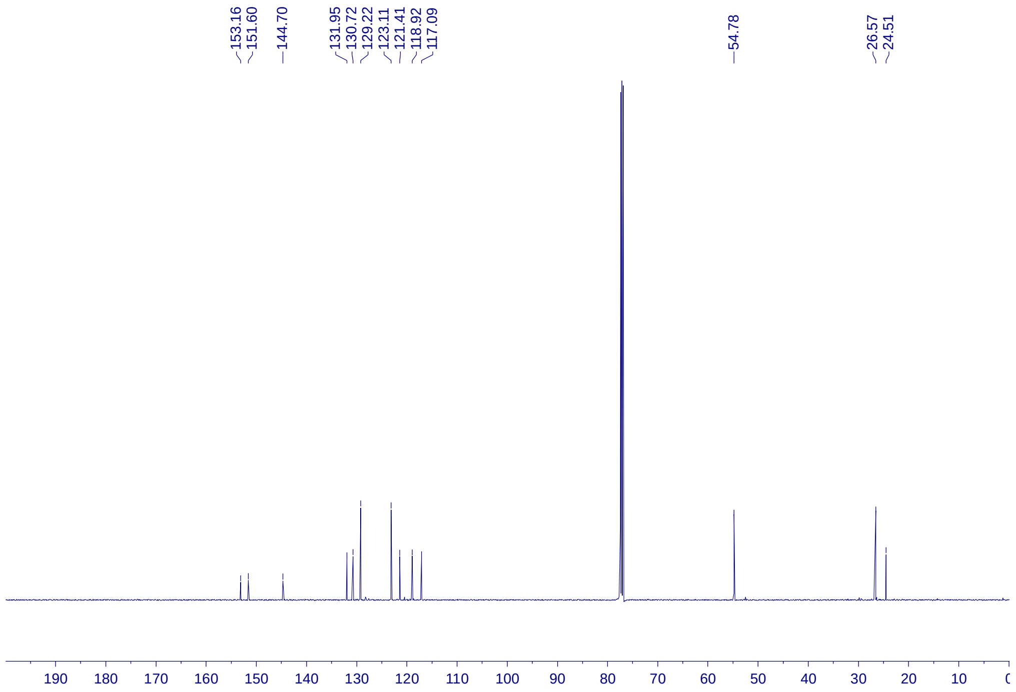

3aw – ^{1}H NMR (500 MHz, $CDCl_3$)

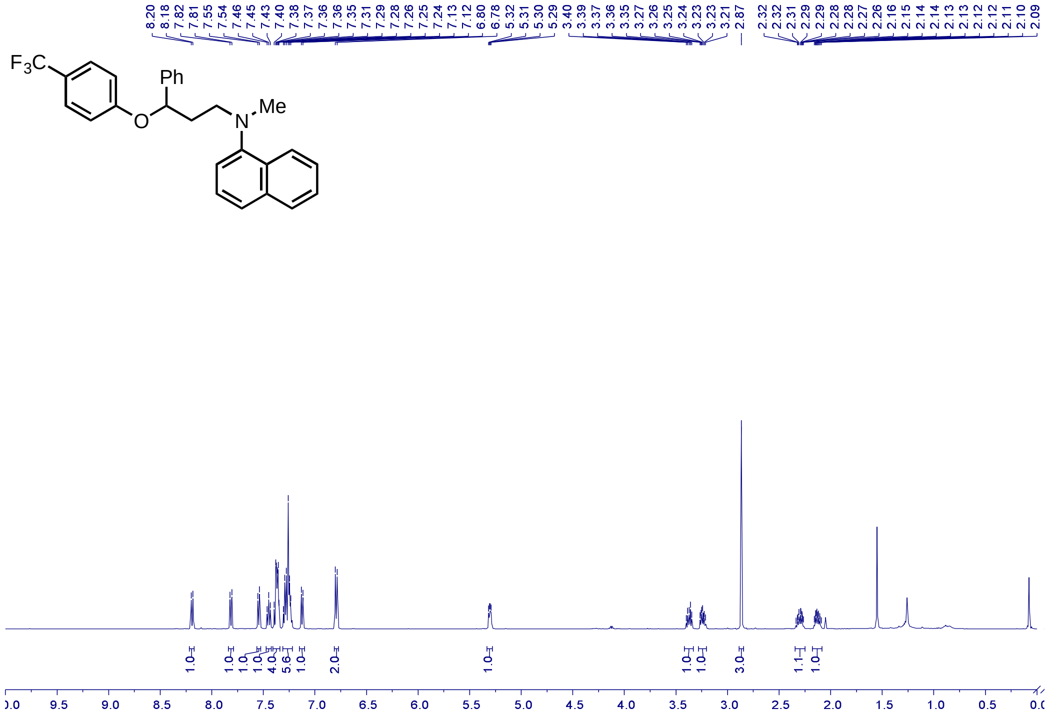

3aw – ^{13}C NMR (126 MHz, $CDCl_3$)

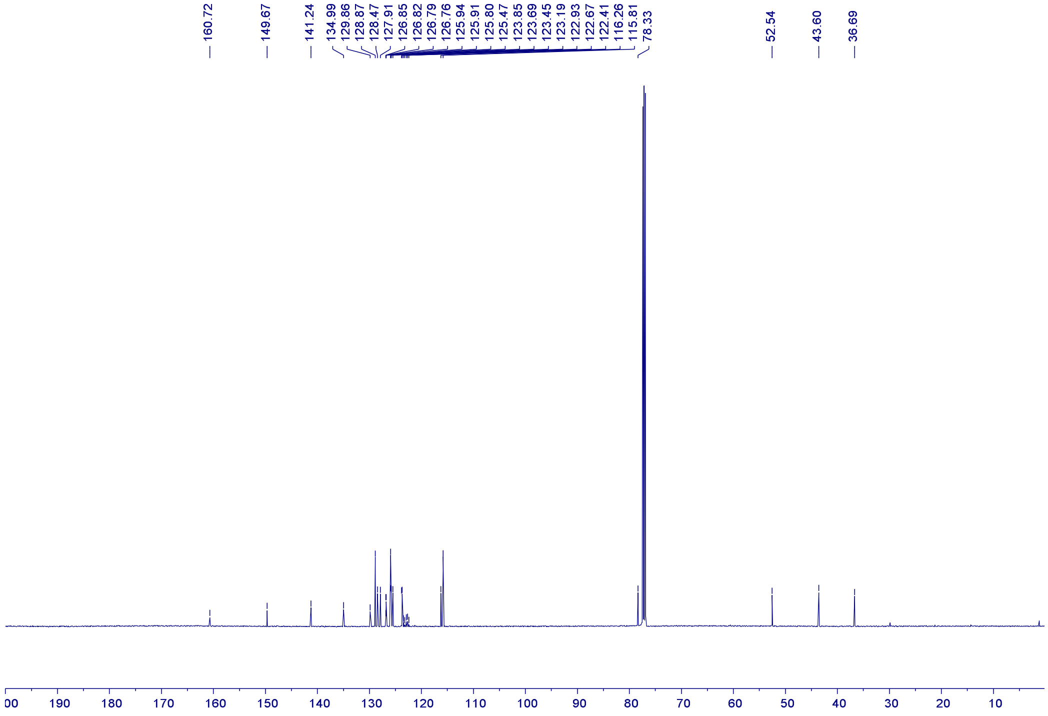

3aw – ^{19}F NMR (375 MHz, $CDCl_3$)

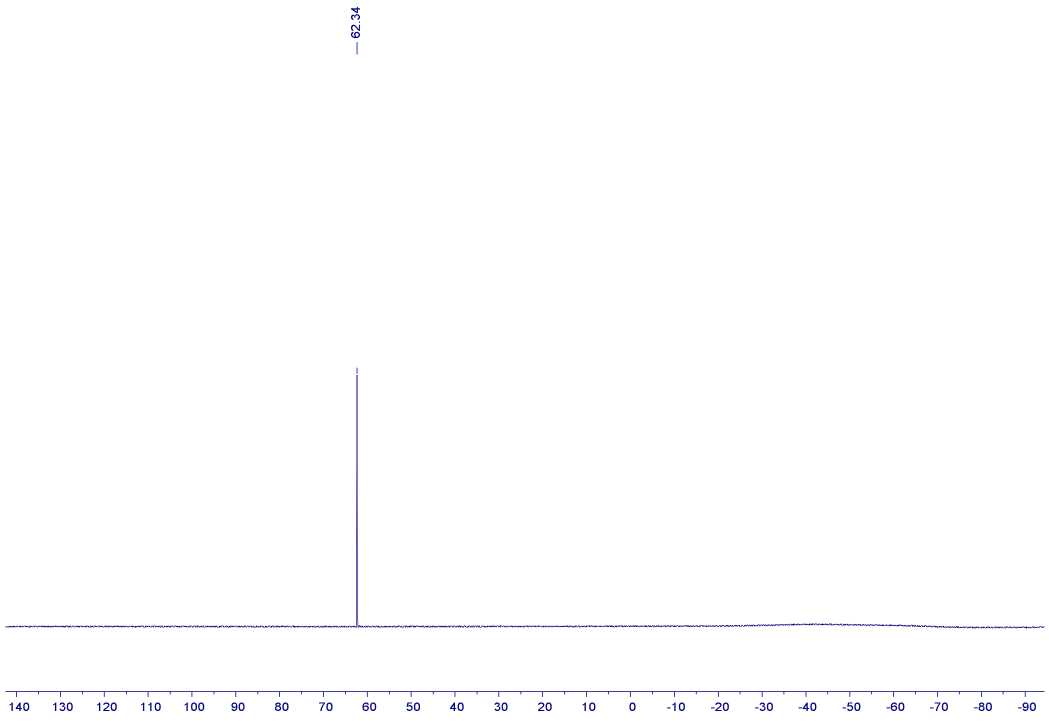

3ax – ^{1}H NMR (400 MHz, $CDCl_3$)

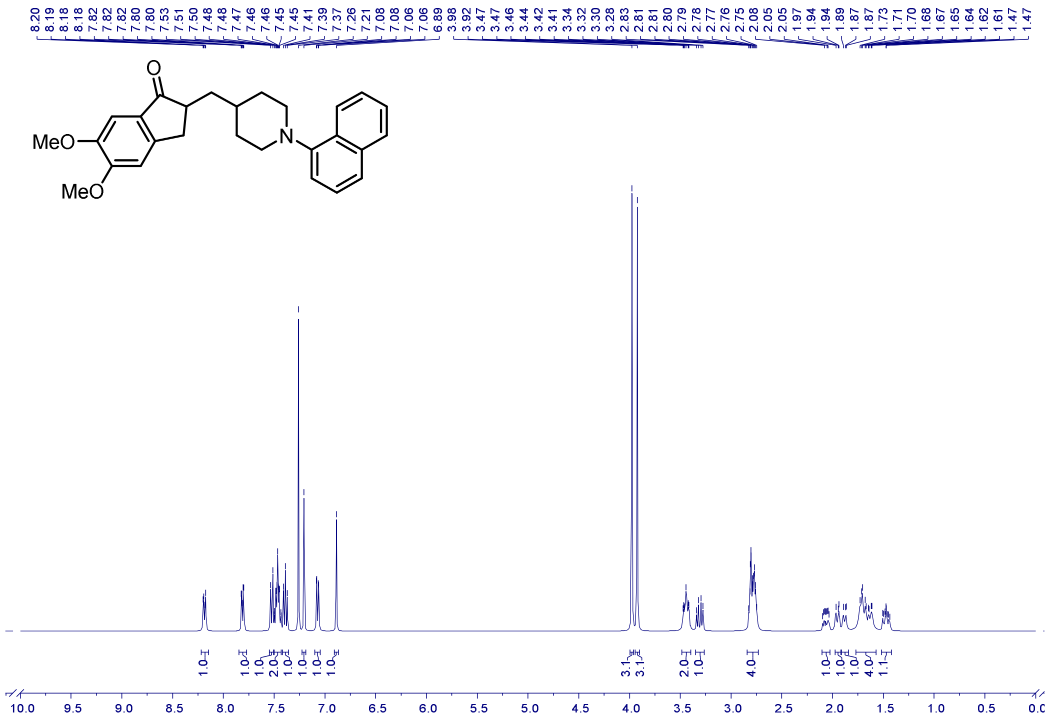

3ax – ^{13}C NMR (101 MHz, $CDCl_3$)

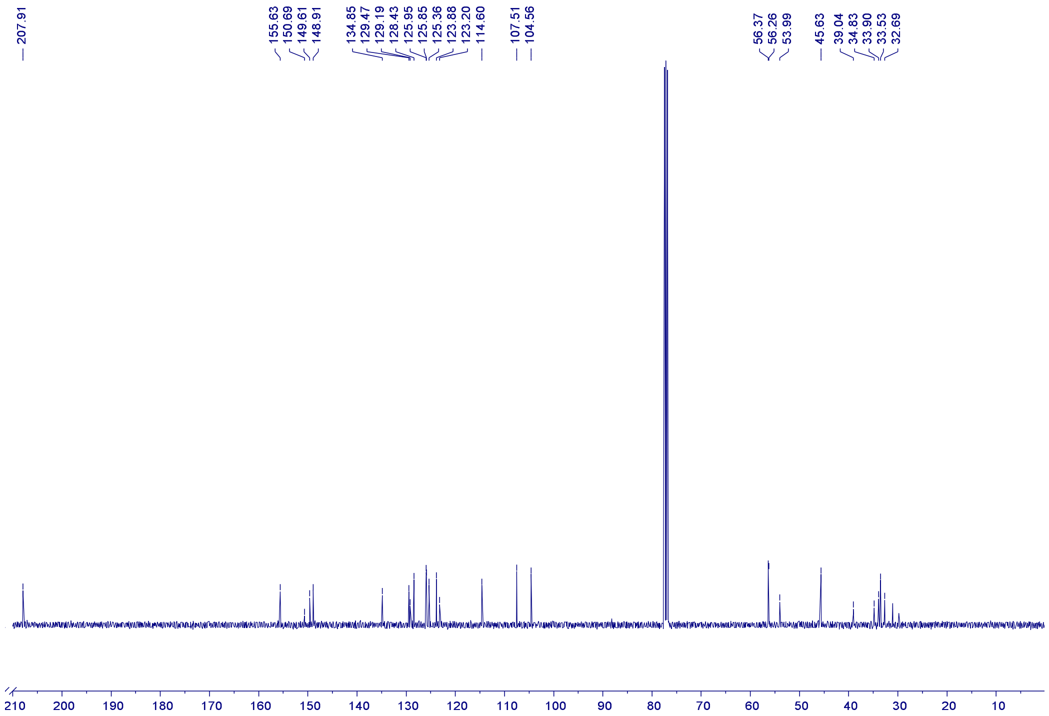

3ay – ^{1}H NMR (500 MHz, $CDCl_3$)

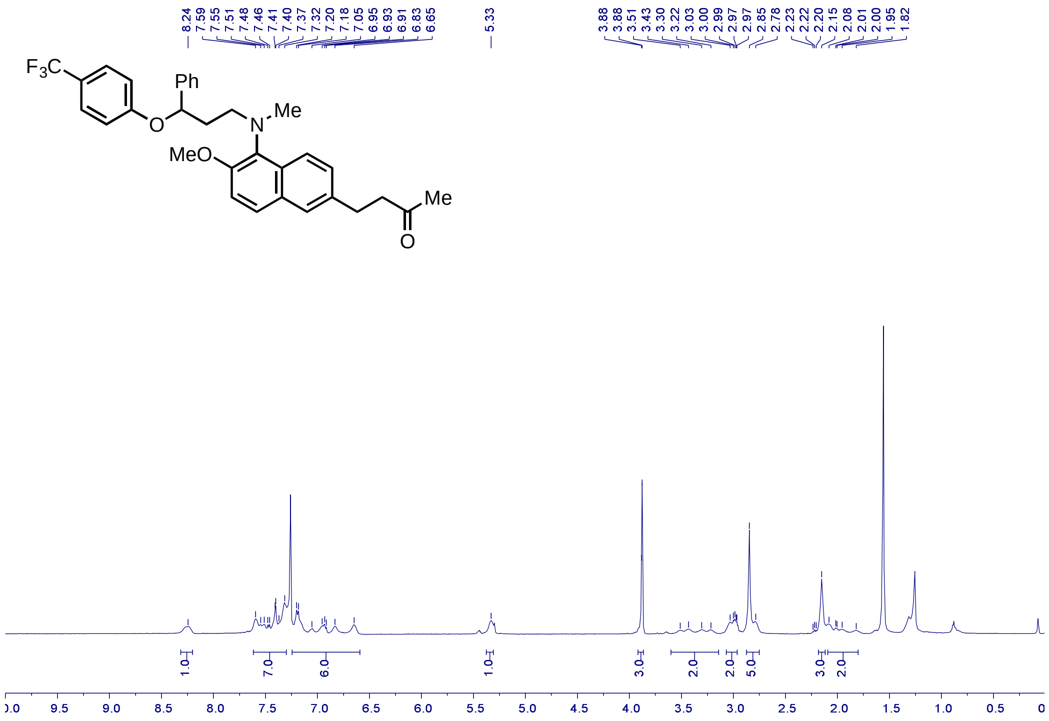

3ay – ^{13}C NMR (150 MHz, $CDCl_3$)

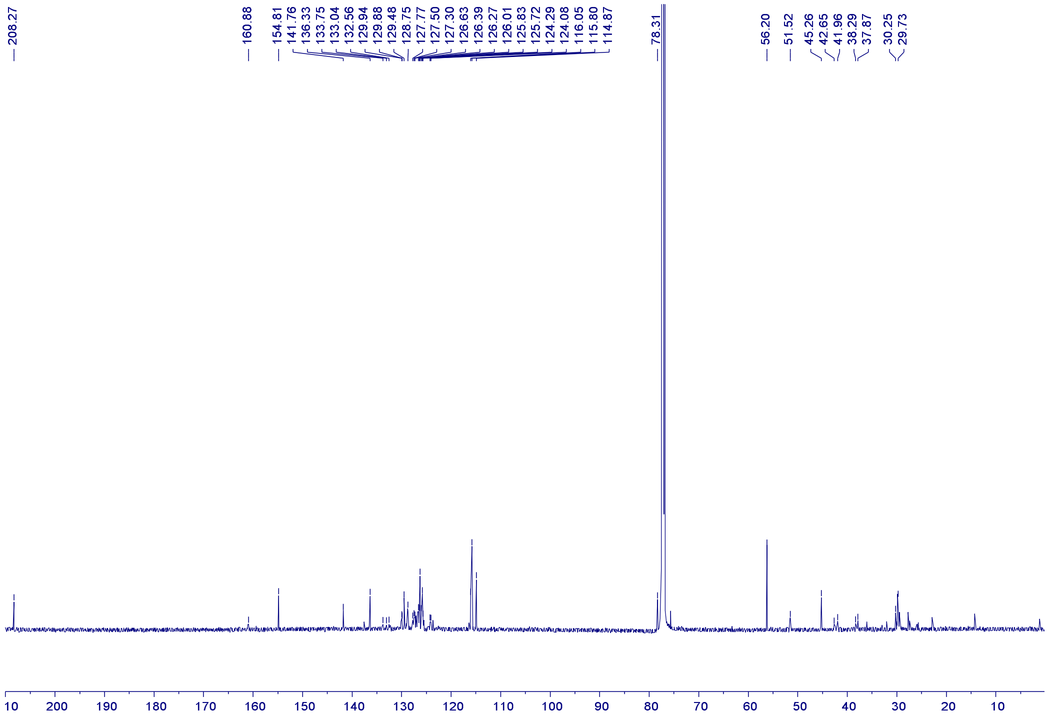

3ay – ^{19}F NMR (376 MHz, $CDCl_3$)

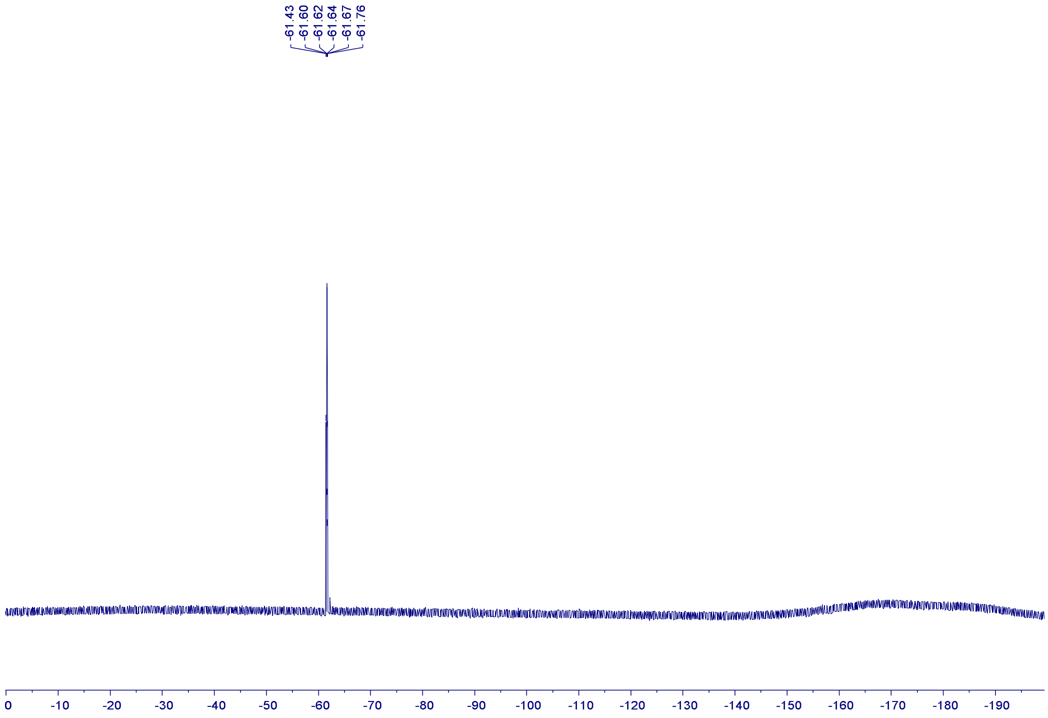

3az – ^{1}H NMR (500 MHz, $CDCl_3$)

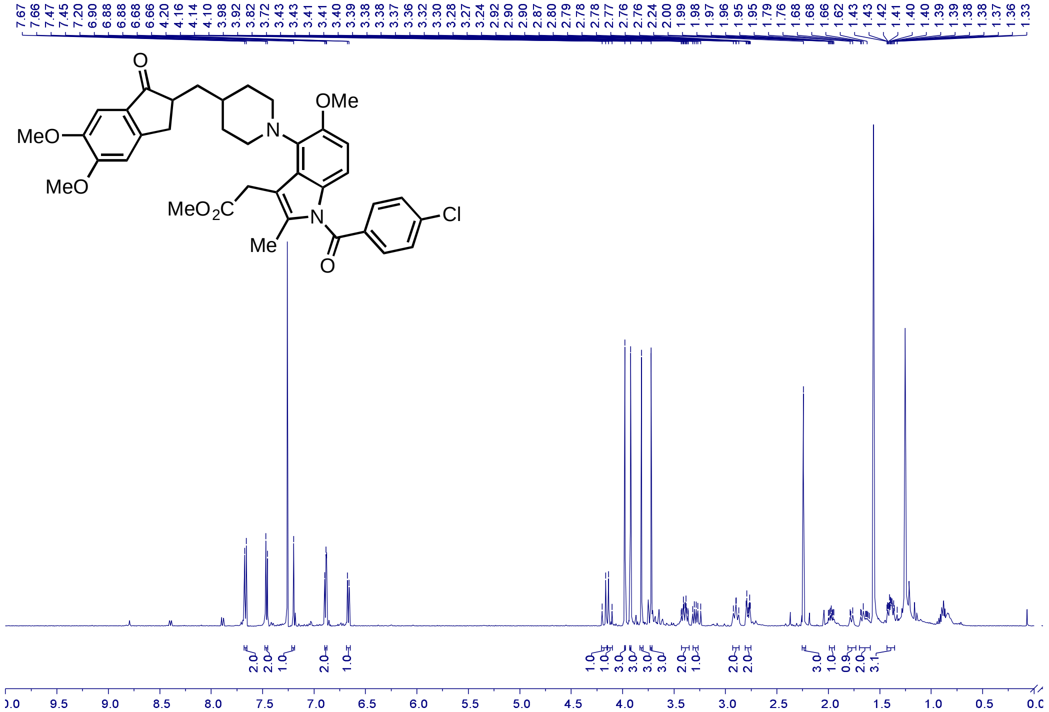

3az – ^{13}C NMR (150 MHz, $CDCl_3$)

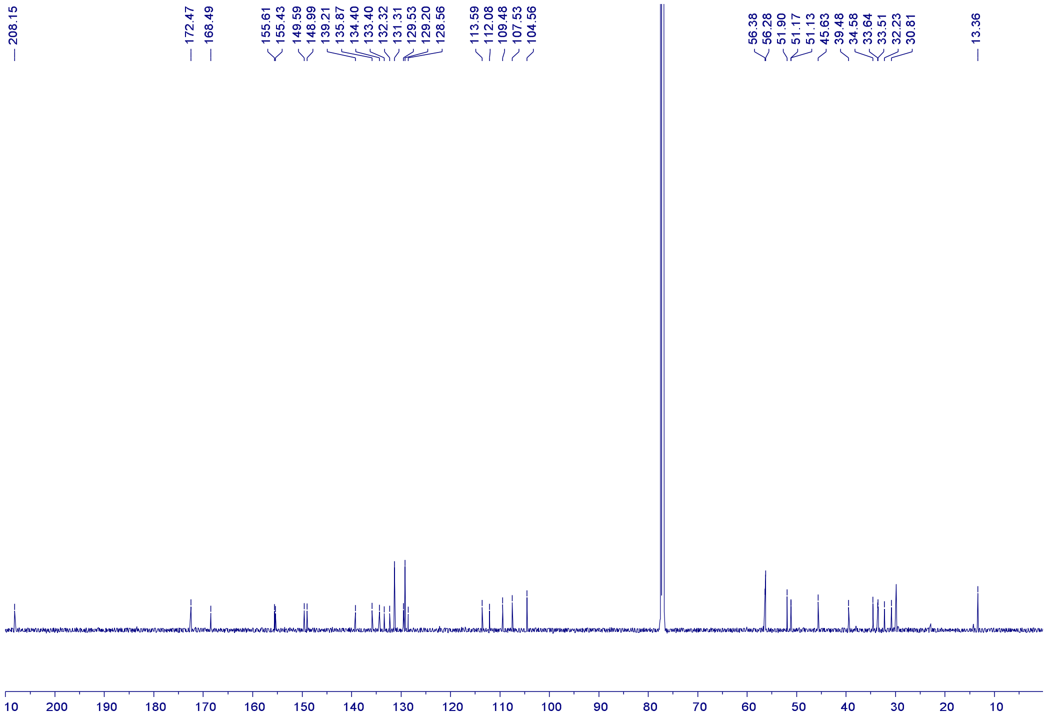

7 References

(1) Luo, B.; Gao, J.-M.; Lautens, M., *Org. Lett.* **2016,** *18*, 4166.

(2) Ito, Y.; Ueda, M.; Takeda, N.; Miyata, O., *Chem. Eur. J.* **2016,** *22*, 2616.

(3) McManus, J. B.; Nicewicz, D. A., *J. Am. Chem. Soc.* **2017,** *139*, 2880.

(4) Girard, S. A.; Hu, X.; Knauber, T.; Zhou, F.; Simon, M.-O.; Deng, G.-J.; Li, C.-J., *Org. Lett.* **2016,** *14*, 5606.

(5) Liu, F.; Zhu, Y.-R.; Song, L.-G.; Lu, J.-M., *Org. Biomol. Chem.* **2016,** *14*, 2563.

(6) Ackermann, L.; Born, R.; Spatz, J. H.; Meyer, D., *Angew. Chem. Int. Ed.* **2005,** *44*, 7216.

(7) Nguyen, M. H.; A. B. Smith, I., *Org. Lett.* **2013,** *15*, 4872.

(8) Vantourout, J. C.; Miras, H. N.; Isidro-Llobet, A.; Sproules, S.; Watson, A. J. B., *J. Am. Chem. Soc.* **2017,** *139*, 4769.

(9) Barton, D. H. R.; Donnelly, D. M. X.; Finet, J.-P.; Guiry, P. J., *J. Chem. Soc., Perkin Trans. 1* **1991**, 2095.

(10) Sasaki, S.; Niko, Y.; Igawa, K.; Konishi, G.-I., *RSC Adv.* **2014,** *4*, 33474.

(11) (a) Zhu, F.; Wang, Z.-X., *Adv. Synth. Catal.* **2013,** *355*, 3694; (b) Hino, T.; Nakagawa, M.; Hashizume, T.; Yamaji, N.; Miwa, Y.; Tsuneoka, K.; Akaboshi, S., *Tetrahedron* **1971,** *27*, 775.

(12) Afanasyev, O. I.; Tsygankov, A. A.; Usanov, D. L.; Chusov, D., *Org. Lett.* **2016,** *18*, 5698.

(13) Topchiy, M. A.; Dzhevakov, P. B.; Rubina, M. S.; Morozov, O. S.; Asachenko, A. F.; Nechaev, M. S., *Eur. J. Org. Chem.* **2016**, 1908.

(14) Zhang, Y.; Yang, X.; Yao, Q.; Ma, D., *Org. Lett.* **2012,** *14*, 3056.

(15) Brand, J. P.; Waser, J., *Org. Lett.* **2012,** *14*, 744.

(16) (a) Fukuzumi, S.; Chiba, M.; Tanaka, T., *Chem. Lett.* **1989**, 31; (b) Fukuzumi, S.; Ishikawa, K.; Hironaka, K.; Tanaka, T., *J. Chem. Soc., Perkin Trans. 2* **1986**, 751.

(17) Roth, H. G.; Romero, N. A.; Nicewicz, D. A., *Synlett* **2016,** *27*, 714.

(18) Rueda-Becerril, M.; Mahé, O.; Drouin, M.; Majewski, M. B.; West, J. G.; Wolf, M. O.; Sammis, G. M.; Paquin, J.-F., *J. Am. Chem. Soc.* **2014,** *136*, 2637.

(19) C. R. Bock, J. A. C.; Gutierrez, A. R.; Meyer, T. J.; Whitten, D. G.; Sullivan, B. P.; Nagle, J. K., *J. Am. Chem. Soc.* **1979,** *101*, 4815.

(20) (a) Dempsey, J. L.; Winkler, J. R.; Gray, H. B., *J. Am. Chem. Soc.* **2010,** *132*, 1060; (b) Hidalgo-Acosta, J. C.; Méndez, M. A.; Scanlon, M. D.; Vrubel, H.; Amstutz, V.; Adamiak, W.; Opallo, M.; Girault, H. H., *Chem. Sci.* **2015,** *6*, 1761.

(21) Gualandi, A.; Marchini, M.; Mengozzi, L.; Natali, M.; Lucarini, M.; Ceroni, P.; Cozzi, P. G., *ACS Catal.* **2015,** *5*, 5927.

(22) Fukuzumi, S.; Chib, M., *J. Chem. Soc., Perkin Trans. 2* **1991**, 1393.

(23) Pavlishchuk, V. V.; Addison, A. W., *Inorg. Chem. Acta* **2000,** *298*, 97.

(24) Caspar, J. V.; Meyer, T. J., *J. Am. Chem. Soc.* **1983,** *105*, 5583.

(25) Frisch, M. J.; Trucks, G. W.; Schlegel, H. B.; Scuseria, G. E.; Robb, M. A.; Cheeseman, J. R.; Scalmani, G.; BaronE, V.; Mennucci, B.; Petersson, G. A.; Nakatsuji, H.; Caricato, M.; Li, X.; Hratchian, H. P.; Izmaylov, A. F.; Bloino, J.; Zheng, G.; Sonnenberg, J. L.; Hada, M.; Ehara, M.; Toyota, K.; Fukuda, R.; Hasegawa, J.; Ishida, M.; Nakajima, T.; Honda, Y.; Kitao, O.; Nakai, H.; Vreven, T.; Jr., J. A. M.; Peralta, J. E.; Ogliaor, F.; Bearpark, M.; Heyd, J. J.; Brothers, E.; Kudin, K. N.; taroverov, V. N.; Keith, T.; Kobayashi, R.; Normand, J.; Raghavachari, K.; Rendell, A.; Burant, J. C.; Iyengar, S. S.; Tomasi, J.; CossI, M.; Rega, N.; Millam, J. M.; Klene, M.; Knox, J. E.; CrossI, J. B.; Bakken, V.; Adamo, C.; Jaramillo, J.; Gomperts, R.; Stratmann, R. E.; Yazyev, O.; Austin, A. J.; Cammi, R.; Pomelli, C.; Ochterski, J. W.; Martin, R. L.; Morokuma, K.; Zakrzewski, V. G.; Voth, G. A.; Salvador, P.; Dannenberg, J. J.; Dapprich, S.; Daniels, A. D.; Farkas, O.; Foresman, J. B.; Ortiz, J. V.; Cioslowski, J.; Fox, D. J., *Gaussian 09* **2013**, revision D.01; Gaussian, Inc.

(26) Parr, R. G.; Yang, W., *J. Am. Chem. Soc.* **1984,** *106*, 4049.

(27) Lu, T.; Chen, F., *J. Comput. Chem.* **2011,** *33*, 580.
